# NRF2 Activation in Trp53;p16-deficient Mice Drives Oral Squamous Cell Carcinoma

**DOI:** 10.1158/2767-9764.CRC-23-0386

**Published:** 2024-02-21

**Authors:** Samera H. Hamad, Rani S. Sellers, Nathan Wamsley, Paul Zolkind, Travis P. Schrank, Michael B. Major, Bernard E. Weissman

**Affiliations:** 1 Lineberger Comprehensive Cancer Center, University of North Carolina at Chapel Hill School of Medicine, Chapel Hill, North Carolina.; 2 Curriculum in Toxicology and Environmental Medicine, University of North Carolina at Chapel Hill School of Medicine, Chapel Hill, North Carolina.; 3 Department of Surgery, Cooper University Hospital, Camden, New Jersey.; 4 Department of Pathology and Laboratory Medicine, University of North Carolina at Chapel Hill School of Medicine, Chapel Hill, North Carolina.; 5 Department of Cell Biology and Physiology, Washington University School of Medicine in St. Louis, St. Louis, Missouri.; 6 Department of Otolaryngology, Washington University School of Medicine in St. Louis, St. Louis, Missouri.; 7 Department of Otolaryngology-Head and Neck Surgery, The University of North Carolina School of Medicine at Chapel Hill, Chapel Hill, North Carolina.

## Abstract

Aberrant activation of the NRF2/NFE2L2 transcription factor commonly occurs in head and neck squamous cell carcinomas (HNSCC). Mouse model studies have shown that NRF2 activation alone does not result in cancer. When combined with classic oncogenes and at the right dose, NRF2 activation promotes tumor initiation and progression. Here we deleted the tumor suppressor genes *p16^INK4A^* and *p53* (referred to as CP mice), which are commonly lost in human HNSCC, in the presence of a constitutively active NRF2^E79Q^ mutant (CPN mice). NRF2^E79Q^ expression in CPN mice resulted in squamous cell hyperplasia or dysplasia with hyperkeratosis in the esophagus, oropharynx, and forestomach. In addition, CPN mice displayed oral cavity squamous cell carcinoma (OSCC); CP mice bearing wild-type NRF2 expression did not develop oral cavity hyperplasia, dysplasia or OSCC. In both CP and CPN mice, we also observed predominantly abdominal sarcomas and carcinomas. Our data show that in the context of p53 and p16 tumor suppressor loss, NRF2 activation serves oncogenic functions to drive OSCC. CPN mice represent a new model for OSCC that closely reflects the genetics of human HNSCC.

**Significance:**

Human squamous cancers frequently show constitutive NRF2 activation, associated with poorer outcomes and resistance to multiple therapies. Here, we report the first activated NRF2-driven and human-relevant mouse model of squamous cell carcinoma that develops in the background of p16 and p53 loss. The availability of this model will lead to a clearer understanding of how NRF2 contributes to the initiation, progression, and therapeutic response of OSCC.

## Introduction

NRF2, also called nuclear factor 2-related factor 2 (NFE2L2), is a main orchestrator of the cellular stress response to reactive oxygen species (ROS), reactive nitrogen species, and electrophilic metabolites and toxins (1–3). If unmitigated, oxidative and electrophilic stress results in DNA, protein, and lipid damage that contributes to many human pathologies, including cellular transformation and cancer (4–6). In the absence of stress, the KEAP1 (Kelch-like ECH-associated protein 1) and CUL3 (Cullin 3) E3 ubiquitin ligase complex promotes NRF2 ubiquitylation and proteasomal degradation, resulting in low, near-undetectable levels of NRF2 protein (3). In the presence of ROS or electrophilic stressors, KEAP1/CUL3-dependent degradation of NRF2 is inhibited, leaving newly transcribed and translated NRF2 free to enter the nucleus and promote the expression of an antioxidant gene expression program (3).

KEAP1 physically binds the ^29^DLG^31^ and ^79^ETGE^82^ motifs in NRF2. Cancer-derived mutations in *NRF2* localize to these residues, resulting in NRF2 stabilization, nuclear localization, and transcriptional activity (7, 8). *NRF2^E79Q^* is one of the most common mutations found in squamous cell carcinomas (SCC), including those of the head and neck, esophagus, lung, and bladder (9–11). Independent of KEAP1 and NRF2 mutation, additional mechanisms result in NRF2 activation in liver cancer, kidney cancer, and breast cancer (1, 12). NRF2 activation is thought to promote cancer initiation and/or progression through oxidative and metabolic stress abatement, metabolic reprogramming, and immune evasion (1, 13–16). Consistent with this notion, NRF2 activity during cancer treatment positively correlates with resistance to standard-of-care radiotherapy, chemotherapy, and possibly immune checkpoint therapy (17–19).

Recent mouse models of cancer show a highly contextualized response to NRF2 activation (4, 16, 20–22). In isolation, loss-of-function mutations in KEAP1 or gain-of-function mutations in NRF2 do not yield cancer (22–25). When combined with the activation of a “classic” oncogene, such as MYC or KRAS^G12D^, NRF2 activation can promote initiation and early progression (22, 26). However, the timing and dosage of NRF2 activity appear critical, as several studies report cancer suppression following high levels of NRF2 activation. For example, lung-restricted NRF2^E79Q^ expression in p53;p16-deficient background promotes *in situ* small cell lung cancer (SCLC) lesions but undergoes silencing in large aggressive SCLC tumors (21). Similarly, in the classic KP model for lung adenocarcinoma driven by Kras^G12D^ activation and p53 loss, moderate NRF2 activation promoted cancer initiation and early progression while strong NRF2 activation blocked progression to advanced cancer (26).

Existing genetically engineered mouse models (GEMM) have revealed important insights in head and neck squamous cell carcinoma (HNSCC) genesis, progression, and therapeutic response (27). However, these models are mostly driven by activating mutations in *Kras*, a gene rarely altered in human HNSCC (9). Therefore, to study the impact of NRF2 activation on HNSCC development, we previously created a *LSL*-*Nrf2^E79Q^* GEMM with inducible expression of a constitutively active NRF2 gene. Targeted expression of this NRF2 mutant in Keratin14 (K14)-expressing cells resulted in squamous cell hyperplasia and hyperkeratosis in the forestomach, esophagus, and oral cavity, as well as loss of epididymal white adipose tissue (23). These phenotypes are consistent with other NRF2 gain-of-function and KEAP1 loss-of-function GEMM studies (28, 29). Notably, expression of NRF2^E79Q^ in K14-positive tissues did not yield tumors, which agrees with other KEAP1 and NRF2 GEMMs (23).

Here we characterize a new GEMM harboring floxed alleles of *Nrf2^E79Q/+^* (referred to as N mice) and the *p16^fl/^^fl^* and *p53^fl/^^fl^* tumor suppressor genes (referred to as CP mice); p53 and p16 are lost in >80% of HNSCC. The mice also possess a *K14-Cre^ERTAM2^* allele to allow tamoxifen (TMX)-inducible expression in K14-positive epithelial tissues. After intraperitoneal inoculation of TMX, we observed hyperplasia in the esophagus, oropharynx, and forestomach in CPN mice. Unexpectedly, we also observed squamous cell carcinoma of the oral cavity (OSCC) in CPN mice; CP mice harboring wild-type NRF2 did not develop OSCC. Thus, we have generated a unique GEMM, where constitutive NRF2 signaling is required for the development of OSCC in the context of p53 and p16 loss. Given the high frequency of mutations in these genes in HNSCC, this GEMM represents a new genetically relevant model of human HNSCC. Our data also establish that NRF2 activation can promote cancer initiation and progression in the absence of a classic oncogene (e.g., KRAS). We also find altered expression of several immune markers within the OSCC and in the histologically normal oral epithelium in CPN mice compared with CP mice, supporting the paradigm that NRF2 activation may reprogram the immune microenvironment.

## Materials and Methods

### GEMM Study Design

The generation of the CPN *K14-Cre^ERTAM2^*; *Trp53^fl/fl^*; *p16^fl/fl^*; *LSL-Nrf2^E79Q/WT^* GEMM used in this study was described previously (21, 23). The *p16^fl/^^fl^* allele is specific for this gene and maintains an intact *p14^ARF^* allele (30). Importantly, this GEMM has been backcrossed for >15 generations at the time of these experiments. The resulting fixed genetic background was confirmed by mouse universal genotyping array (MUGA) analysis (31). Genotypes from tail snips of all mice, before and after tumor initiation, was performed by Celplor using primers presented in Hamad and colleagues (21).

To assess the effects of NRF2 activation on tumor development in K14-positive tissues, we used two experimental groups, CP mice (*K14-Cre^ERTAM2^*; *Nrf2^WT/WT^*; *Trp53^fl/fl^*; *p16^fl/^^fl^*; *n* = 20, 10♀ and 10♂) and CPN mice (*K14-Cre^ERTAM2^*; *Trp53^fl/fl^*; *p16^fl/fl^*; *LSL-Nrf2^E79Q/WT^*; *n* = 24, 11♀ and 13♂), possessing a single *LSL-Nrf2^E79Q/WT^* allele. The number of mice per group was based on a calculated one-sided *P* value < 0.05, Fisher exact test (FET) had 88% power with *N* = 20 mice per group, we used 50 females verses 50 males. We treated all mice with TMX (100 mg/kg × 5 days, 100 µL i.p.) using intraperitoneal inoculation at 6–8 weeks of age to activate the heterozygous *Nrf2^E79Q^* allele and inactivate the *Trp53* and *p16* alleles in all K14-positive tissues. The mice were then monitored by the University of North Carolina-Lineberger Comprehensive Cancer Center (UNC-LCCC) Animal Studies Core for weight and health status. Mice were sacrificed when they showed signs of distress (i.e., labored breathing and/or weight loss or other body conditions such as fur ruffling, difficulty in walking and hunched posture) or at the end timepoint (60 weeks). Upon sacrifice, we harvested heads, esophagus, stomach, and any tissues with macroscopic findings for further characterization.

We previously generated OSCC in the CP mice by exposure to 4-nitroquinoline 1-oxide (4NQO) for 8 weeks (20 µg/mL in drinking water), 3 days after treatment with TMX (100 mg/kg × 5 days, 100 µL i.p.; ref. 32). We also performed a control experiment to confirm the activation of *Nrf2^E79Q^* mutant allele in the CPN mice. For this experiment, we treated CP and CPN mice as above and sacrificed the mice at 6 weeks after the TMX treatment.

### Compliance with Ethical Standards

This study was conducted after approval by Institutional Animal Care and Use Committee at the University of North Carolina at Chapel Hill (Chapel Hill, NC; Protocol # 19-242.0).

### Tissue Processing, Hematoxylin and Eosin, and IHC Labeling

Harvested tissues were immersion fixed in 10% neutral buffered formalin at room temperature. Tissues were then sent to the UNC-LCCC Pathology Services Core for paraffin embedding, sectioning, and staining with hematoxylin and eosin (H&E). IHC of tissue samples was performed as previously described on the Ventana Discovery Ultra Automated IHC platform using the following primary antibodies: NRF2 [Abcam, catalog no. ab137550, RRID:AB_2687540 (1:500)]; Pan-cytokeratin [PNCK; Z0622, Dako (1:500)]; and Vimentin [5741, Cell Signaling Technology (1:500)] (33). Tissue slides were incubated with Discovery OmniMap anti-Rabbit horseradish peroxidase (760–4311) for 32 minutes at room temperature, treated with 3,3′-diaminobenzidine (DAB) and counterstained with hematoxylin. While assessing the tumors, Dr. Sellers, the veterinary pathologist, was blinded from the mice genotypes.

### Targeted Mass Spectrometry Analysis

Proteins were extracted from formalin-fixed paraffin-embedded tissues as described in Wamsley and colleagues (34). Briefly, specimens were deparaffinized using Xylenes and sequential ethanol washes, extracted in a lysis buffer of 2,2,2-trifuoroethanol and 100 mmol/L Tris-HCl (pH 8.0) at 50% (v/v), and then digested using Lysyl endopeptidase (Wako Chemicals, 12902541) and trypsin (Promega, PR-V5113). Peptides were desalted by SDB-RPS spin columns (Affinisep, Spin-RPS-M.T1.96) and quantified by a bicinchoninic acid protein assay (Thermo Fisher Scientific, catalog no. 23225).

A total of 1 µg of endogenous tryptic peptides per run were separated by reverse-phase nano-high performance liquid chromatography (HPLC) and analyzed using an Orbitrap Eclipse Tribrid mass spectrometer (Thermo Fisher Scientific) with FAIMS Pro, as described previously (34). A custom Optimized-Internal-Standard Parallel Reaction Monitoring targeted mass spectrometry (OIS-PRM) method was used as reported previously (34). Stable isotope labeled (SIL) internal standard peptides are cataloged in Supplementary Table S1 and were injected at a nominal abundance 40 fmol each for every 1 µg of endogenous peptide. Optimal FAIMS CVs between −30 and −80 were selected for each SIL peptide based on maximal peak area in survey analyses. The Thermo Fisher Scientific Instrument Application Programming Interface was used to control dynamic switching of FAIMS CVs throughout each run. Peak area ratios and chromatogram plots for internal standard triggered parallel reaction monitoring (IS-PRM) data were generated using an in-house tool available on github (https://github.com/nwamsley1/AutoPRM.jl).

### Human OSCC RNA-sequencing Data Analysis

To evaluate The Cancer Genome Atlas (TCGA) Mutational Landscape, the R maftools package was used to summarize and visualize variant calls (35, 36). Copy-number calls (Gistic) were obtained via the Broad Firehose Portal (20). Focal deletions (Gistic −2) and focal amplifications (+2) were also considered (20, 37). Variant calls were downloaded using the R TCGA-biolinks package; calls performed with VarScan (38) were used for all analyses. NRF2 splice variant calls were used without modification as reported previously (39).

To analyze OSCC patient survival, clinical data were downloaded from the Broad Firehose Portal (20), and unified with additional clinical data from Liu and colleagues (40). Human papillomavirus (HPV)-positive cases were identified and excluded on the basis of metadata available through the Broad Firehose Portal (20). Tumors from “Alveolar ridge,” “buccal mucosa,” “floor of mouth,” “oral cavity,” and “oral tongue” subsites were all considered oral cavity tumors, and all are included in this study (Supplementary Table S2). Survival statistics were generated with the R survival package (v3.2-7) and visualized with the R Survminer package (0.4.8). We used *P* value to present the log-rank test results.

### Statistical Analysis

We used GraphPad Prism 8 software (GraphPad Prism, RRID:SCR_002798) to present the data. FET and Gehan–Breslow–Wilcoxon tests were used to identify the differences between the CP and CPN mice. A *P* value less than 0.05 (typically ≤ 0.05) was considered statistically significant. For mass spectrometry (MS) data analysis, a principal component analysis (PCA) was performed on the protein expression data. If more than half the values for any protein were missing, that protein was dropped from the PCA. The position of each tumor core along the first principal component of these data was considered the NRF2 activity score. Missing values were imputed as the lowest non-missing value observed across the entire experiment for each respective protein. *P* values for box-and-whisker plots were calculated with a Mann–Whitney U test and adjusted by a Benjamini–Hochberg procedure (41). Spearman rank order correlation for scatter plots of protein abundance is reported with *P* values adjusted by a Benjamini–Hochberg procedure (41).

### Data Availability

Organizational code/scripts are available on GitHub (https://github.com/nwamsley1/AutoPRM.jl) and (https://github.com/TravisParkeSchrank/Hamad_Mm_NRF2_OralCav_TCGA). The MS proteomics data have been deposited to the ProteomeXchange Consortium via the PRIDE partner repository with the dataset identifier PXD048887 (https://ftp.pride.ebi.ac.uk/pride/data/archive/2024/01/PXD048887). The GEMM generated in this study is available upon request to the corresponding authors.

## Results

To dissect the role of NRF2-active signaling in tumor development in a GEMM possessing mutations frequently found in human HNSCC, we generated two groups of mice—*Tg(Krt14-cre^ERTAM2^)*; *Trp53^fl/^^fl^*; *p16^fl/fl^*; *LSL-Nrf2^WT/WT^* (CP mice) and *Tg(Krt14-cre^ERTAM2^)*; *Trp53^fl/fl^*; *p16^fl/fl^*; *LSL-Nrf2^E79Q/^^WT^* (CPN mice). We previously reported that in CP and CPN mice, inhalation of Adeno-Cre led to the development of SCLC with no appearance of SCC lesions (21). To determine whether NRF2 activation could drive tumor development in any K14-positive tissue, we took an agnostic approach by treating the mice with TMX intraperitoneally at 6–8 weeks of age. After administration of TMX, we monitored mice up to 14 months for tumor development or other disease processes. We confirmed the activation of the *Nrf2^E79Q^* allele in the CPN mice by NRF2 IHC 6 weeks after TMX treatment (Supplementary Table S3). We also observed oral cavity, esophageal, and stomach epithelial hyperplasia with hyperkeratosis in the CPN mice at this timepoint (Supplementary Table S3). Statistically significant differences in overall survival between the experimental groups were not observed (Table 1; Supplementary Fig. S1A). However, sex-specific and genotype-specific survival differences were observed. Male CPN mice showed decreased survival as compared with CP mice due to sacrifice associated with weight loss (Supplementary Fig. S1B–S1D). We found inflammatory eye disease, necessitating early sacrifice, in all female CP mice (10/10), while only 1/10 male CP mice showed this problem. Interestingly, only 3 out of 13 female CPN mice and none of the male CPN mice (0/11) developed this eye phenotype.

**FIGURE 1:**
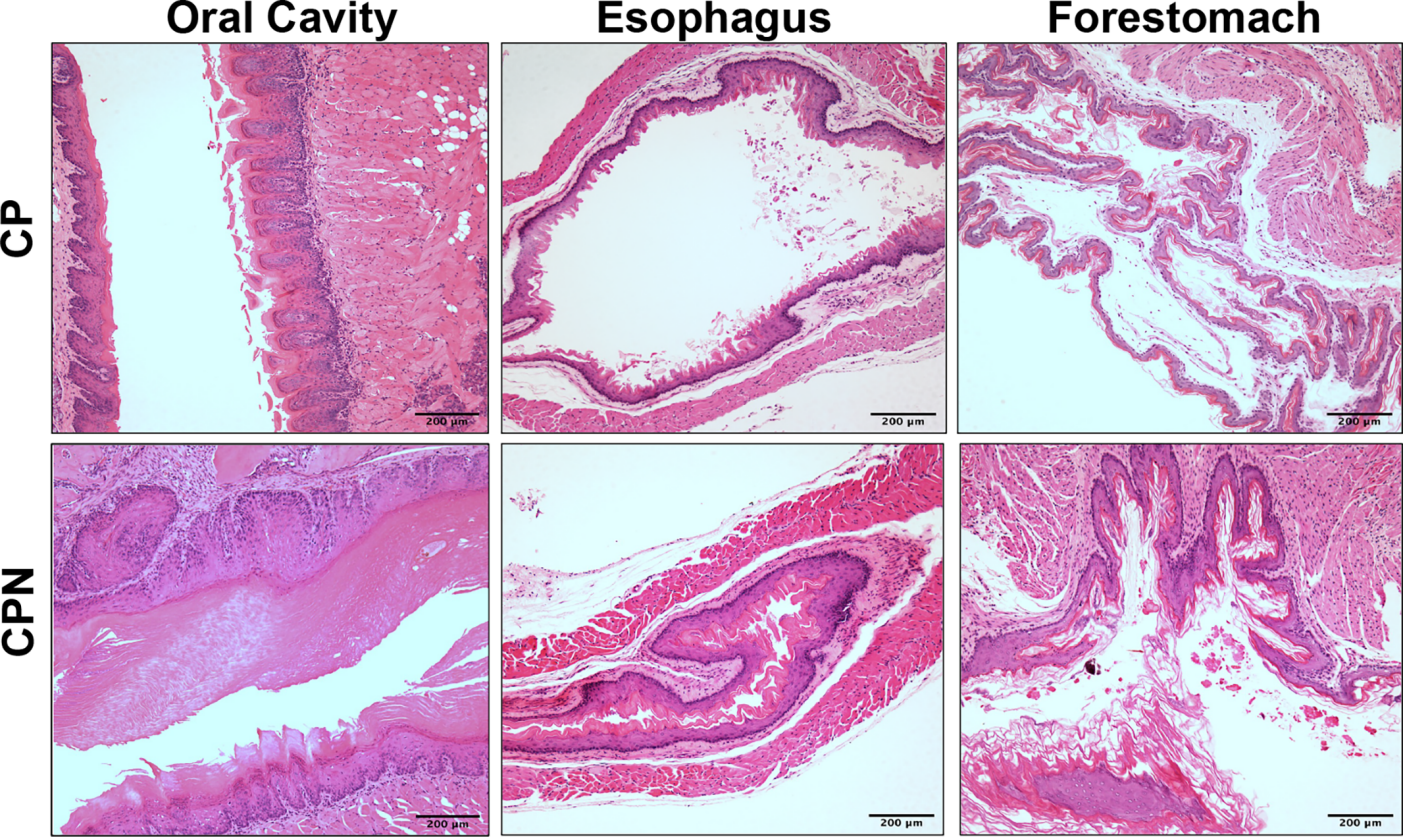
Examples of upper gastrointestinal tract from CP and CPN mice. Example of oral cavity, esophagus, and forestomach of CPN and CPN mice stained for H&E.

In agreement with previous reports of NRF2 active mouse models, NRF2 activity in the CPN mice resulted in hyperkeratosis and hyperplasia of the upper gastrointestinal tract and oral cavity (Table 1; Fig. 1). In addition, we observed carcinomas of the ear in both genotypes, which likely evolved in response to irritation caused by the metal ear tags used to identify individual mice (Supplementary Fig. S2; ref. 42). Both CP and CPN mice also developed spindle cell sarcomas, a common tumor in Trp53 and p16 knockout mice (30, 43), on the ear, salivary gland, neck, legs, lung, abdomen, bladder, and uterus (Supplementary Fig. S3; Supplementary Table S4). We also observed a smaller number of carcinomas on the neck, skin, and salivary gland (Supplementary Table S4). There was no significant difference between the number of these tumors developed by CPN (8/24) and CP (10/20) mice (*P* = 0.13 by FET).

**TABLE 1:** Classification of abnormal growth in CP and CPN mice

Genotype	Sex (*n*)	Survival—weeks after tamoxifen Mean ± SD (range)	Oral, esophagus, and forestomach hyperplasia and hyperkeratosis	CIS	OSCC
CP *n* = 20	Female (*n* = 10)	20 ± 8 (10–30)	0	0	0
	Male (*n* = 10)	29 ± 12 (13–48)	0	0	0
CPN *n* = 24	Female (*n* = 13)	23 ± 13 (10–55)	13	1	5
	Male (*n* = 11)	21 ± 9 (3–30)	10^a^	3	5

NOTE: Statistical analysis by Fisher exact test shows a significant change (*P* = 0.004) in number of OSCC and CIS developed by CPN compared with CP mice.

Abbreviations: CIS: carcinoma *in situ*; OSCC: oral squamous cell carcinoma.

^a^Missing one oral cavity sample.

### NRF2 Activation Drives OSCC Development in the Absence of Trp53 and p16 Expression

**FIGURE 2:**
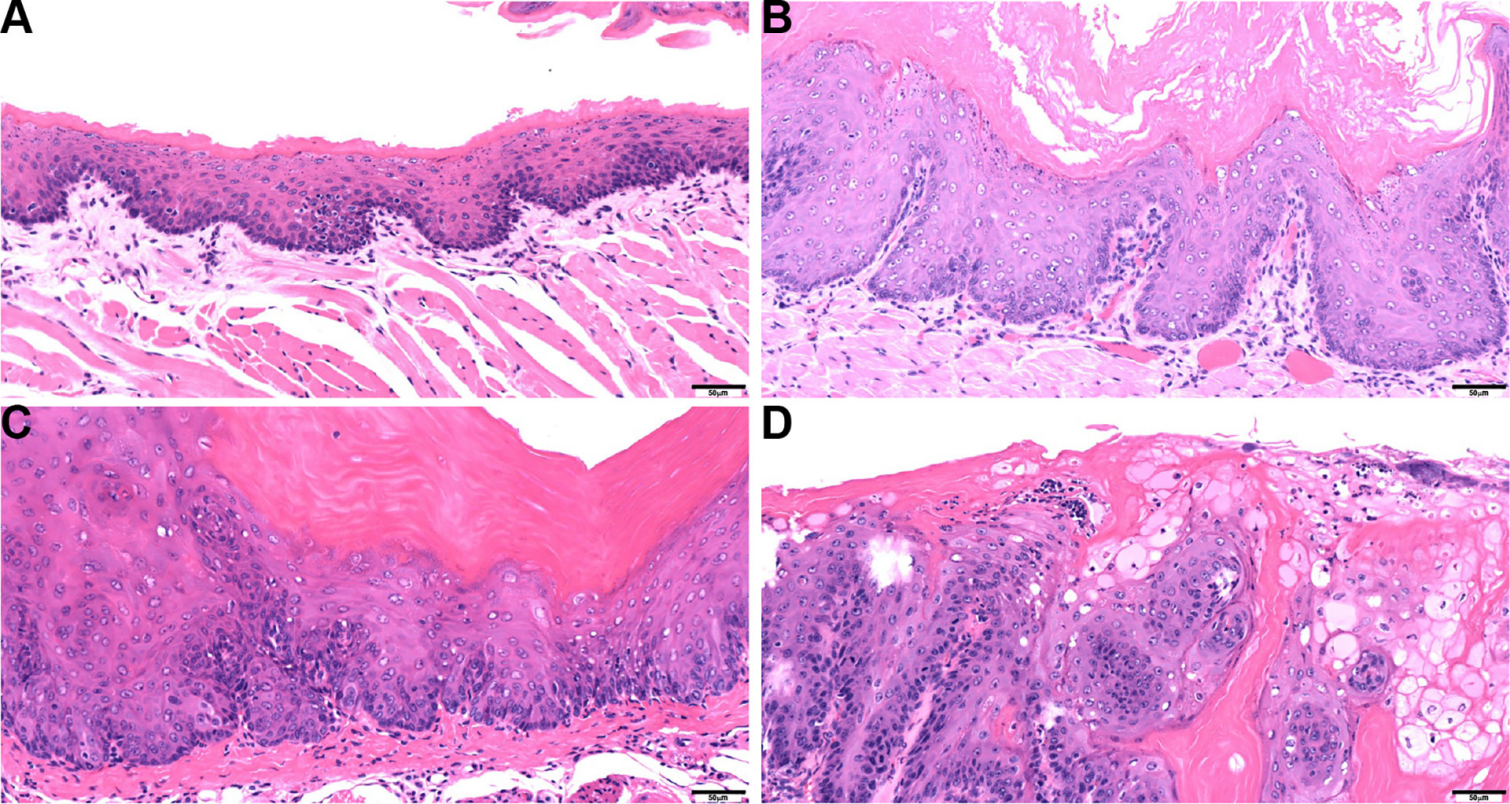
Histologic profile of normal and epithelial lesions of the oral mucosa from CP and CPN mice. Normal epithelium (CP; **A**); epithelial hyperplasia with hyperkeratosis (CPN; **B**); CPN (**C**); and oral squamous cell carcinoma (CPN; **D**). Scale bar = 50 µm. Images were taken using an Olympus DP38 digital camera on an Olympus BX46 microscope.

**FIGURE 3:**
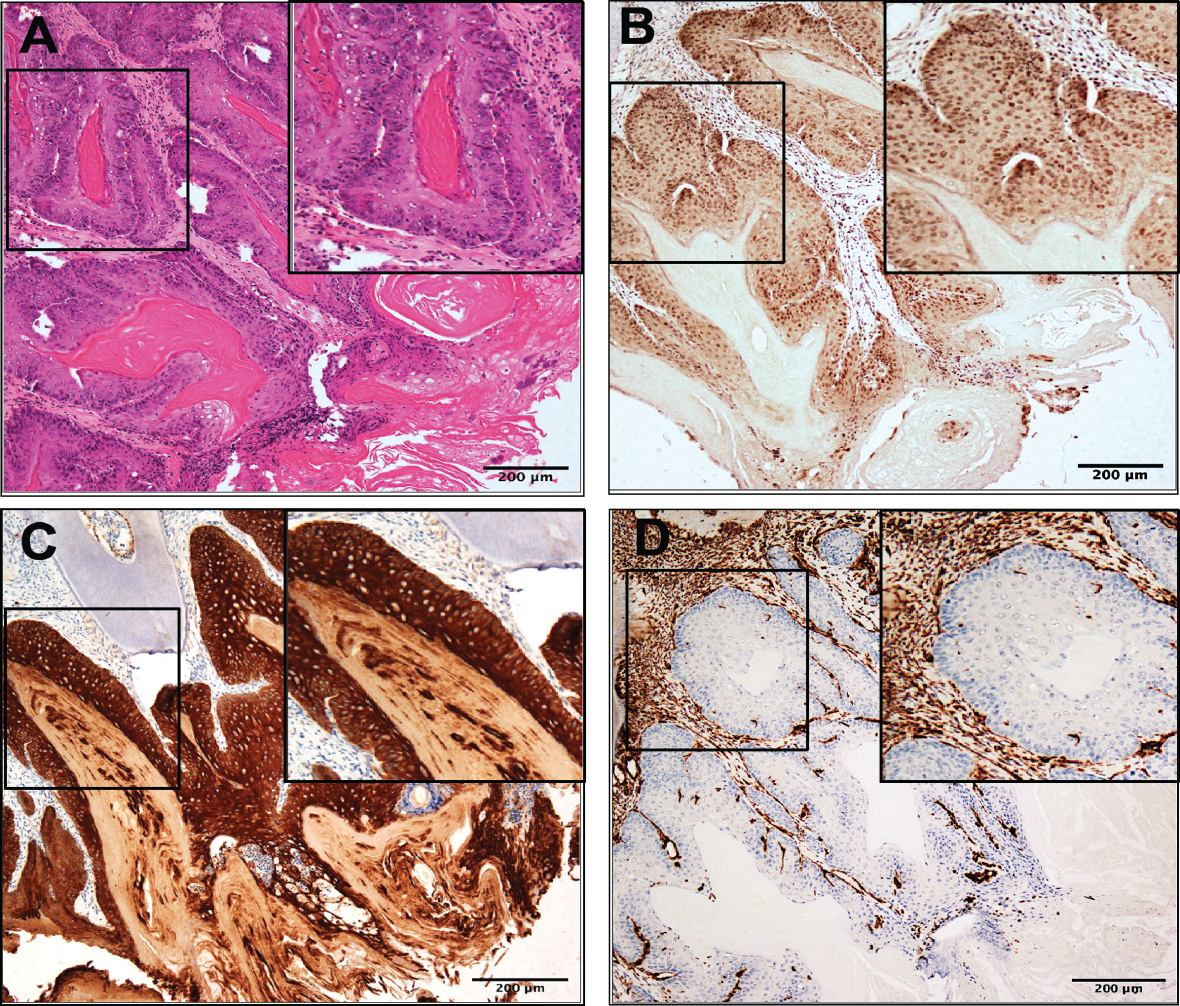
Characterization of oral squamous cell carcinomas from CPN mice. Invasive OSCC from a CPN mouse stained for H&E (**A**). **B,** NRF2. PANCK (**C**) and vimentin (**D**). Scale bar = 200 µm. Images were taken using a BX61-Neville microscope.

Strikingly, beyond the normal and hyperplastic with hyperkeratosis epithelium in the oral cavities of CPN mice (Fig. 2A and B), we discovered tumors, including 4 mice with carcinoma *in situ* (CIS; 1 female and 3 males; Fig. 2C) and 10 mice with SCC (5 females and 5 males; Fig. 2D; Table 1). In contrast, none of the Nrf2^WT/WT^ CP mice developed tumors in the oral cavity. As expected, we observed nuclear NRF2 expression in CPN OSCC by IHC (Fig. 3). We confirmed the epithelial origin of these tumors by demonstrating expression of cytoplasmic PANCK, an epithelial cell marker, and the absence of vimentin, a mesenchymal cell marker. We also observed the presence of keratin pearls, consistent with the histology of SCC.

### Proteomic Characterization of Oral Cavity Tissues from CP and CPN Mice

**FIGURE 4:**
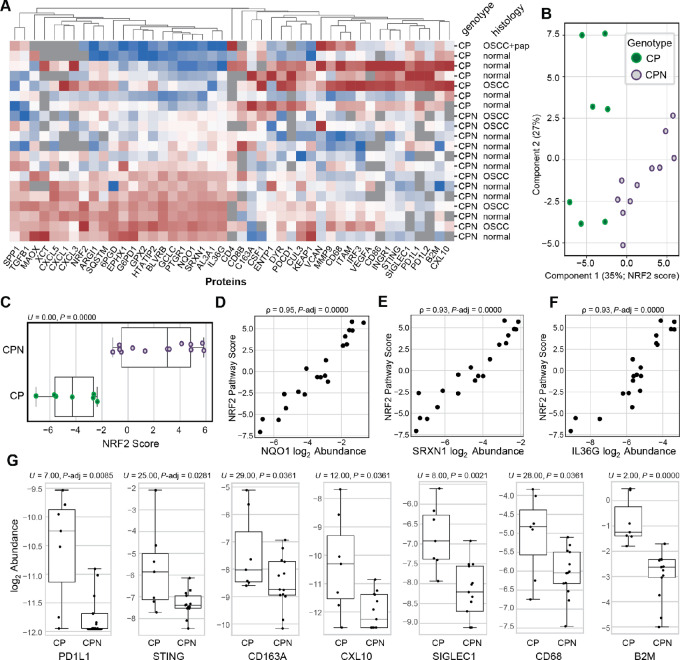
Targeted proteomic analyses of oral cavity tissues from CP and CPN mice. **A,** Heat map of protein expression from oral tissues of CP (*K14-Cre^ERTAM2^*; *p16^−^^/^^−^*; *p53^−^^/^^−^*) and CPN (*K14-Cre^ERTAM2^*; *p16^−^^/^^−^*; *p53^−^^/^^−^*; *NRF2^E79Q/WT^*) mice, with columns normalized by Z-scores; protein abundances were measured by IS-PRM. See also Supplementary Table S5. **B,** PCA plot from protein abundances shown in A; the first two principal components explained 35% and 27% of the total variance respectively. **C,** Plot of the NRF2 score in CP and CPN mice. The score was calculated using 10 NRF2 targets within the first principal component. **D,** Correlation of the NQO1 protein expression with the NRF2 pathway score. **E,** Correlation of the SRXN1 protein expression with the NRF2 pathway score. **F,** Correlation of the IL36G protein expression with the NRF2 pathway score. For correlations of all proteins correlated with the NRF2 score, see Supplementary Fig. S4. **G,** Box-and-whisker plots of protein abundance in CP and CPN oral cavity tissues. For box-and-whisker plots of abundance for all proteins, see Supplementary Fig. S5. Adjusted *P* values were calculated with a Mann–Whitney U test. The normal tissue from the oral cavity of the CPN mice showed hyperplasia with hyperkeratosis as shown in Fig. 2B.

To further validate and extend the IHC characterization, we used our recently reported OIS-PRM approach to quantify 68 selected proteins in tumor and normal murine oral cavity tissues (34). This panel of targeted proteins included NRF2, KEAP1, and CUL3; 10 canonical NRF2 transcriptional targets; and 54 cytokines and immune markers (Supplementary Table S1). Histologically normal tissue from CP mice and normal and OSCC tissues from CPN mice were analyzed. In addition, we characterized two OSCCs arising in 4NQO carcinogen-treated CP mice. Unsupervised hierarchical clustering revealed that irrespective of tissue histology, NRF2 protein and its target genes were significantly increased in CPN mice as compared with CP and CP/4NQO tissues (Fig. 4A; Supplementary Table S5). The effect of NRF2 activation dominated the first dimension of a PCA (Fig. 4B and C). Plotting protein abundance against a composite NRF2 activity score showed strong correlation for specific proteins, including NRF2 targets NQO1 and SXRN1 (Fig. 4D and E; Supplementary Fig. S4). Consistent with previous reports, IL36G protein expression strongly correlated with NRF2 pathway activity (Fig. 4F; refs. 34, 44, 45). Several immune markers were significantly diminished in CPN mice tissue relative to CP samples, including PD1L1, STING, CD163A, CD68, and B2M (Fig. 4G; Supplementary Figs. S4 and S5). Together, these data are the first to show that constitutive NRF2 activation promotes the development of murine OSCC (*P = 0.004* by FET) and reprograms the proteome and immune microenvironment.

### Activating Mutations Appear in Human OSCC, Associated with Poorer Clinical Outcomes

**FIGURE 5:**
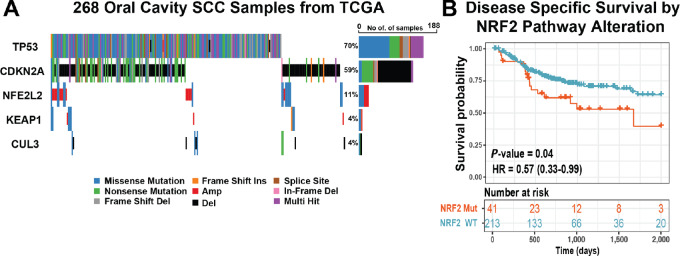
NRF2 activity in human OSCC. TP53, CDKN2A, and NRF2 pathway mutational profiles from 268 HPV—oral cavity squamous cell carcinoma in TCGA. **A,** Waterfall plot—relative frequency and mutational classes. Color—mutational class. **B,** Kaplan–Meier plot—disease specific survival of the 254 patients with HPV—oral cavity squamous cell carcinoma with available outcomes data, stratified by NRF2 pathway alteration status. Red—Altered NRF2 pathway. Blue—wild-type NRF2 pathway. *P* value represents log-rank test. HR—hazard ratio with 95% confidence interval.

Activation of NRF2 occurs frequently in SCC, such as lung, head and neck, and bladder (9–11). Therefore, we examined gene expression from TCGA consortium to determine whether activated NRF2 signaling also appears in human OSCC. In HPV(−) oral cavity SCC (OSCC), we found *NFE2L2*(NRF2), *KEAP1*, and *CUL3* genes were altered in 11%, 4%, and 4% of tumors, respectively (Fig. 5A). *NFE2L2* splice variants can abrogate binding of NRF2 to KEAP1, resulting in NRF2 pathway activation (46). Thus, for OSCC, we found that 43/268 cases (16%) oral cavity cases have one or more pathway activating mutations, CNV, or splice variants. In line with other studies (47, 48), we found that patients with NRF2 pathway alteration had decreased disease-specific survival (Fig. 5B). As such, NRF2 pathway alterations are common in OSCC and are associated with poor clinical outcomes.

## Discussion

Previous studies have established that constitutive activation of NRF2 signaling is observed in squamous cell carcinomas of the head and neck (HNSCC), esophagus, and lung (1, 29).The precise function of NRF2 activation in tumorigenesis remains unresolved, though context dependencies of cancer stage, strength of activation and cooperating genetic mutations are critical (22, 26, 49, 50). Our studies establish a requisite role for NRF2 activation in the development of murine OSCC, specifically in a background of p53 and p16 deficiency. These data raise two important points. First, previous studies have suggested that at high doses, NRF2 activation can inhibit tumor initiation and/or progression in mouse models (21, 25, 26, 51). The appearance of OSCC in CPN mice strongly suggests that heterozygous NRF2^E79Q^ expression provides an efficient dose of NRF2 activity to drive tumor initiation in this GEMM. Whether homozygous NRF2^E79Q^ yields less squamous cancer than the heterozygous state in a CP background remains to be determined. Second, studies examining the effects of activated NRF2 signaling upon tumorigenesis have used GEMMs driven by oncogenes, mainly RAS family mutations (22, 26). However, the CP *Trp53^fl/fl^*; *p16^fl/^^fl^* GEMM lacks a classic oncogenic driver, which suggests that NRF2 can act as an oncogene in specific genetic contexts.

Among the hallmarks of NRF2-active tumors is a diminished tumor-suppressive immune response (52). Consistent with this, using a targeted OIS-PRM approach to quantify immune infiltration, we detected a marked reduction in tumor associated macrophage markers CD163A and CD68 in CPN samples relative to those from CP mice. Intriguingly, there is also diminished expression of IFN-responsive proteins including B2M, PD-L1, PD-L2, STING, and IRF3 in CPN samples. The downregulation of B2M, a component of the MHC class I molecule necessary for tumor antigen presentation, provides further evidence to support that constitutive NRF2 activity in this GEMM may have both oncogenic and immunoevasive properties, a phenomenon that has been recently reported in human cancers (53).

We did not expect the appearance of sarcomas in CP and CPN mice because the *K14-Cre^ERTAM2^* allele restricts expression of Cre recombinase to epithelial cells/tissues (K14-positive tissues; Supplementary Table S3). While the *Cre^ERTAM2^* transgene could be “leaky”—mice may express Cre recombinase in K14-negative tissues—other studies have established a strong specificity for this tissue-restricted allele (54). However, we did not observe these tumors upon aging of untreated mice for 14 months, arguing against leaky Cre expression. Furthermore, the epithelial cells in the OSCCs in the CPN mice were positive for NRF2 protein by IHC, while the sarcomas from CPN mice unexpectedly were not NRF2 positive (Fig. 3B; Supplementary Fig. S3). In contrast, SCCs from the ears of 2 CPN mice stained strongly for NRF2; the two ear SCCs from CP mice were negative by NRF2 IHC (Supplementary Fig. S2). Previous studies have shown that mouse epithelial tumors often undergo an epithelial-to-mesenchymal (EMT) transition as they progress, consistent with a more aggressive pathology (55–57). Consistent with this notion, we observed some sarcomas that showed small areas of PANCK expression among the vimentin-positive cells (Supplementary Fig. S3). Because previous reports showed that NRF2 can inhibit the EMT process after damage to lungs, the absence of NRF2 protein expression in the CPN sarcomas may indicate a requirement for its silencing to allow tumor progression, likely though an epigenetic mechanism (58, 59). We have previously observed NRF2 silencing in large neuroendocrine lung cancers in the CPN mice (21).

Caveats for this study come from the development of sex-specific phenotypes that impacted survival. First, inflammatory eye disease in all female CP mice (10/10) requiring their sacrifice by 30 weeks. As such, it is possible that some of the CP mice may have been sacrificed before tumor development. Interestingly, only 10% of the male CP mice (1/10) developed eye problems, supporting a sex-dependent phenotype. Furthermore, only 3/13 CPN female mice developed an eye infection, implicating a protective role for NRF2 in the eye. Further analysis of the cause and type of this eye inflammation is needed. Second, we observed more mice with weight loss in male CPN mice than in male CP mice (Supplementary Fig. S1B). Though possibly driven by malnutrition resulting from the hyperkeratosis of the oral, esophagus and forestomach observed in only CPN mice, we did not observe problems with food intake in the N mice (23). However, our future studies will identify the cause of weight loss in the CPN mice and its potential impact on tumor development.

In summary, this study presents the first GEMM demonstrating that activated NRF2 signaling can drive the development of OSCC. The availability of this OSCC GEMM will provide an important reagent for studies identifying the cellular and environmental factors that drive its progression to metastatic disease. As novel NRF2 inhibitors become available, this GEMM will allow their assessment at different stages of tumor development. Therefore, our GEMM should provide a human OSCC-relevant model to better understand the molecular events associated with the initiation, progression, and treatment of this cancer.

## Supplementary Material

Figure S1 Figure S1 shows survival curves and weight loss of CP and CPN mice after treatment with tamoxifen.

Figure S2 Figure S2 shows examples of ear squamous cell carcinoma (SCC) from CP and CPN mice.

Figure S3 Figure S3 shows examples of abdominal tumors from CP and CPN mice.

Figure S4 Figure S4 shows scatterplots Protein abundances measured by targeted protein mass spectrometry in CP and CPN oral cavity tissues.

Figure S5 Figure S5 shows box-and-whisker plots of protein abundance as measured by targeted protein mass spectrometry CP and CPN oral cavity tissues.

Table S1 Table S1 shows mouse SIL catalogue.

Table S2 Table S2 shows TCGA data used to analyze mutations in TP53, CDKN2A, and NRF2 pathways.

Table S3 Table S3 shows IHC-NRF2 score in control tissues from CP & CPN mice treated with TMX

Table S4 Table S4 shows other invasive tumors developed by CP and CPN mice.

Table S5 Table S5 shows protein pivot results.
