# Supplementary material for: NRF2 Activation in Trp53;p16-deficient Mice Drives Oral Squamous Cell Carcinoma
Source: Cancer Res Commun. 2024 Feb 21;4(2):487–95. doi: 10.1158/2767-9764.CRC-23-0386 (PMC10880604; doi:10.1158/2767-9764.CRC-23-0386)
Supplement: Figure S1 — shows survival curves and weight loss of CP and CPN mice after treatment with tamoxifen. [file crc-23-0386-s01.docx]

**
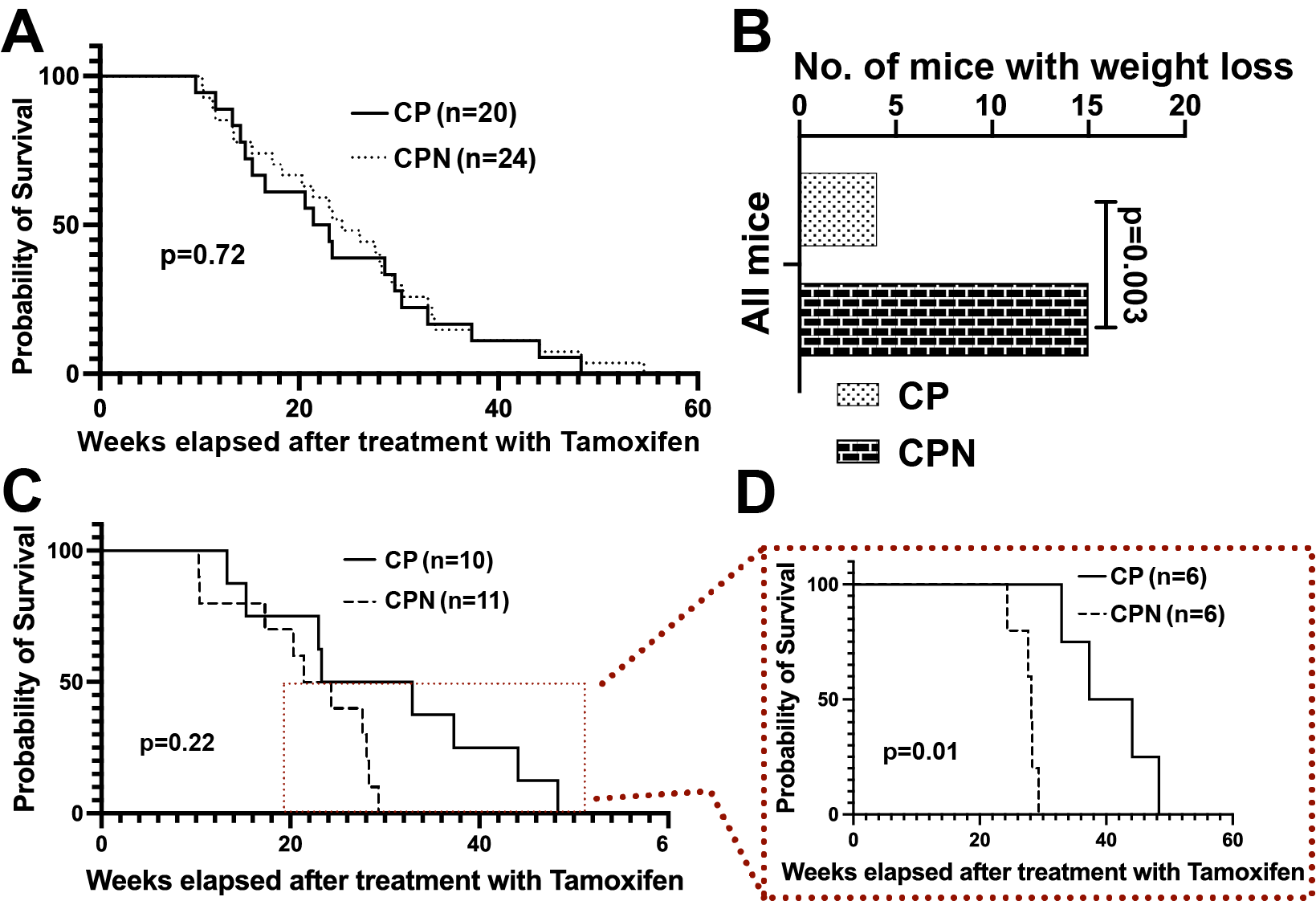
**

**Figure S1.** **Survival curves and weight loss of CP and CPN mice after treatment with tamoxifen. A.** Survival curve of CP mice versus CPN mice; **B.** Number of mice with weight loss in both genotypes; **C.** Survival curve of all male CP versus male CPN mice; **D.** Inset from Panel **C** demonstrating a significant difference in survival of male mice after ~30 weeks. P-values calculated by Gehan-Breslow-Wilcoxon test.
