# Supplementary material for: NRF2 Activation in Trp53;p16-deficient Mice Drives Oral Squamous Cell Carcinoma
Source: Cancer Res Commun. 2024 Feb 21;4(2):487–95. doi: 10.1158/2767-9764.CRC-23-0386 (PMC10880604; doi:10.1158/2767-9764.CRC-23-0386)
Supplement: Figure S2 — shows examples of ear squamous cell carcinoma (SCC) from CP and CPN mice. [file crc-23-0386-s02.docx]

**
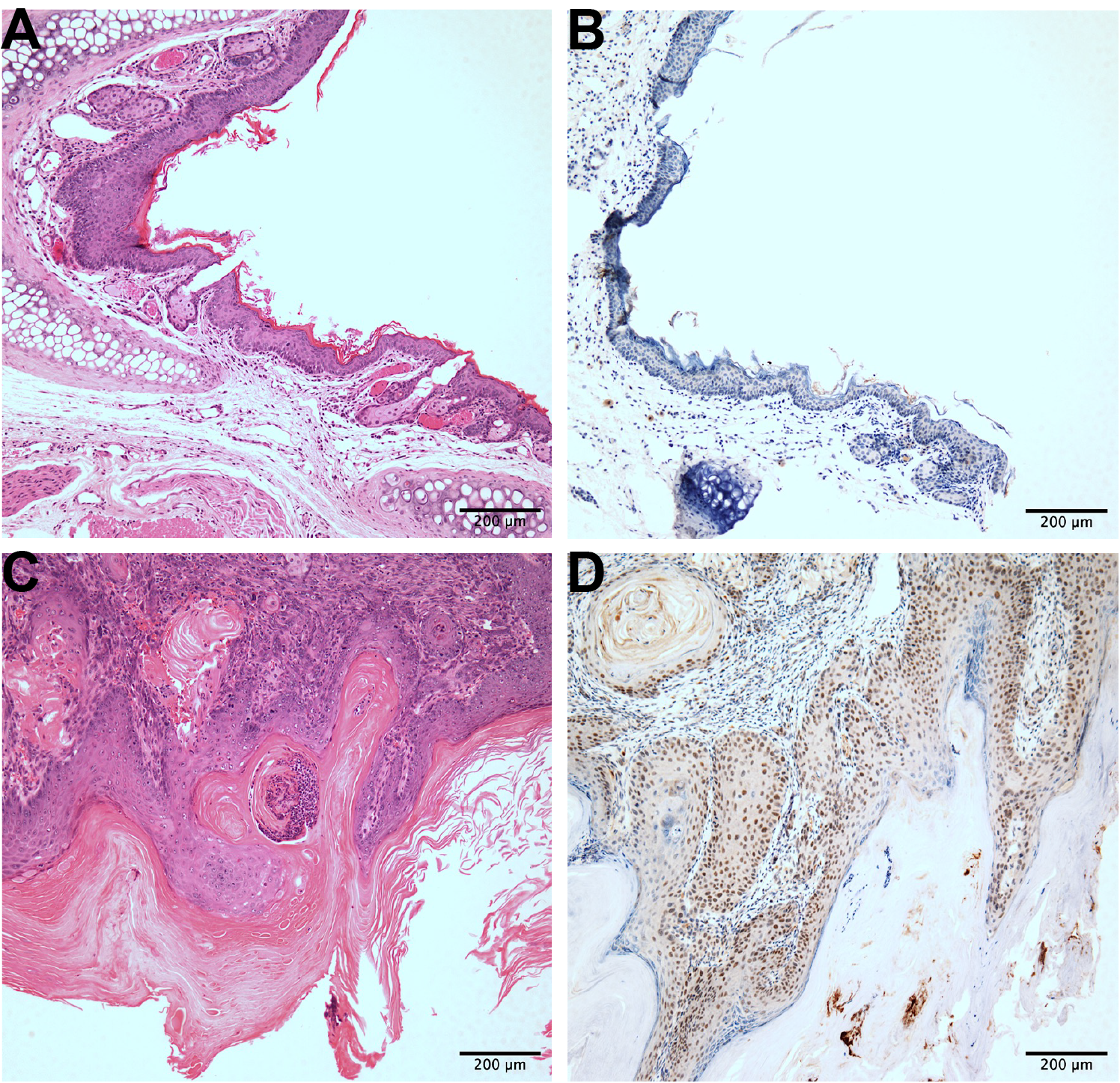
**

**Figure S2.** **Examples of** **ear squamous cell carcinoma (SCC) from CP and CPN mice. A.** H&E and **B.** NRF2-IHC of ear SCC from a CP mouse; **C.** H&E and **D.** NRF2-IHC of ear SCC from a CPN mouse, scalebar=200μm. Images were taken using a BX61-Neville microscope.
