# Supplementary material for: NRF2 Activation in Trp53;p16-deficient Mice Drives Oral Squamous Cell Carcinoma
Source: Cancer Res Commun. 2024 Feb 21;4(2):487–95. doi: 10.1158/2767-9764.CRC-23-0386 (PMC10880604; doi:10.1158/2767-9764.CRC-23-0386)
Supplement: Figure S3 — shows examples of abdominal tumors from CP and CPN mice. [file crc-23-0386-s03.docx]

**
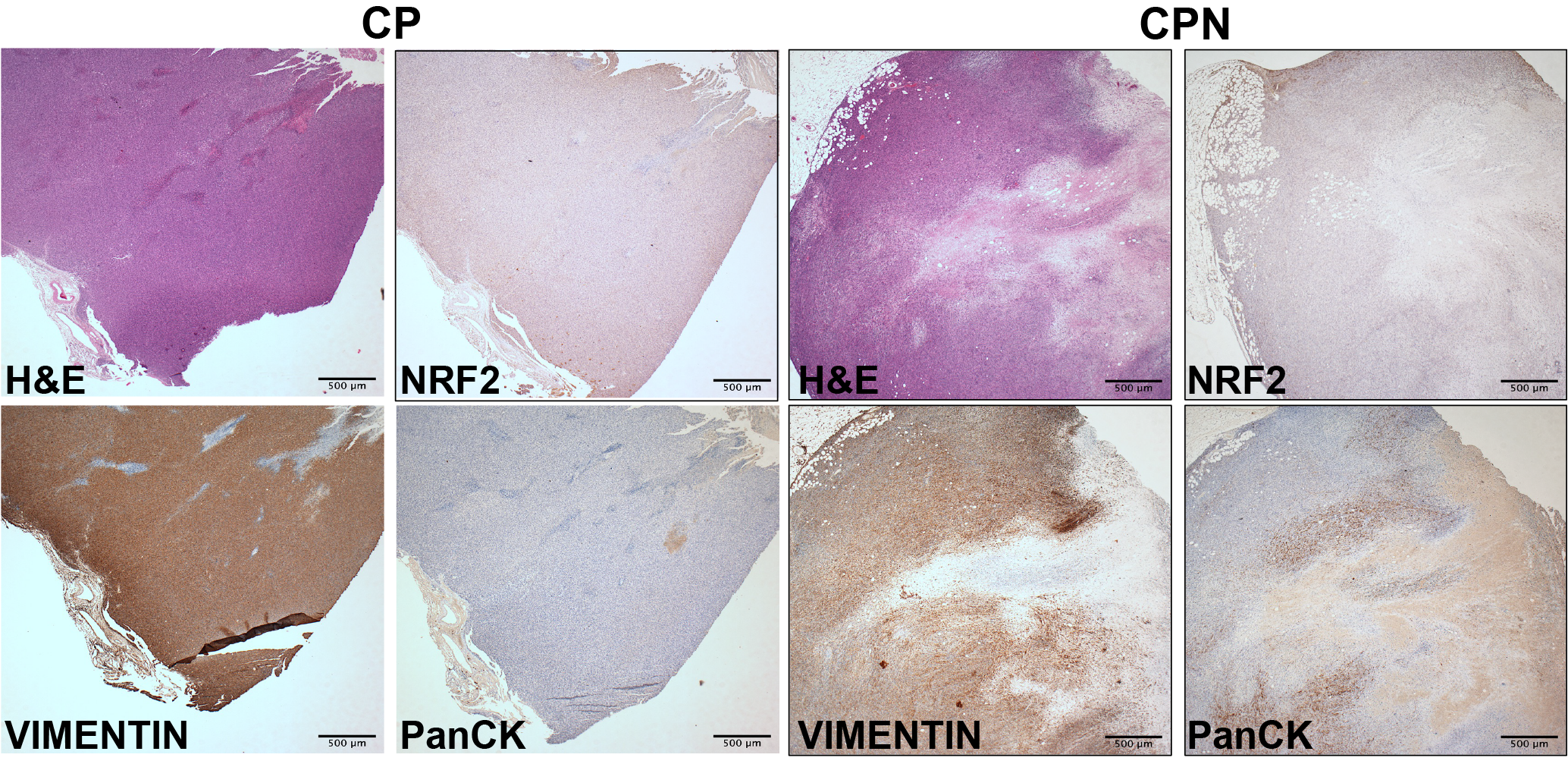
**

**Figure S3.** **Examples of abdominal tumors from CP and CPN mice.** Scalebar=500μm. Images were taken using a BX61-Neville microscope.
