# Supplementary material for: NRF2 Activation in Trp53;p16-deficient Mice Drives Oral Squamous Cell Carcinoma
Source: Cancer Res Commun. 2024 Feb 21;4(2):487–95. doi: 10.1158/2767-9764.CRC-23-0386 (PMC10880604; doi:10.1158/2767-9764.CRC-23-0386)
Supplement: Figure S4 — shows scatterplots Protein abundances measured by targeted protein mass spectrometry in CP and CPN oral cavity tissues. [file crc-23-0386-s04.pdf]

Figure S4. Scatter plots of protein abundance in CP and CPN oral cavity tissues. Scatter plots of protein abundance against the NRF2 score were calculated using the first principal component as described in the methods. Protein abundances were measured by targeted protein mass spectrometry in CP and CPN oral cavity tissues. Spearman rank order correlation is reported with p-values adjusted by a Benjamin Hochberg procedure (34).

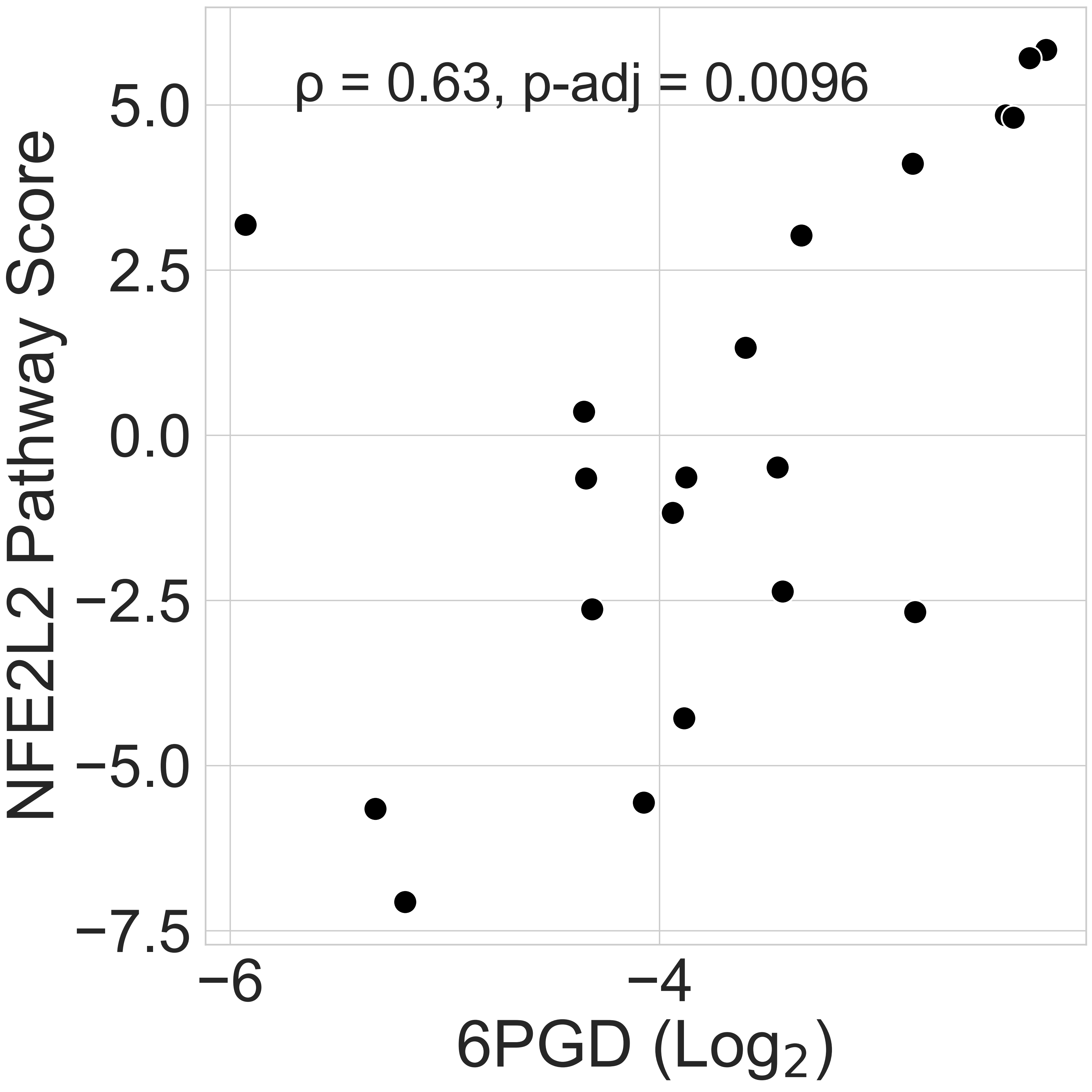
ρ = 0.63, p-adj = 0.0096
NFE2L2 Pathway Score
5.0
2.5
0.0
−2.5
−5.0
−7.5
−6
−4
6PGD (Log2)

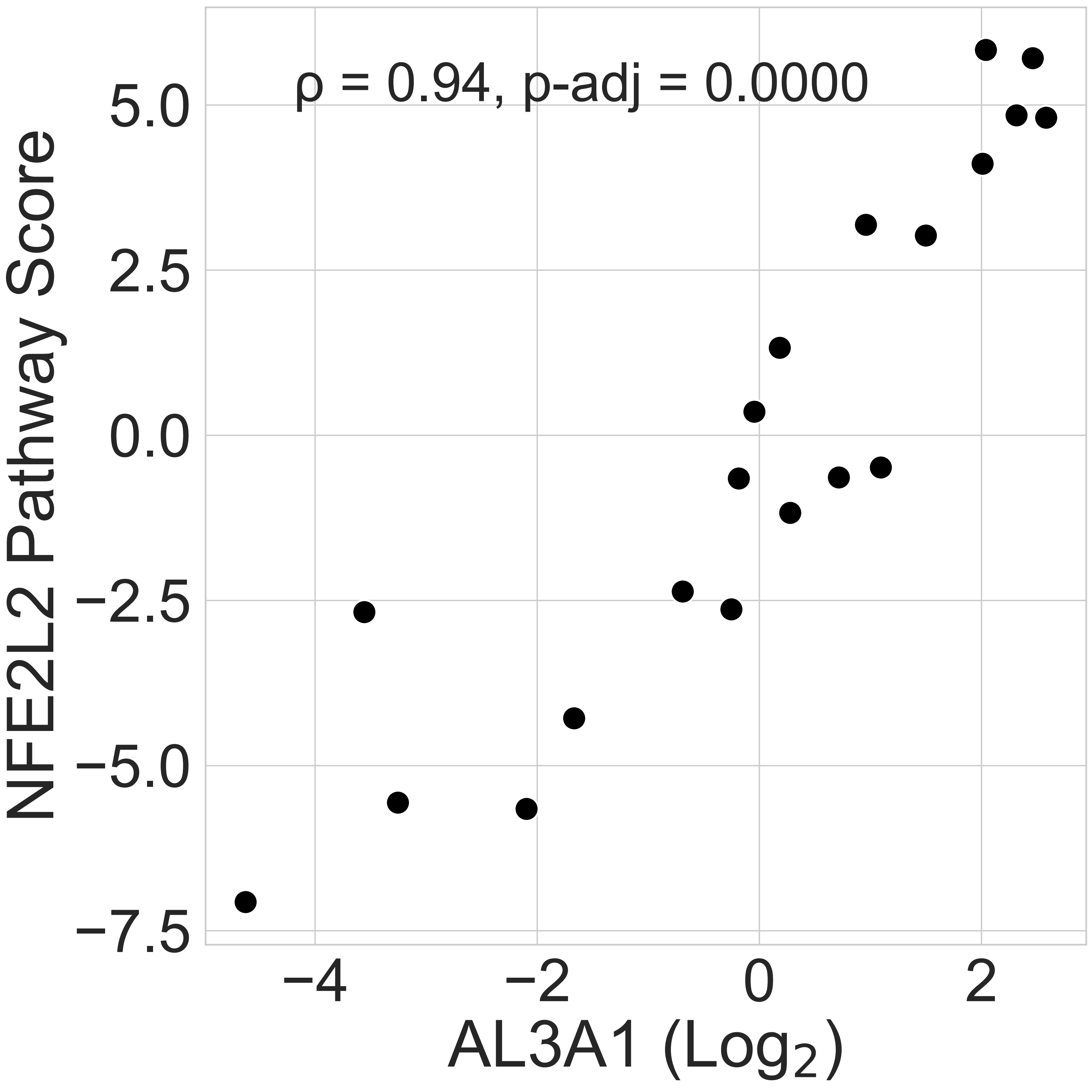

ρ = 0.94, p-adj = 0.0000
NFE2L2 Pathway Score
AL3A1 (Log2)
5.0
2.5
0.0
−2.5
−5.0
−7.5
−4
−2
0
2

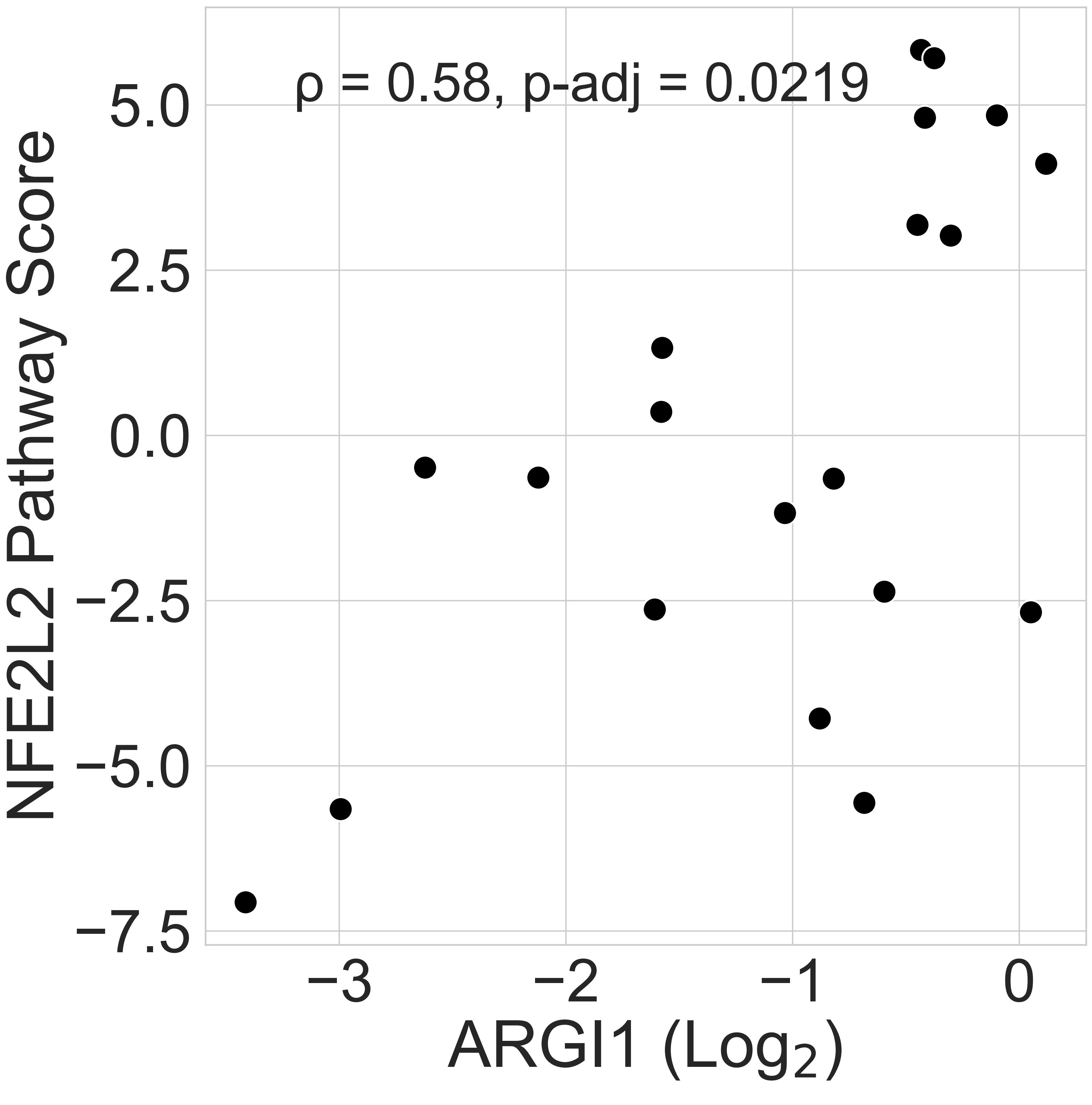
ρ = 0.58, p-adj = 0.0219
NFE2L2 Pathway Score
5.0
2.5
0.0
−2.5
−5.0
−7.5
−3
−2
−1
0
ARGI1 (Log2)

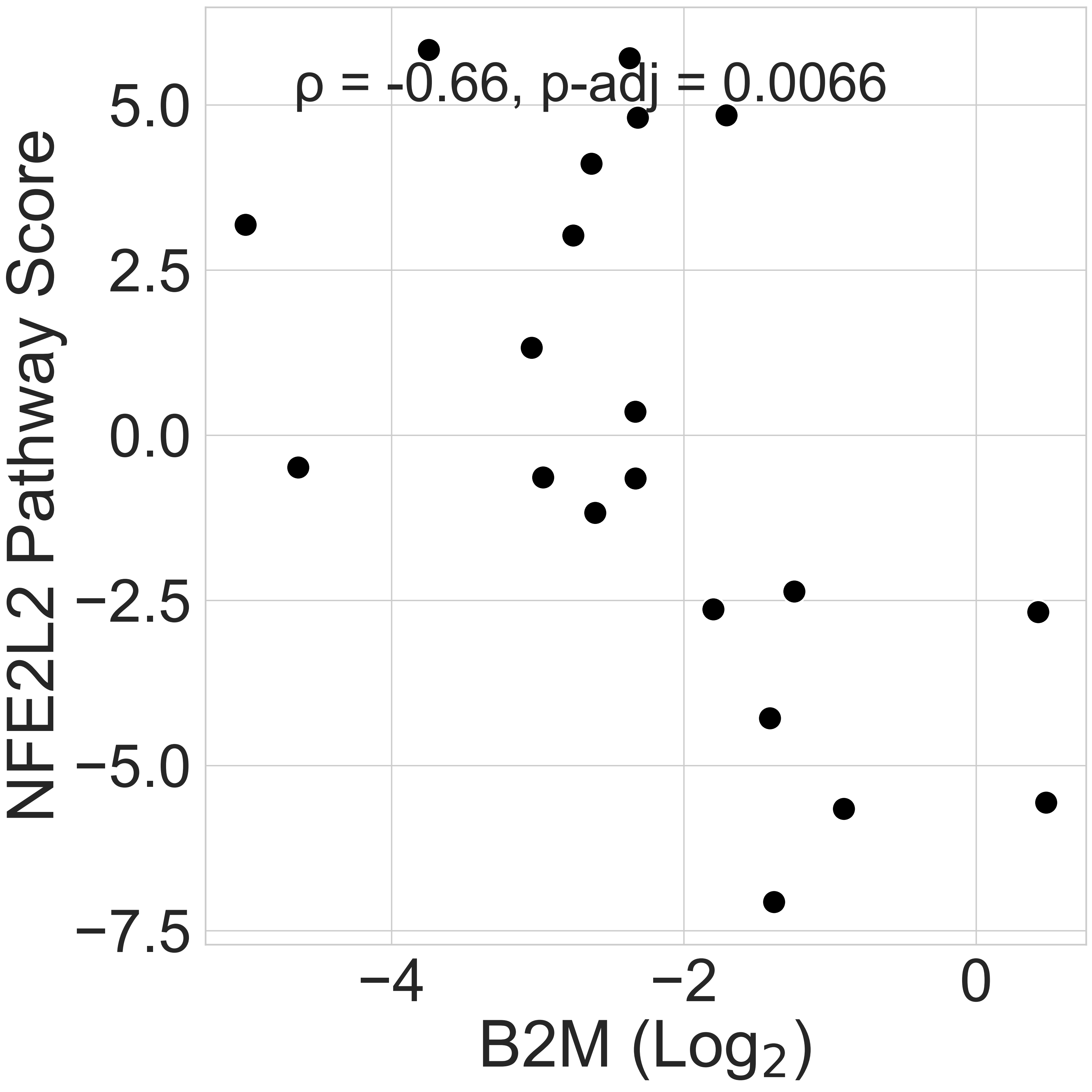
ρ = -0.66, p-adj = 0.0066
NFE2L2 Pathway Score
5.0
2.5
0.0
−2.5
−5.0
−7.5
−4
−2
0
B2M (Log₂)

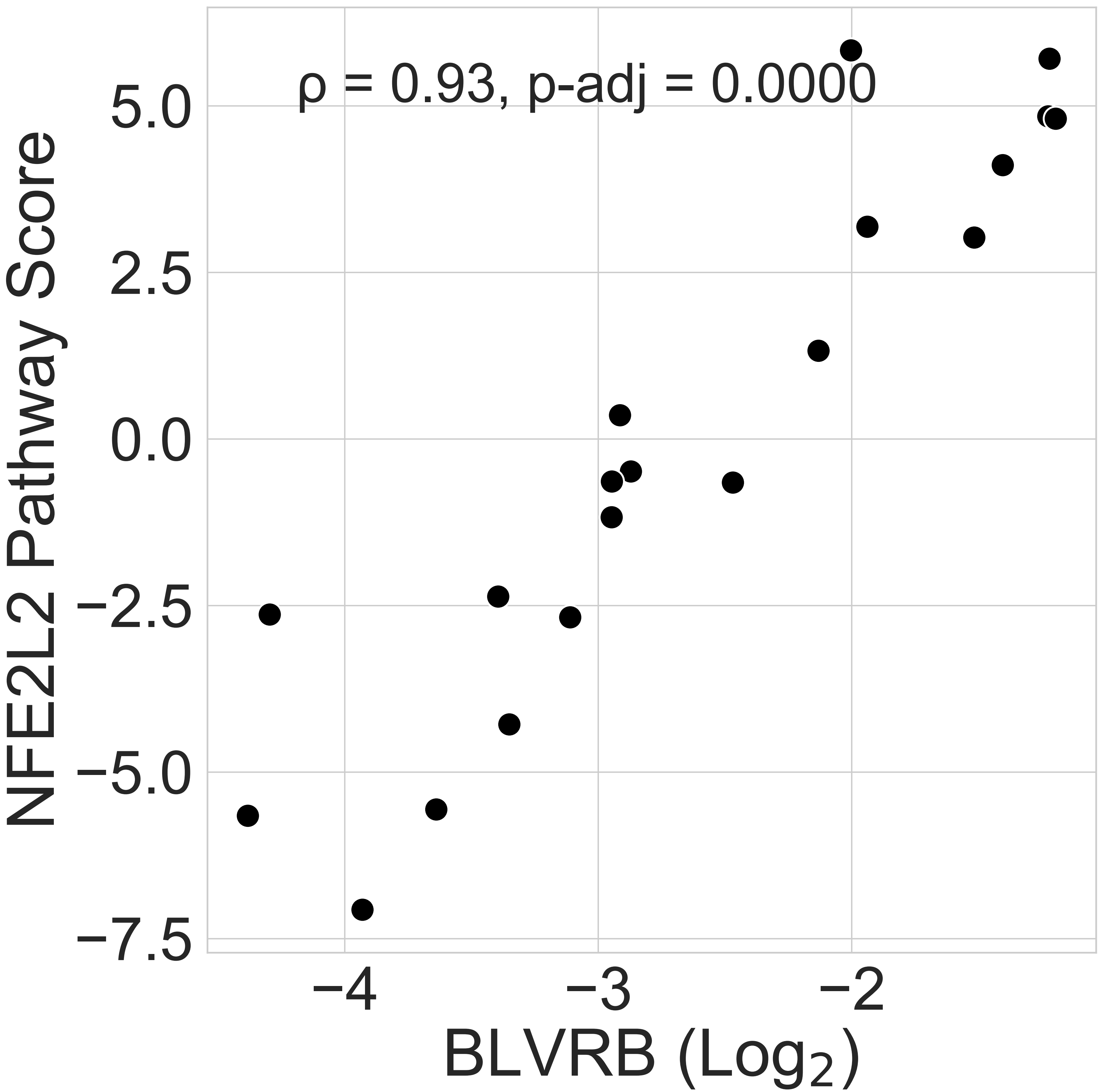
ρ = 0.93, p-adj = 0.0000
5.0
2.5
0.0
−2.5
−5.0
−7.5
NFE2L2 Pathway Score
−4
−3
−2
BLVRB (Log2)

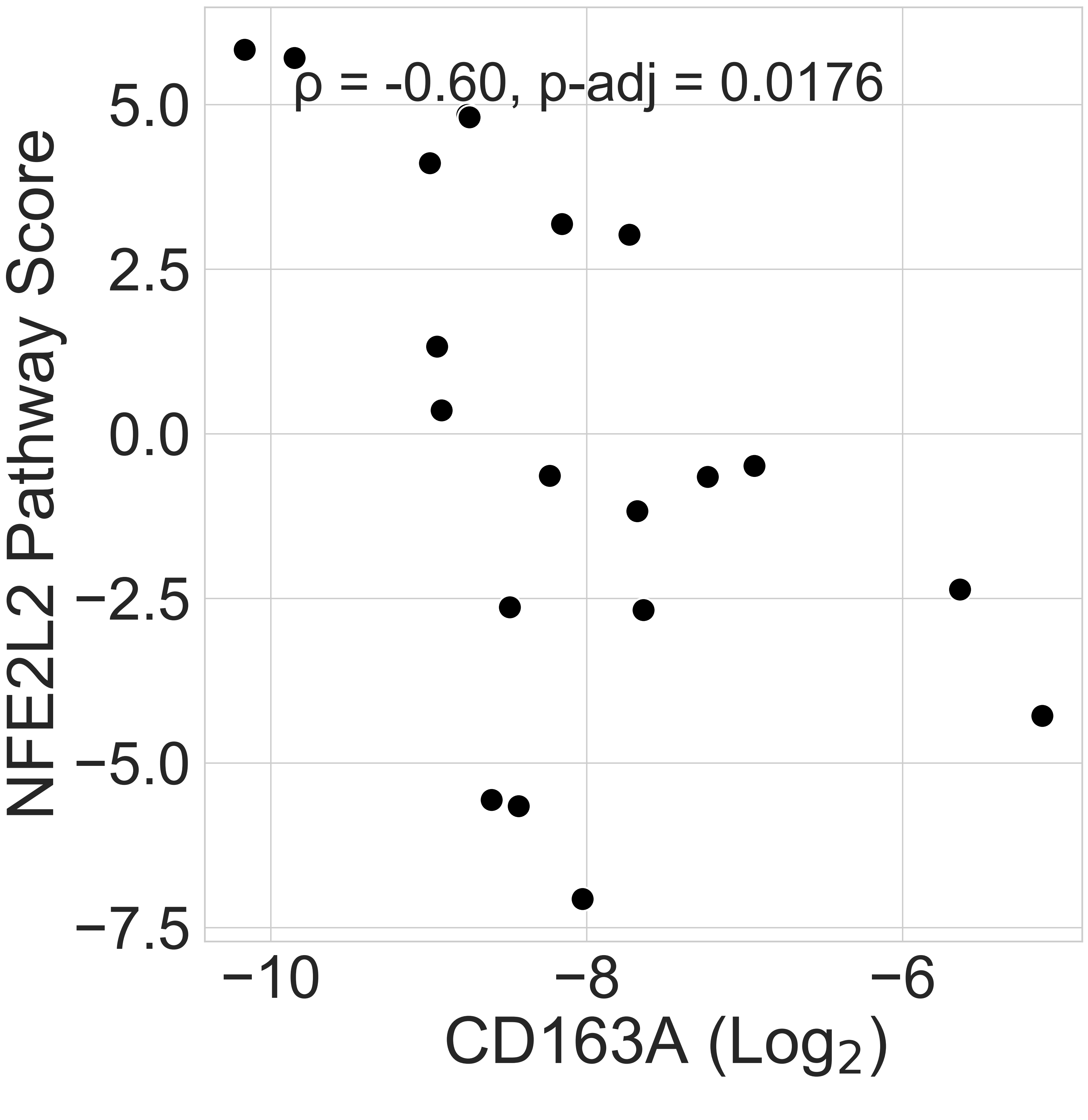

ρ = -0.60, p-adj = 0.0176
5.0
2.5
0.0
−2.5
−5.0
−7.5
NFE2L2 Pathway Score
−10
−8
−6
CD163A (Log₂)

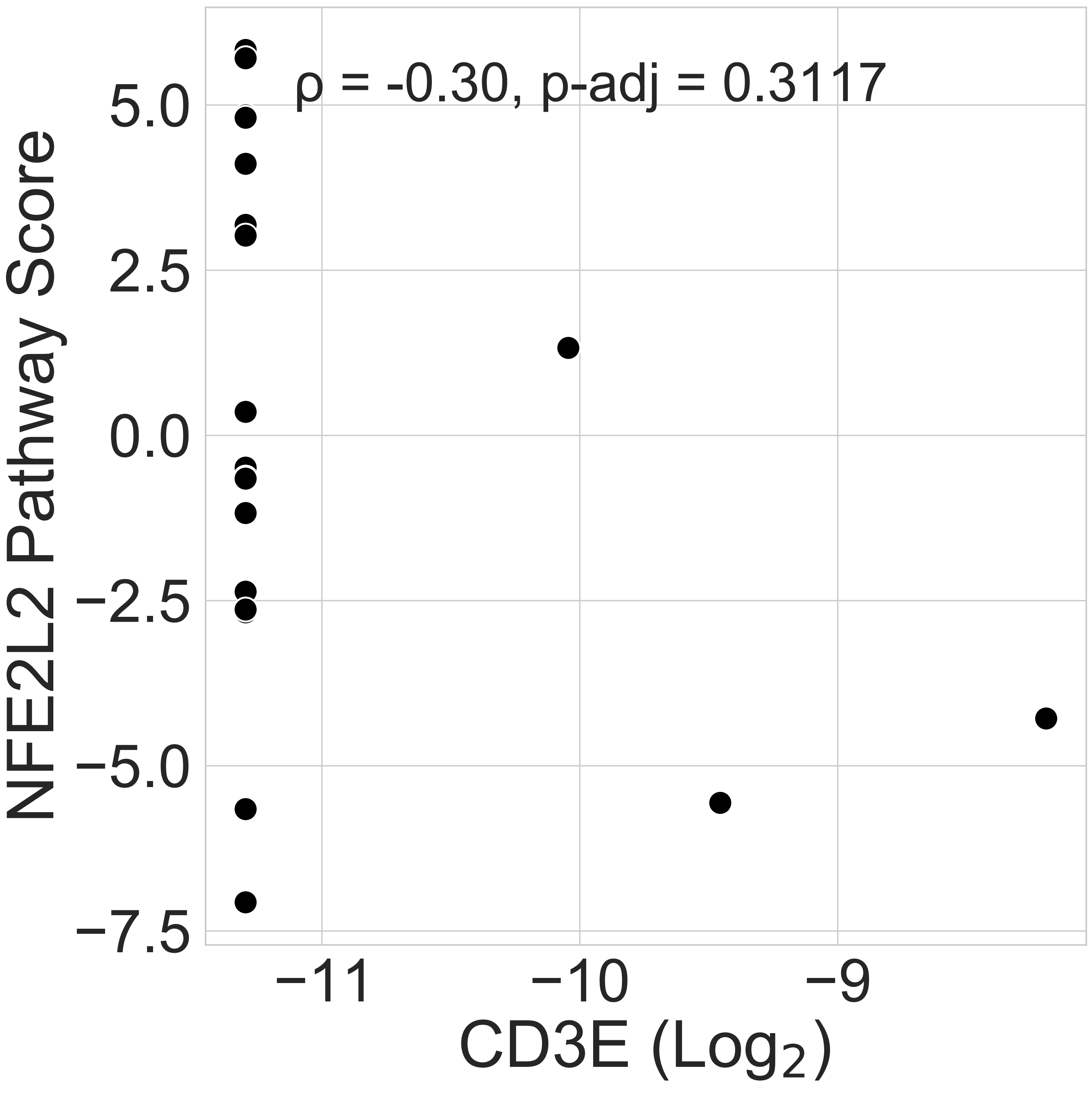

ρ = -0.30, p-adj = 0.3117
NFE2L2 Pathway Score
5.0
2.5
0.0
−2.5
−5.0
−7.5
−11
−10
−9
CD3E (Log2)

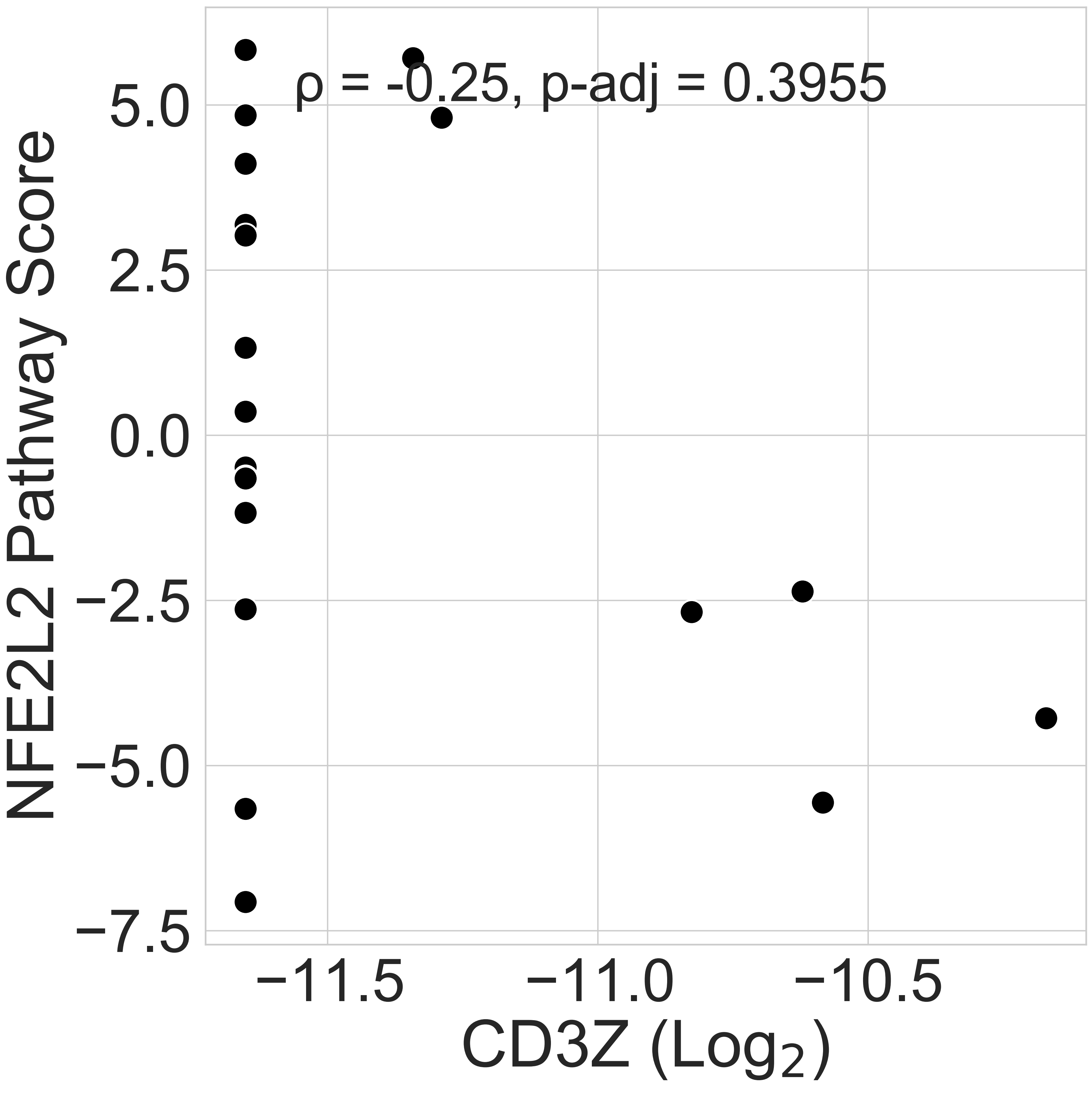
ρ = -0.25, p-adj = 0.3955
NFE2L2 Pathway Score
5.0
2.5
0.0
−2.5
−5.0
−7.5
−11.5
−11.0
−10.5
CD3Z (Log2)

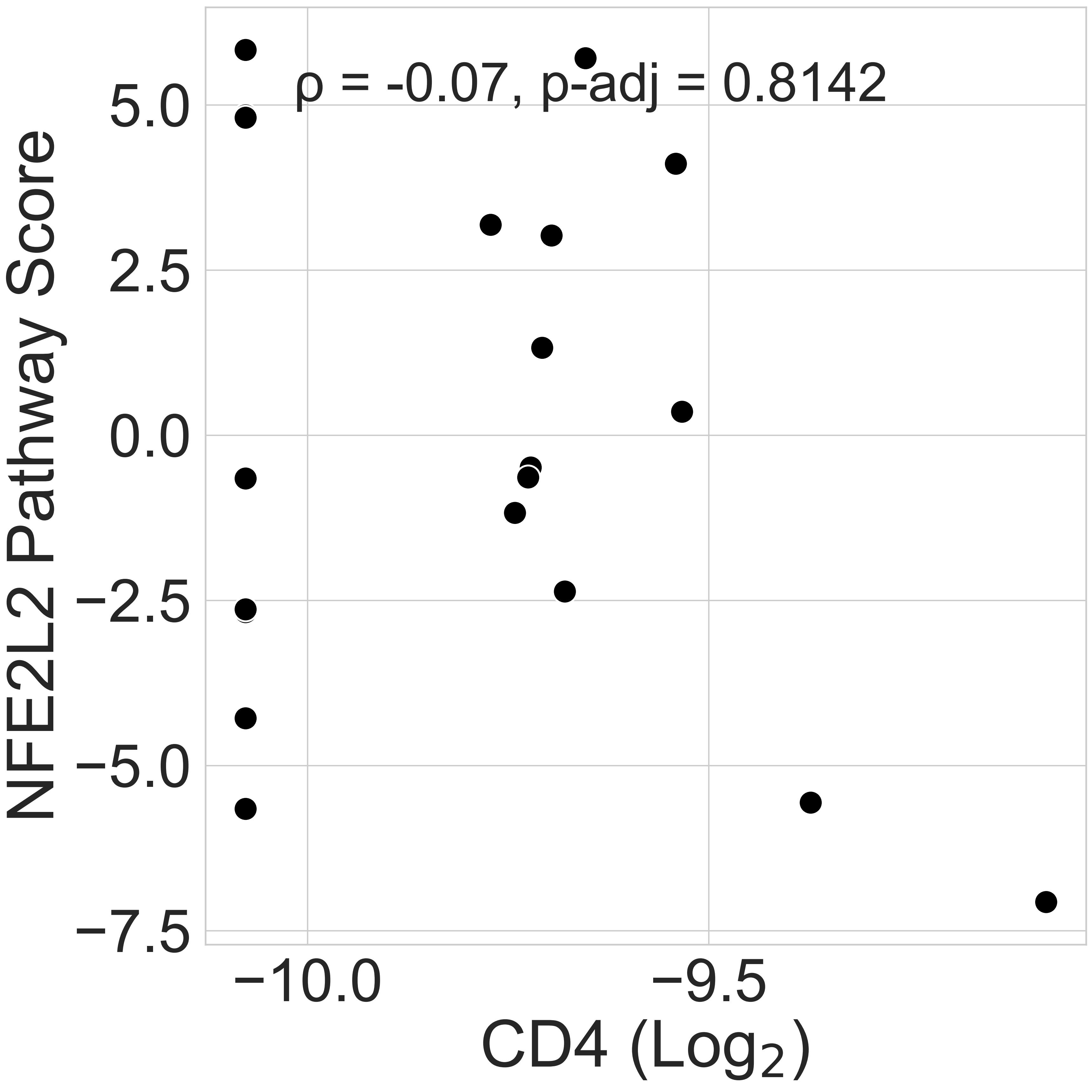

ρ = -0.07, p-adj = 0.8142
NFE2L2 Pathway Score
5.0
2.5
0.0
−2.5
−5.0
−7.5
−10.0
−9.5
CD4 (Log₂)

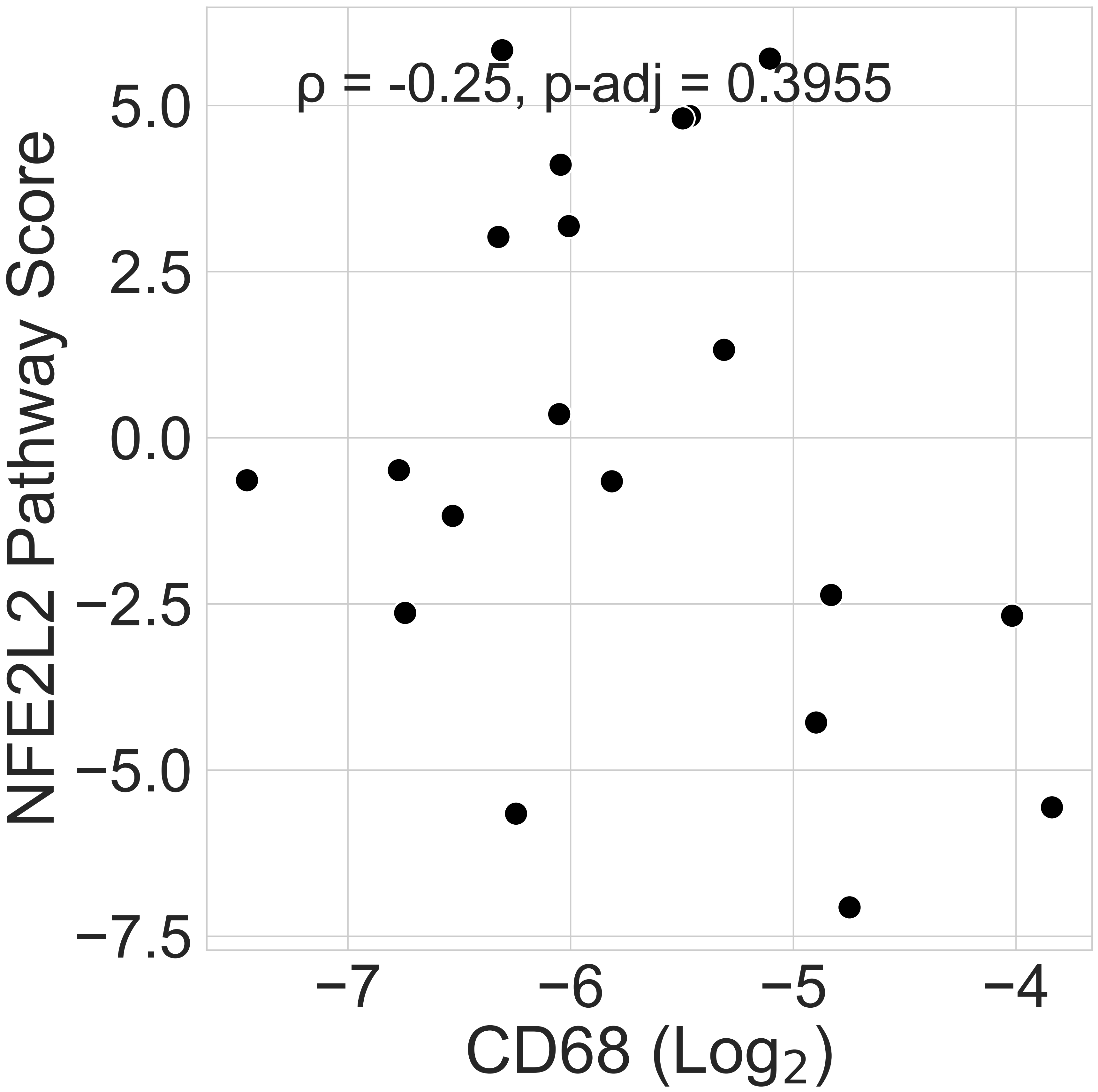
ρ = -0.25, p-adj = 0.3955
NFE2L2 Pathway Score
5.0
2.5
0.0
−2.5
−5.0
−7.5
−7
−6
−5
−4
CD68 (Log2)

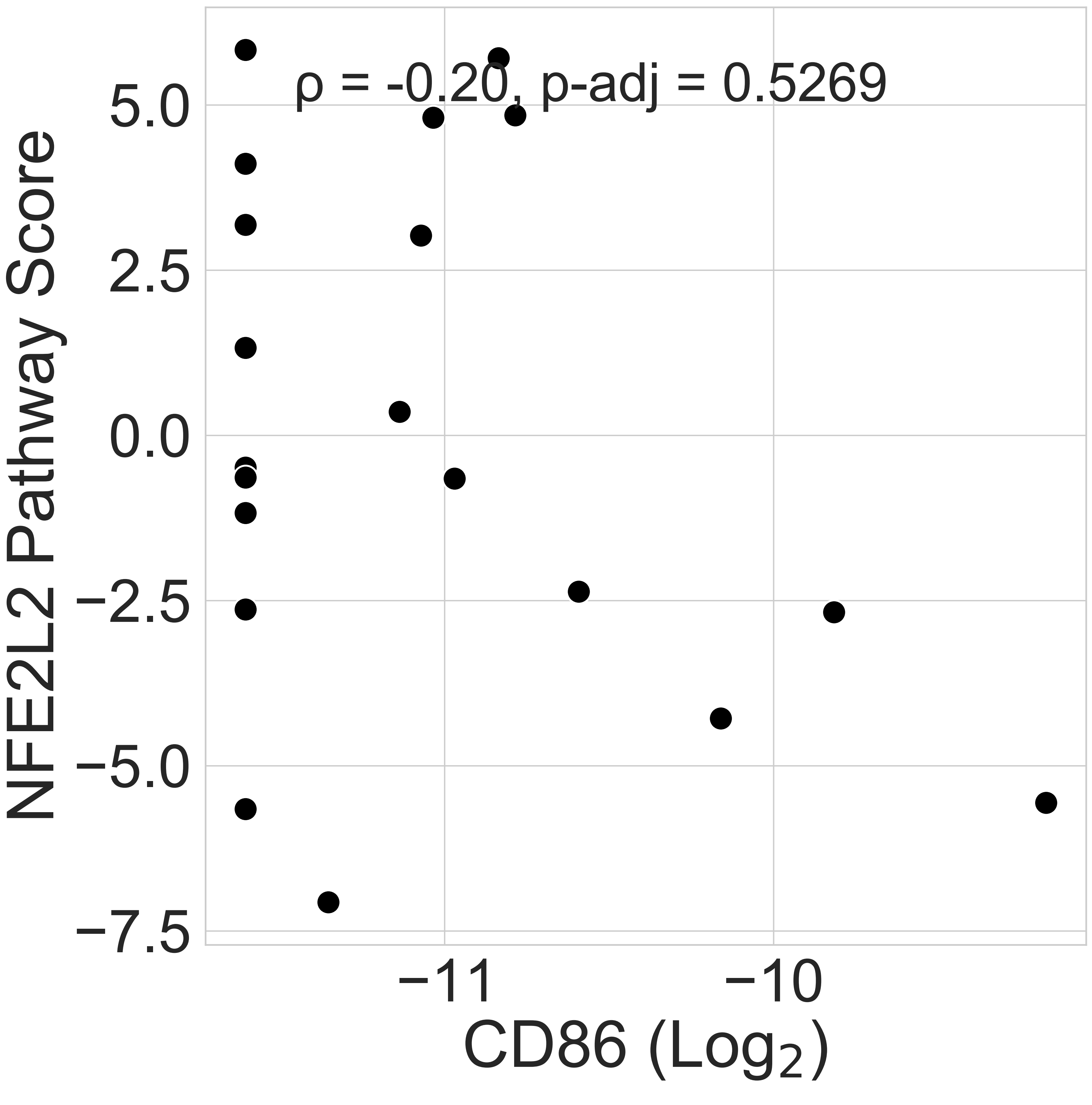

ρ = -0.20, p-adj = 0.5269
NFE2L2 Pathway Score
5.0
2.5
0.0
−2.5
−5.0
−7.5
−11
−10
CD86 (Log₂)

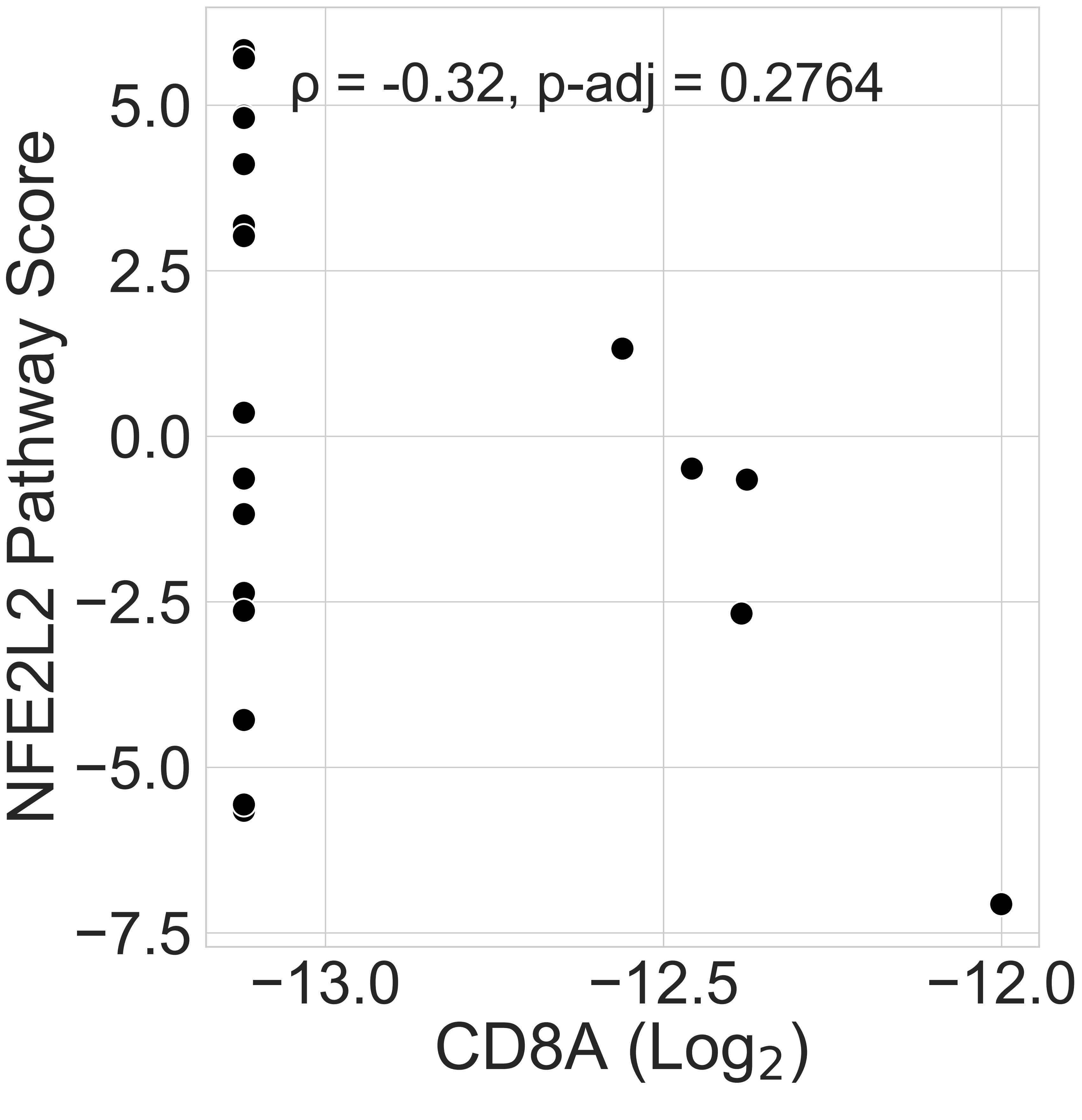

ρ = -0.32, p-adj = 0.2764
NFE2L2 Pathway Score
5.0
2.5
0.0
−2.5
−5.0
−7.5
−13.0
−12.5
−12.0
CD8A (Log2)

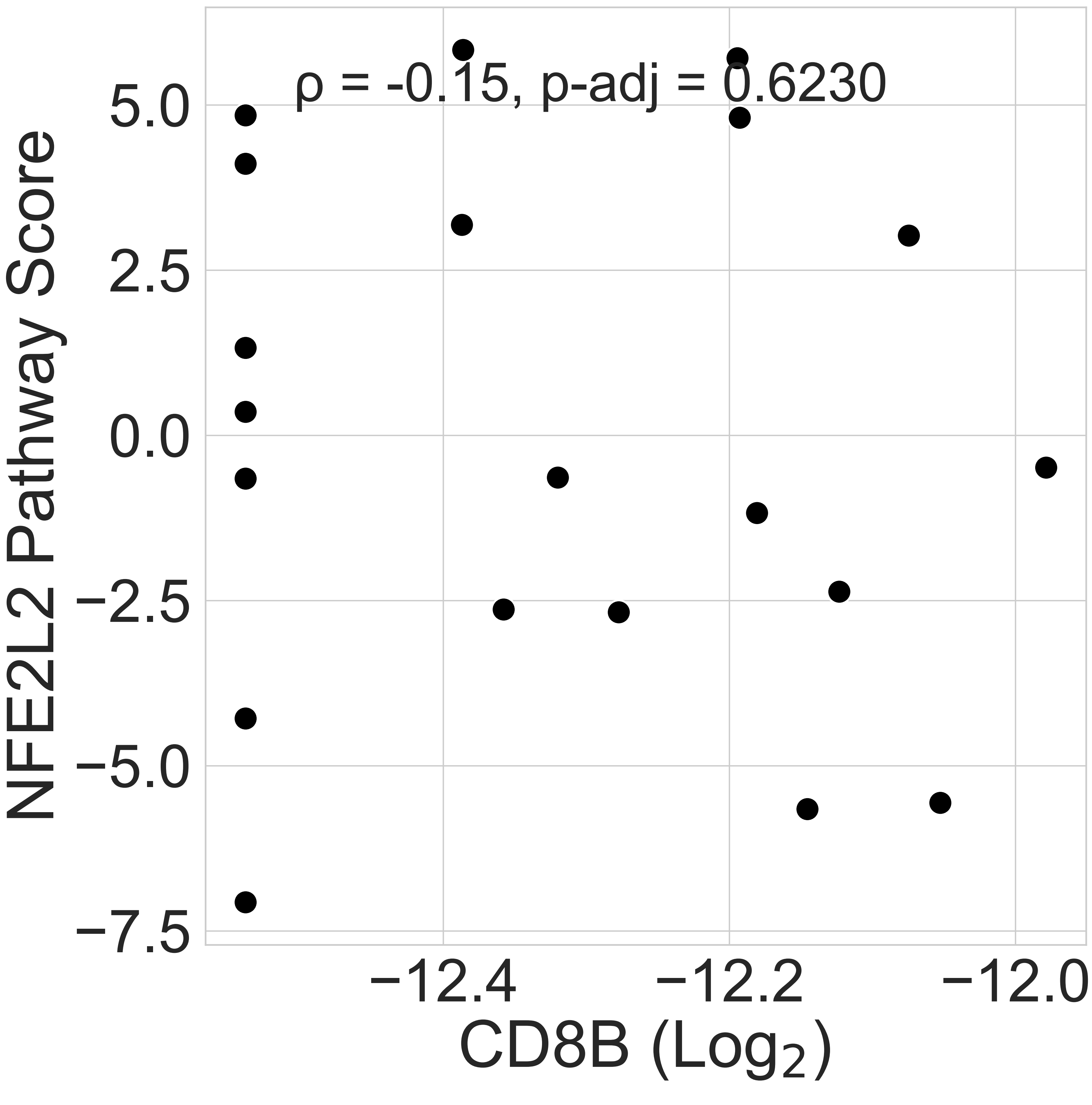

ρ = -0.15, p-adj = 0.6230
5.0
2.5
0.0
−2.5
−5.0
−7.5
−12.4
−12.2
−12.0
NFE2L2 Pathway Score
CD8B (Log2)

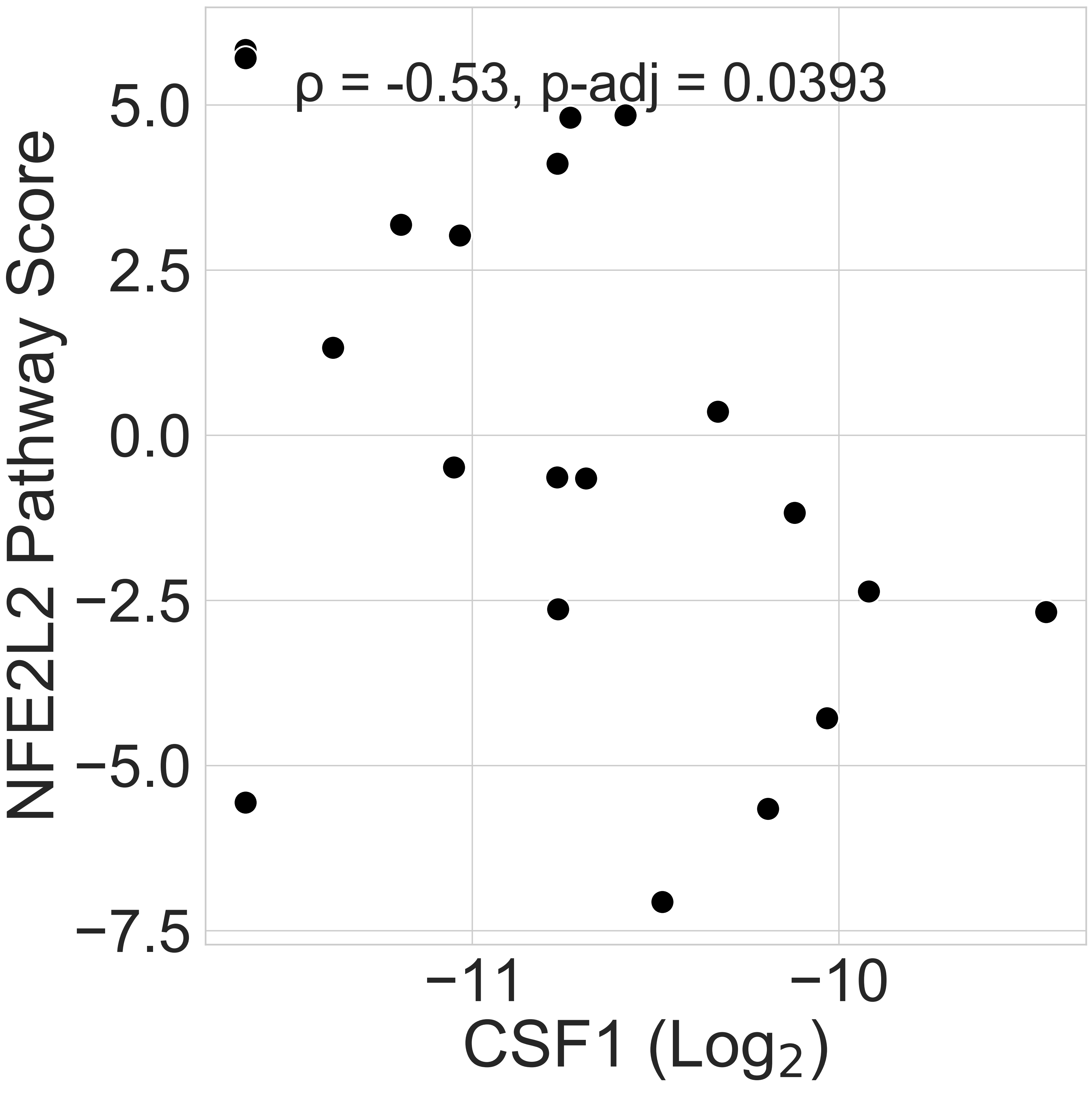
ρ = -0.53, p-adj = 0.0393
NFE2L2 Pathway Score
5.0
2.5
0.0
−2.5
−5.0
−7.5
−11
−10
CSF1 (Log₂)

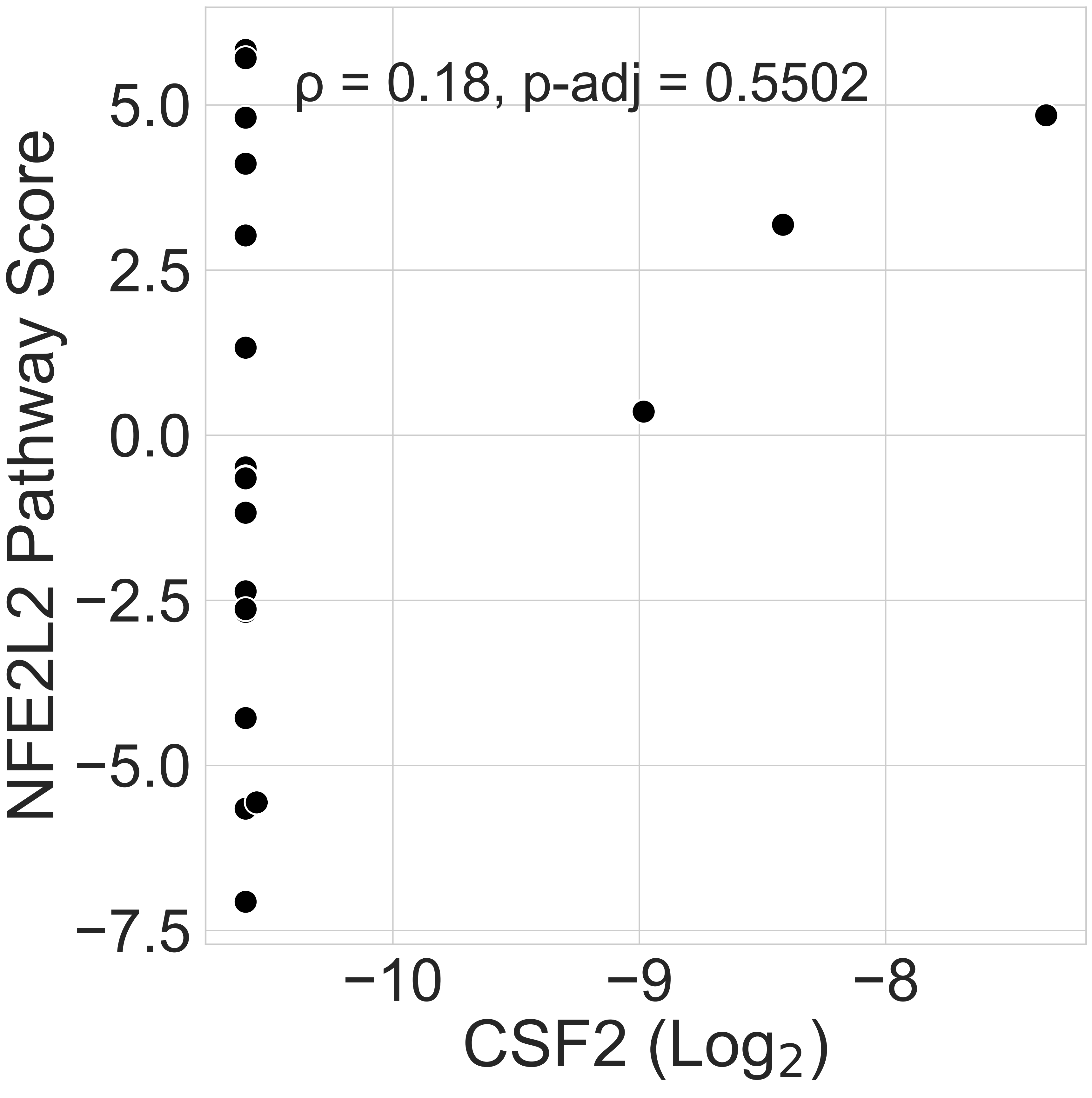
ρ = 0.18, p-adj = 0.5502
NFE2L2 Pathway Score
5.0
2.5
0.0
−2.5
−5.0
−7.5
−10
−9
−8
CSF2 (Log₂)

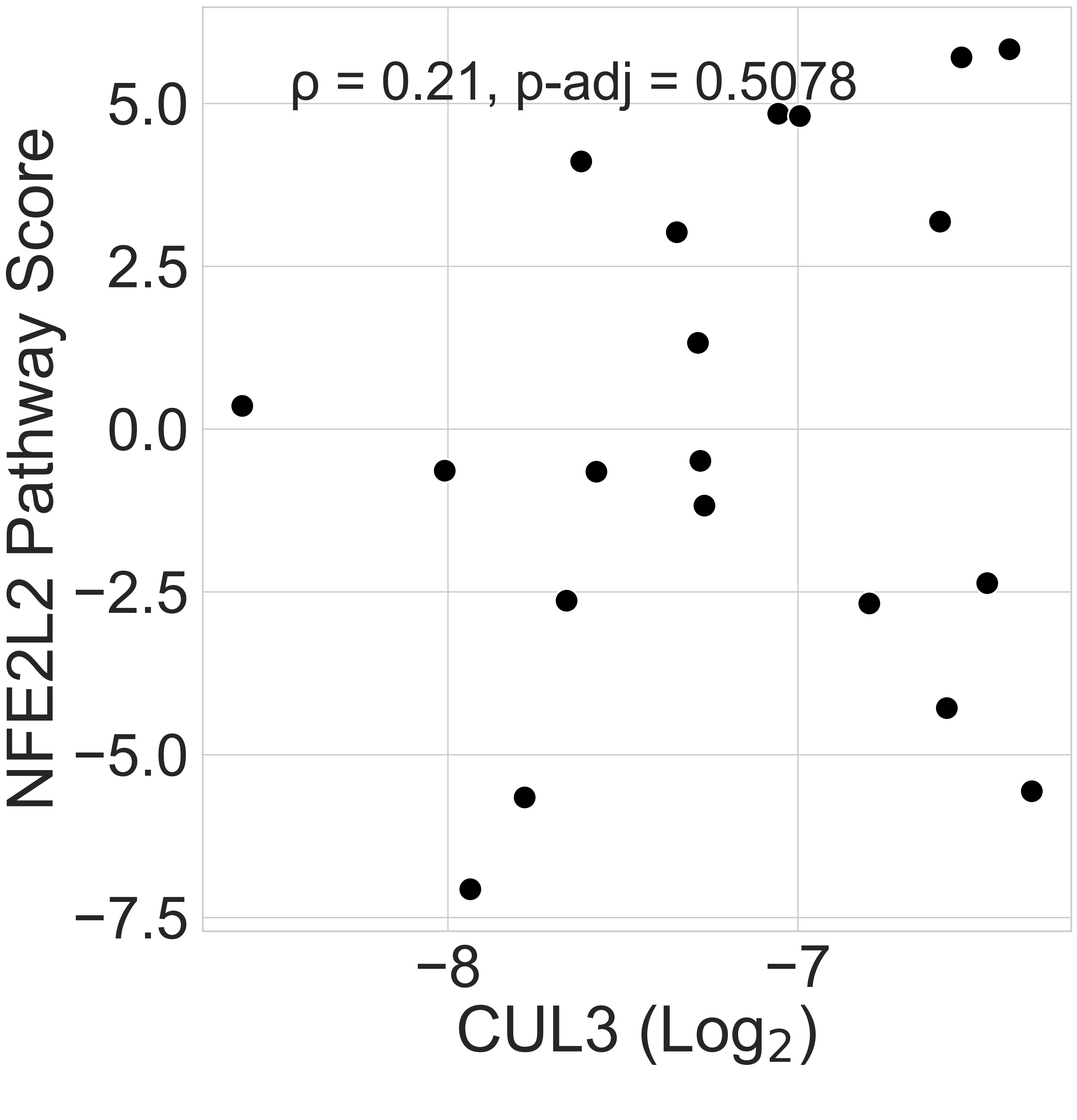

ρ = 0.21, p-adj = 0.5078
NFE2L2 Pathway Score
5.0
2.5
0.0
−2.5
−5.0
−7.5
−8
−7
CUL3 (Log₂)

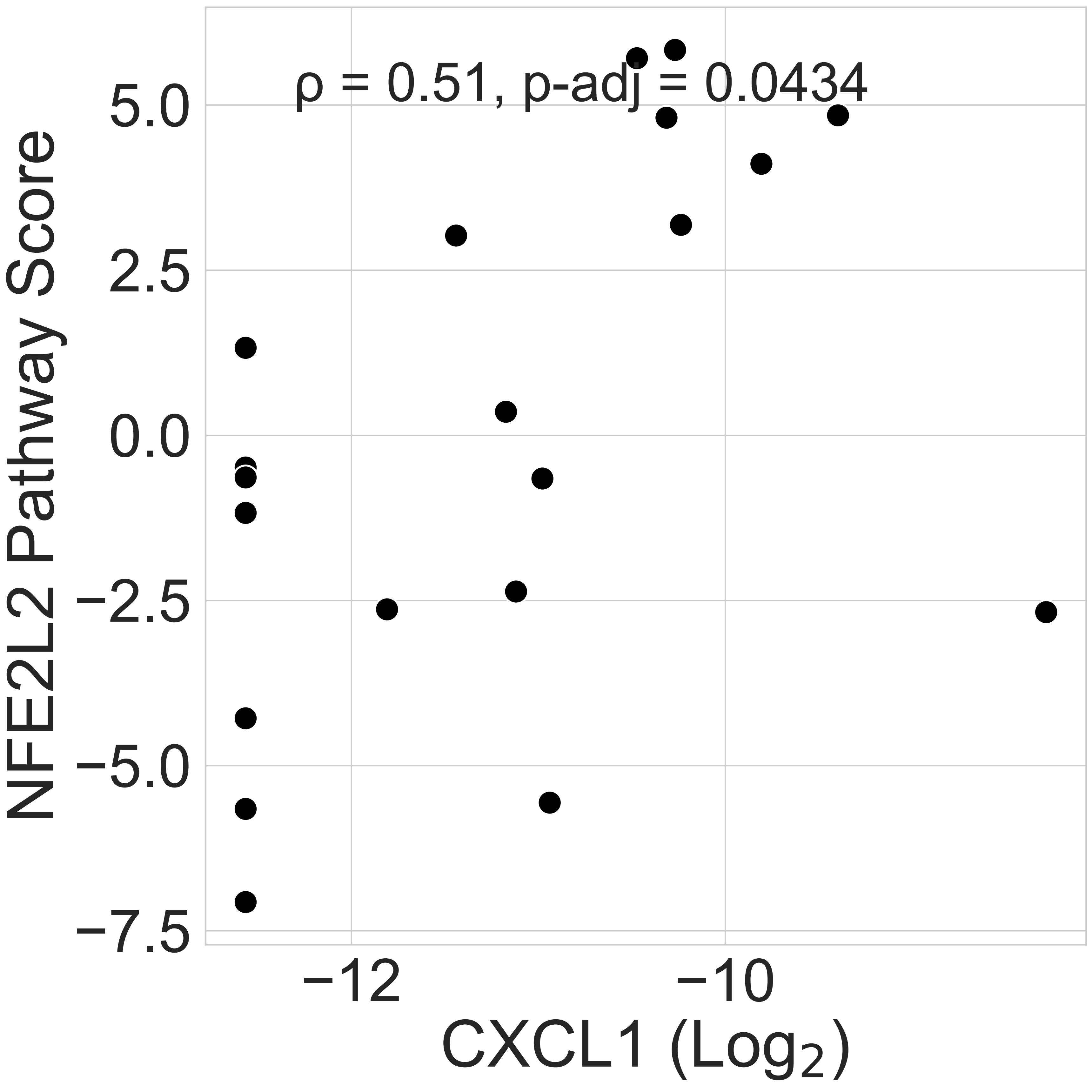

ρ = 0.51, p-adj = 0.0434
NFE2L2 Pathway Score
5.0
2.5
0.0
−2.5
−5.0
−7.5
−12
−10
CXCL1 (Log2)

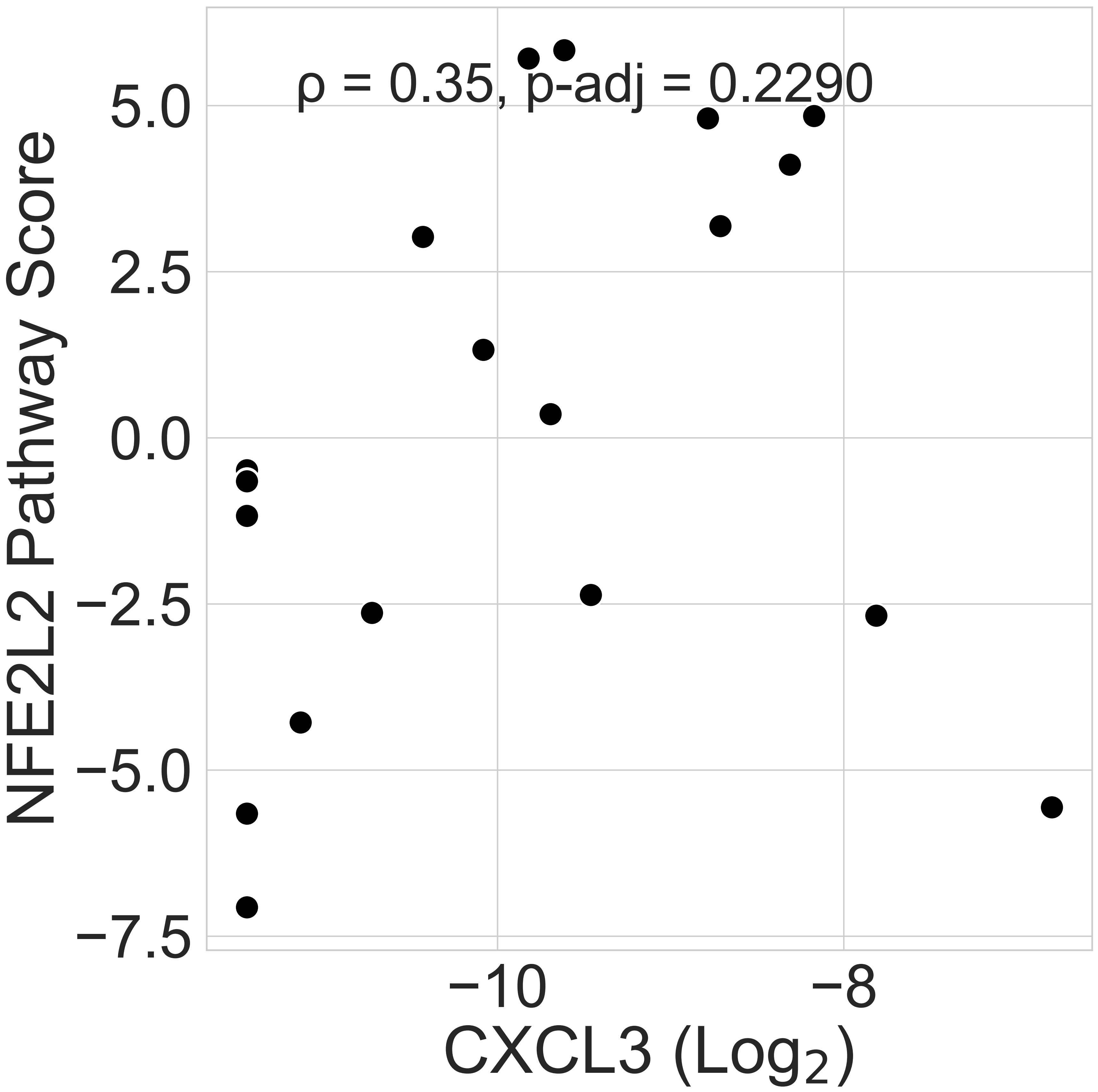

ρ = 0.35, p-adj = 0.2290
NFE2L2 Pathway Score
5.0
2.5
0.0
−2.5
−5.0
−7.5
−10
−8
CXCL3 (Log2)

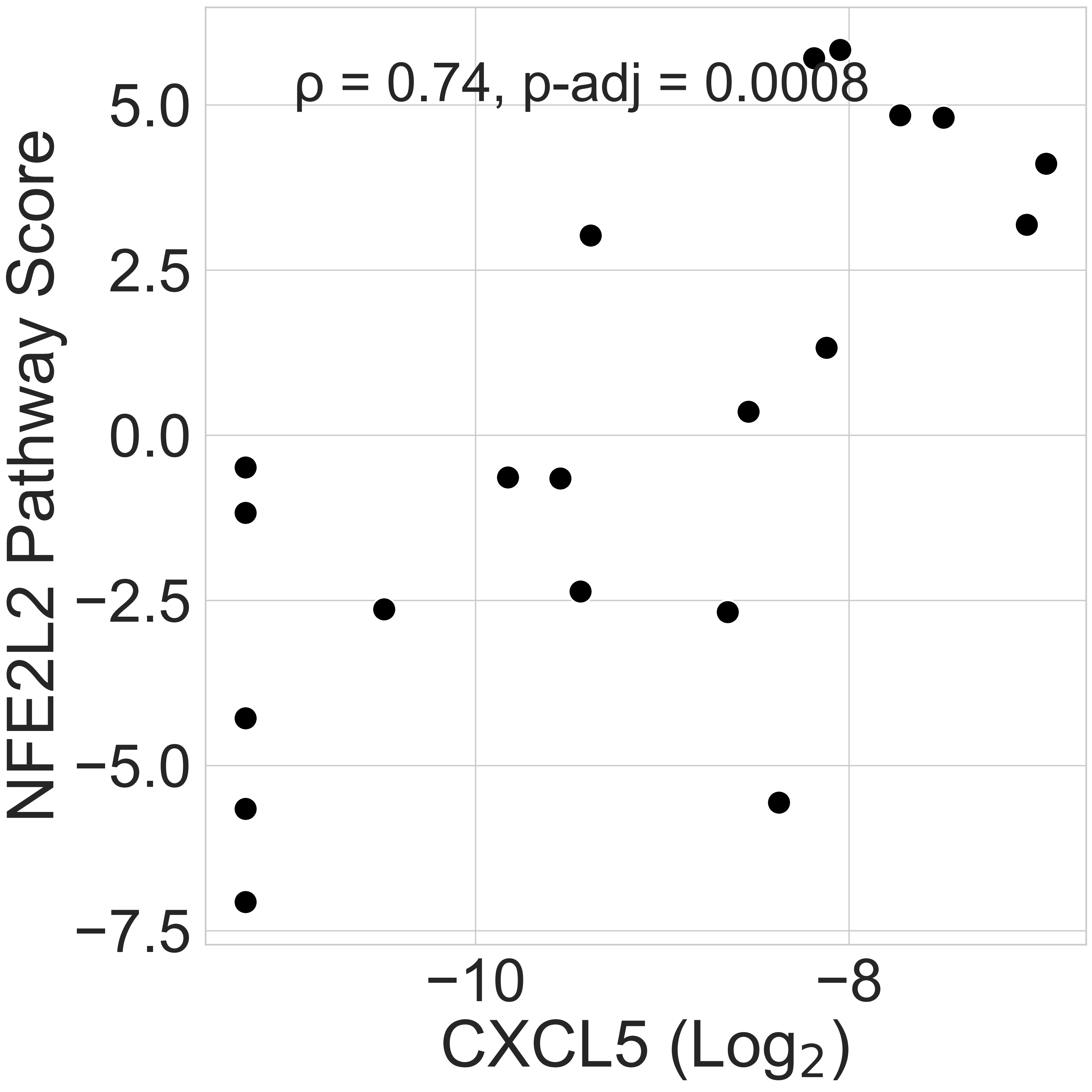
ρ = 0.74, p-adj = 0.0008
NFE2L2 Pathway Score
5.0
2.5
0.0
−2.5
−5.0
−7.5
−10
−8
CXCL5 (Log2)

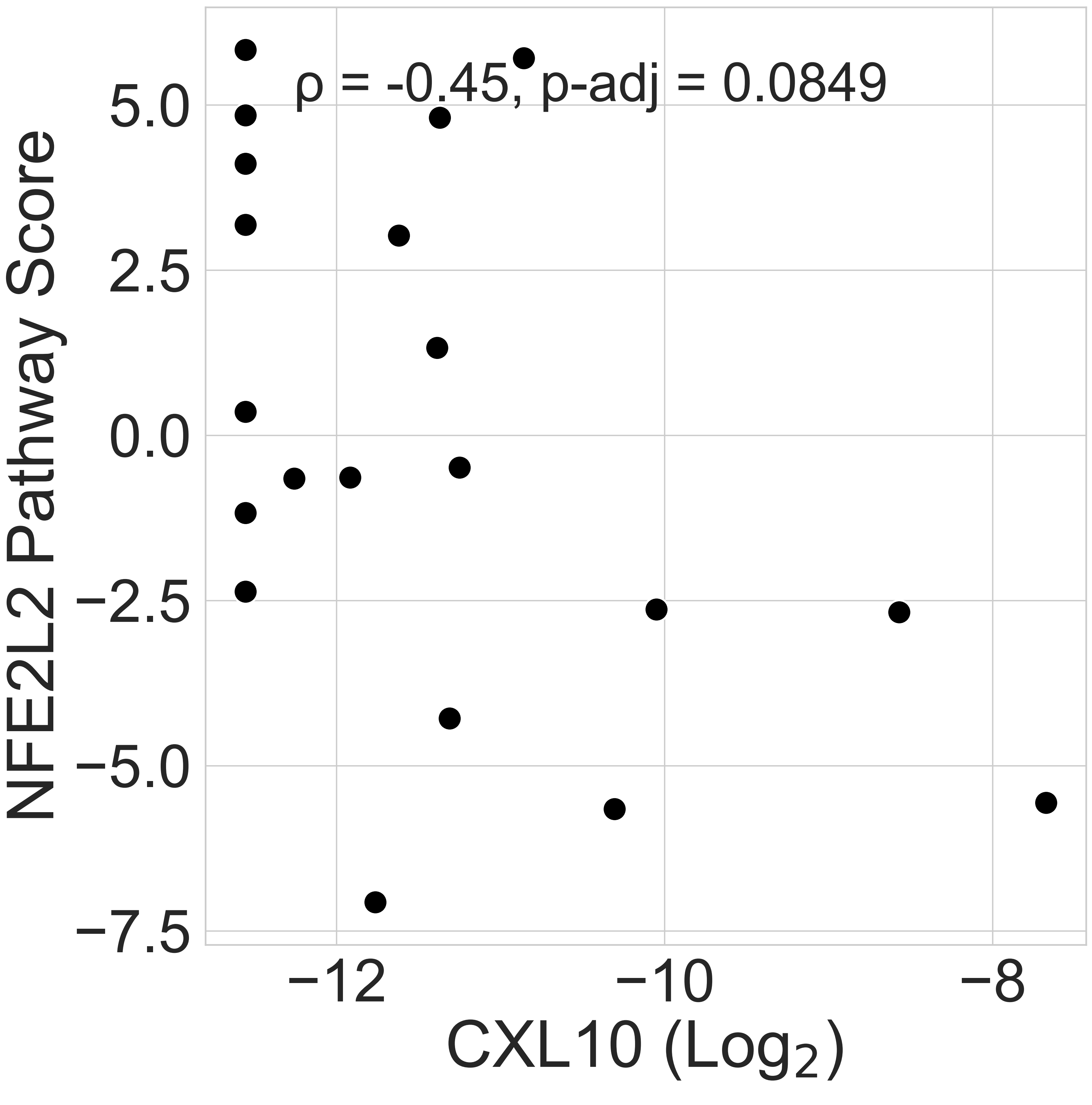
ρ = -0.45, p-adj = 0.0849
NFE2L2 Pathway Score
5.0
2.5
0.0
−2.5
−5.0
−7.5
−12
−10
−8
CXL10 (Log₂)

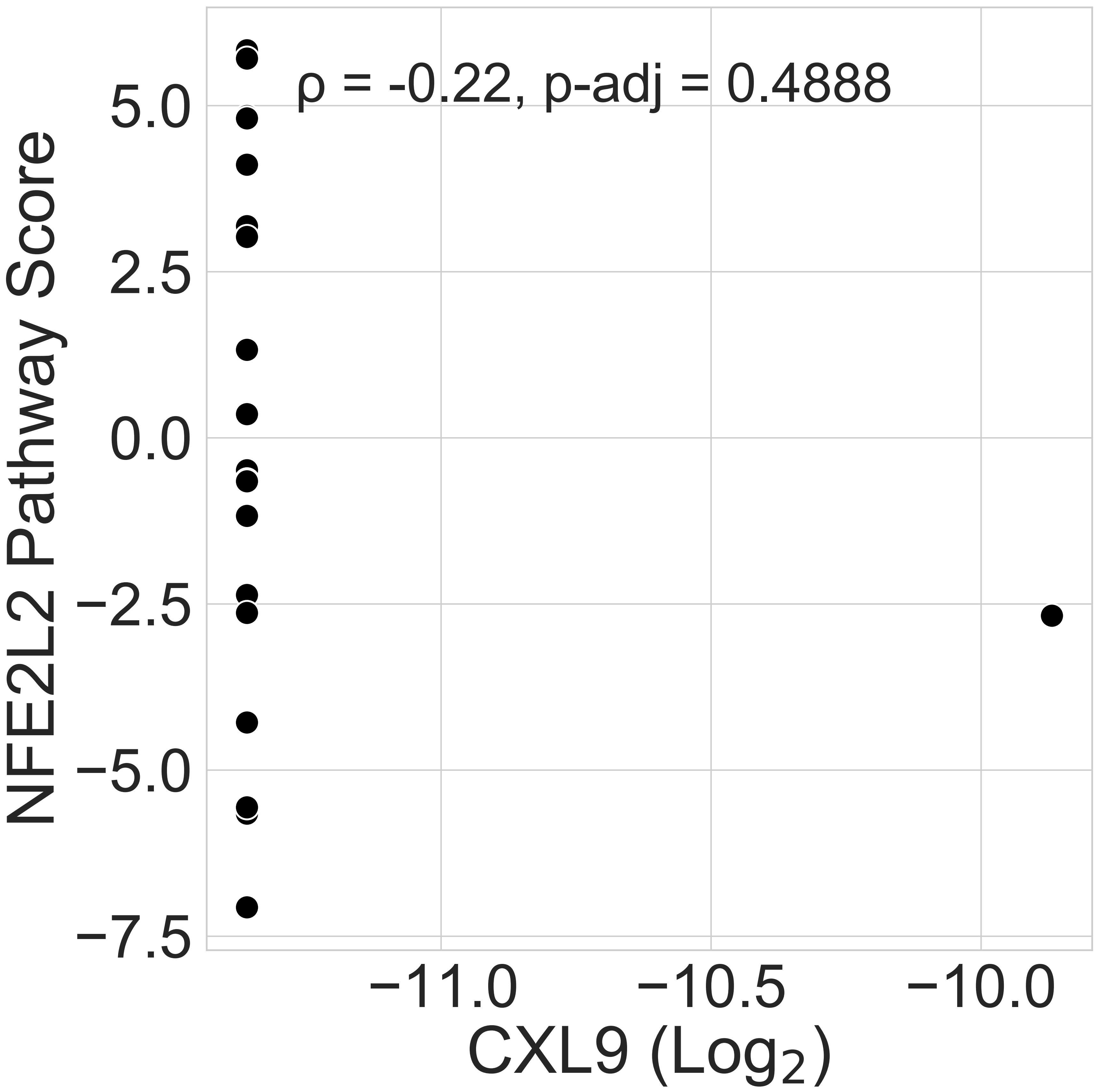
ρ = -0.22, p-adj = 0.4888
5.0
2.5
0.0
−2.5
−5.0
−7.5
NFE2L2 Pathway Score
−11.0
−10.5
−10.0
CXL9 (Log₂)

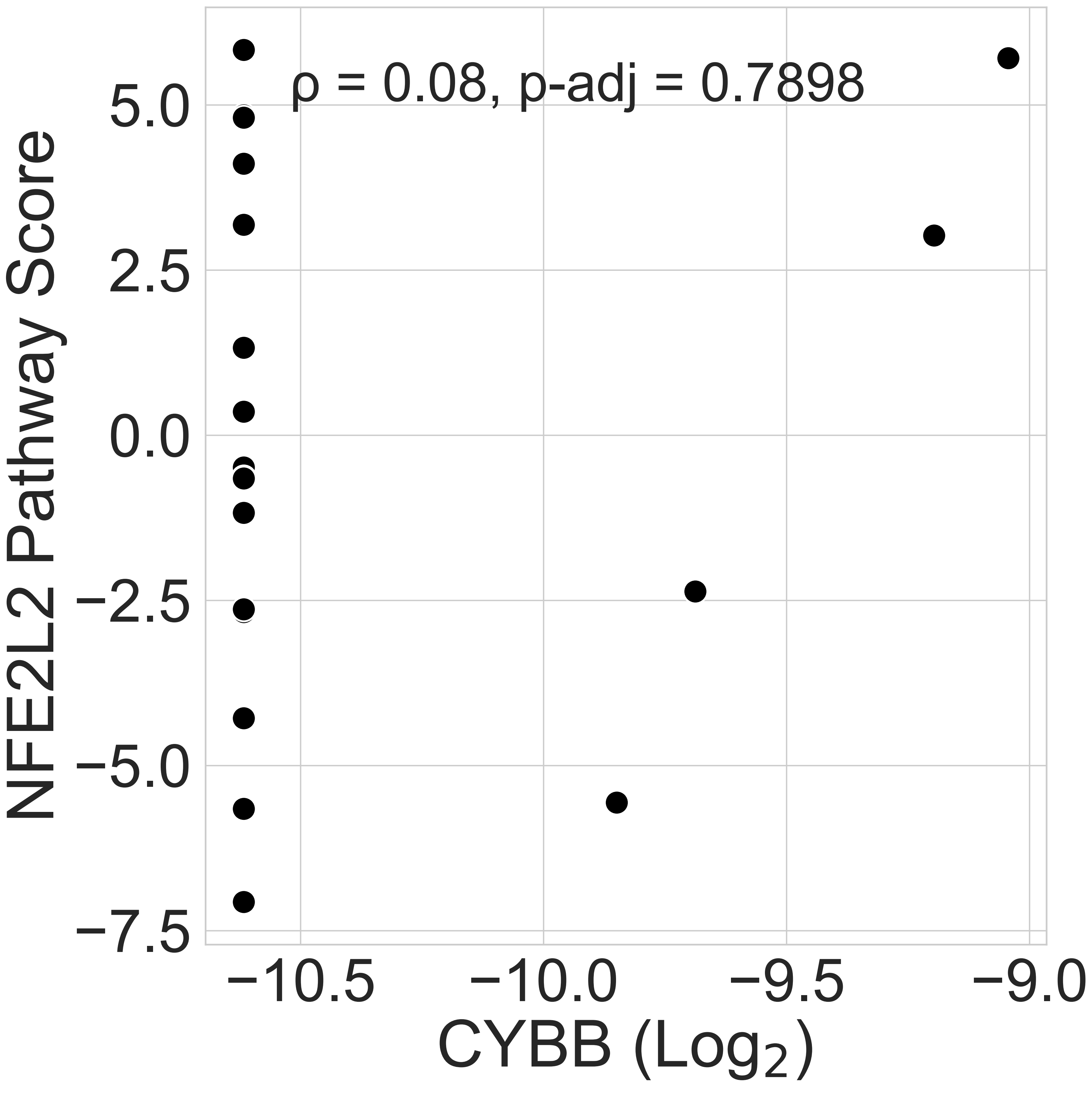
ρ = 0.08, p-adj = 0.7898
NFE2L2 Pathway Score
5.0
2.5
0.0
−2.5
−5.0
−7.5
−10.5
−10.0
−9.5
−9.0
CYBB (Log₂)

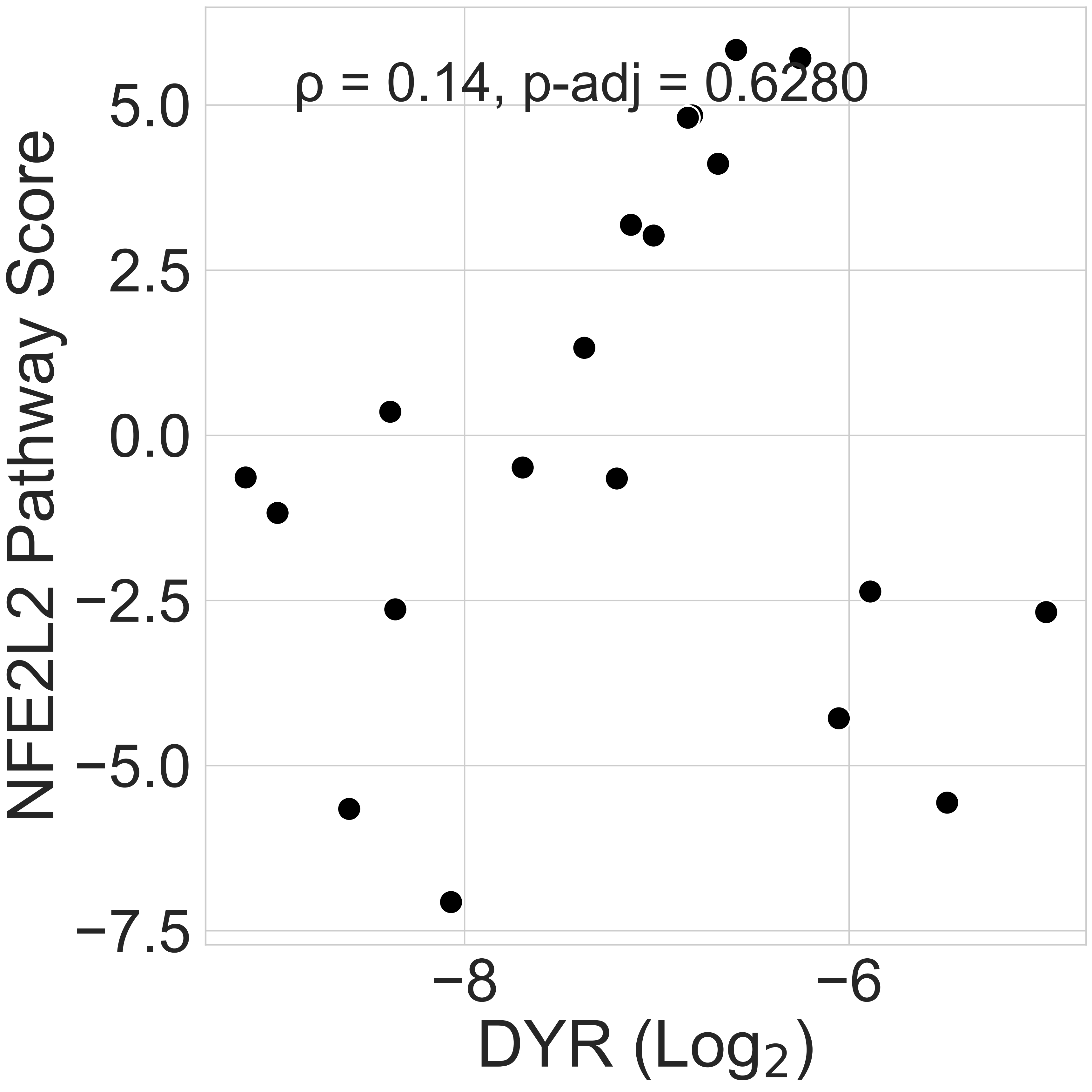
ρ = 0.14, p-adj = 0.6280
5.0
2.5
0.0
−2.5
−5.0
−7.5
NFE2L2 Pathway Score
−8
−6
DYR (Log2)

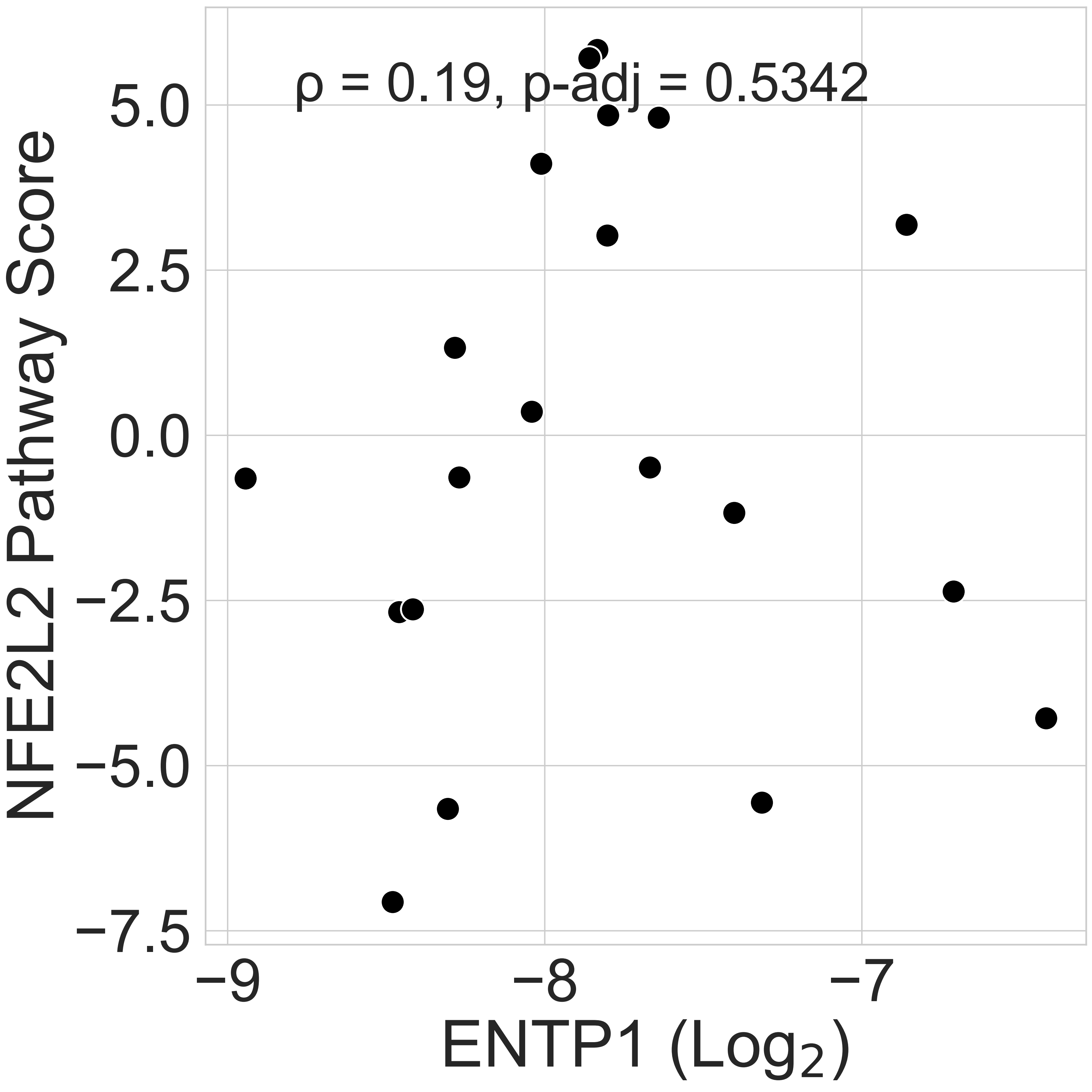
ρ = 0.19, p-adj = 0.5342
NFE2L2 Pathway Score
5.0
2.5
0.0
−2.5
−5.0
−7.5
−9
−8
−7
ENTP1 (Log2)

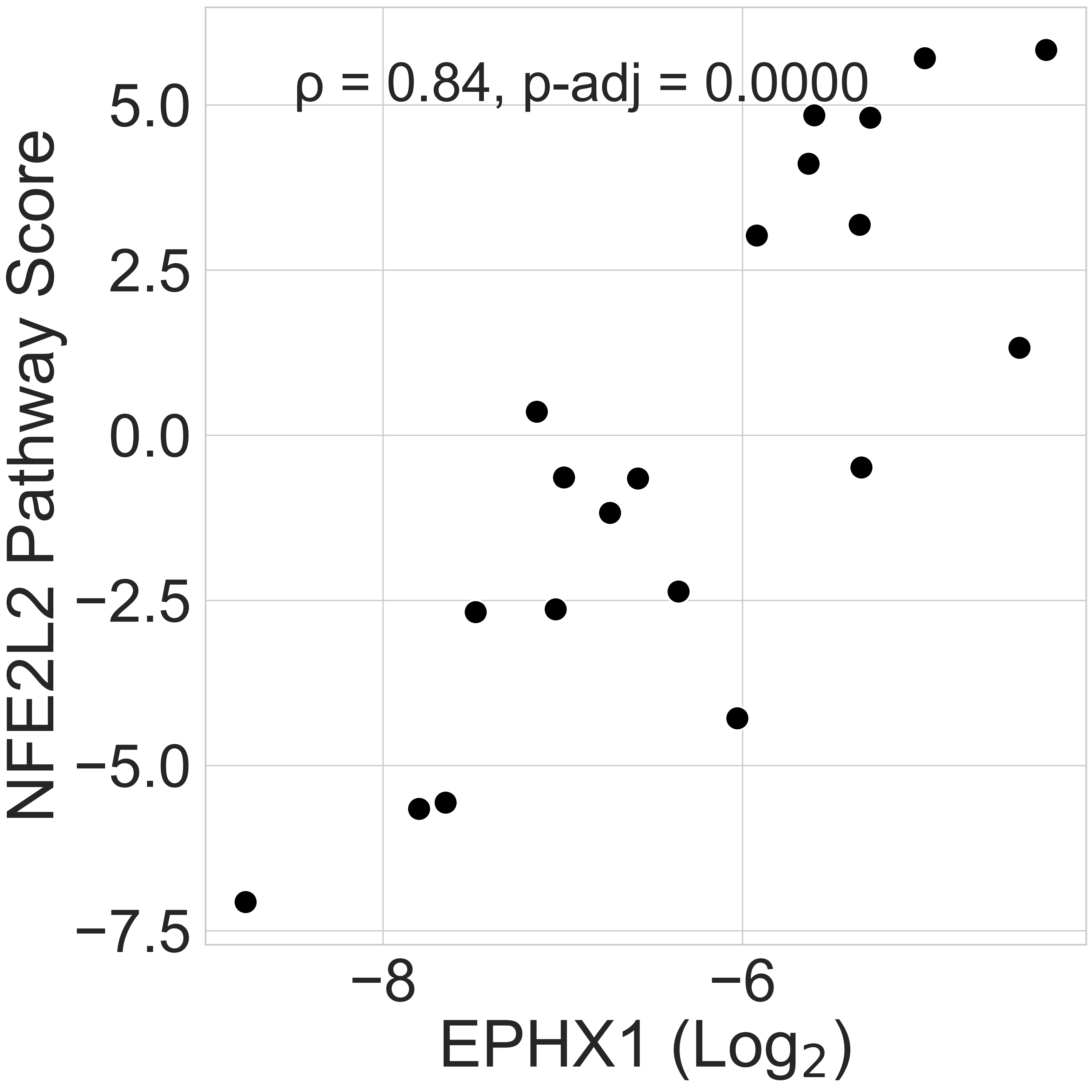

ρ = 0.84, p-adj = 0.0000
NFE2L2 Pathway Score
5.0
2.5
0.0
−2.5
−5.0
−7.5
−8
−6
EPHX1 (Log$_2$)

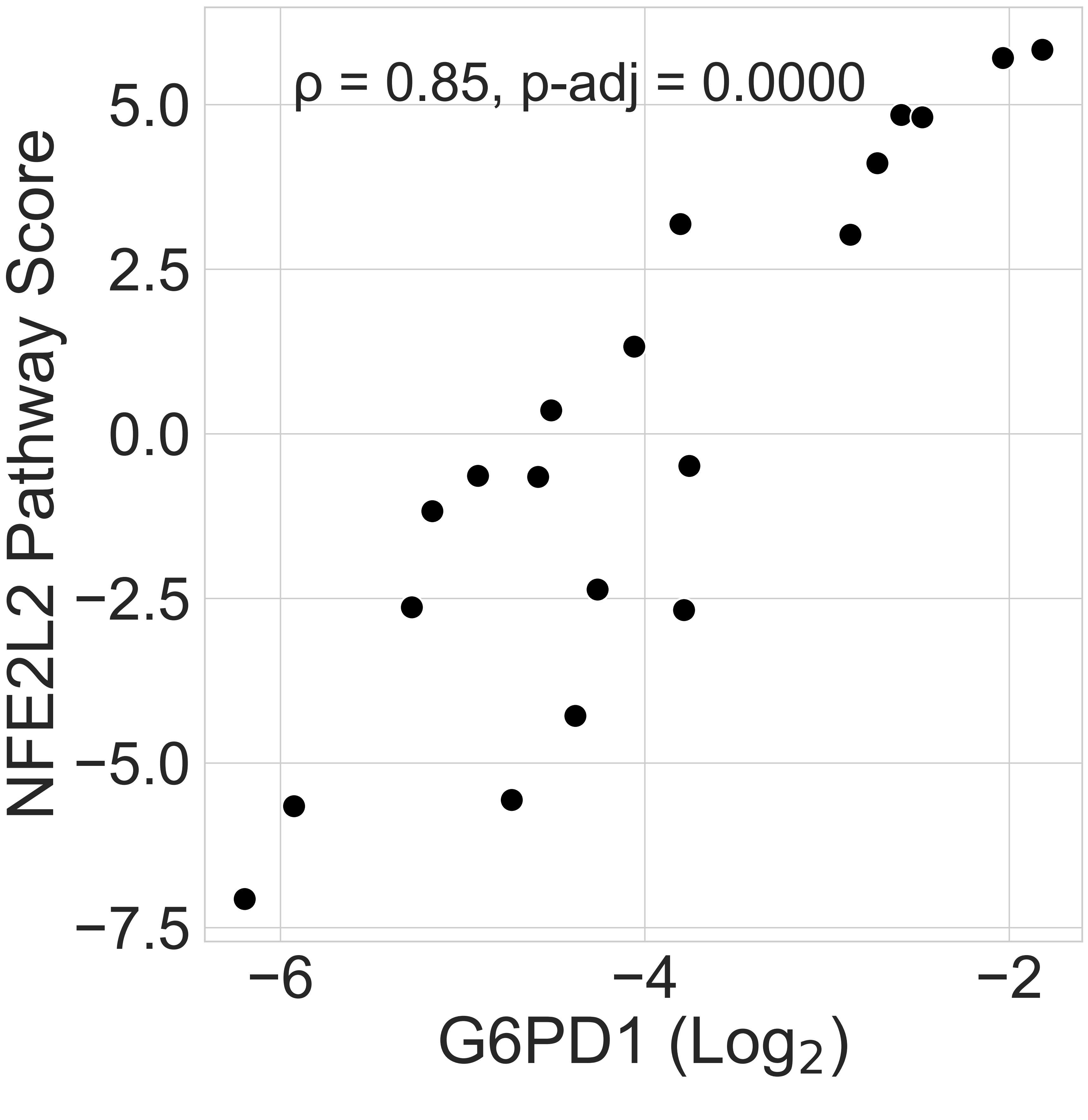
ρ = 0.85, p-adj = 0.0000
NFE2L2 Pathway Score
5.0
2.5
0.0
−2.5
−5.0
−7.5
−6
−4
−2
G6PD1 (Log2)

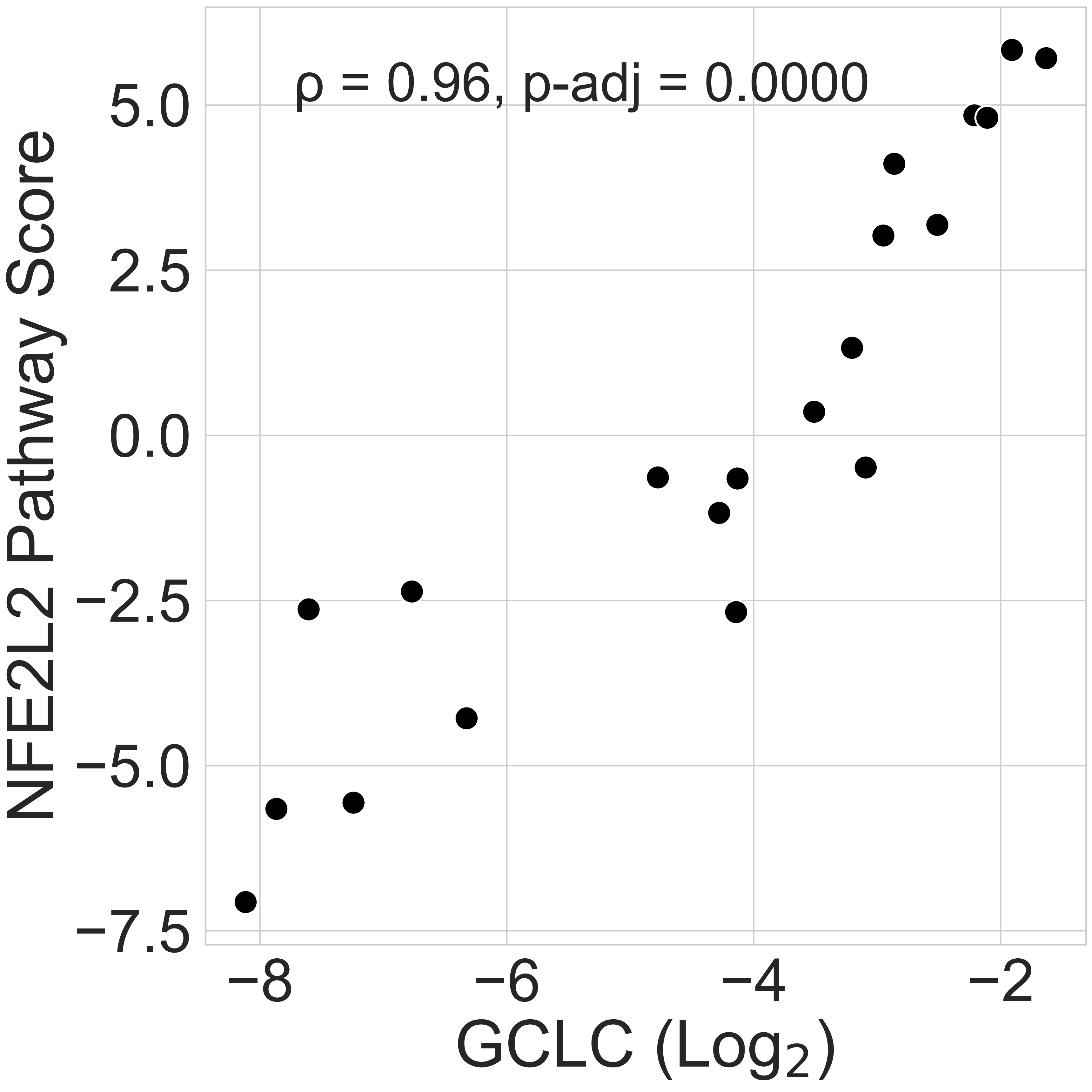
ρ = 0.96, p-adj = 0.0000
NFE2L2 Pathway Score
GCLC (Log₂)
5.0
2.5
0.0
−2.5
−5.0
−7.5
−8
−6
−4
−2

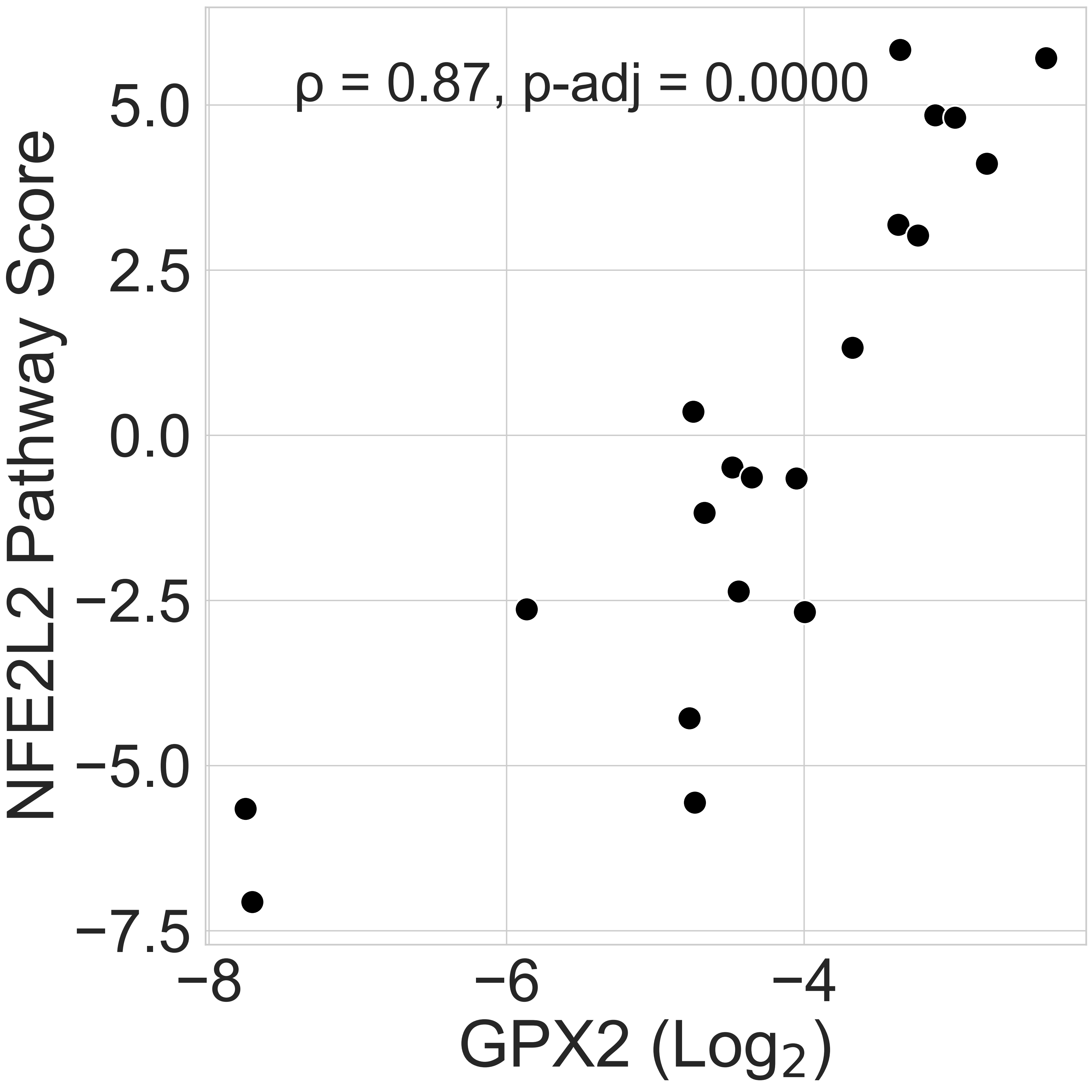
ρ = 0.87, p-adj = 0.0000
NFE2L2 Pathway Score
5.0
2.5
0.0
−2.5
−5.0
−7.5
−8
−6
−4
GPX2 (Log₂)

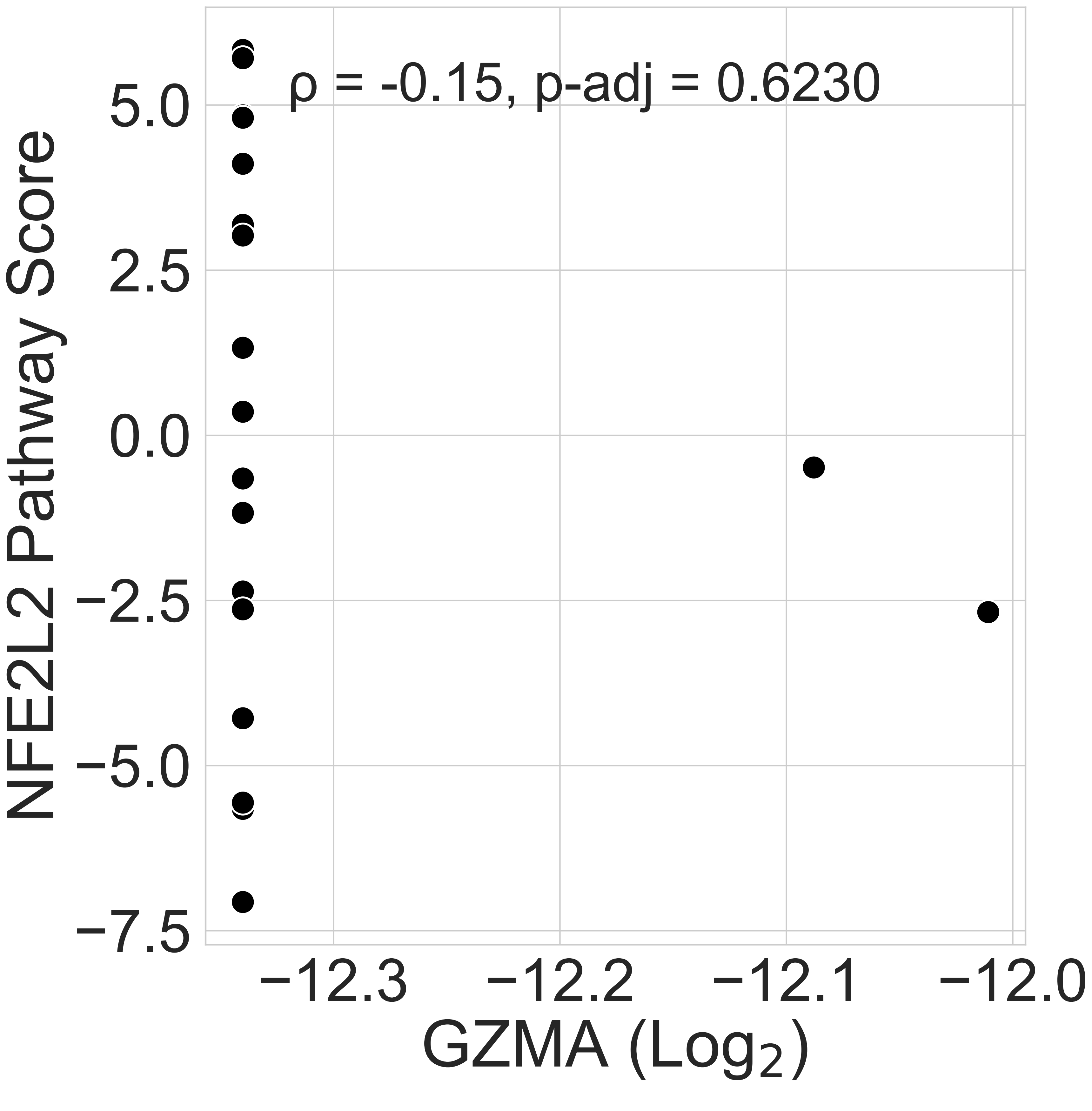

ρ = -0.15, p-adj = 0.6230
NFE2L2 Pathway Score
5.0
2.5
0.0
−2.5
−5.0
−7.5
−12.3
−12.2
−12.1
−12.0
GZMA (Log2)

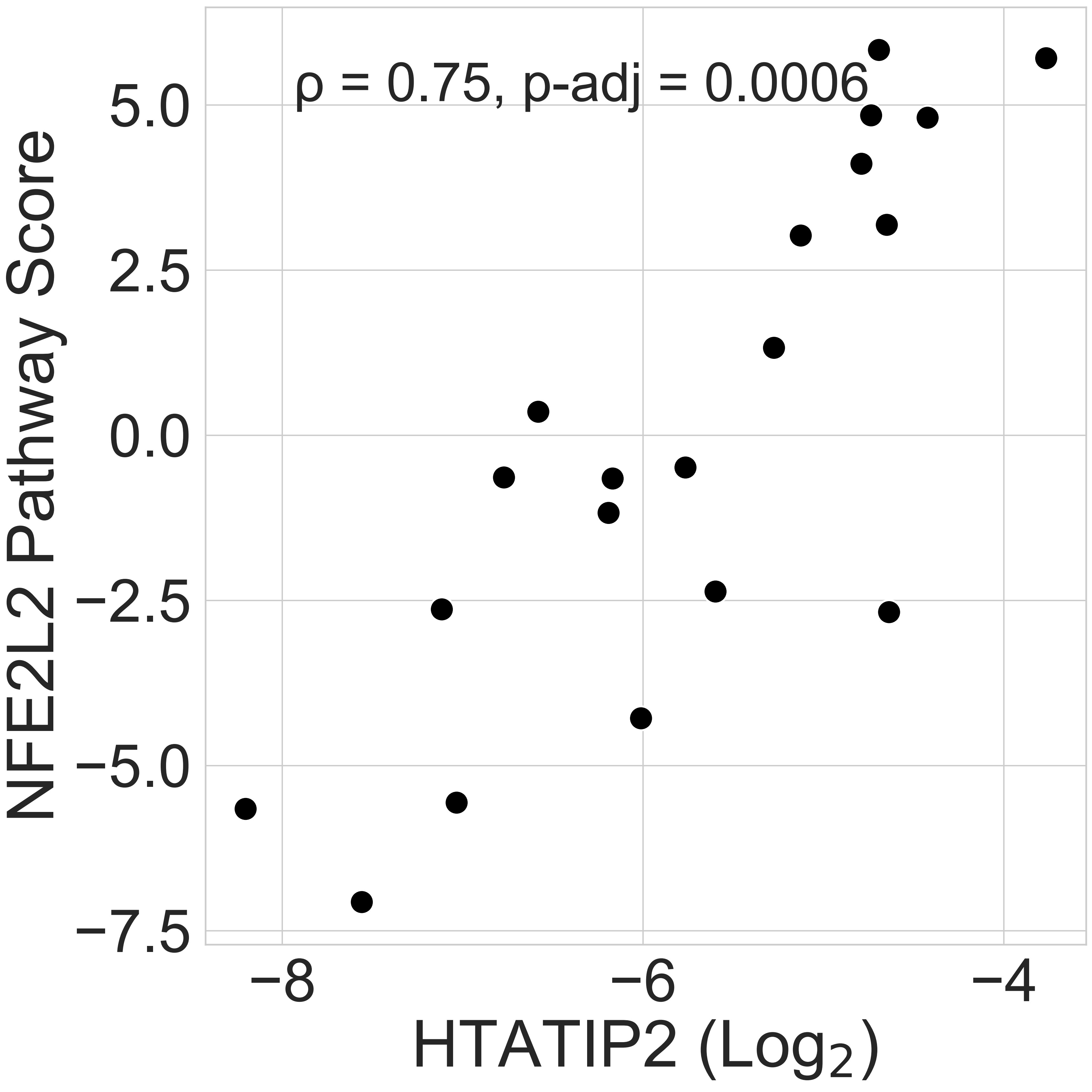

ρ = 0.75, p-adj = 0.0006
5.0
2.5
0.0
−2.5
−5.0
−7.5
NFE2L2 Pathway Score
−8
−6
−4
HTATIP2 (Log2)

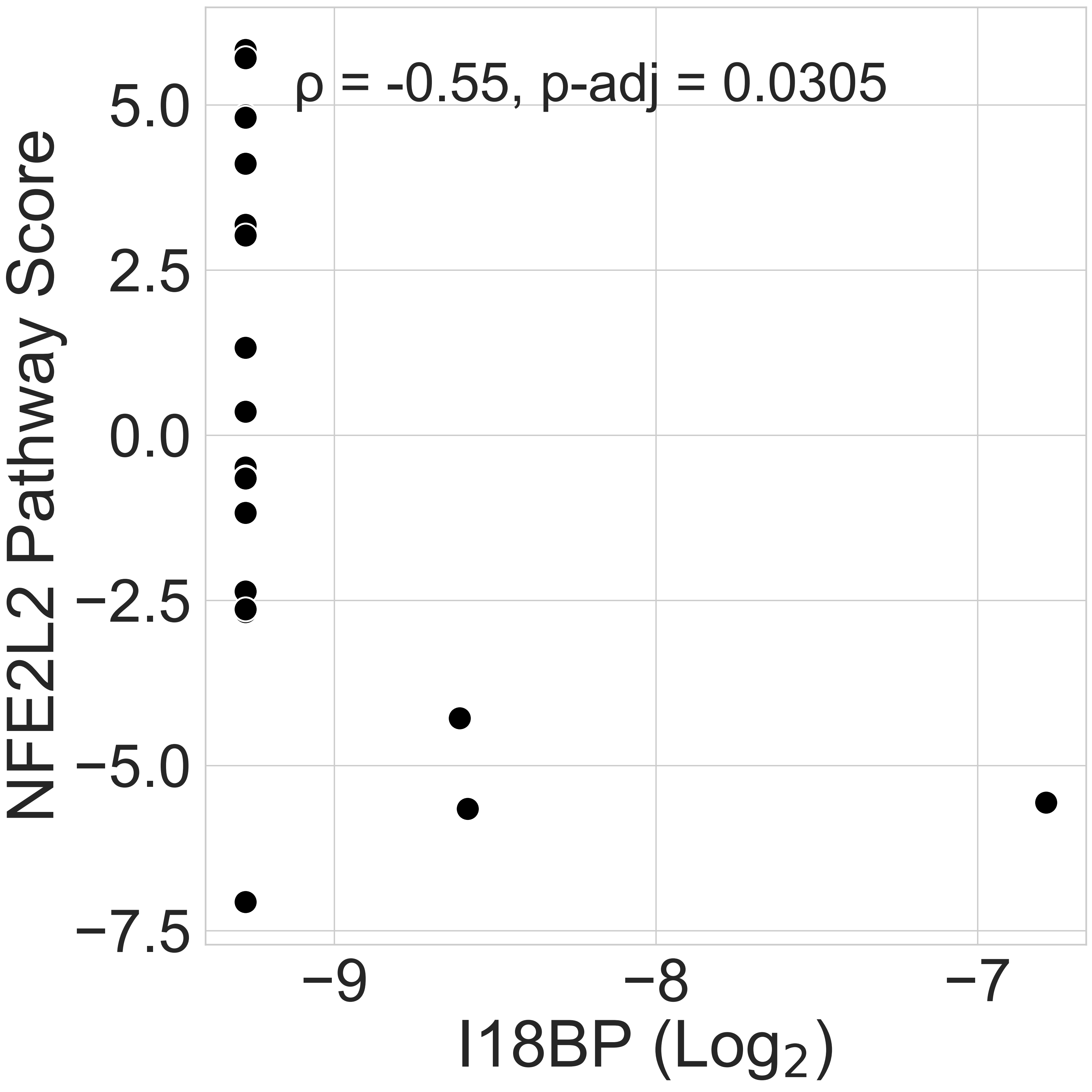

ρ = -0.55, p-adj = 0.0305
NFE2L2 Pathway Score
5.0
2.5
0.0
−2.5
−5.0
−7.5
−9
−8
−7
I18BP (Log2)

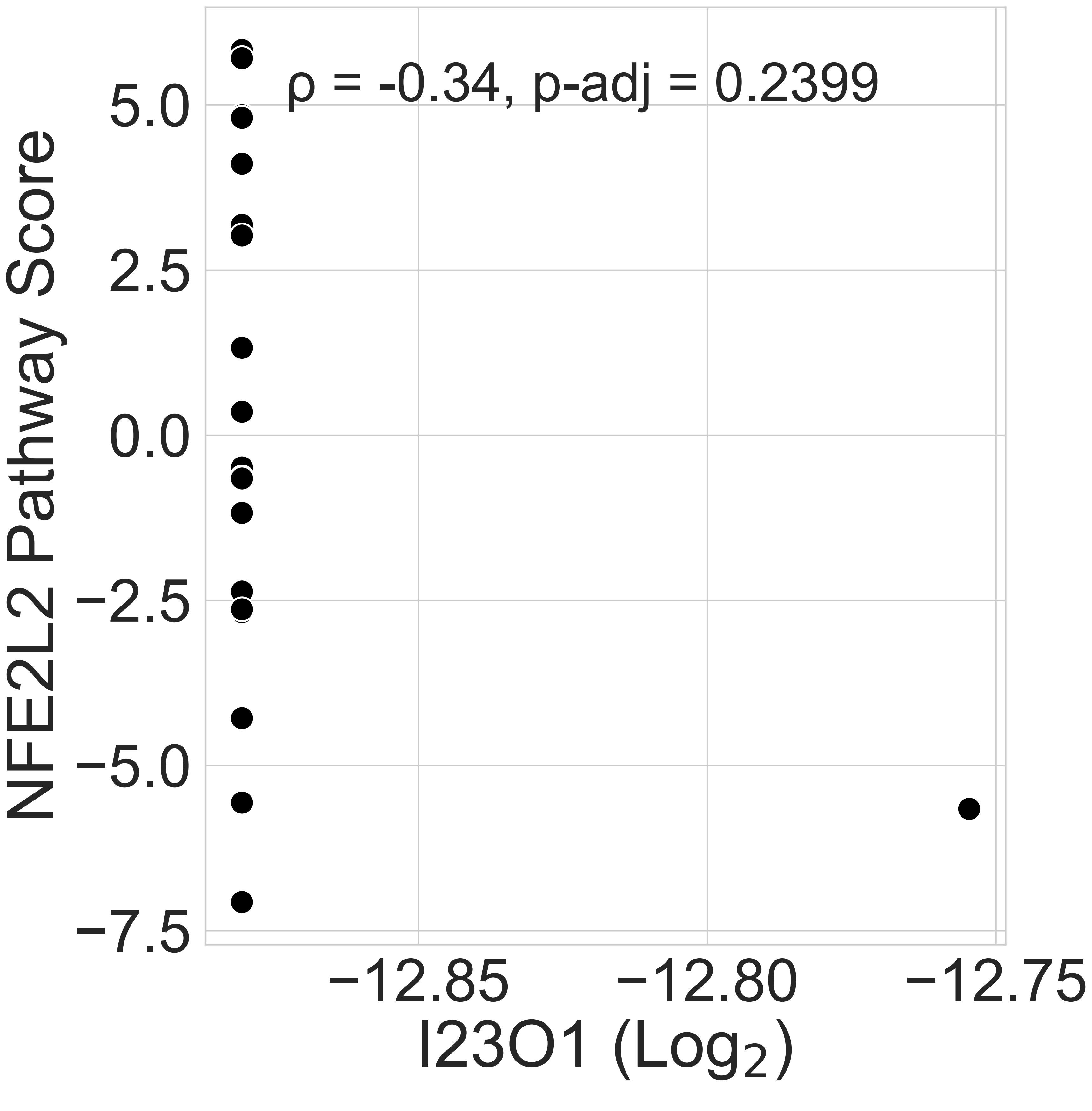

ρ = -0.34, p-adj = 0.2399
NFE2L2 Pathway Score
5.0
2.5
0.0
−2.5
−5.0
−7.5
−12.85
−12.80
−12.75
I23O1 (Log2)

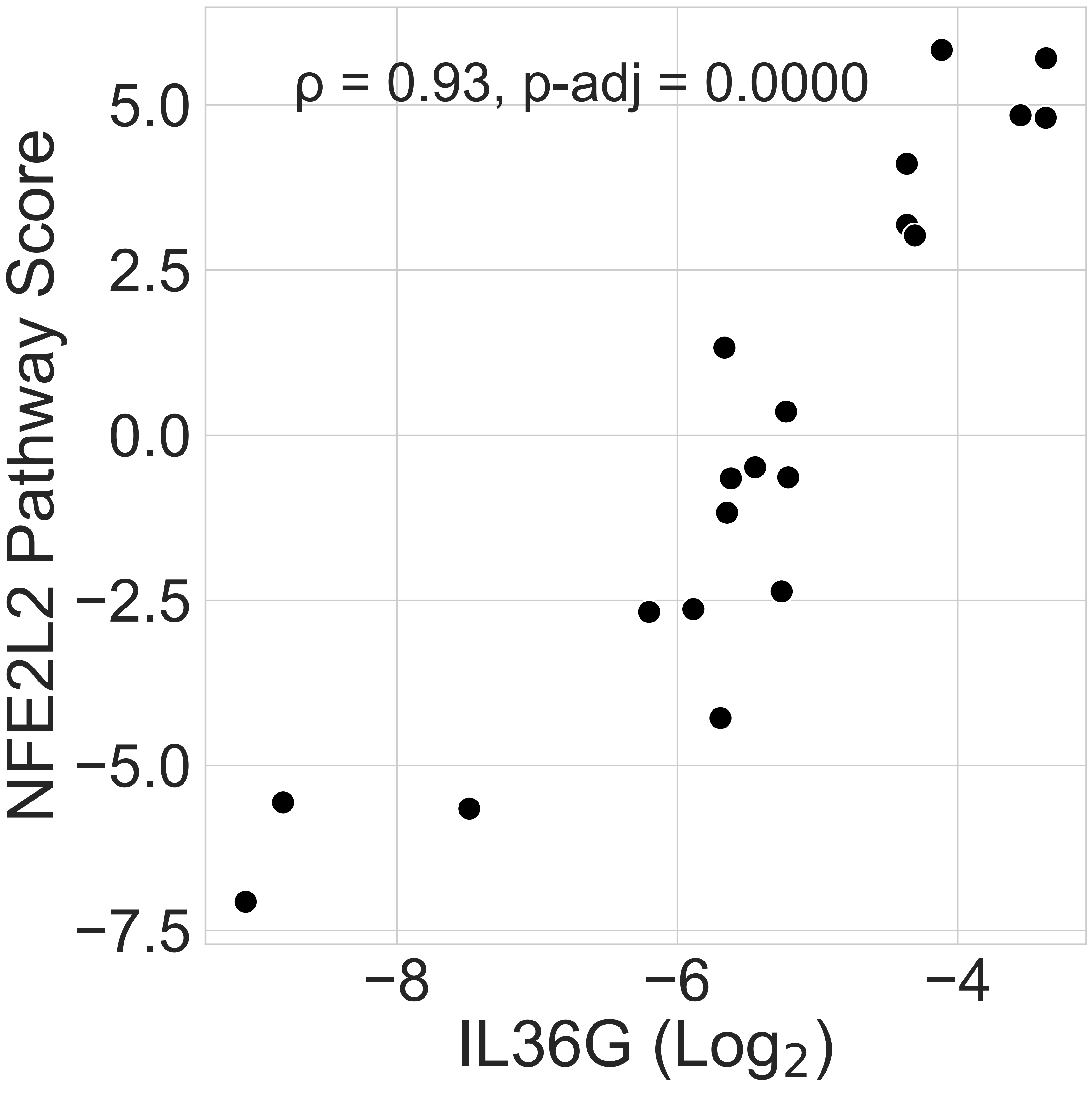
ρ = 0.93, p-adj = 0.0000
NFE2L2 Pathway Score
5.0
2.5
0.0
−2.5
−5.0
−7.5
−8
−6
−4
IL36G (Log₂)

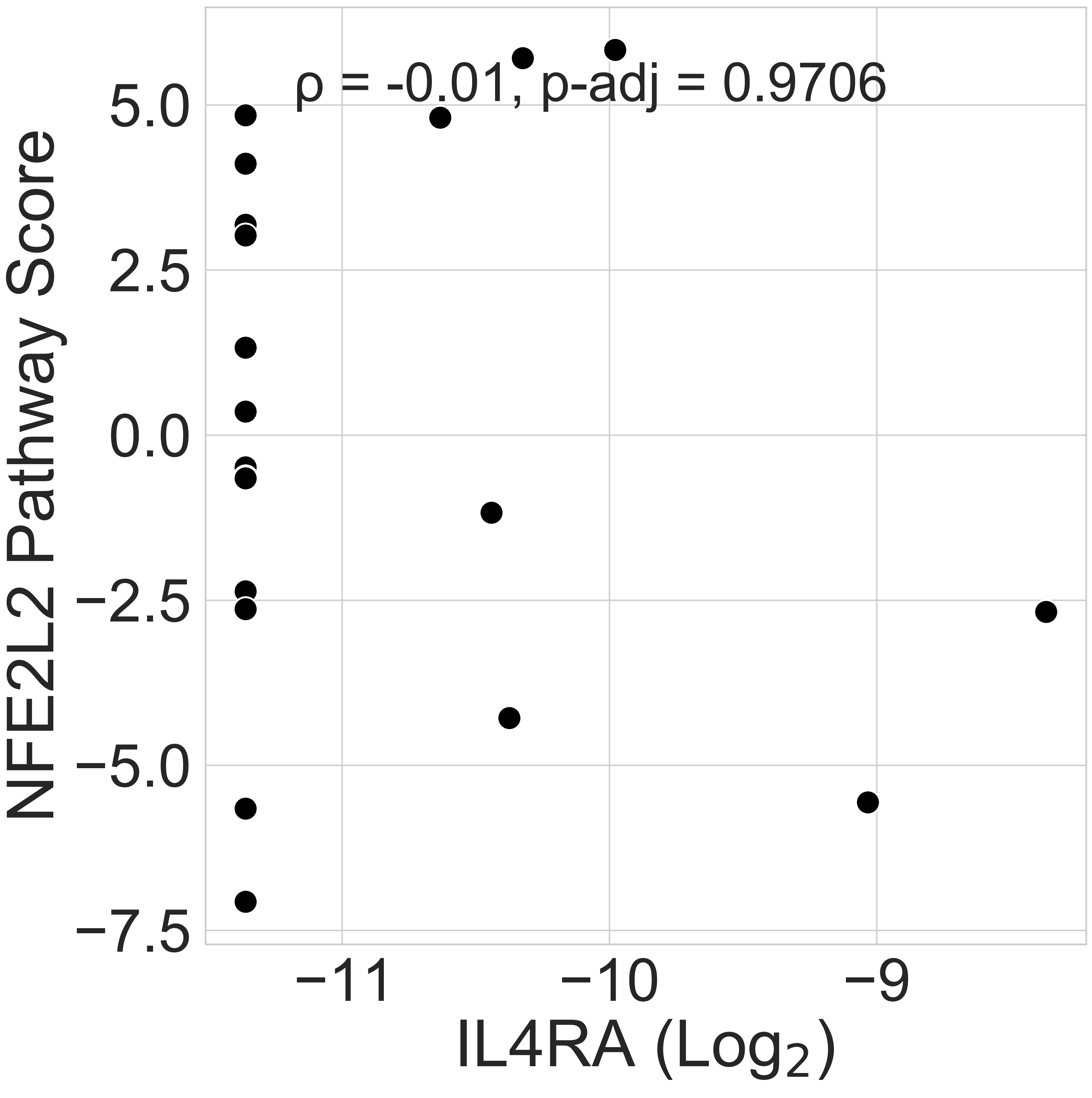
ρ = -0.01, p-adj = 0.9706
NFE2L2 Pathway Score
5.0
2.5
0.0
−2.5
−5.0
−7.5
−11
−10
−9
IL4RA (Log2)

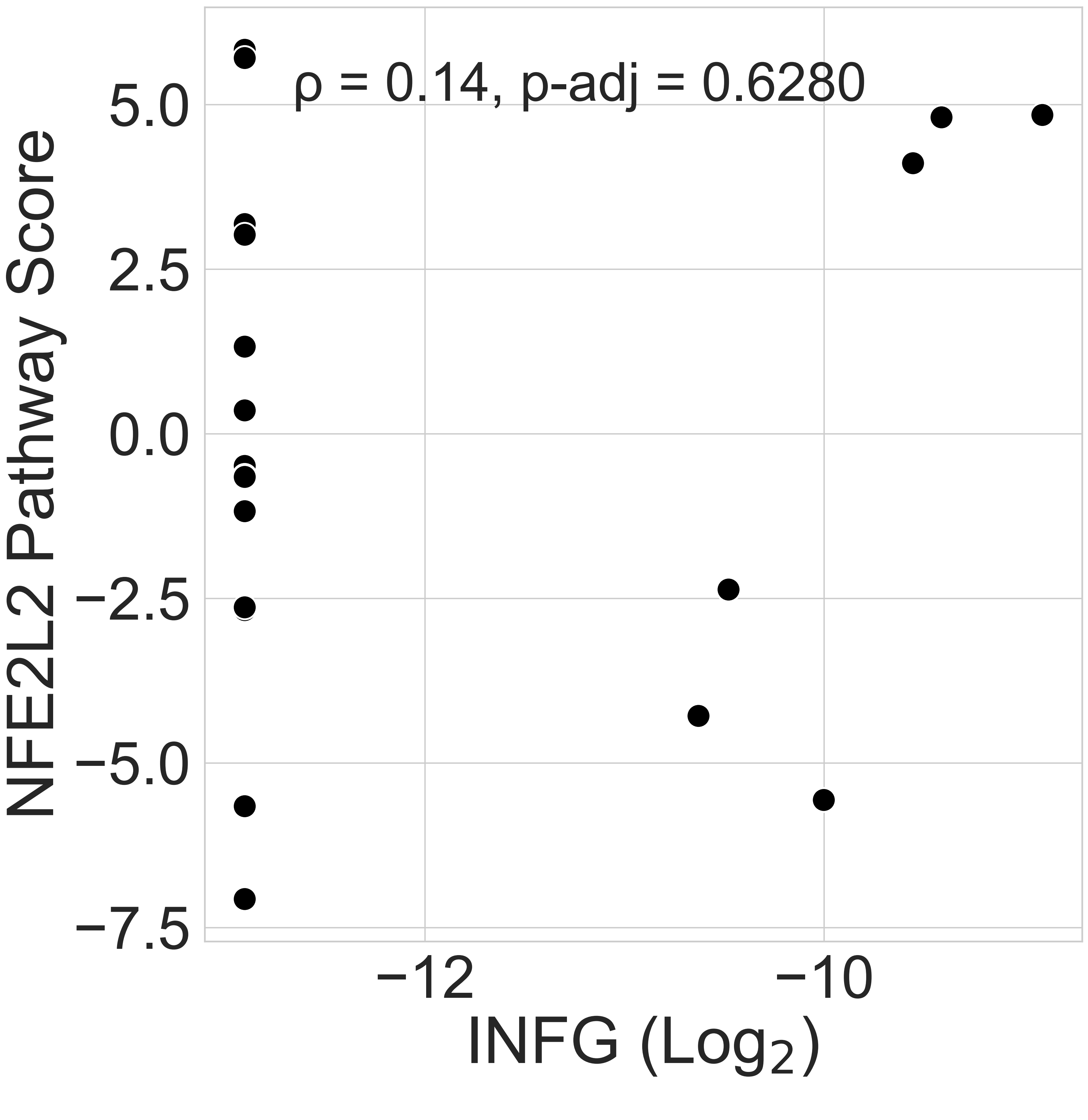

ρ = 0.14, p-adj = 0.6280
NFE2L2 Pathway Score
5.0
2.5
0.0
−2.5
−5.0
−7.5
−12
−10
INFG (Log2)

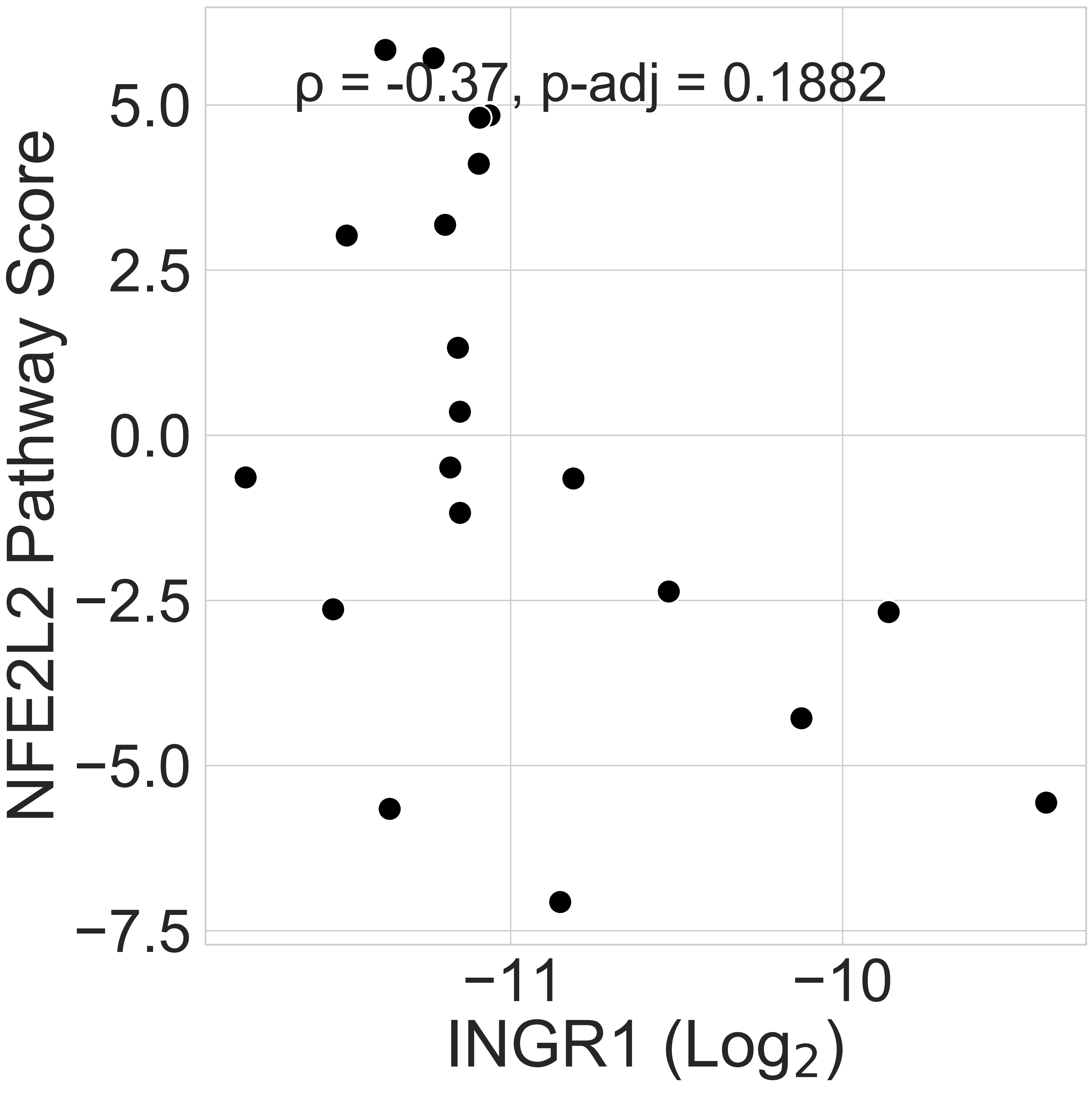
ρ = -0.37, p-adj = 0.1882
NFE2L2 Pathway Score
5.0
2.5
0.0
−2.5
−5.0
−7.5
−11
−10
INGR1 (Log2)

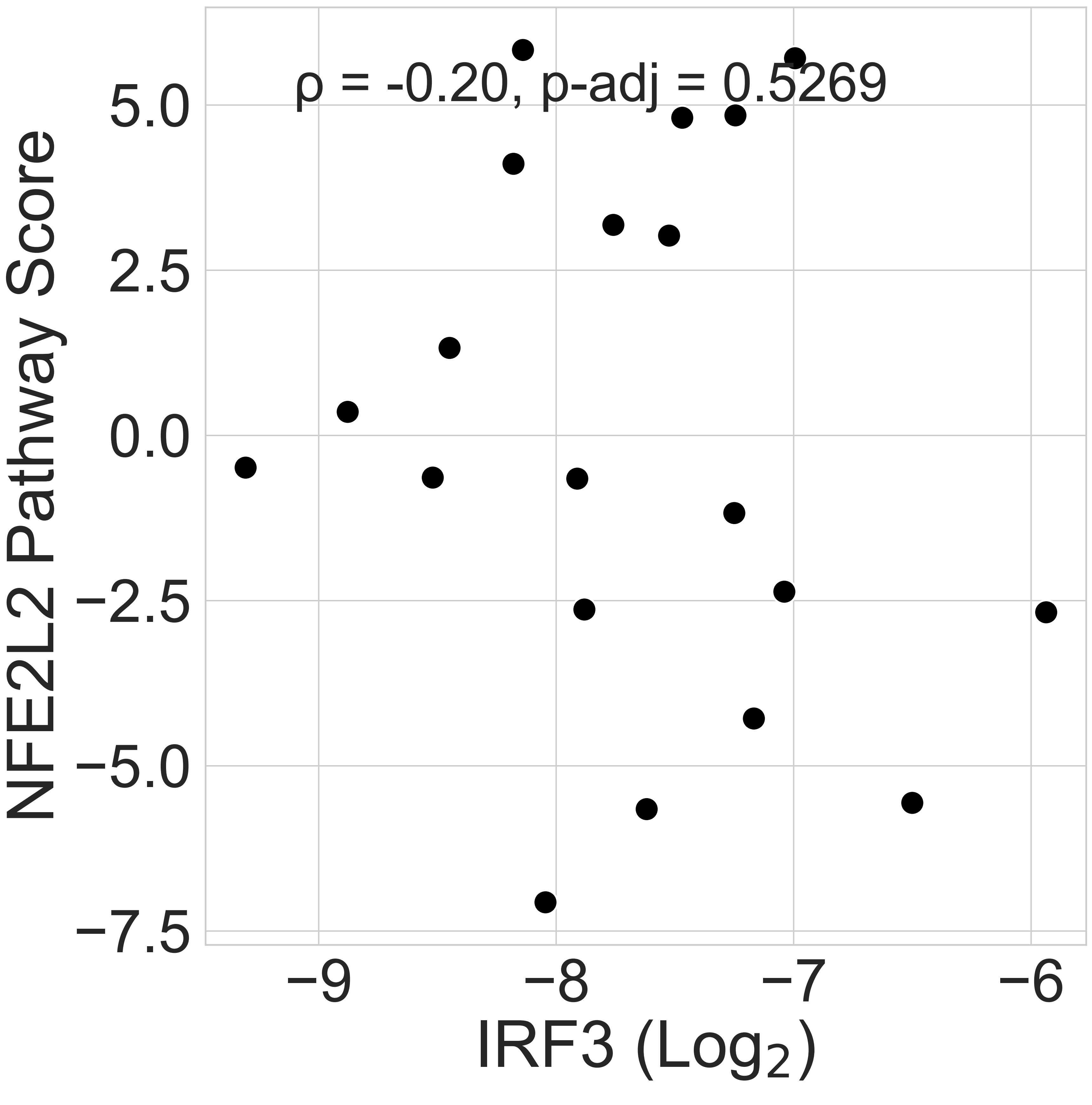
ρ = -0.20, p-adj = 0.5269
5.0
2.5
0.0
−2.5
−5.0
−7.5
NFE2L2 Pathway Score
−9
−8
−7
−6
IRF3 (Log2)

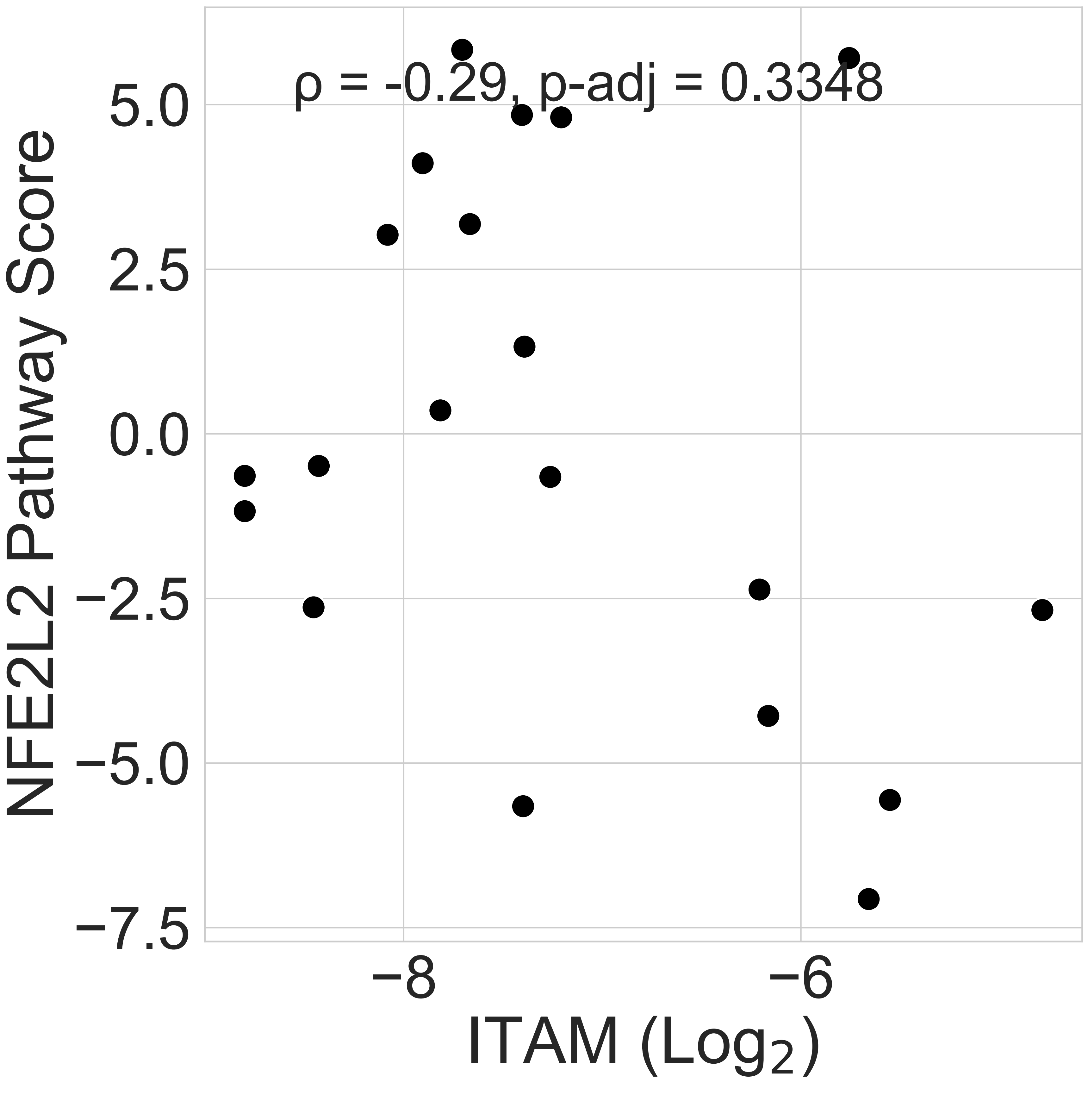

ρ = -0.29, p-adj = 0.3348
5.0
2.5
0.0
−2.5
−5.0
−7.5
NFE2L2 Pathway Score
−8
−6
ITAM (Log2)

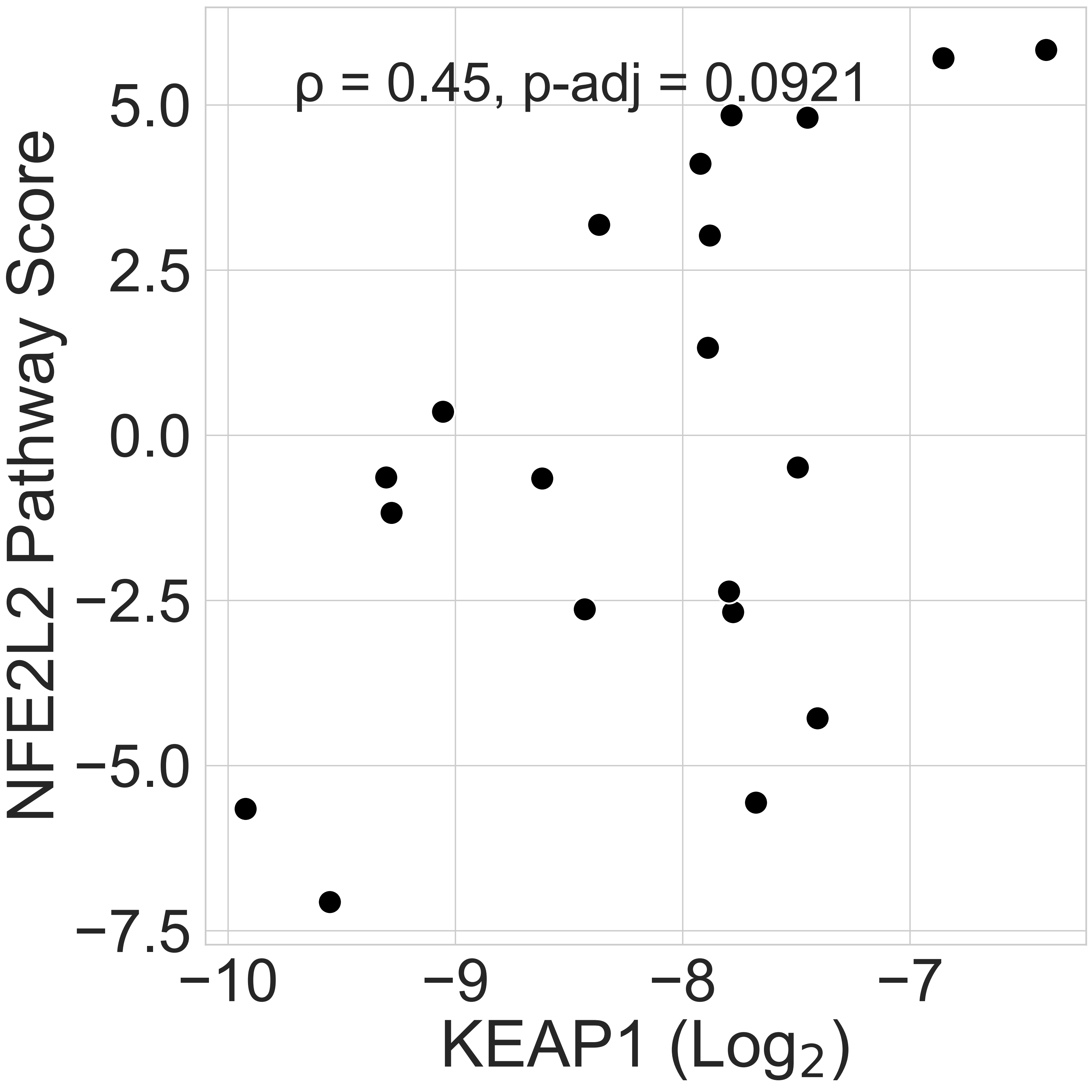

ρ = 0.45, p-adj = 0.0921
5.0
2.5
0.0
−2.5
−5.0
−7.5
NFE2L2 Pathway Score
−10
−9
−8
−7
KEAP1 (Log₂)

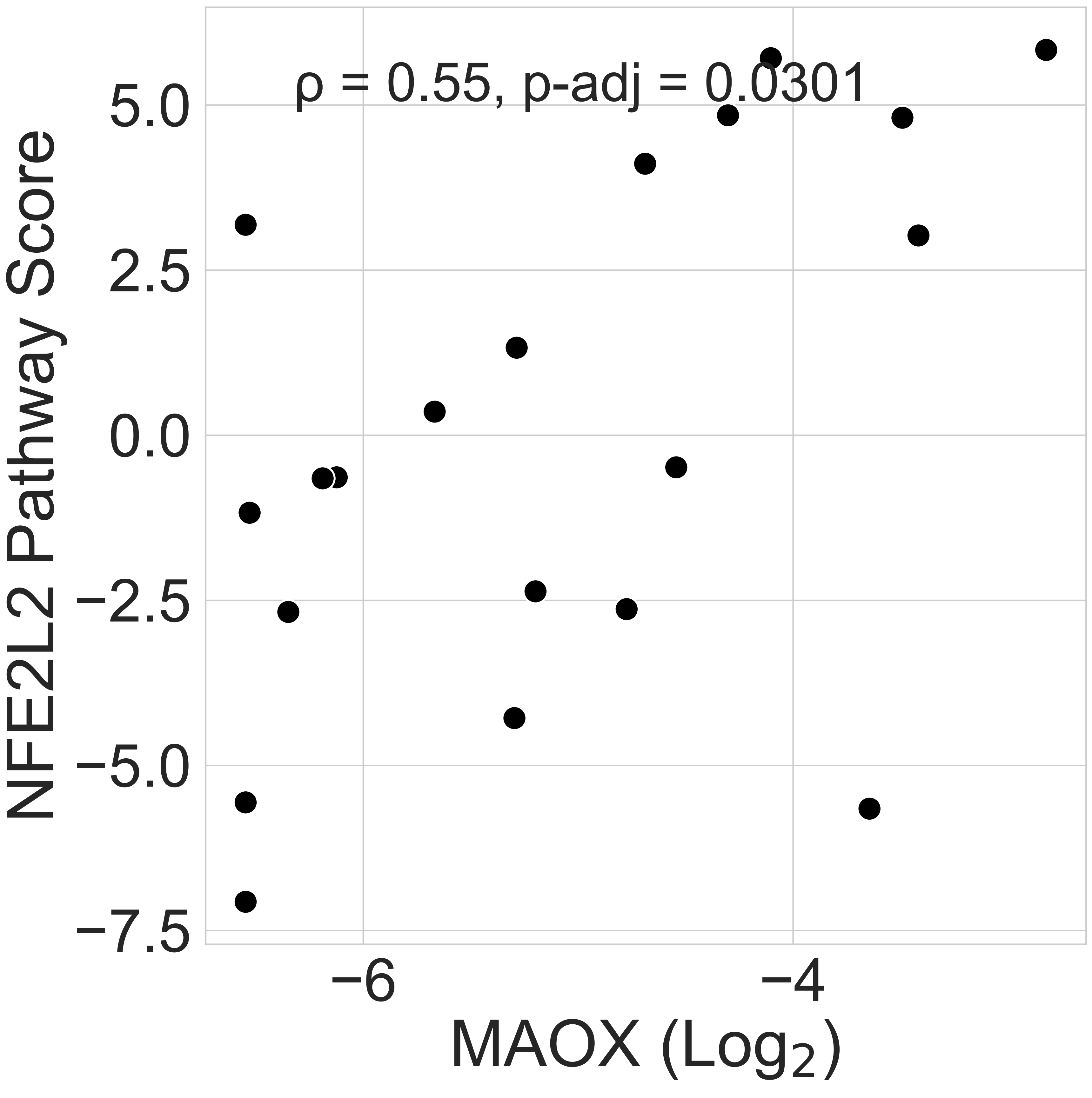

ρ = 0.55, p-adj = 0.0301
NFE2L2 Pathway Score
5.0
2.5
0.0
−2.5
−5.0
−7.5
−6
−4
MAOX (Log2)

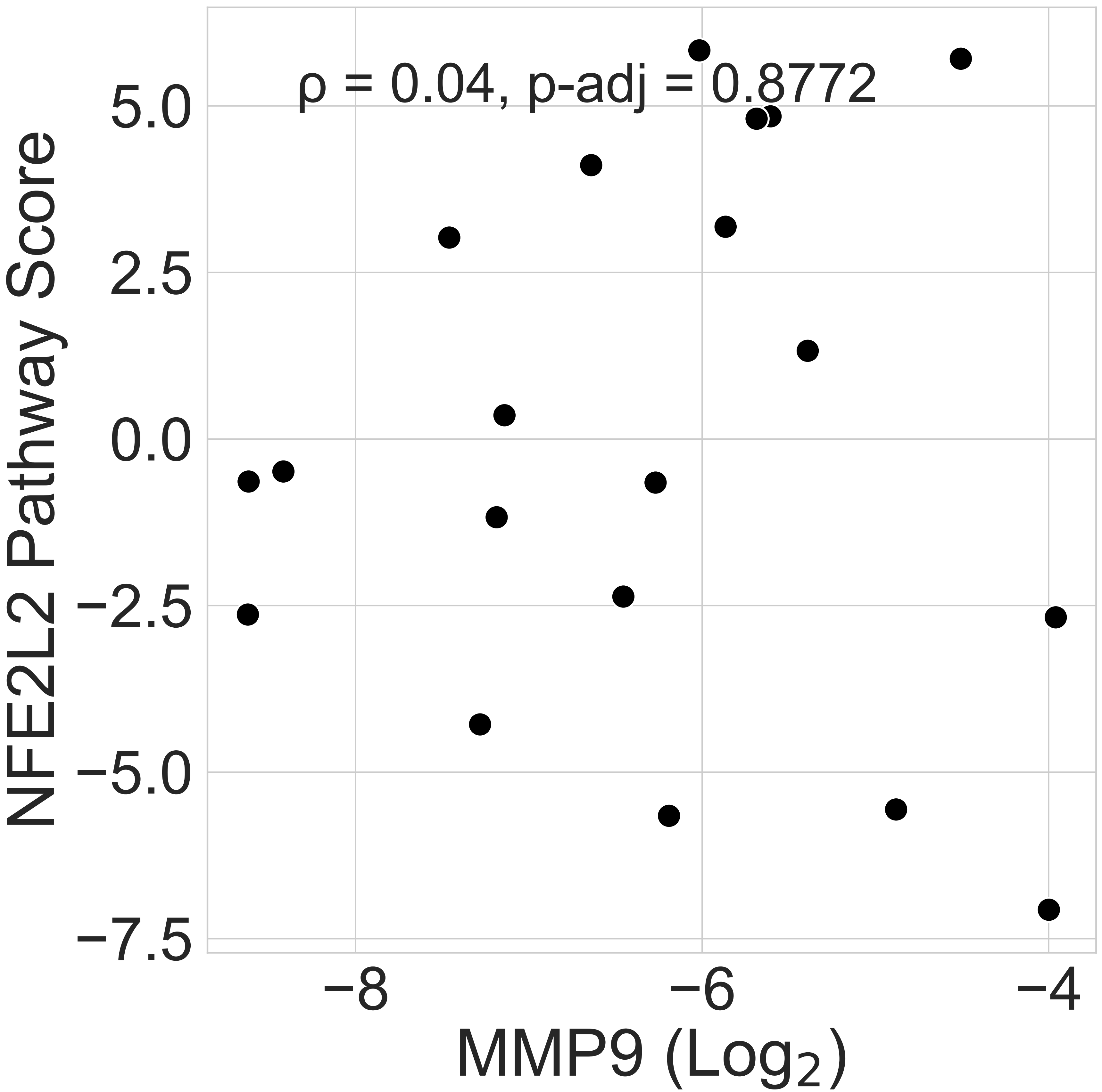
ρ = 0.04, p-adj = 0.8772
NFE2L2 Pathway Score
5.0
2.5
0.0
−2.5
−5.0
−7.5
−8
−6
−4
MMP9 (Log₂)

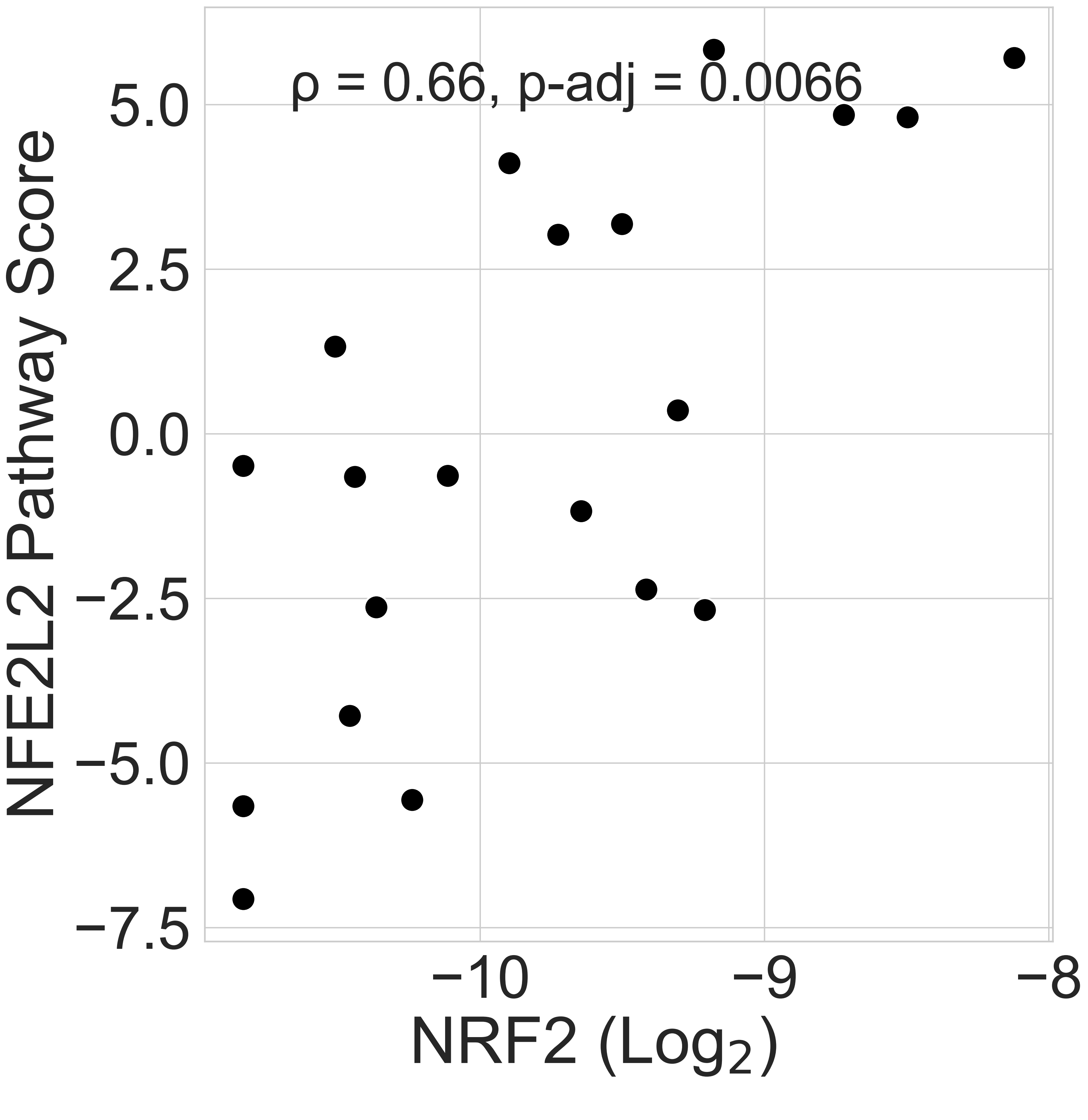
ρ = 0.66, p-adj = 0.0066
NFE2L2 Pathway Score
5.0
2.5
0.0
−2.5
−5.0
−7.5
−10
−9
−8
NRF2 (Log2)

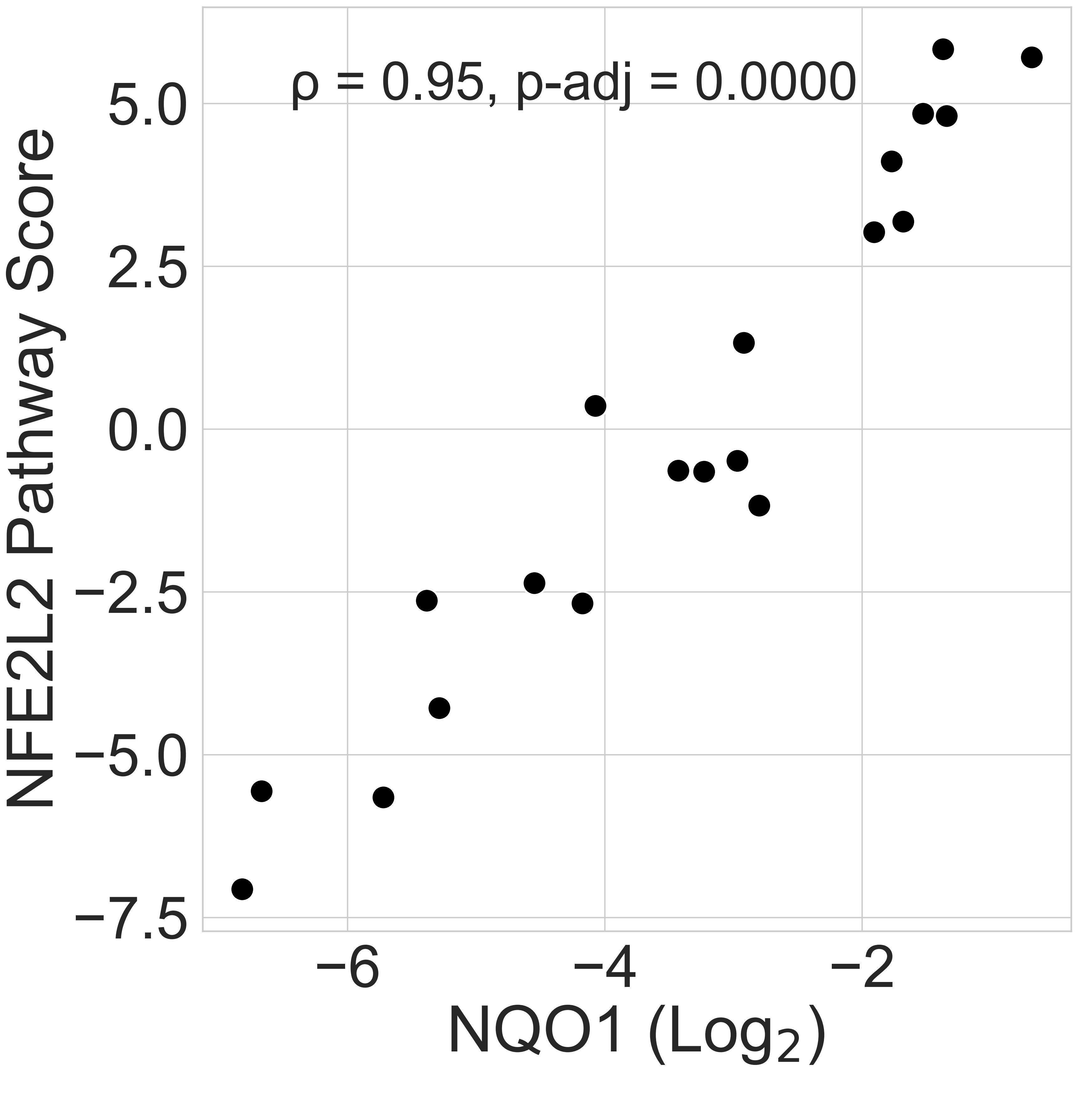

ρ = 0.95, p-adj = 0.0000
NFE2L2 Pathway Score
5.0
2.5
0.0
−2.5
−5.0
−7.5
−6
−4
−2
NQO1 (Log₂)

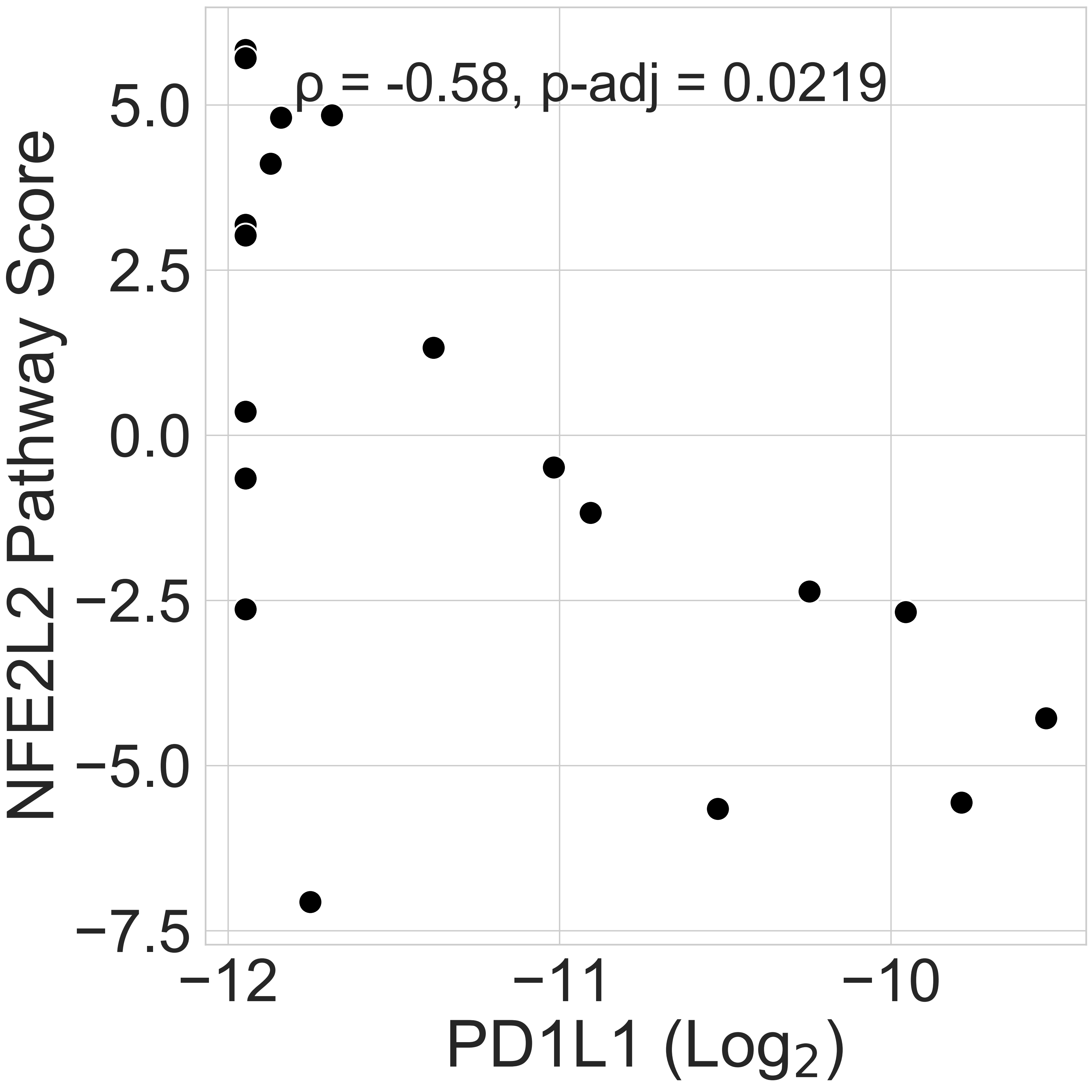

ρ = -0.58, p-adj = 0.0219
NFE2L2 Pathway Score
5.0
2.5
0.0
−2.5
−5.0
−7.5
−12
−11
−10
PD1L1 (Log₂)

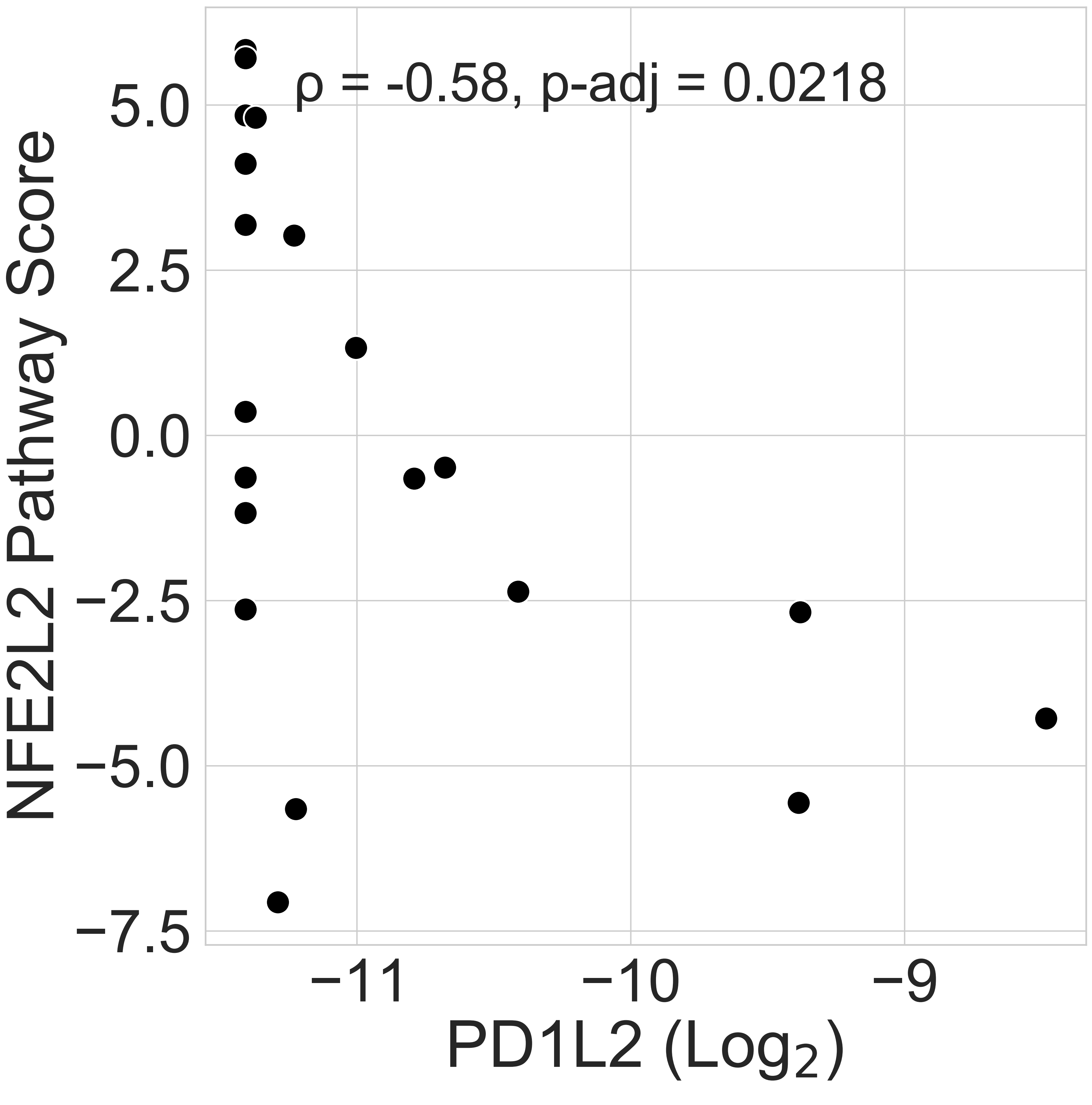

ρ = -0.58, p-adj = 0.0218
NFE2L2 Pathway Score
5.0
2.5
0.0
−2.5
−5.0
−7.5
−11
−10
−9
PD1L2 (Log₂)

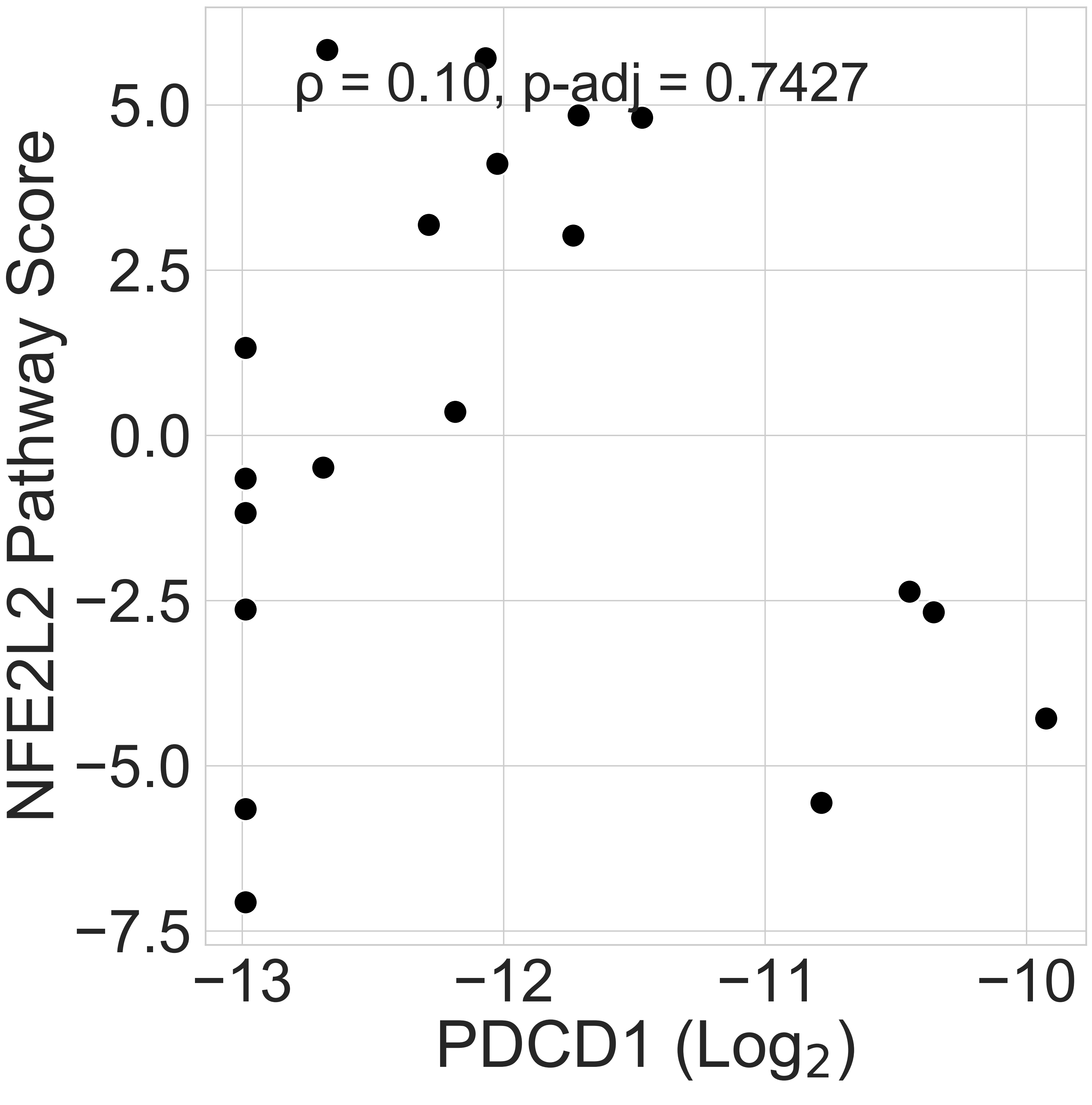

ρ = 0.10, p-adj = 0.7427
NFE2L2 Pathway Score
5.0
2.5
0.0
−2.5
−5.0
−7.5
−13
−12
−11
−10
PDCD1 (Log₂)

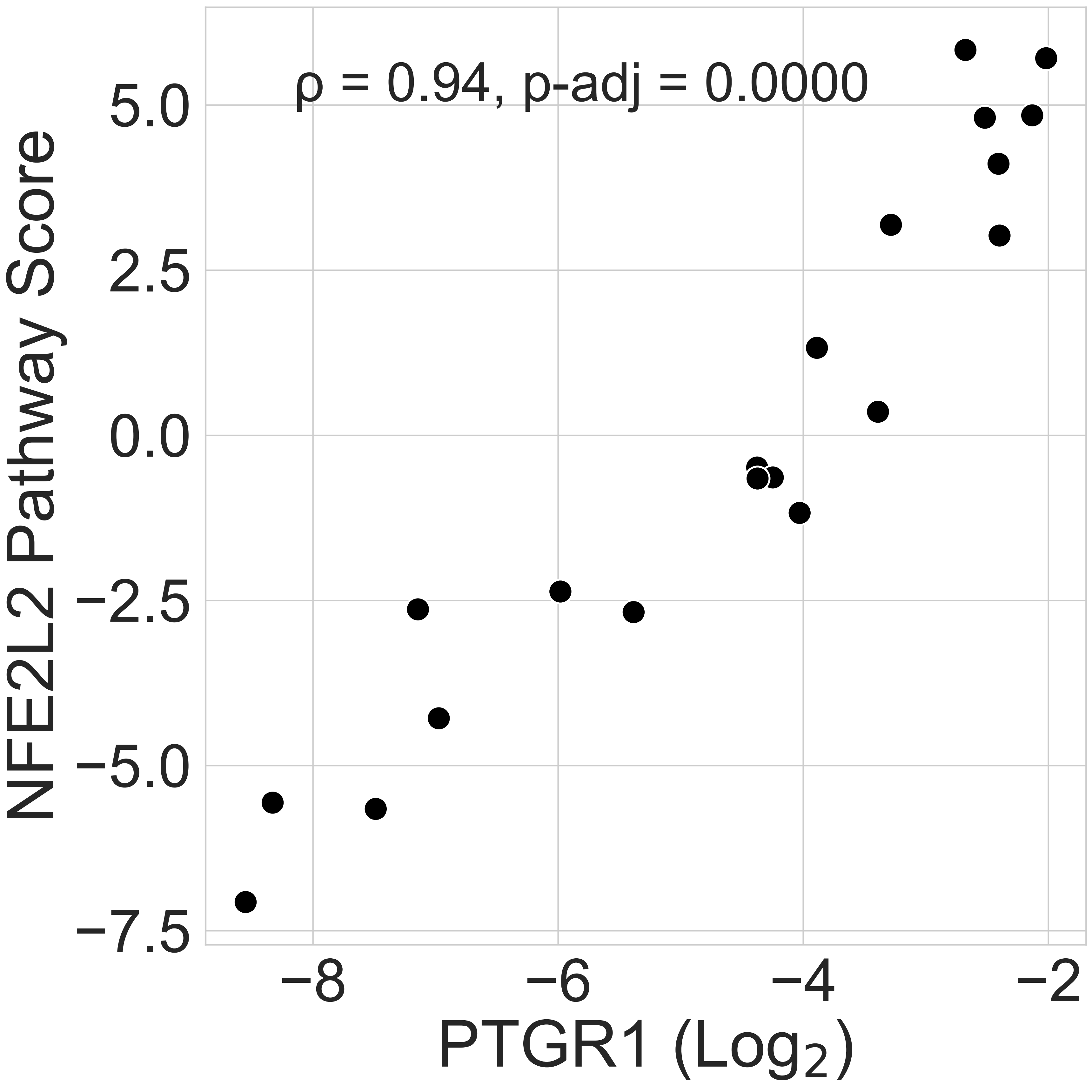
ρ = 0.94, p-adj = 0.0000
NFE2L2 Pathway Score
5.0
2.5
0.0
−2.5
−5.0
−7.5
−8
−6
−4
−2
PTGR1 (Log2)

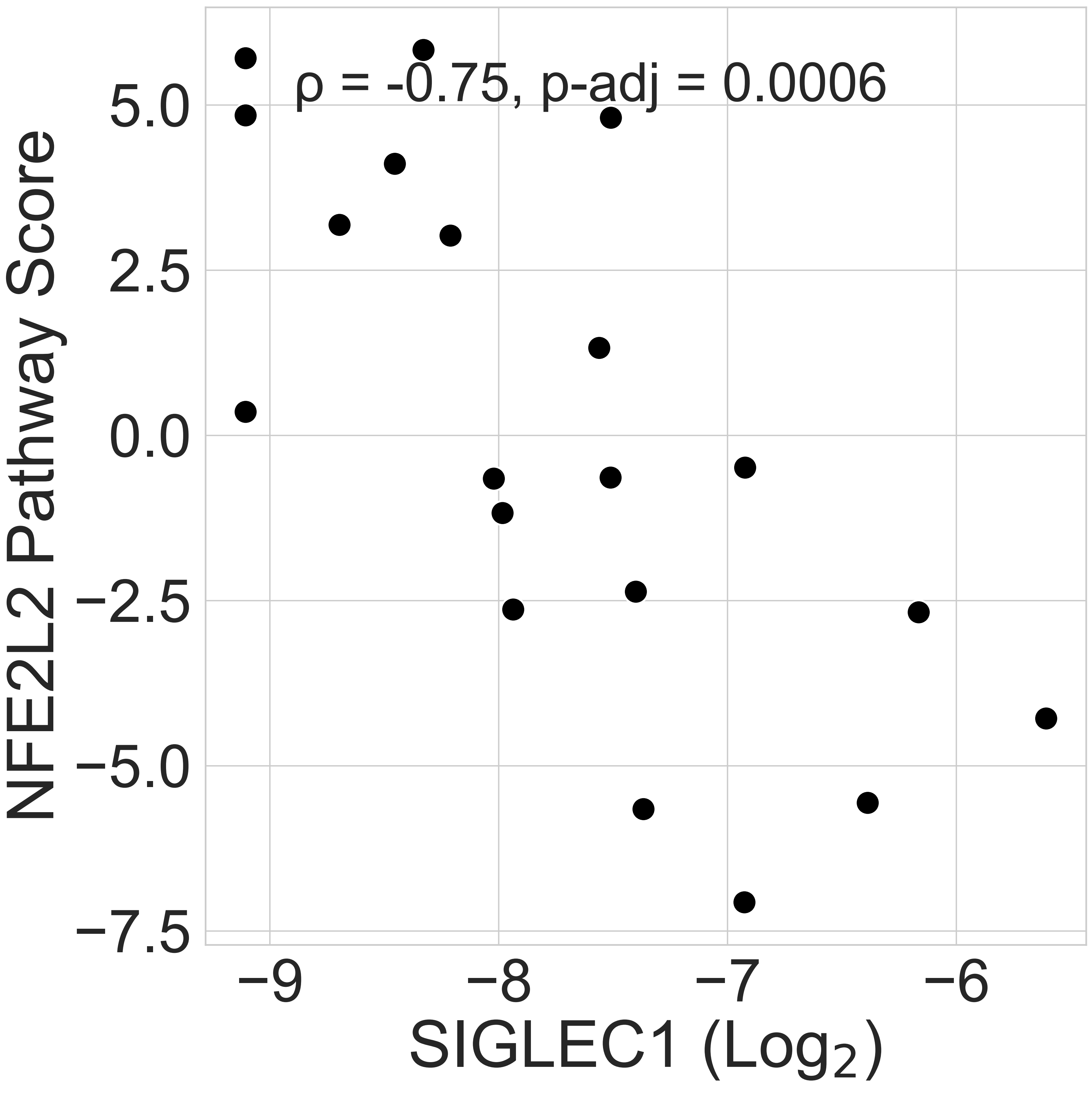
ρ = -0.75, p-adj = 0.0006
NFE2L2 Pathway Score
5.0
2.5
0.0
−2.5
−5.0
−7.5
−9
−8
−7
−6
SIGLEC1 (Log₂)

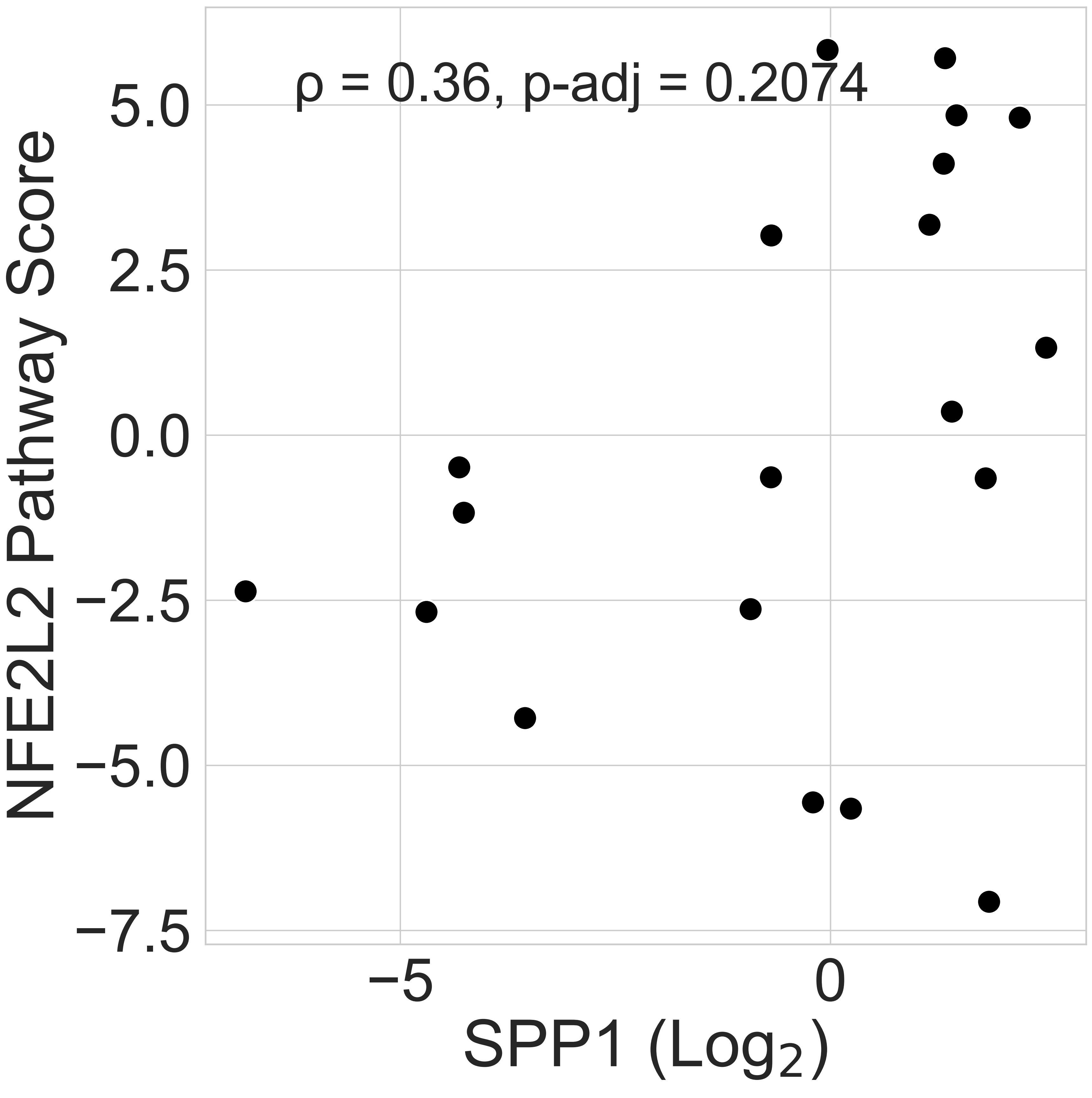

ρ = 0.36, p-adj = 0.2074
NFE2L2 Pathway Score
5.0
2.5
0.0
−2.5
−5.0
−7.5
−5
0
SPP1 (Log2)

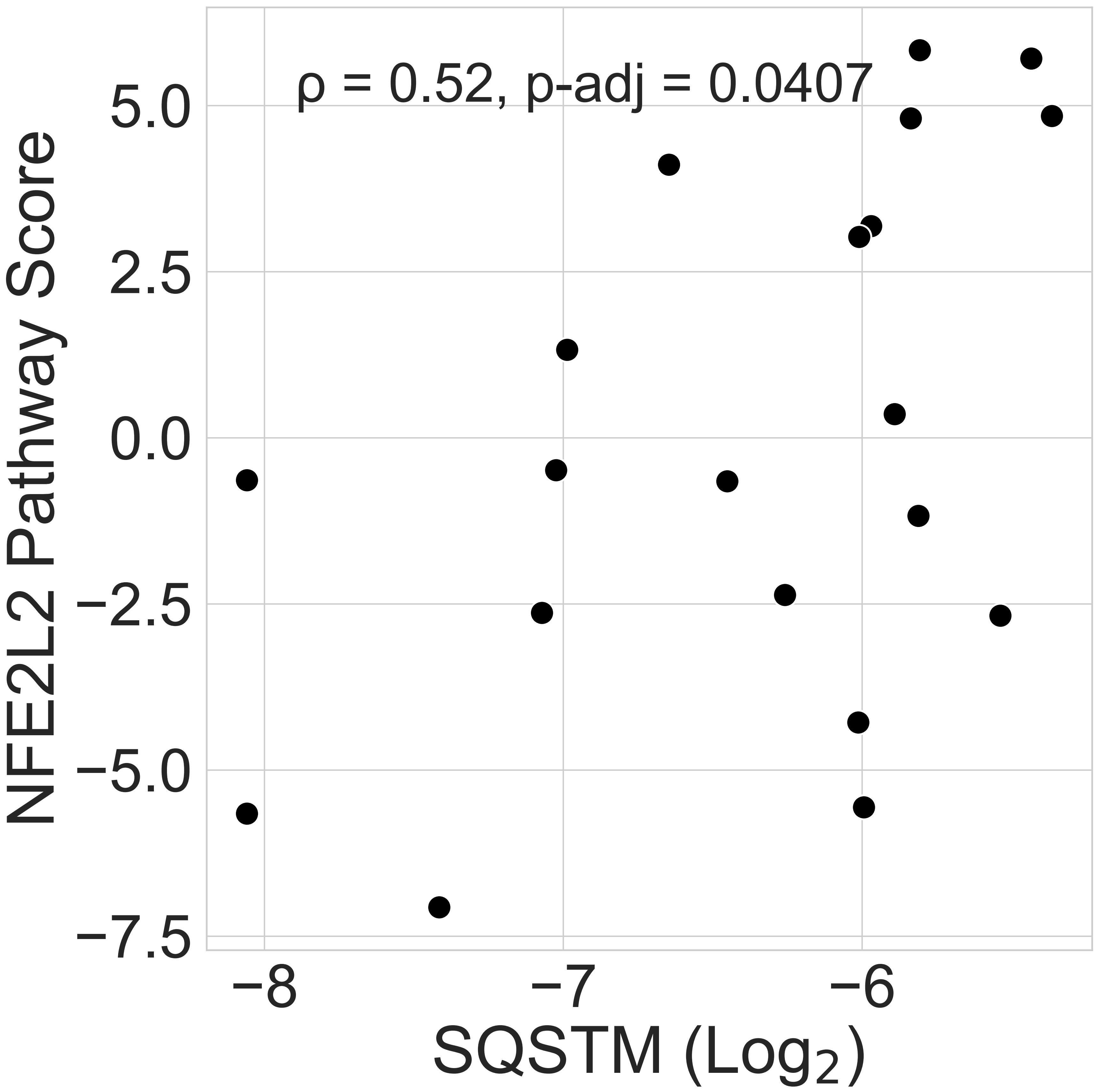
ρ = 0.52, p-adj = 0.0407
NFE2L2 Pathway Score
5.0
2.5
0.0
−2.5
−5.0
−7.5
−8
−7
−6
SQSTM (Log2)

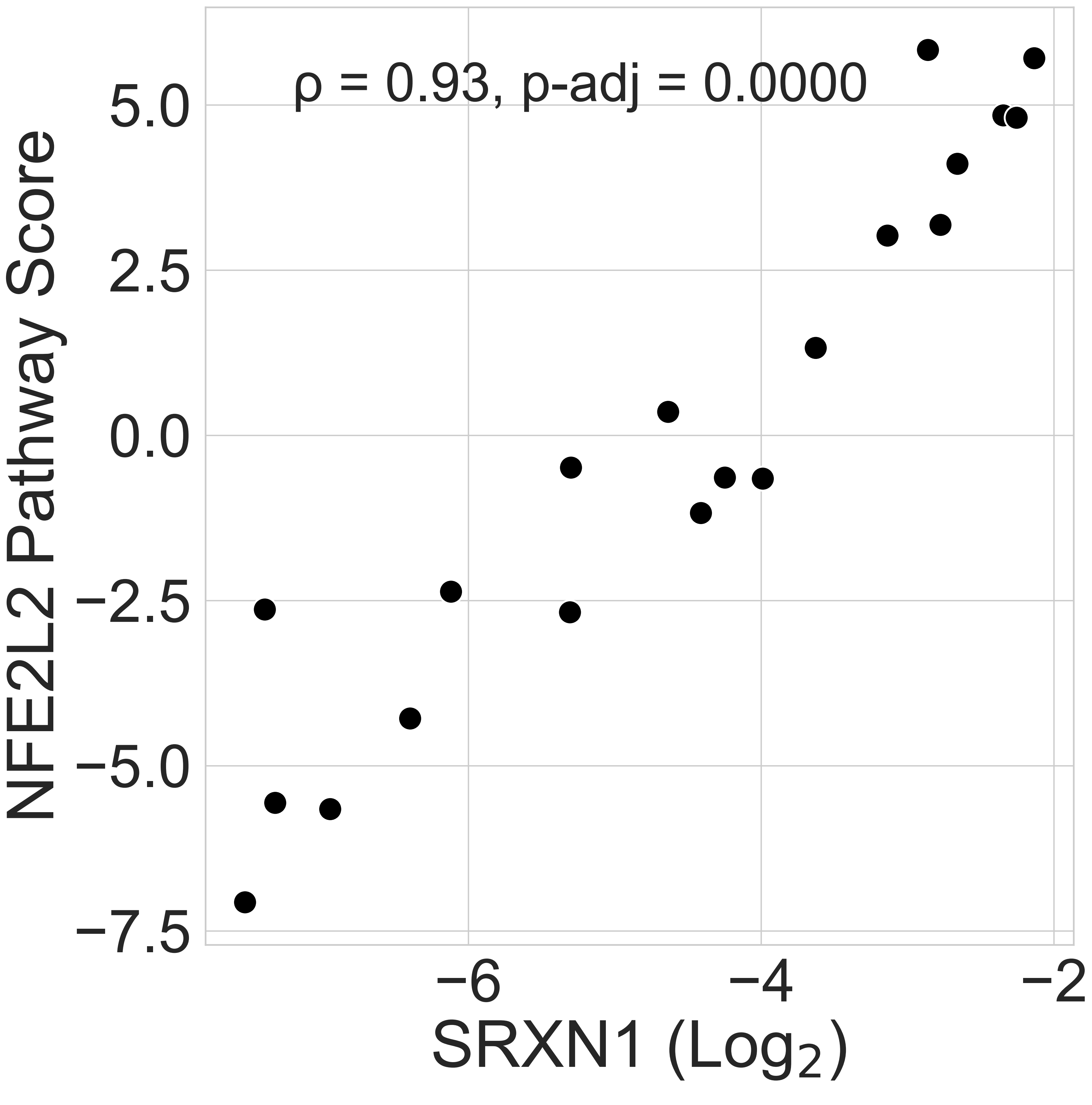

ρ = 0.93, p-adj = 0.0000
NFE2L2 Pathway Score
5.0
2.5
0.0
−2.5
−5.0
−7.5
−6
−4
−2
SRXN1 (Log₂)

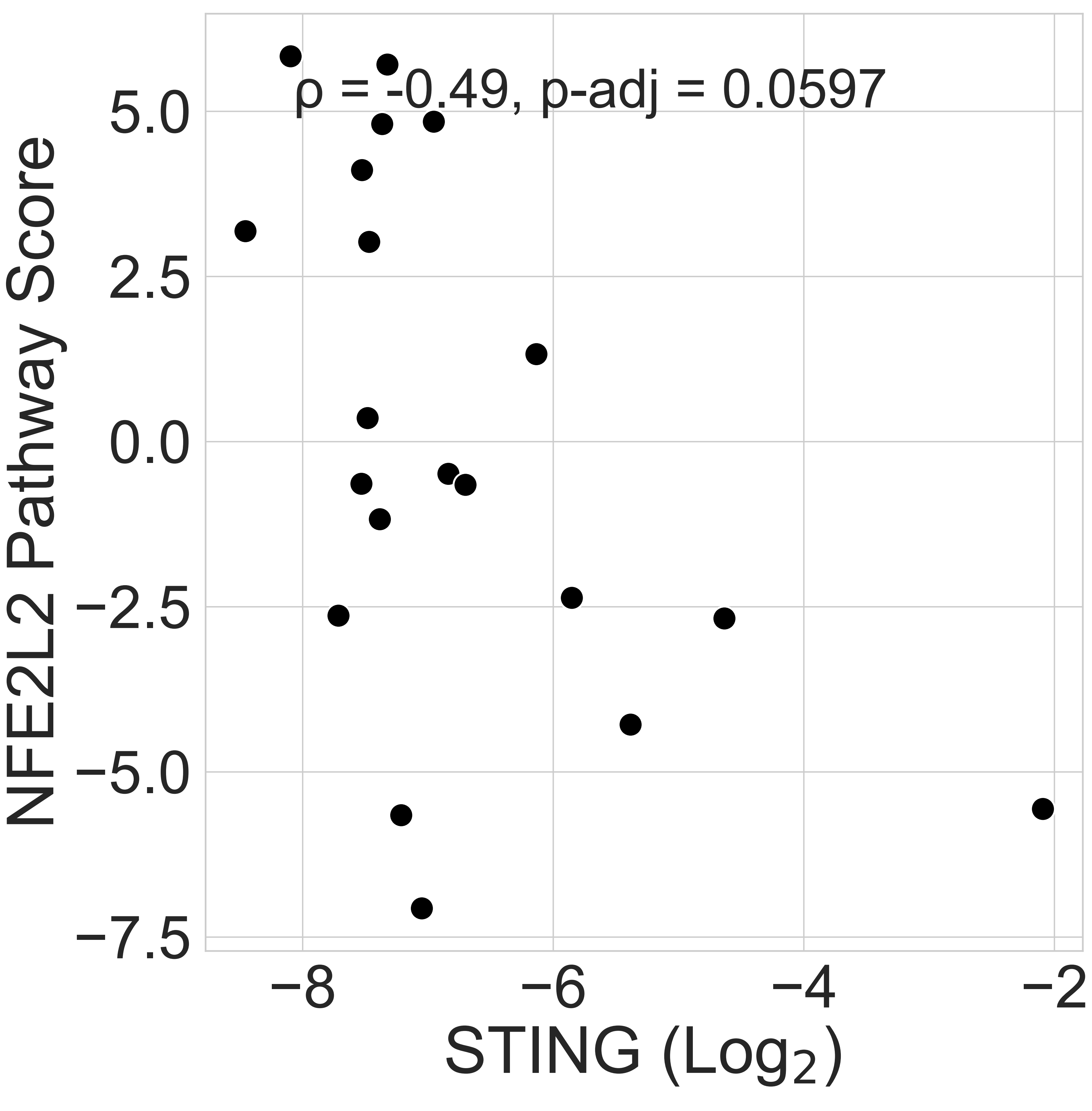
ρ = -0.49, p-adj = 0.0597
NFE2L2 Pathway Score
5.0
2.5
0.0
−2.5
−5.0
−7.5
−8
−6
−4
−2
STING (Log2)

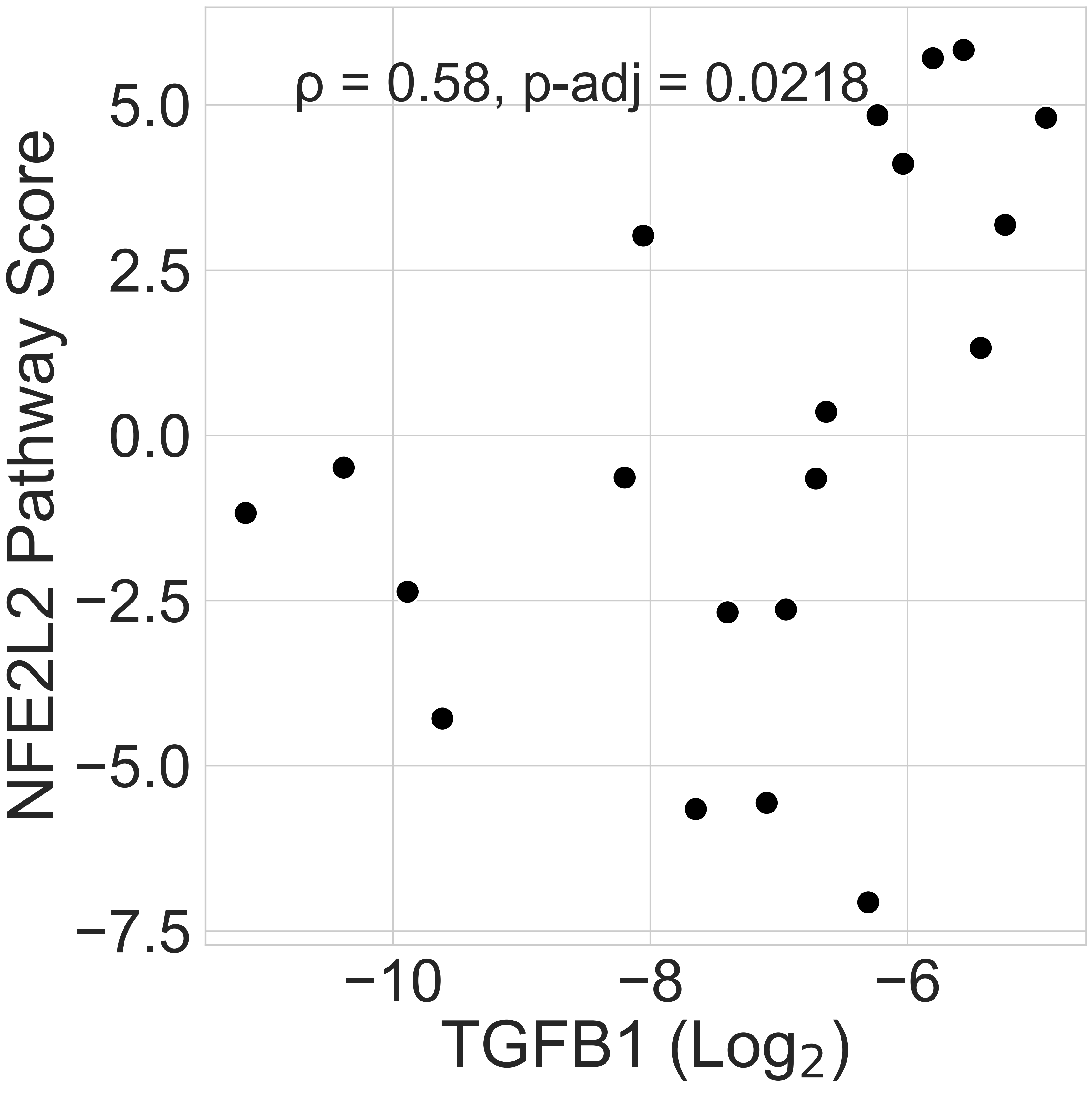

ρ = 0.58, p-adj = 0.0218
NFE2L2 Pathway Score
5.0
2.5
0.0
−2.5
−5.0
−7.5
−10
−8
−6
TGFB1 (Log2)

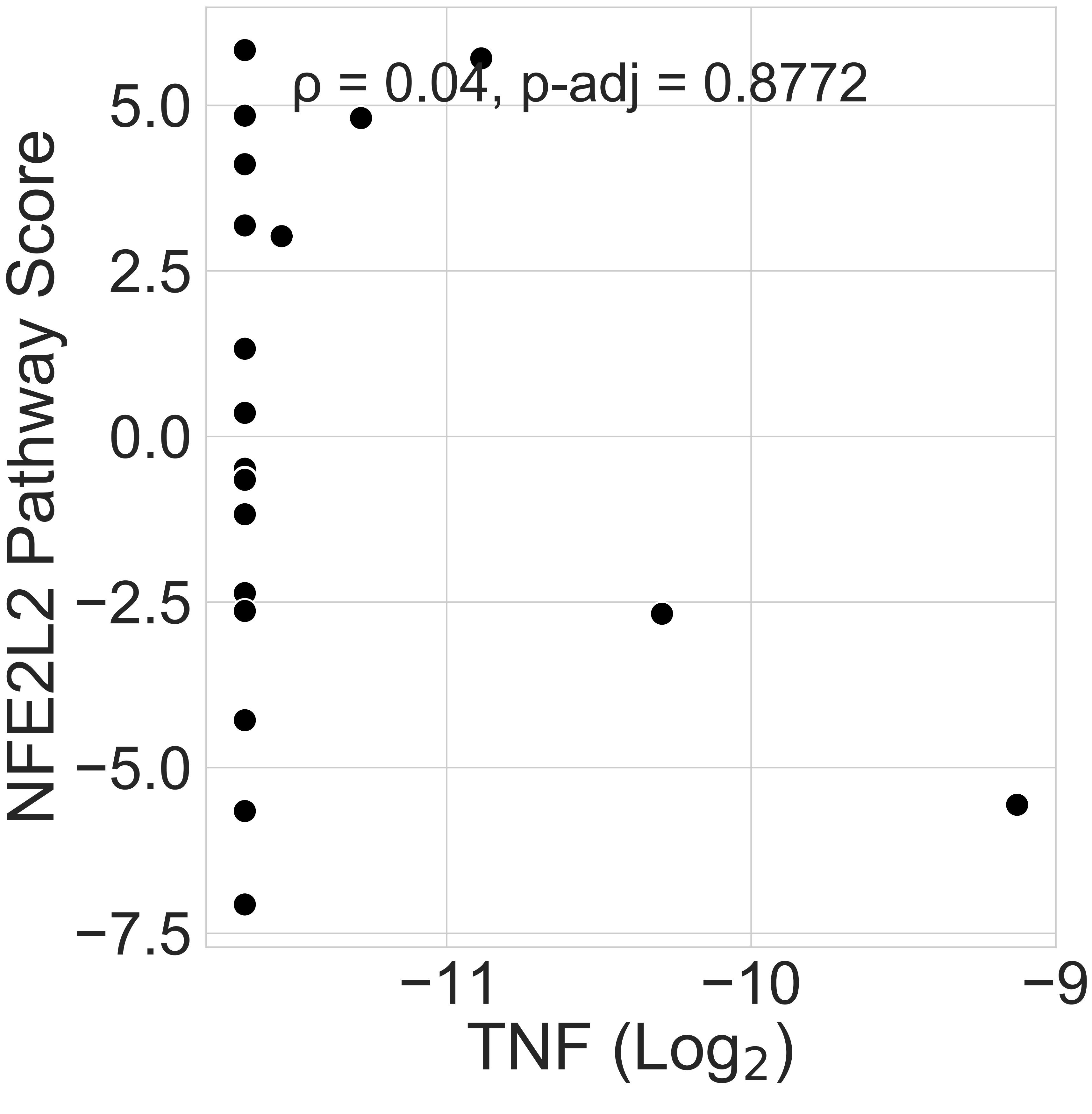

ρ = 0.04, p-adj = 0.8772
5.0
2.5
0.0
−2.5
−5.0
−7.5
NFE2L2 Pathway Score
−11
−10
−9
TNF (Log2)

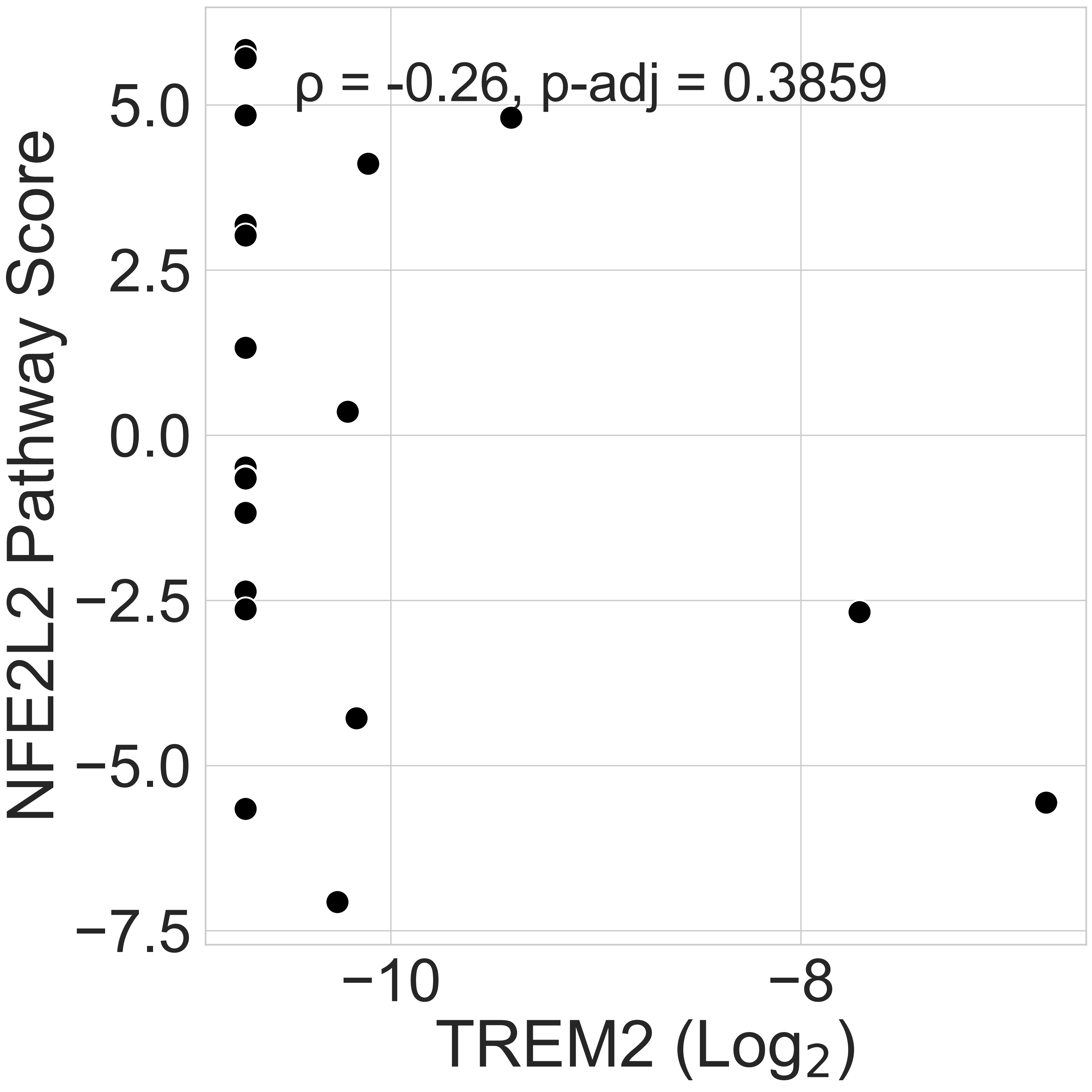

ρ = -0.26, p-adj = 0.3859
NFE2L2 Pathway Score
5.0
2.5
0.0
−2.5
−5.0
−7.5
−10
−8
TREM2 (Log₂)

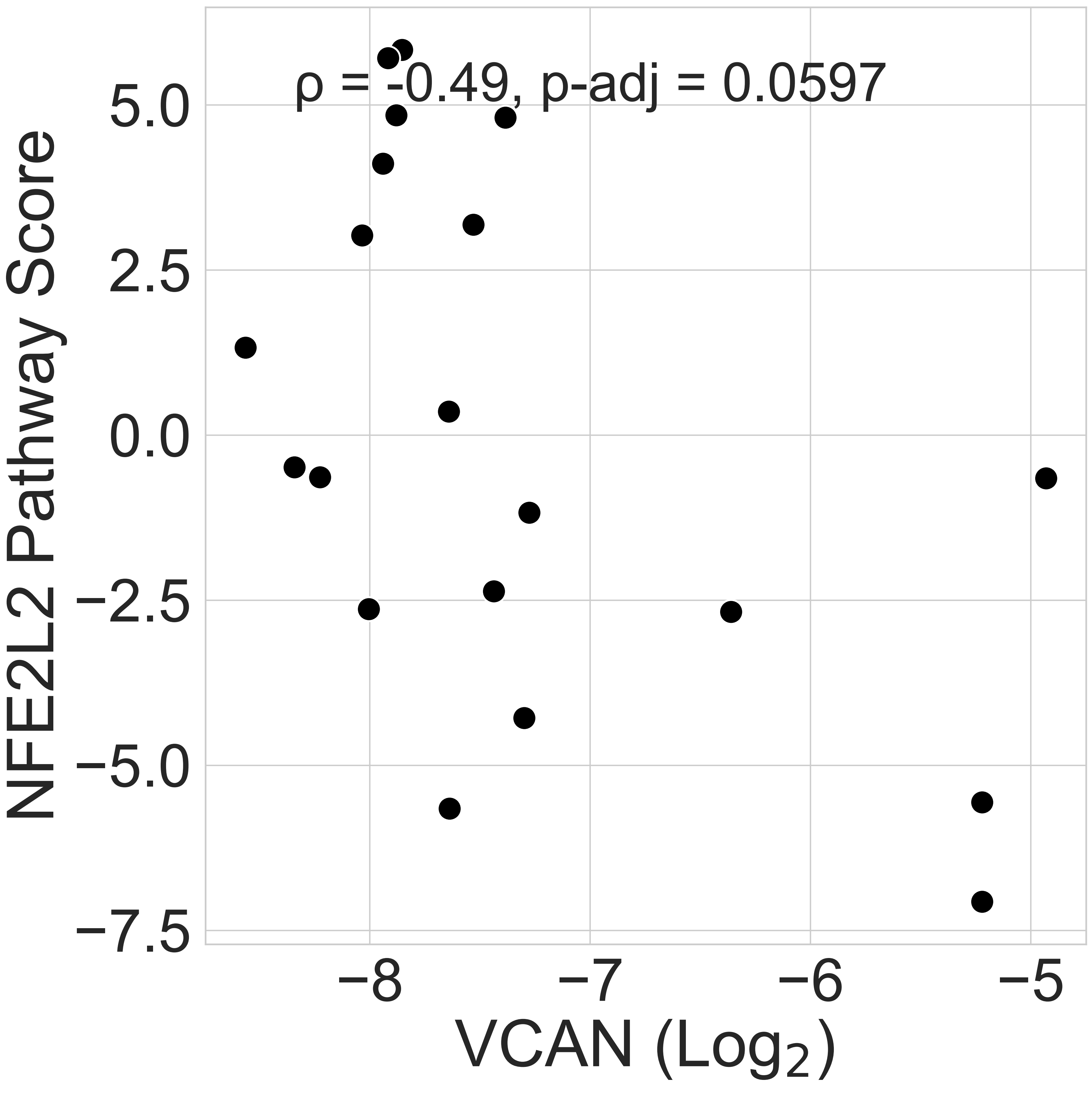
ρ = -0.49, p-adj = 0.0597
NFE2L2 Pathway Score
5.0
2.5
0.0
−2.5
−5.0
−7.5
−8
−7
−6
−5
VCAN (Log₂)

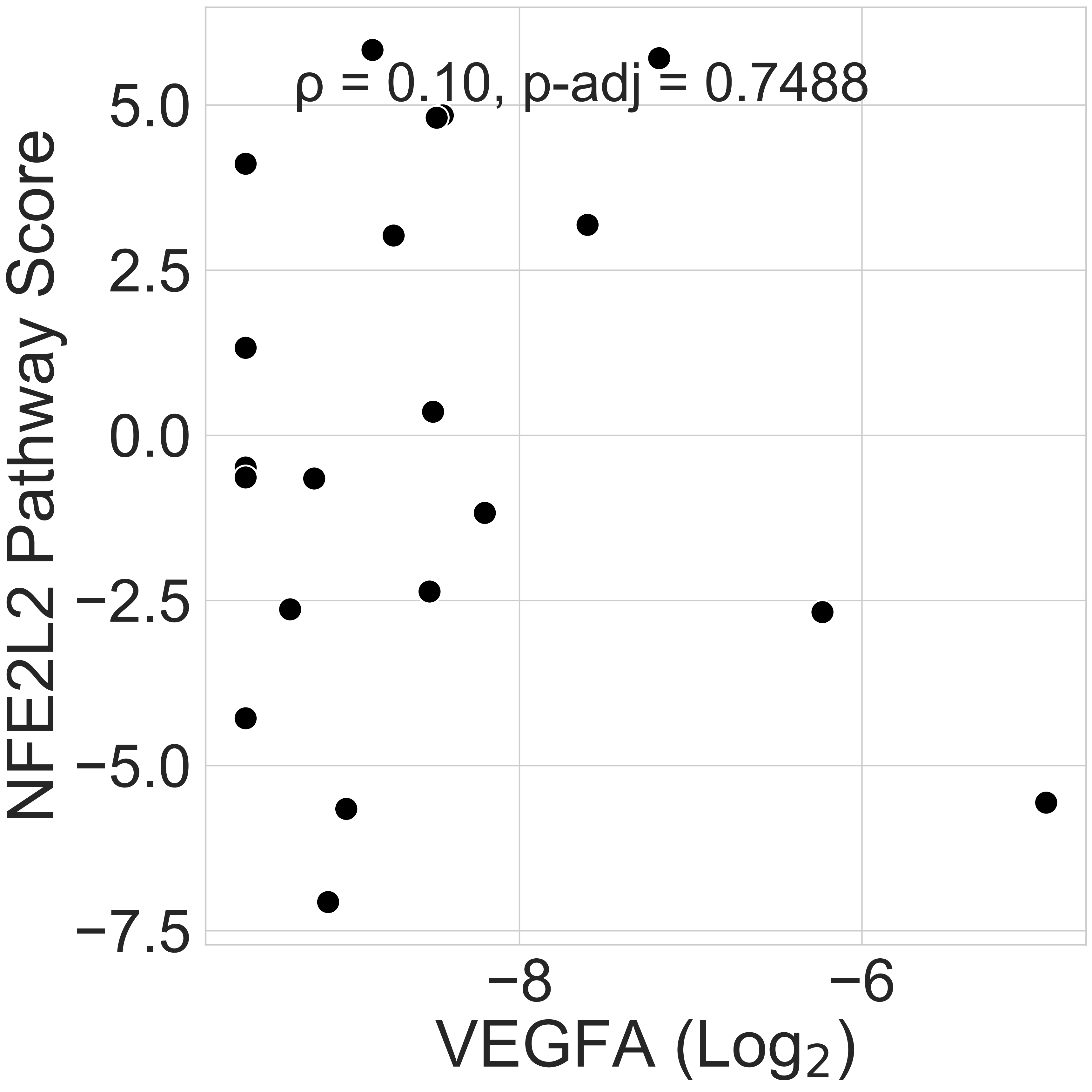
ρ = 0.10, p-adj = 0.7488
NFE2L2 Pathway Score
5.0
2.5
0.0
−2.5
−5.0
−7.5
−8
−6
VEGFA (Log2)

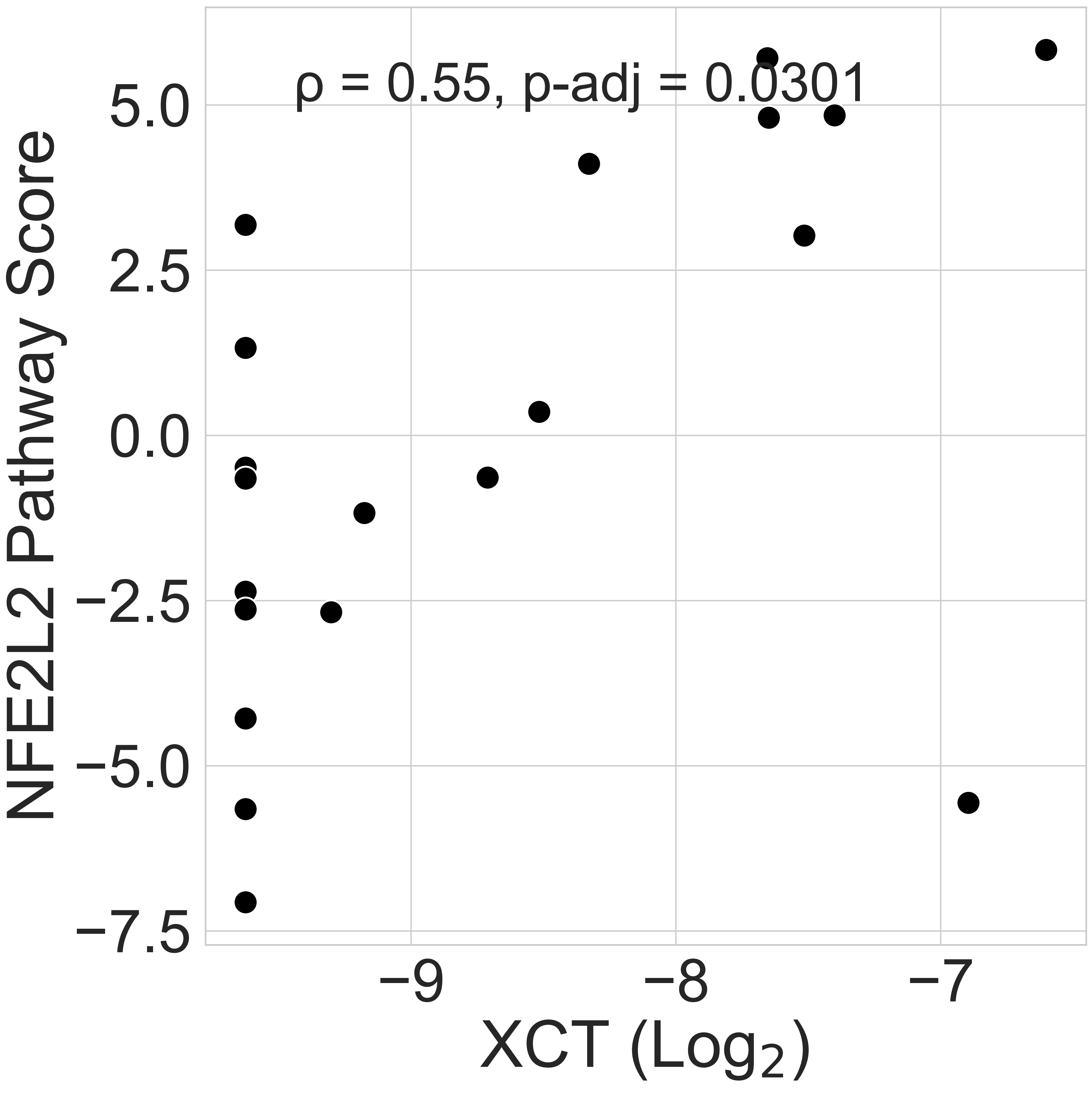
ρ = 0.55, p-adj = 0.0301
5.0
2.5
0.0
−2.5
−5.0
−7.5
NFE2L2 Pathway Score
−9
−8
−7
XCT (Log₂)
