# Supplementary material for: NRF2 Activation in Trp53;p16-deficient Mice Drives Oral Squamous Cell Carcinoma
Source: Cancer Res Commun. 2024 Feb 21;4(2):487–95. doi: 10.1158/2767-9764.CRC-23-0386 (PMC10880604; doi:10.1158/2767-9764.CRC-23-0386)
Supplement: Figure S5 — shows box-and-whisker plots of protein abundance as measured by targeted protein mass spectrometry CP and CPN oral cavity tissues. [file crc-23-0386-s05.pdf]

Figure S5. Box-and-whisker plots of protein abundance in CP and CPN oral cavity tissues. Box-and-whisker plots of protein abundance as measured by targeted protein mass spectrometry were generated as described in the methods. P-values were calculated with a Mann Whitney U-test and adjusted by a Benjamin Hochberg procedure (34).

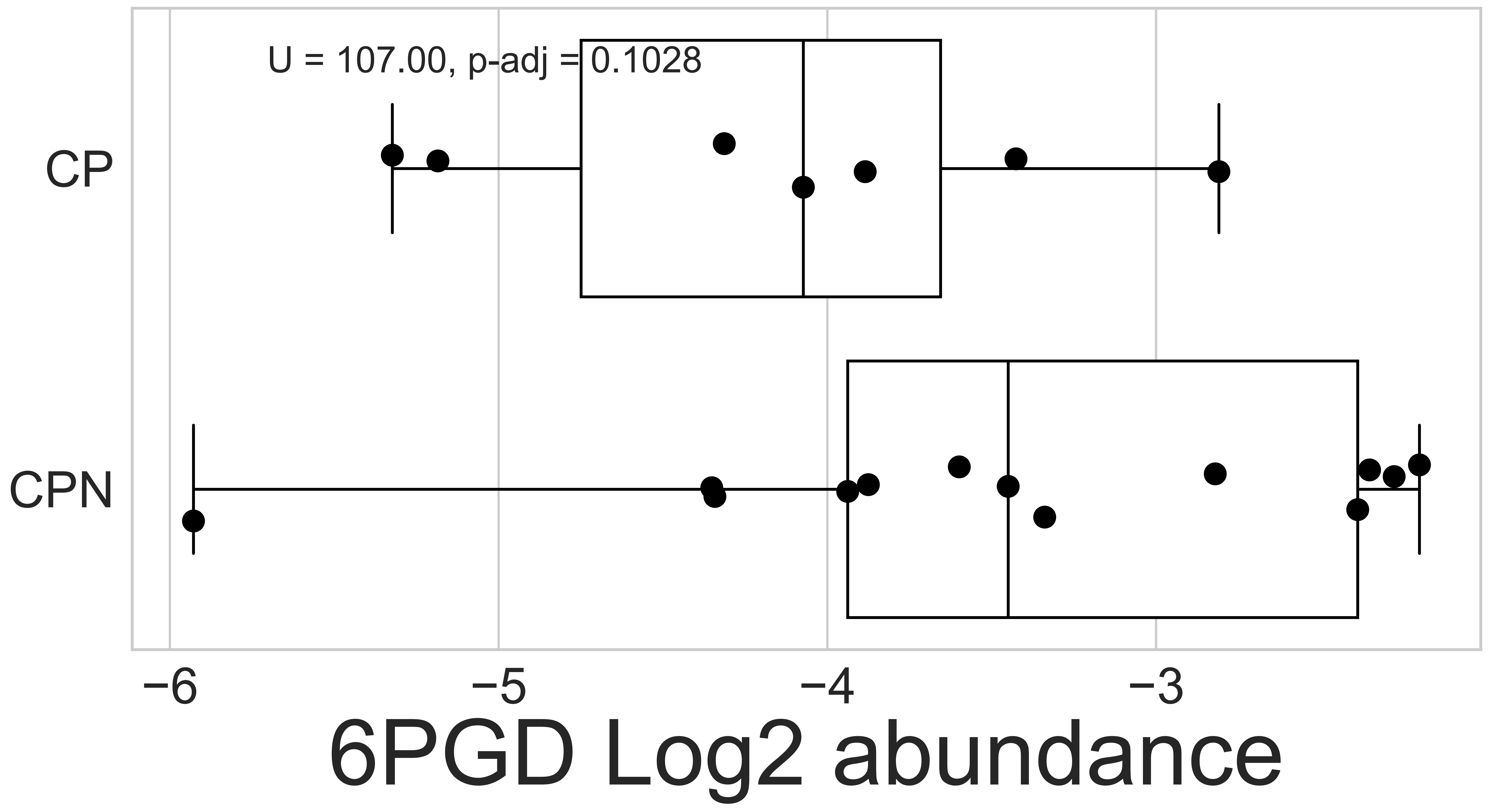
U = 107.00, p-adj = 0.1028
CP
CPN
−6
−5
−4
−3
6PGD Log2 abundance

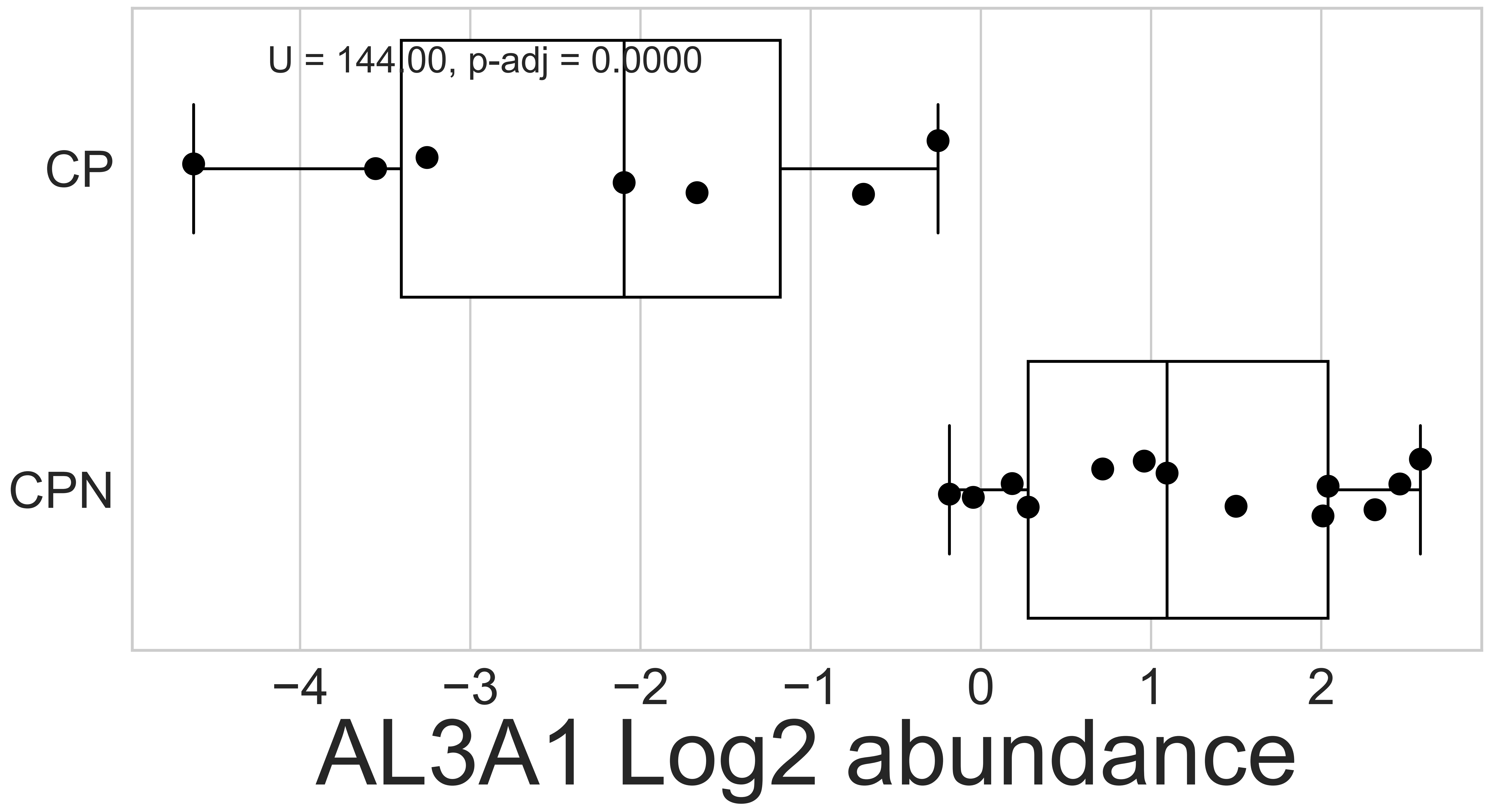
U = 144.00, p-adj = 0.0000
CP
CPN
−4
−3
−2
−1
0
1
2
AL3A1 Log2 abundance

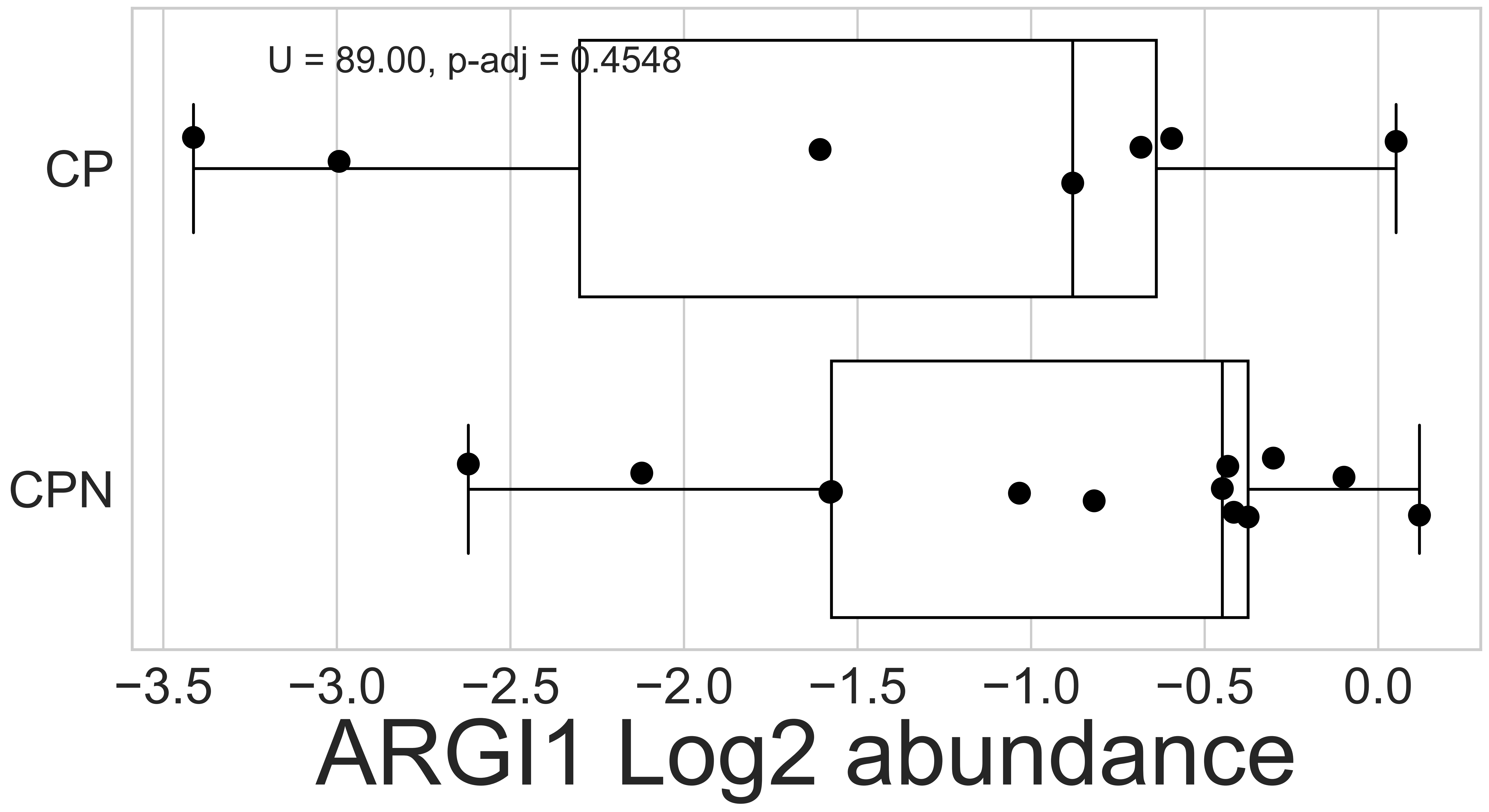
U = 89.00, p-adj = 0.4548
CP
CPN
−3.5
−3.0
−2.5
−2.0
−1.5
−1.0
−0.5
0.0
ARGI1 Log2 abundance

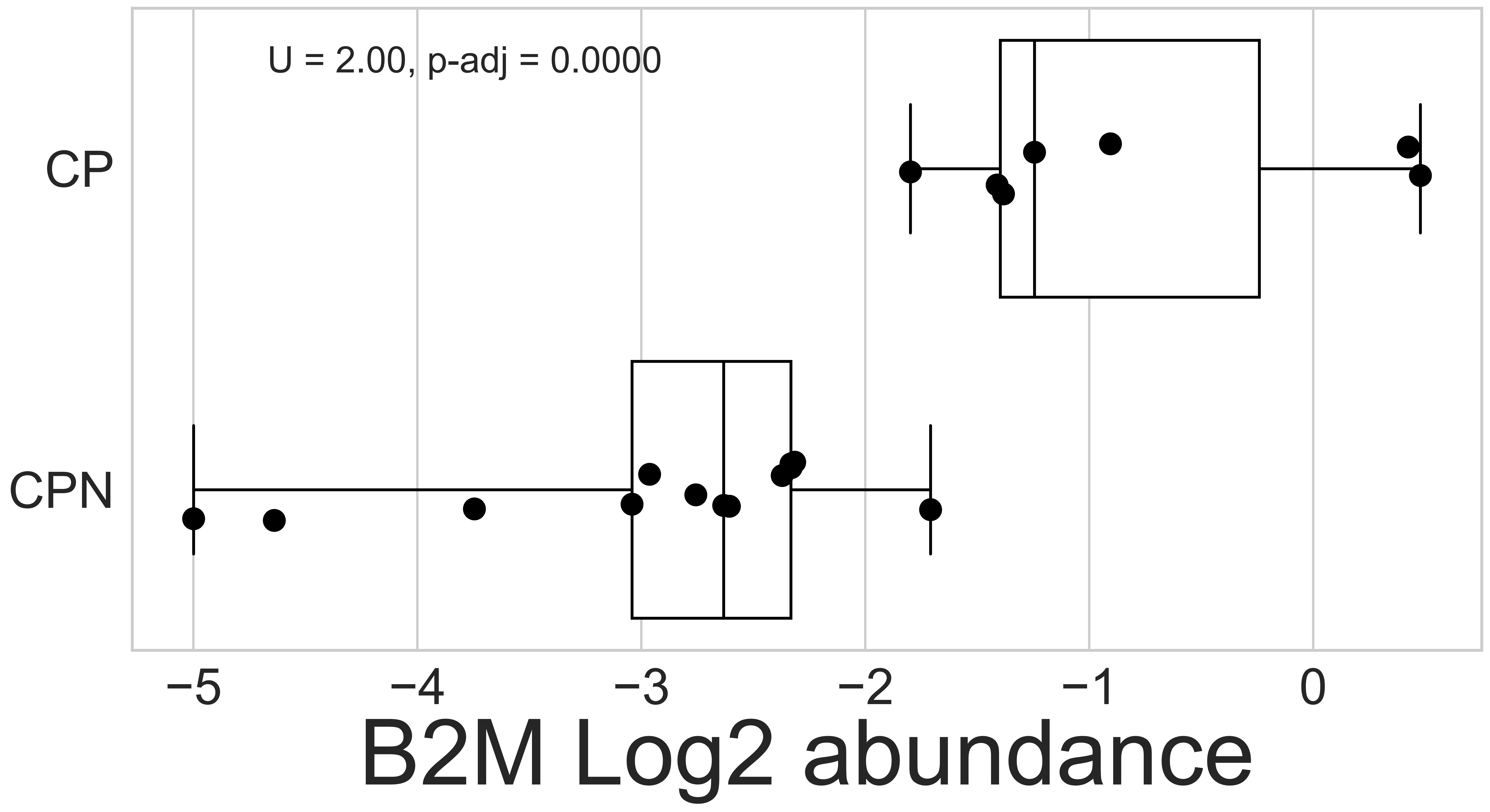
U = 2.00, p-adj = 0.0000
CP
CPN
−5
−4
−3
−2
−1
0
B2M Log2 abundance

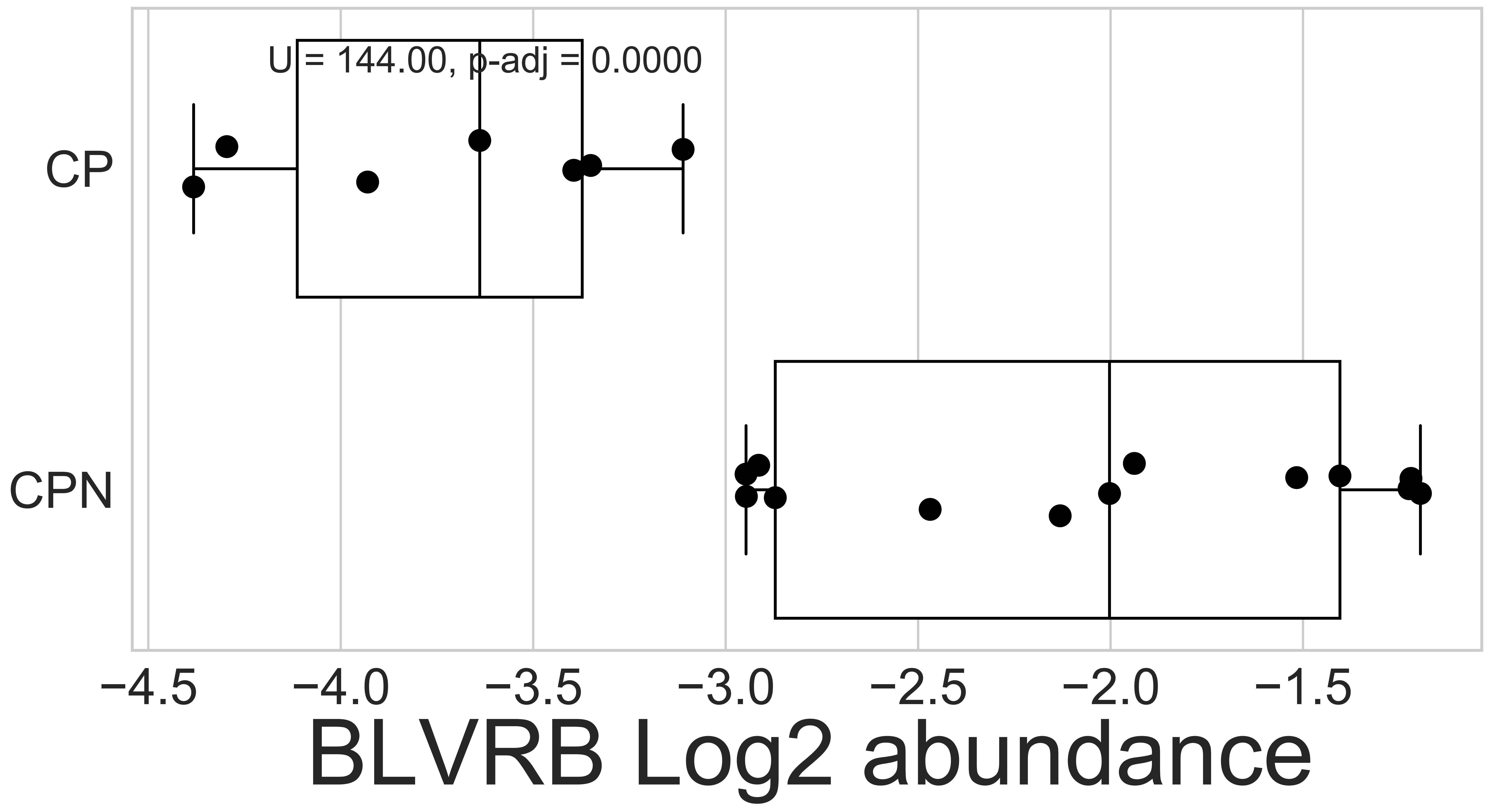
U = 144.00, p-adj = 0.0000
CP
CPN
−4.5
−4.0
−3.5
−3.0
−2.5
−2.0
−1.5
BLVRB Log2 abundance

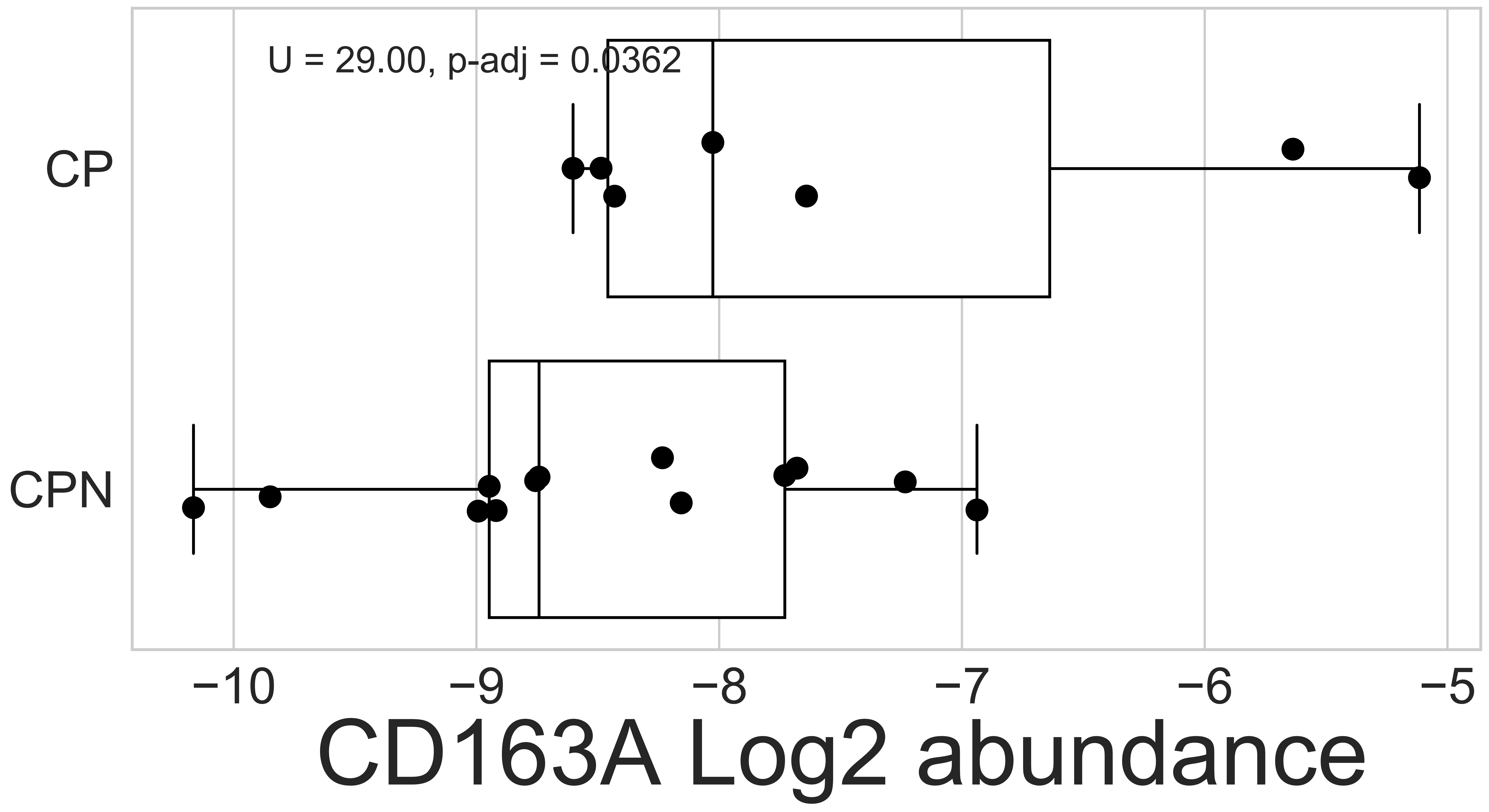
U = 29.00, p-adj = 0.0362
CP
CPN
−10
−9
−8
−7
−6
−5
CD163A Log2 abundance

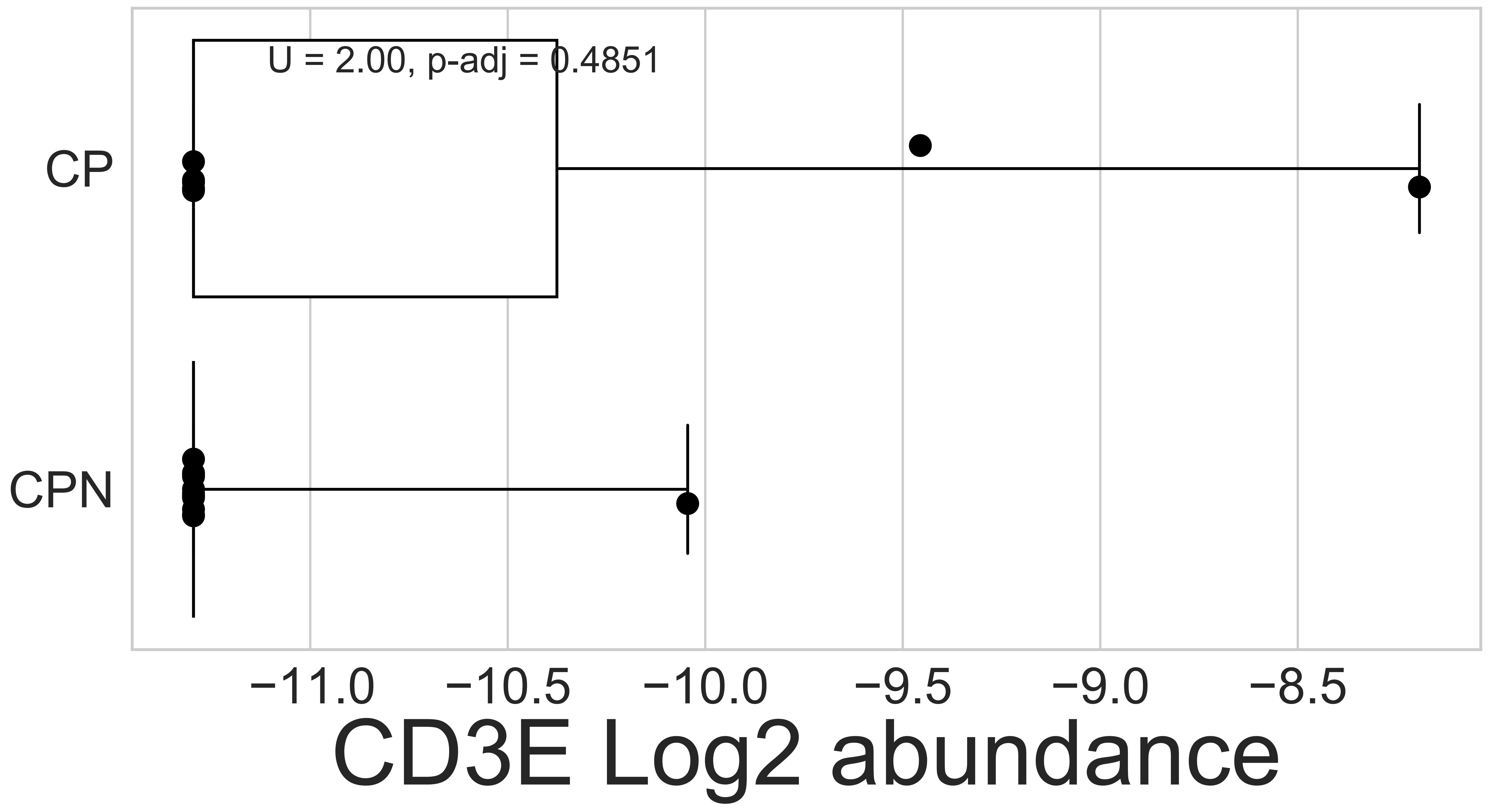
U = 2.00, p-adj = 0.4851
CP
CPN
−11.0
−10.5
−10.0
−9.5
−9.0
−8.5
CD3E Log2 abundance

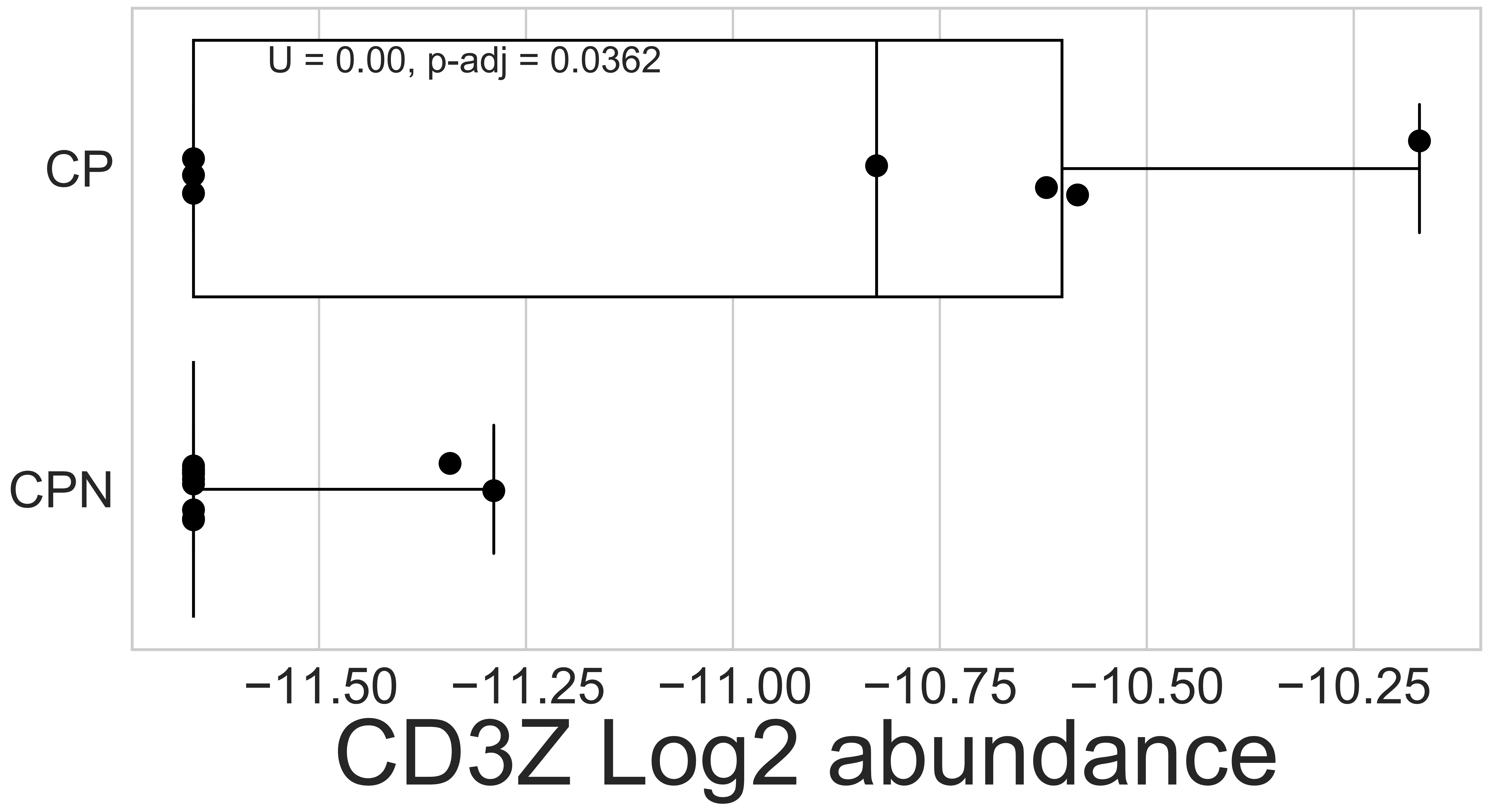
U = 0.00, p-adj = 0.0362
CP
CPN
−11.50
−11.25
−11.00
−10.75
−10.50
−10.25
CD3Z Log2 abundance

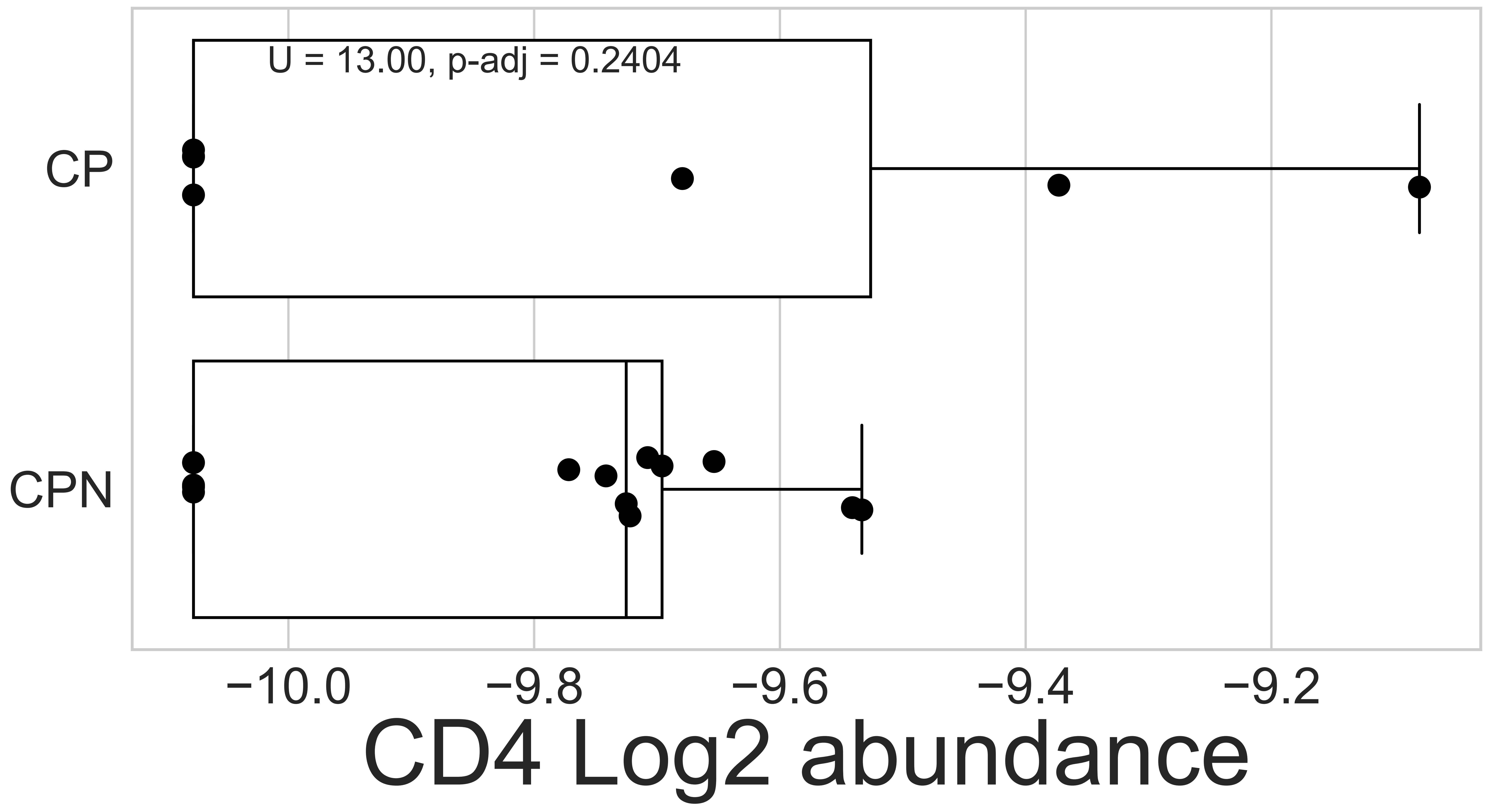
U = 13.00, p-adj = 0.2404
CP
CPN
−10.0
−9.8
−9.6
−9.4
−9.2
CD4 Log2 abundance

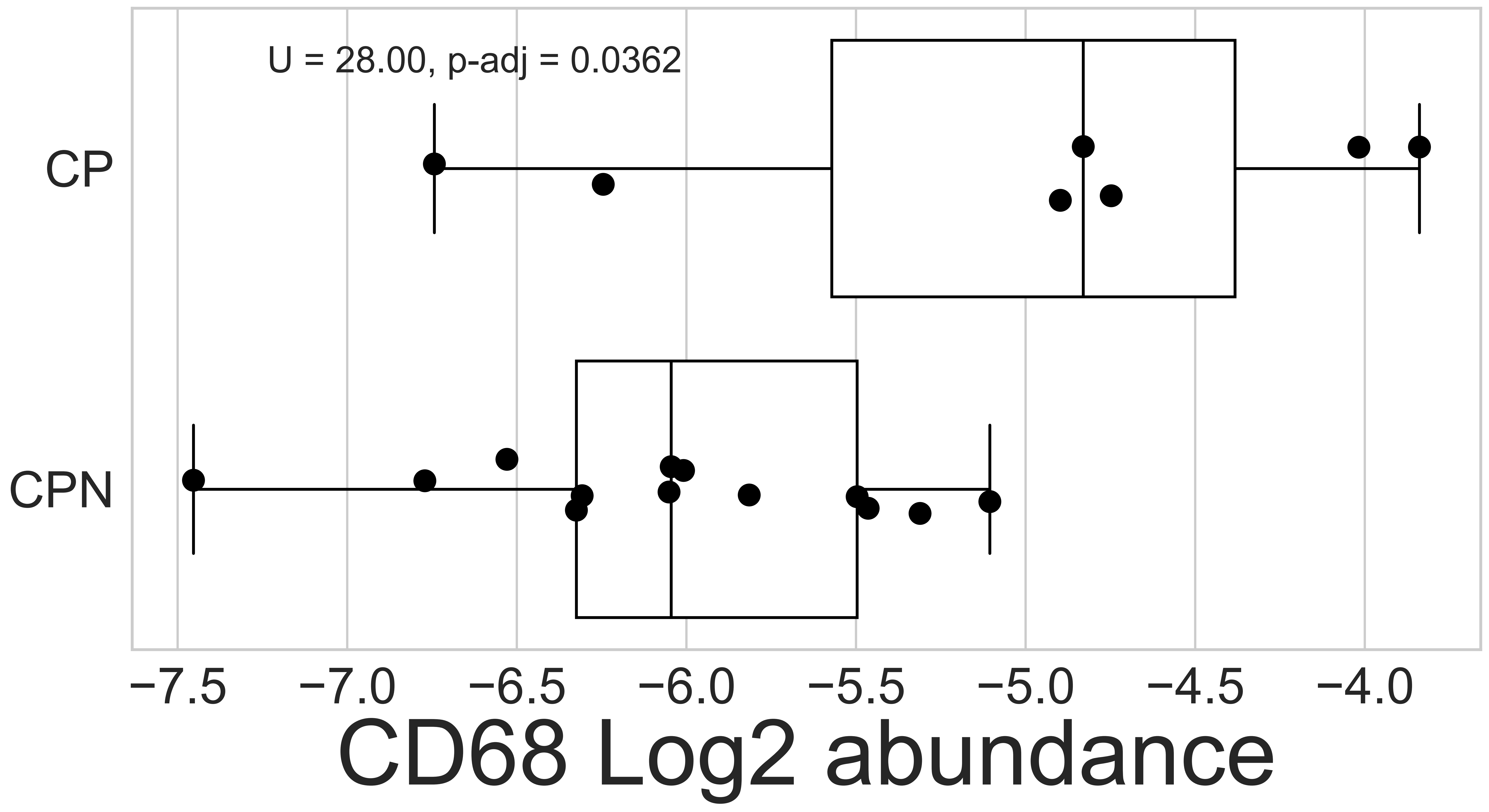

U = 28.00, p-adj = 0.0362
CP
CPN
−7.5
−7.0
−6.5
−6.0
−5.5
−5.0
−4.5
−4.0
CD68 Log2 abundance

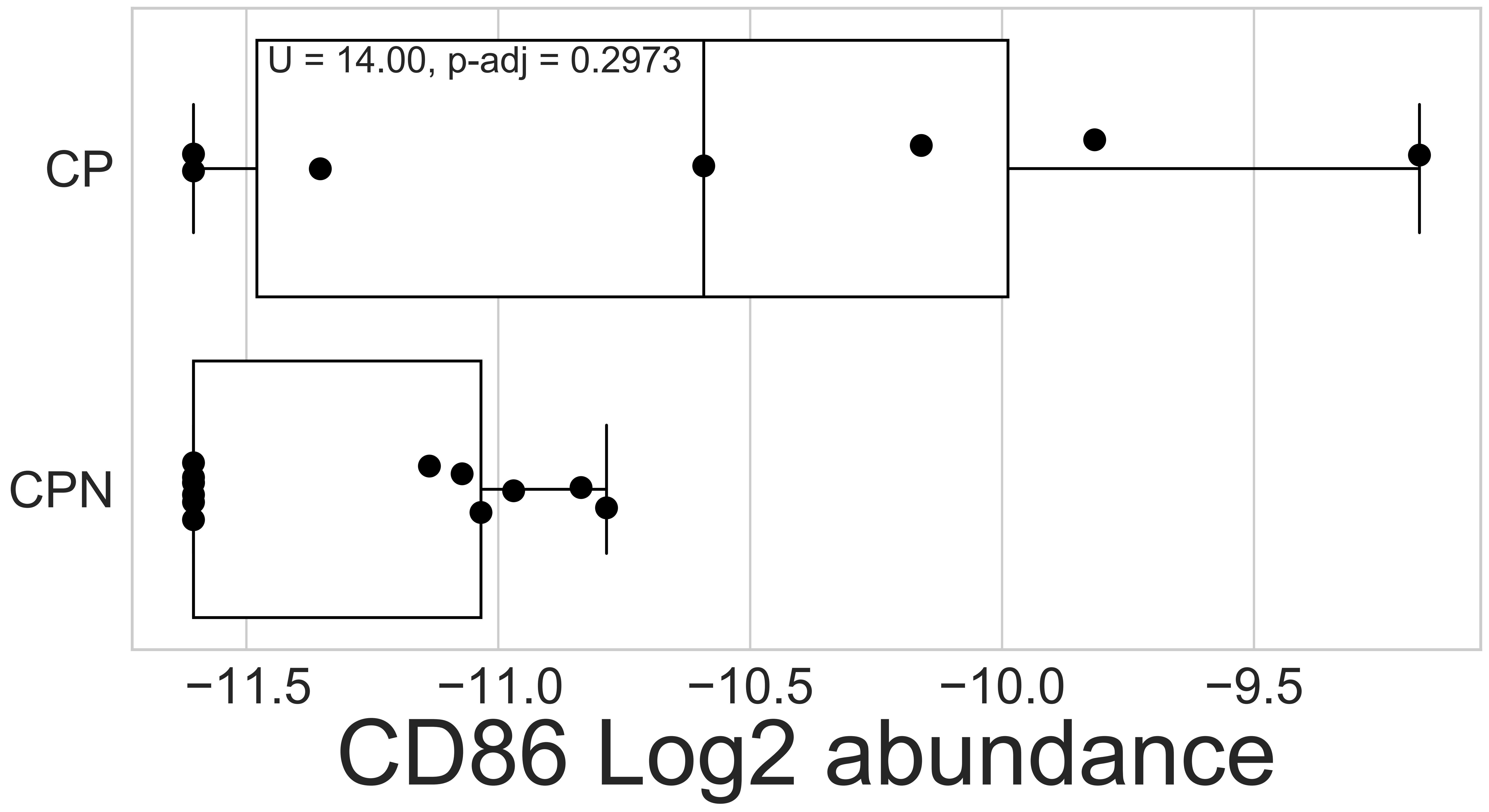
U = 14.00, p-adj = 0.2973
CP
CPN
−11.5
−11.0
−10.5
−10.0
−9.5
CD86 Log2 abundance

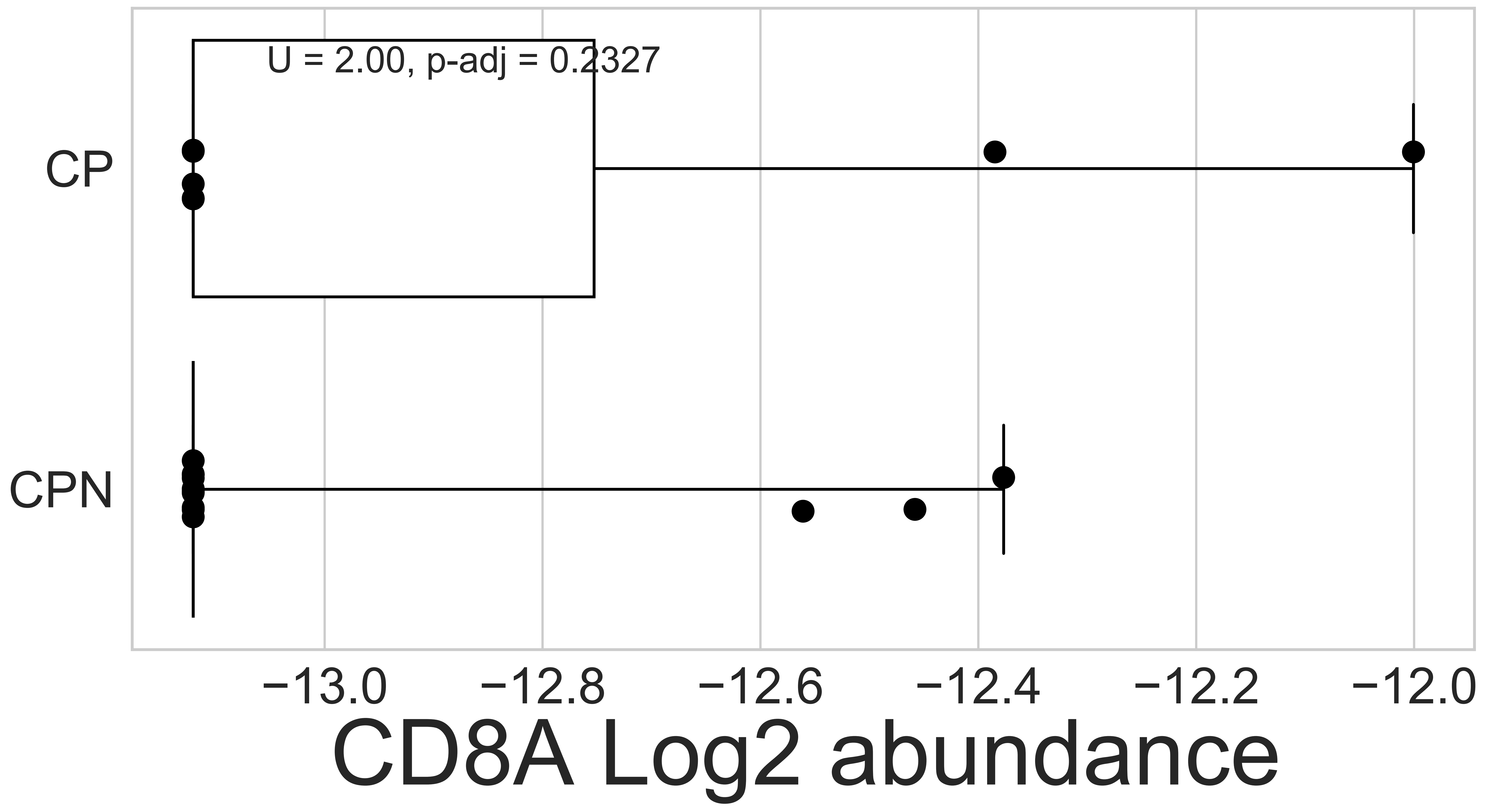

U = 2.00, p-adj = 0.2327
CP
CPN
−13.0
−12.8
−12.6
−12.4
−12.2
−12.0
CD8A Log2 abundance

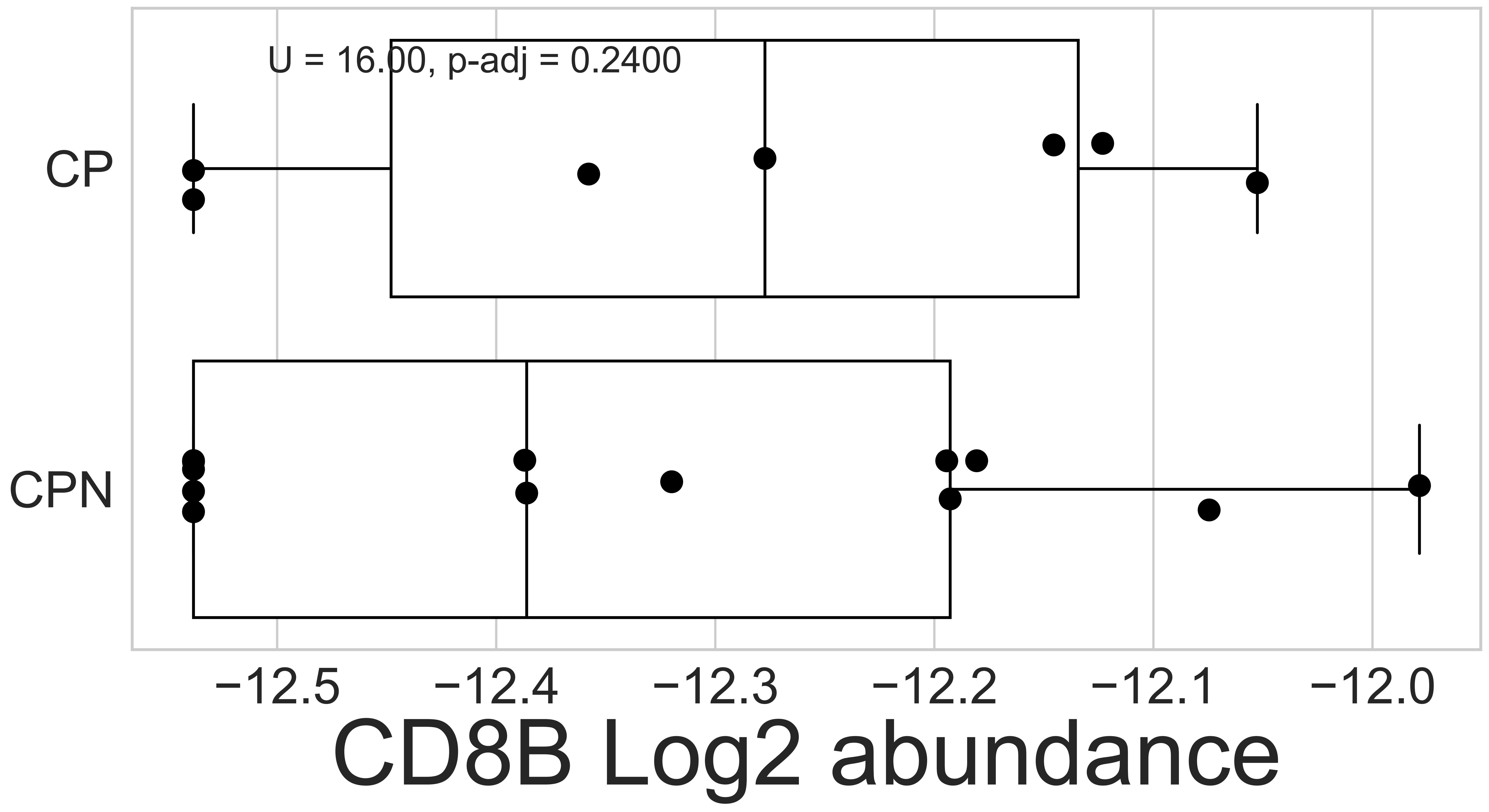
U = 16.00, p-adj = 0.2400
CP
CPN
−12.5
−12.4
−12.3
−12.2
−12.1
−12.0
CD8B Log2 abundance

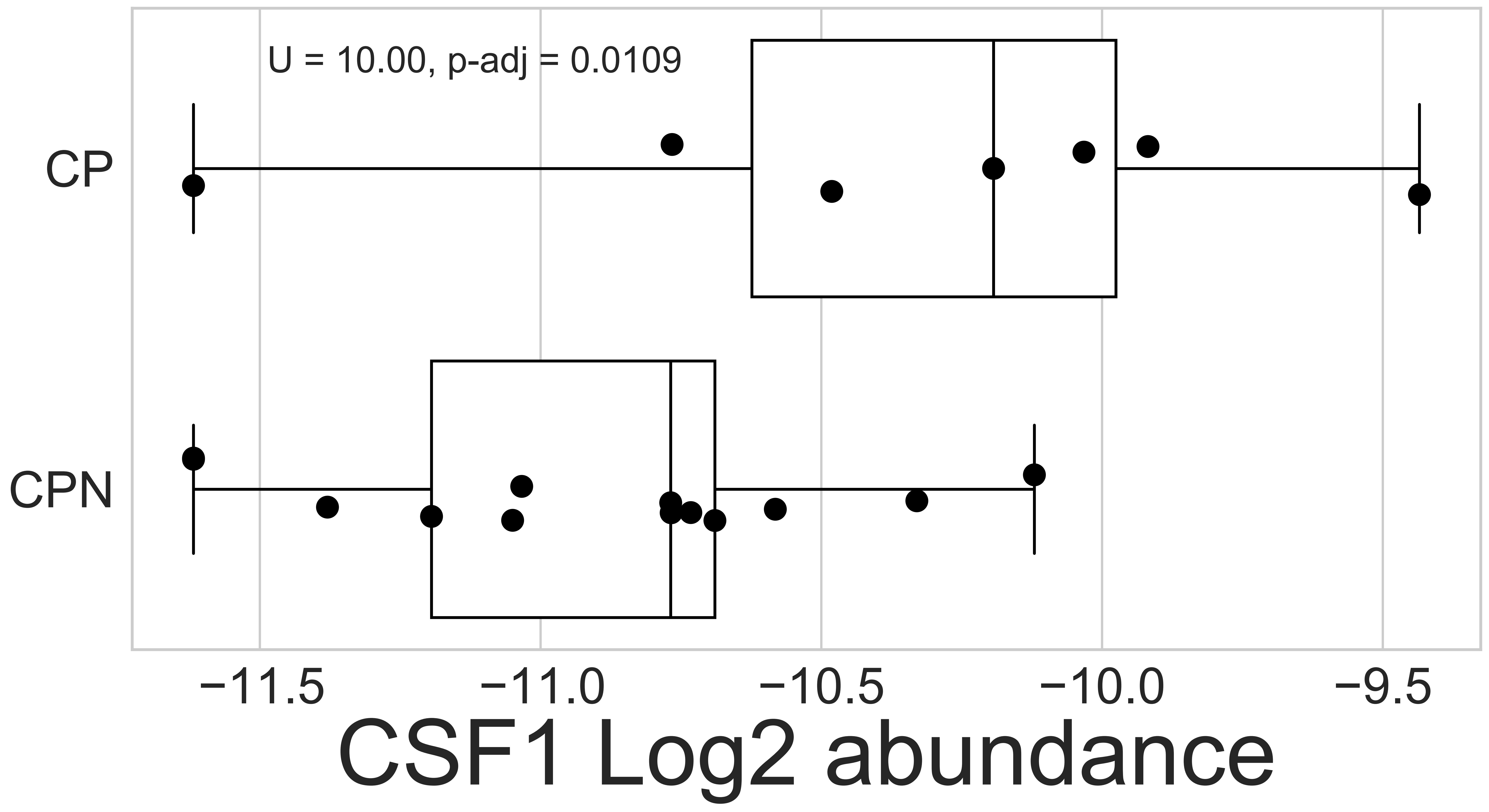

U = 10.00, p-adj = 0.0109
CP
CPN
−11.5
−11.0
−10.5
−10.0
−9.5
CSF1 Log2 abundance

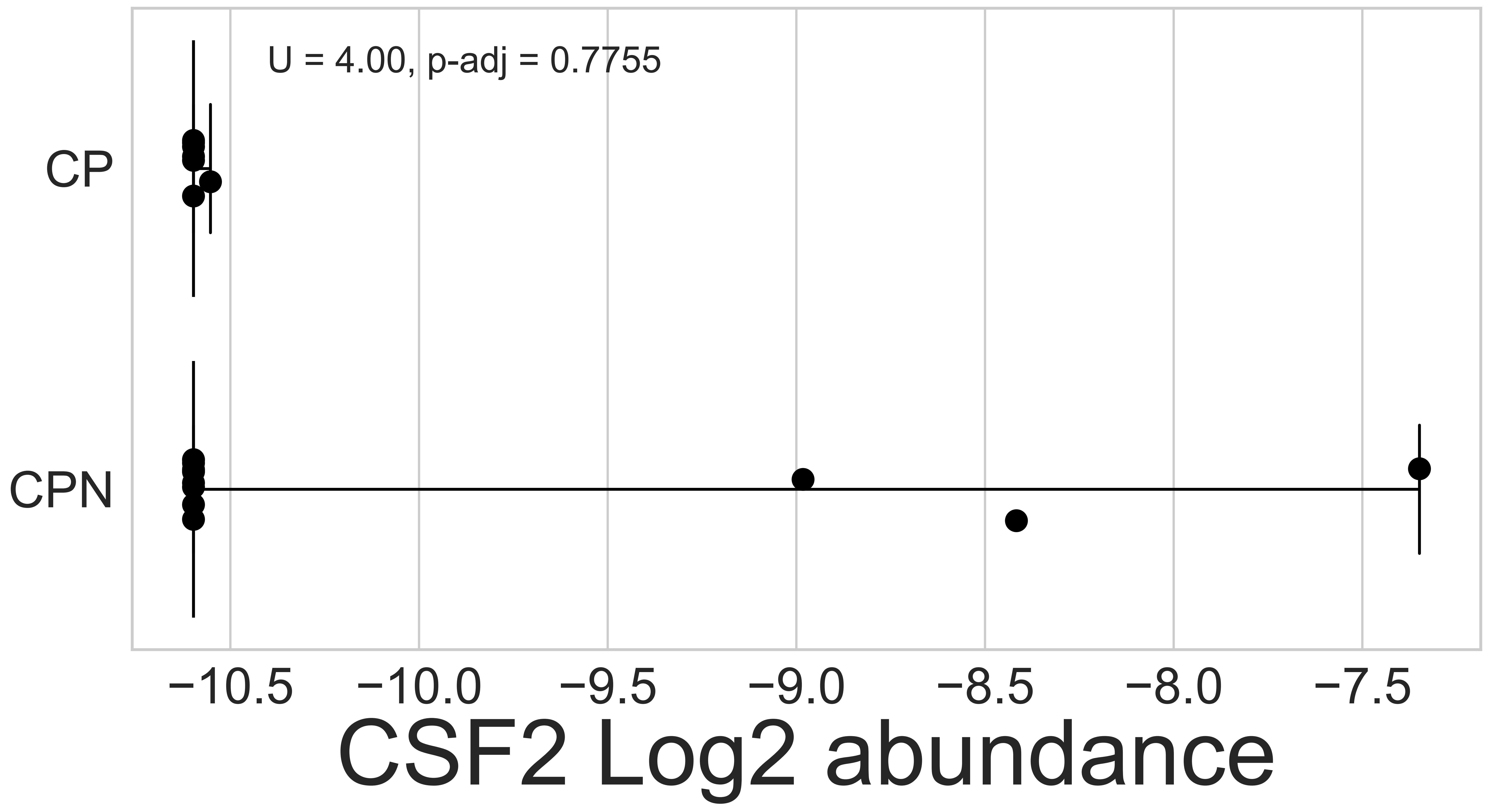
U = 4.00, p-adj = 0.7755
CP
CPN
−10.5
−10.0
−9.5
−9.0
−8.5
−8.0
−7.5
CSF2 Log2 abundance

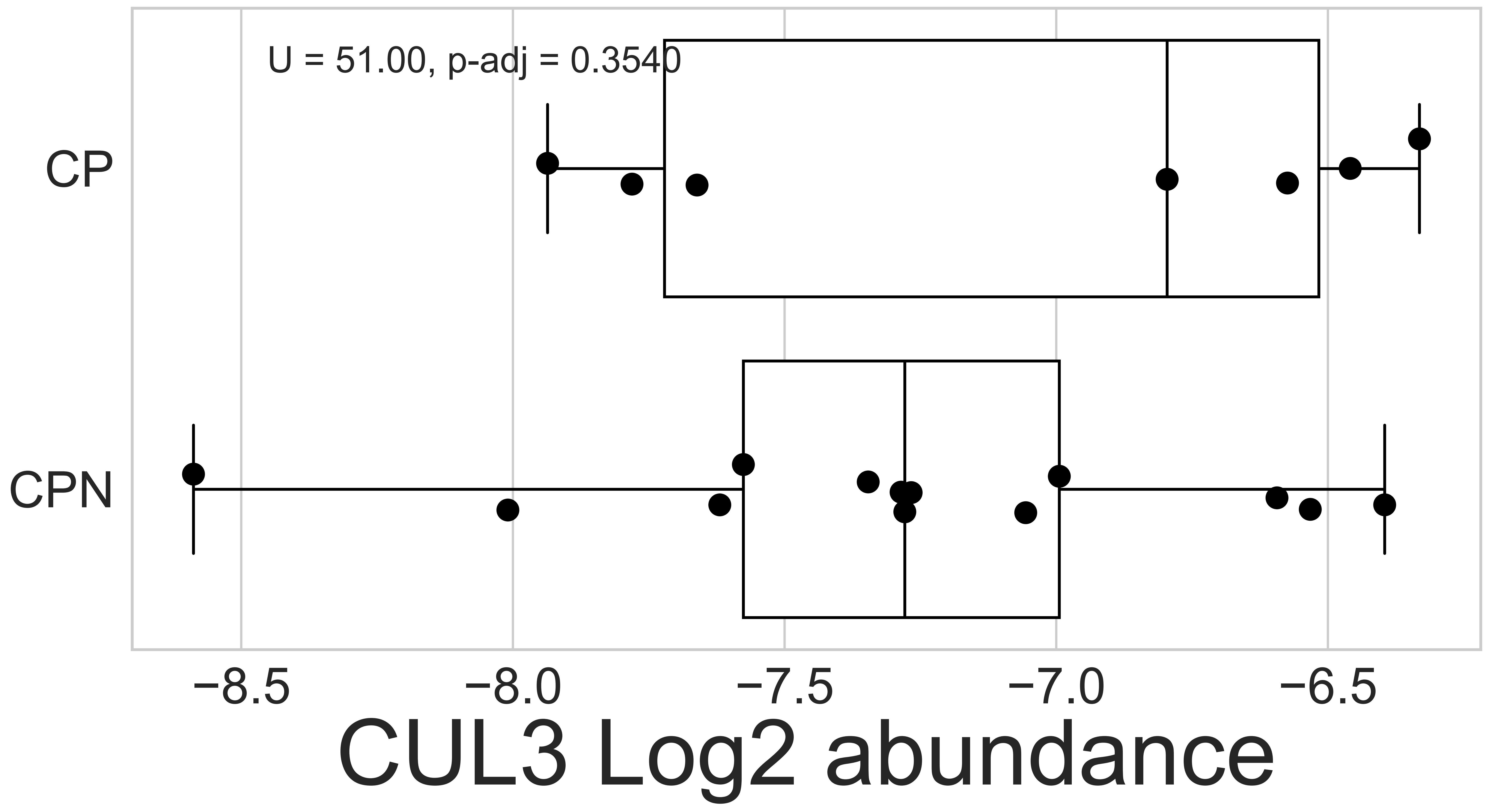
U = 51.00, p-adj = 0.3540
CP
CPN
−8.5
−8.0
−7.5
−7.0
−6.5
CUL3 Log2 abundance

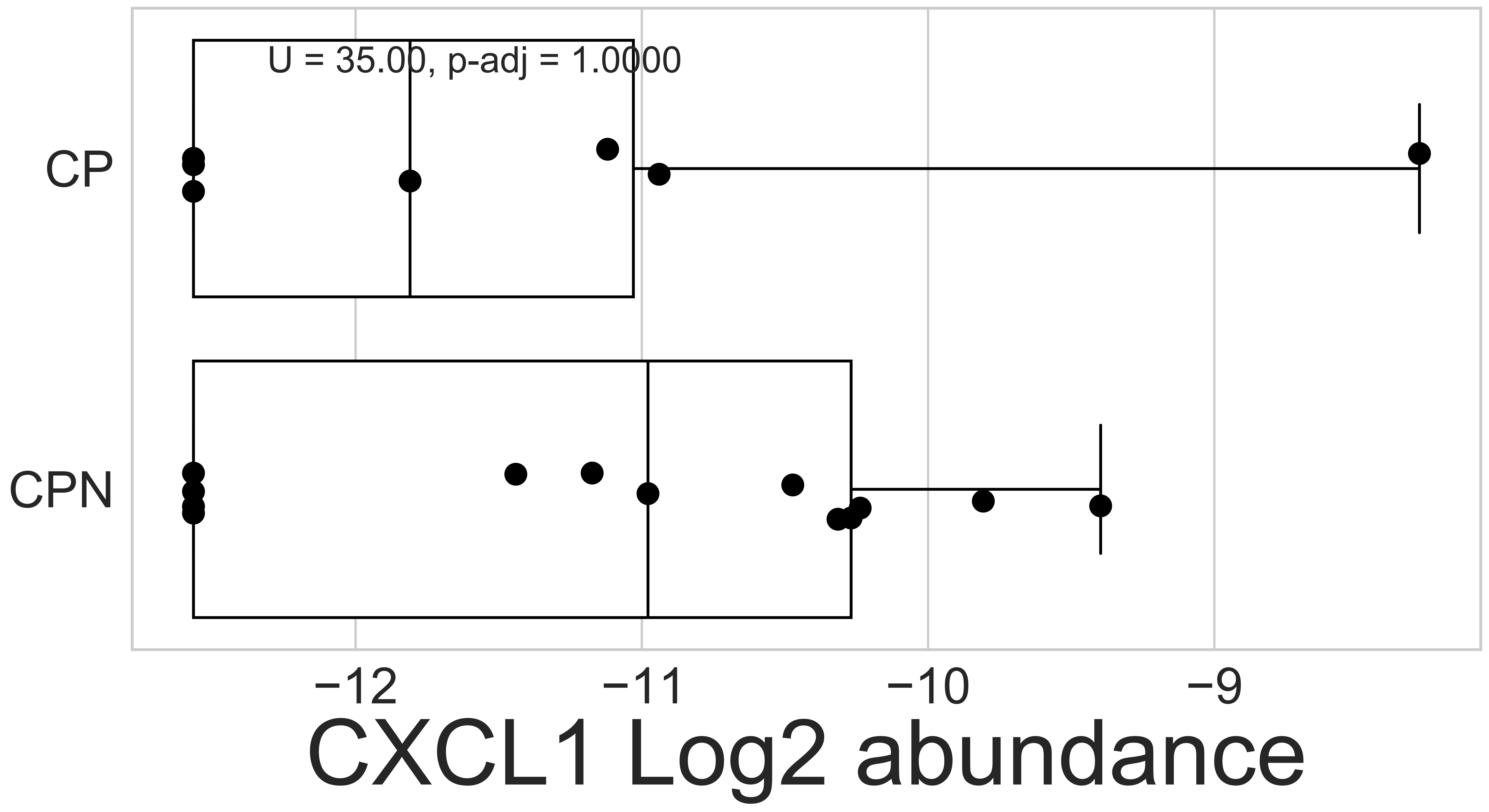
U = 35.00, p-adj = 1.0000
CP
CPN
−12
−11
−10
−9
CXCL1 Log2 abundance

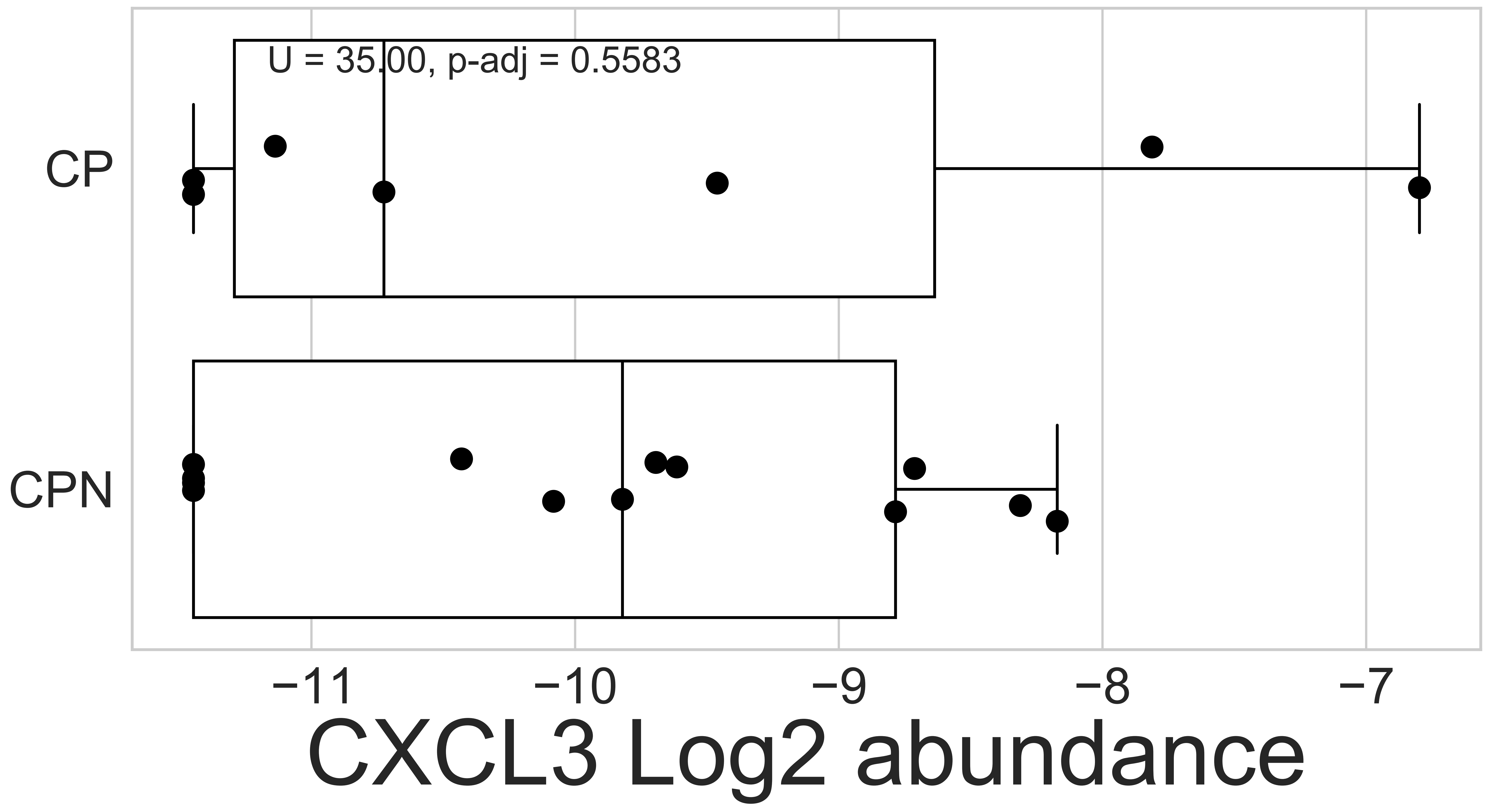
U = 35.00, p-adj = 0.5583
CP
CPN
−11
−10
−9
−8
−7
CXCL3 Log2 abundance

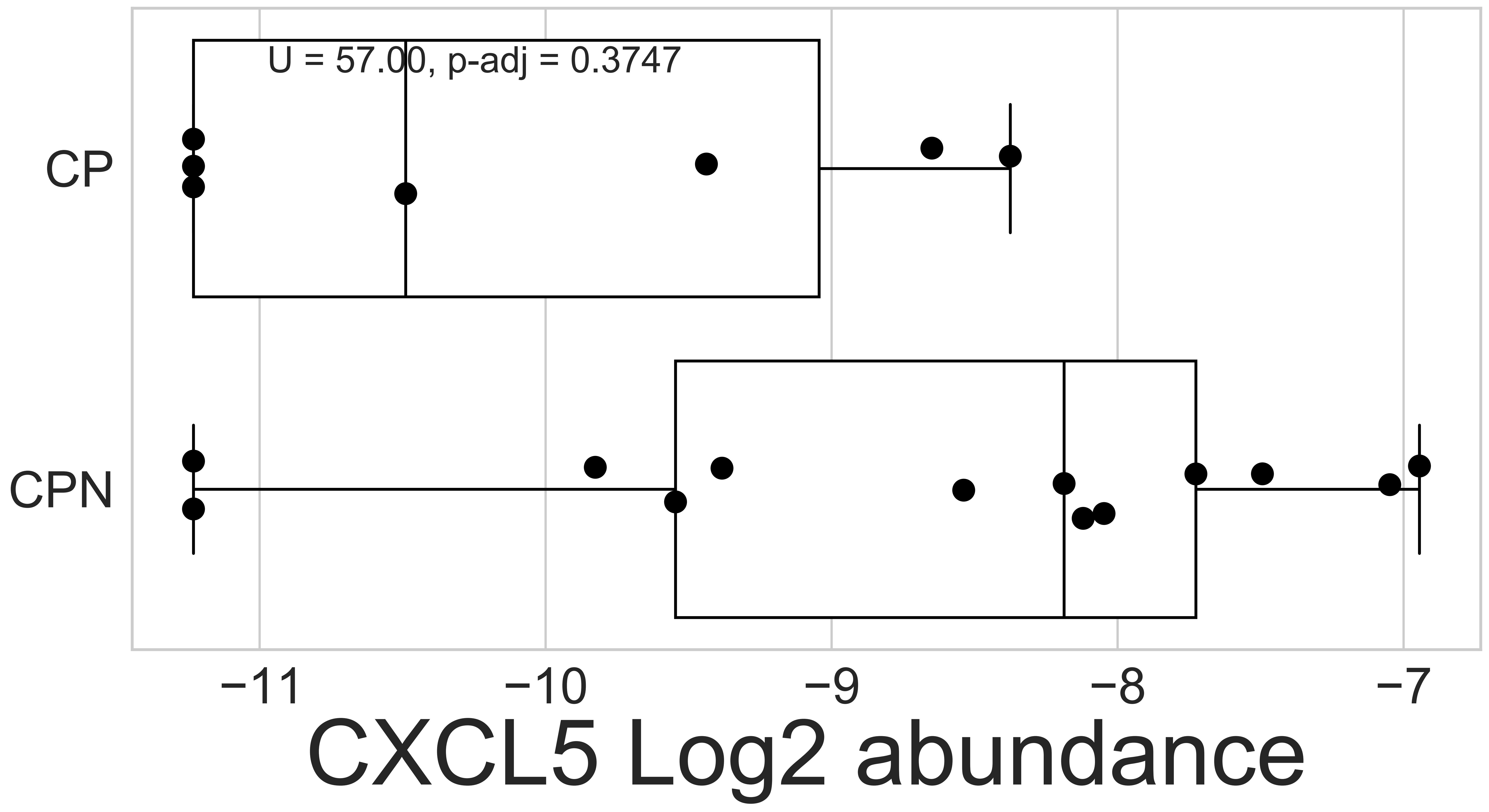
U = 57.00, p-adj = 0.3747
CP
CPN
−11
−10
−9
−8
−7
CXCL5 Log2 abundance

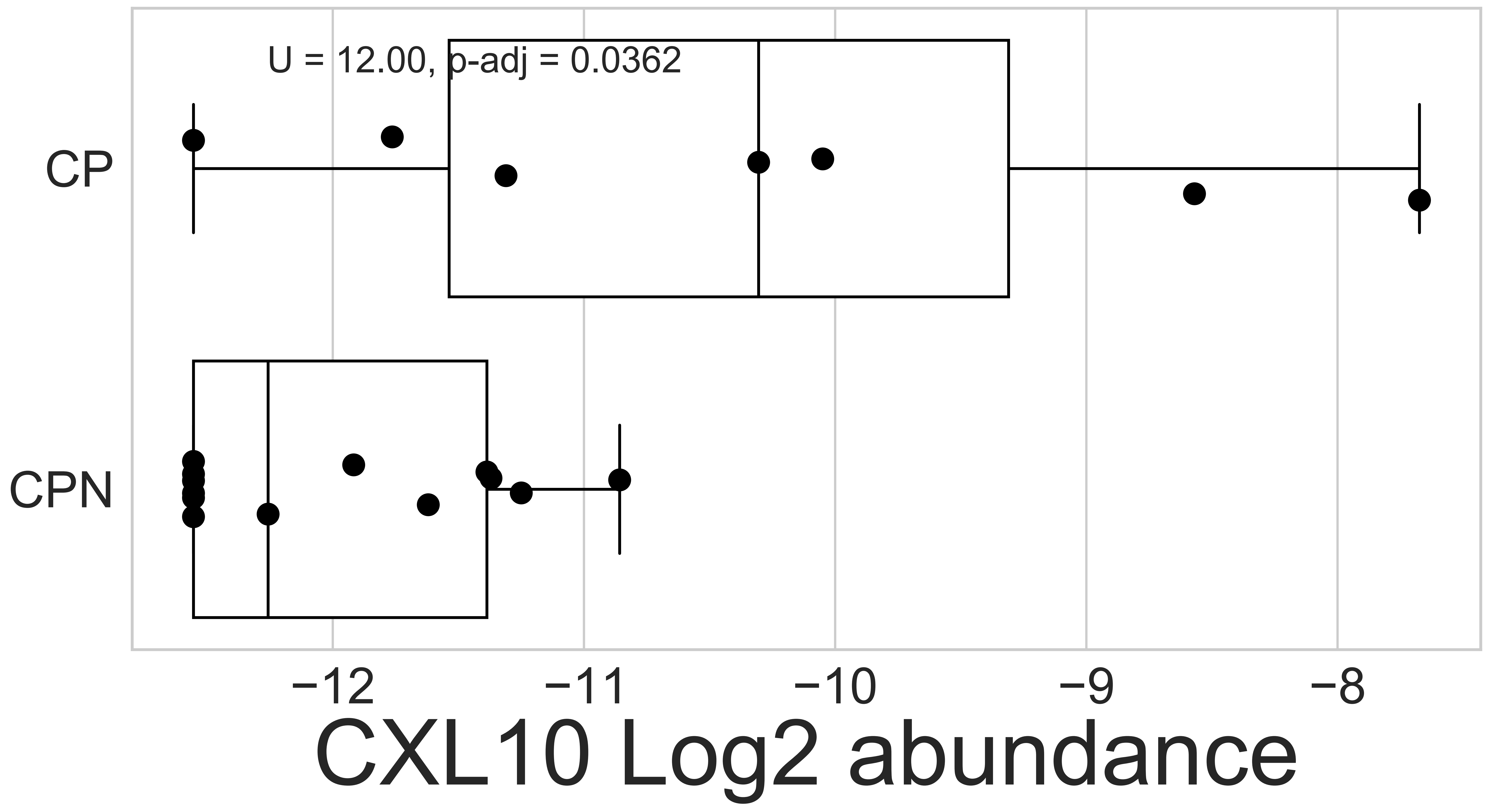
U = 12.00, p-adj = 0.0362
CP
CPN
−12
−11
−10
−9
−8
CXL10 Log2 abundance

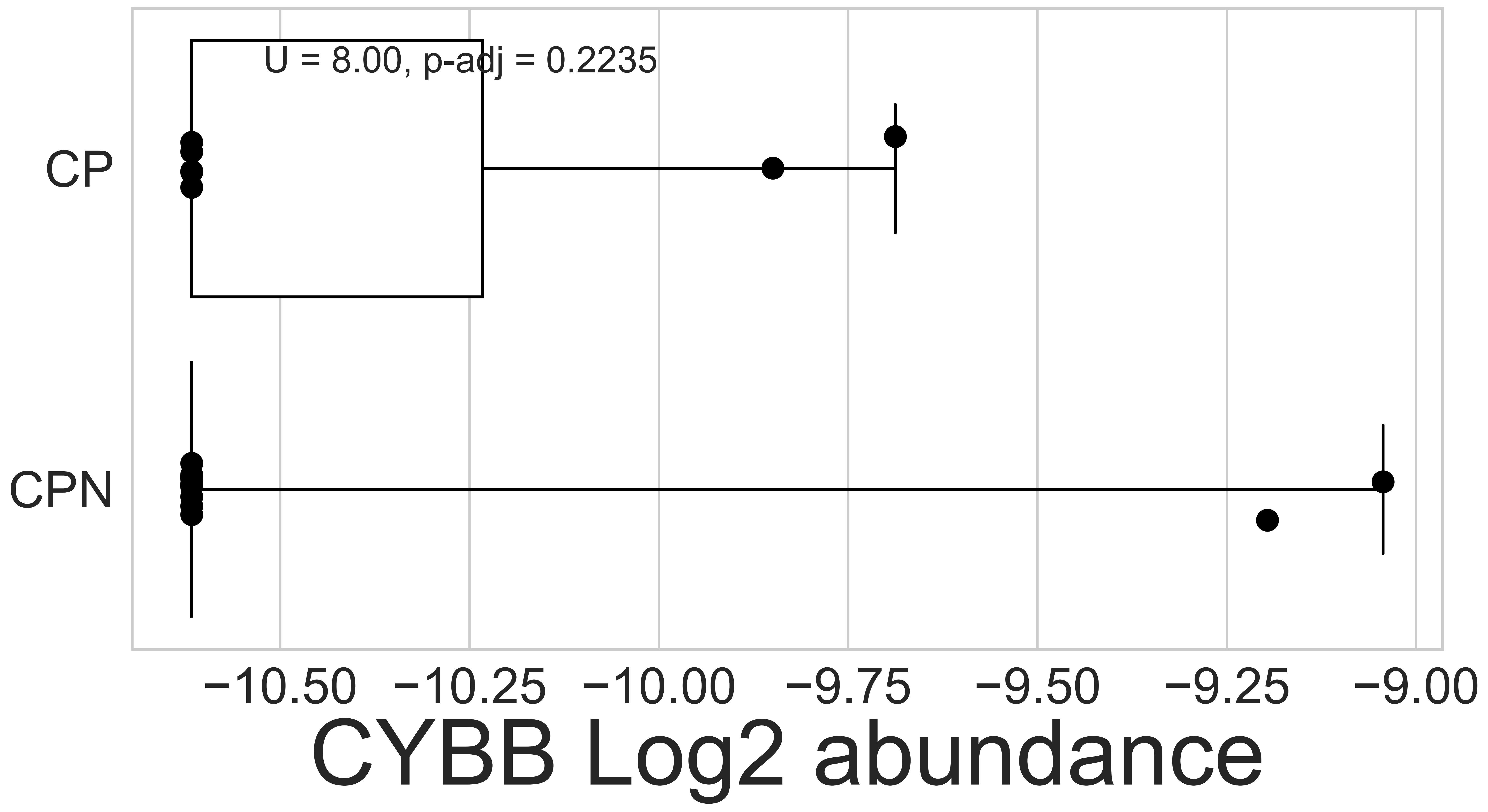
U = 8.00, p-adj = 0.2235
CP
CPN
−10.50
−10.25
−10.00
−9.75
−9.50
−9.25
−9.00
CYBB Log2 abundance

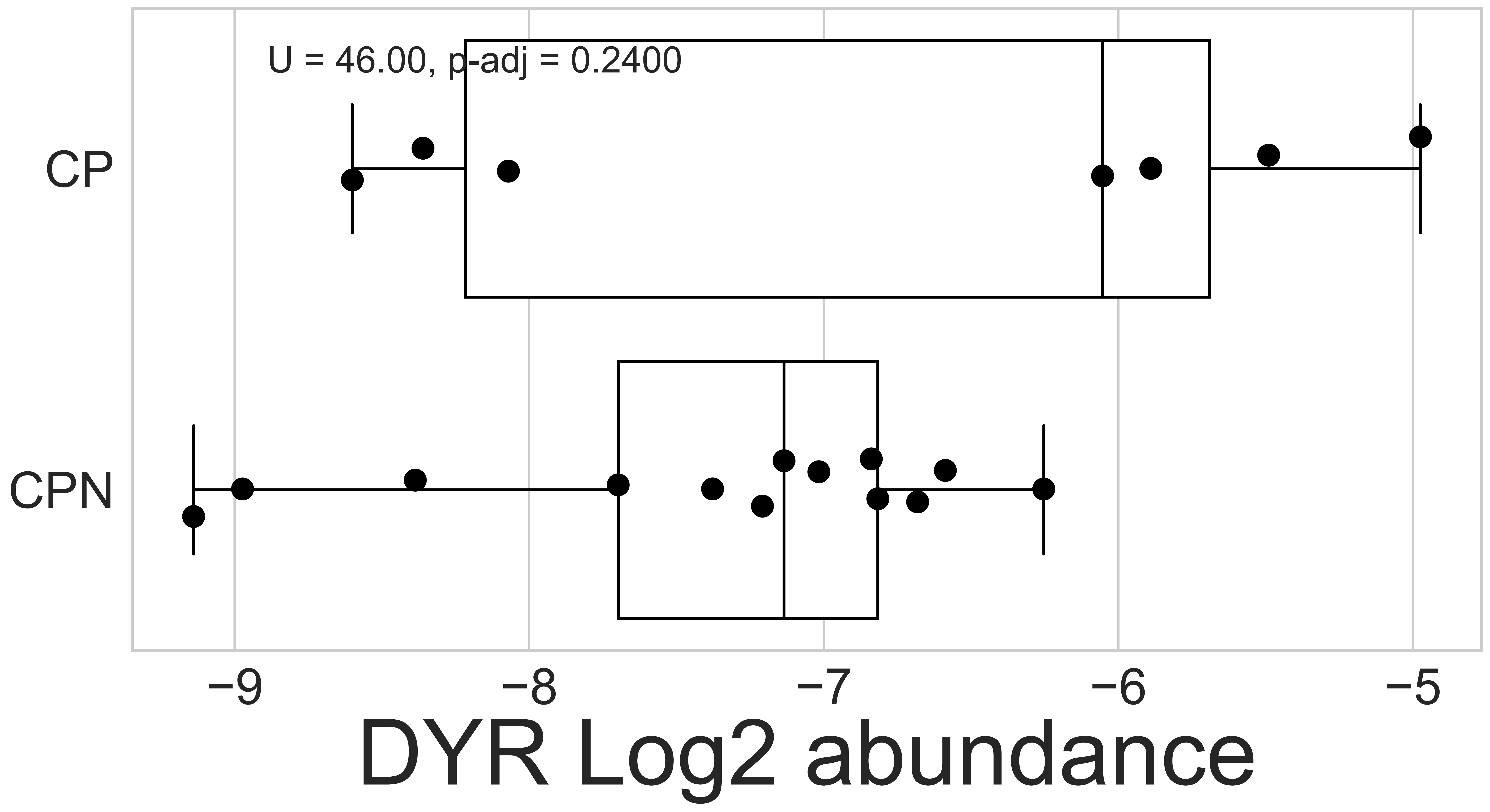

U = 46.00, p-adj = 0.2400
CP
CPN
−9
−8
−7
−6
−5
DYR Log2 abundance

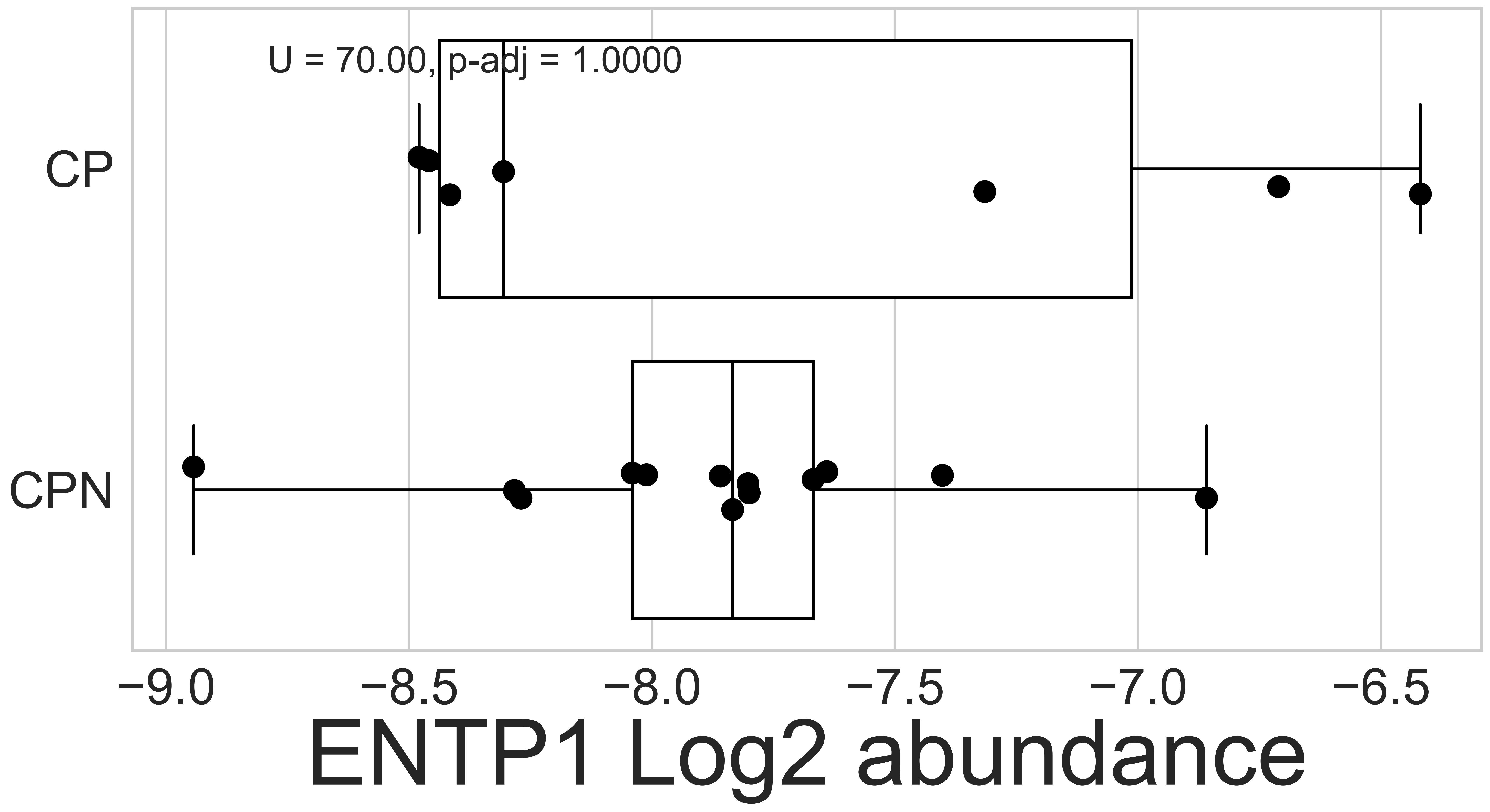

U = 70.00, p-adj = 1.0000
CP
CPN
−9.0
−8.5
−8.0
−7.5
−7.0
−6.5
ENTP1 Log2 abundance

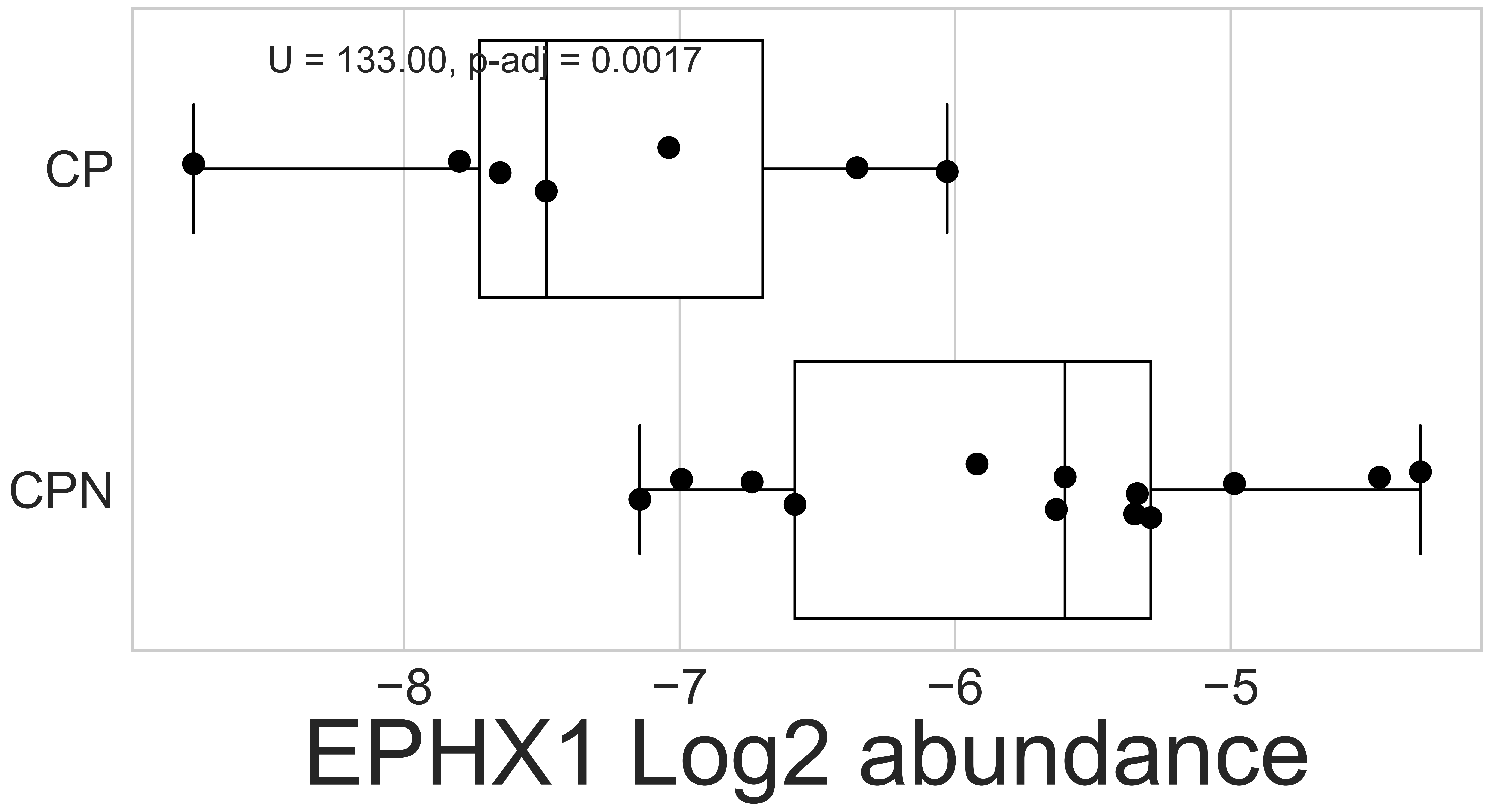
U = 133.00, p-adj = 0.0017
CP
CPN
−8
−7
−6
−5
EPHX1 Log2 abundance

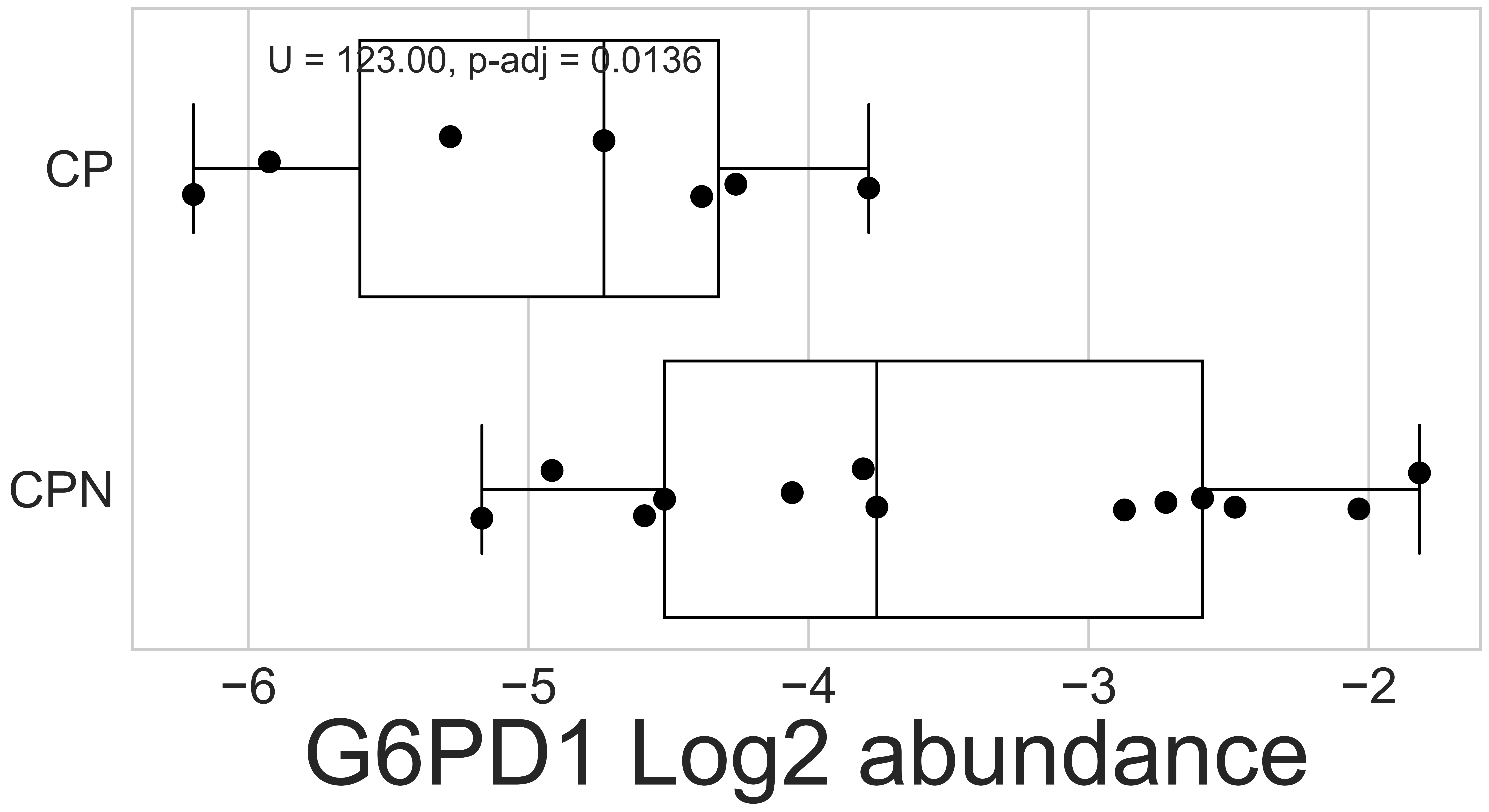
U = 123.00, p-adj = 0.0136
CP
CPN
−6
−5
−4
−3
−2
G6PD1 Log2 abundance

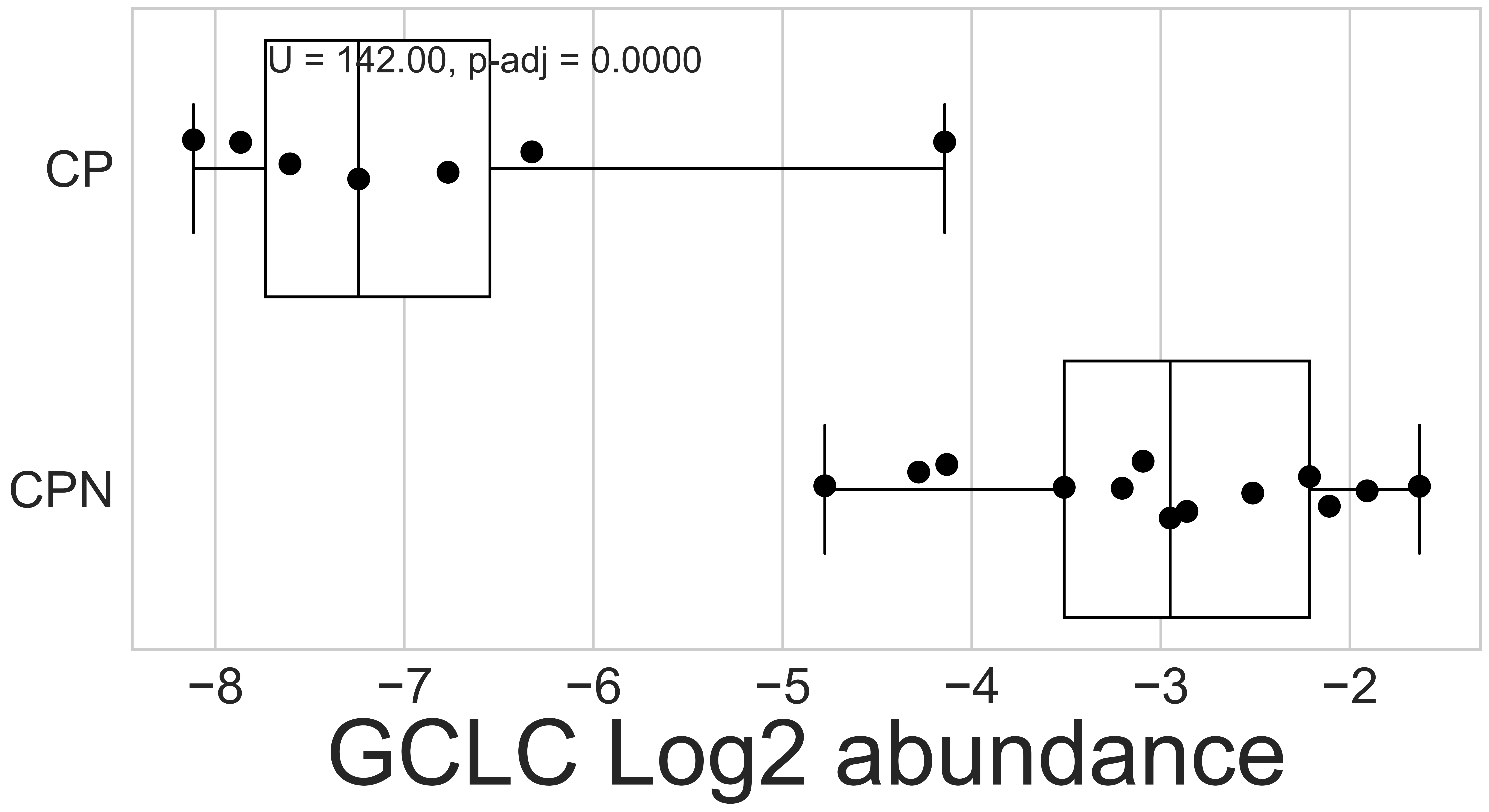
U = 142.00, p-adj = 0.0000
CP
CPN
−8
−7
−6
−5
−4
−3
−2
GCLC Log2 abundance

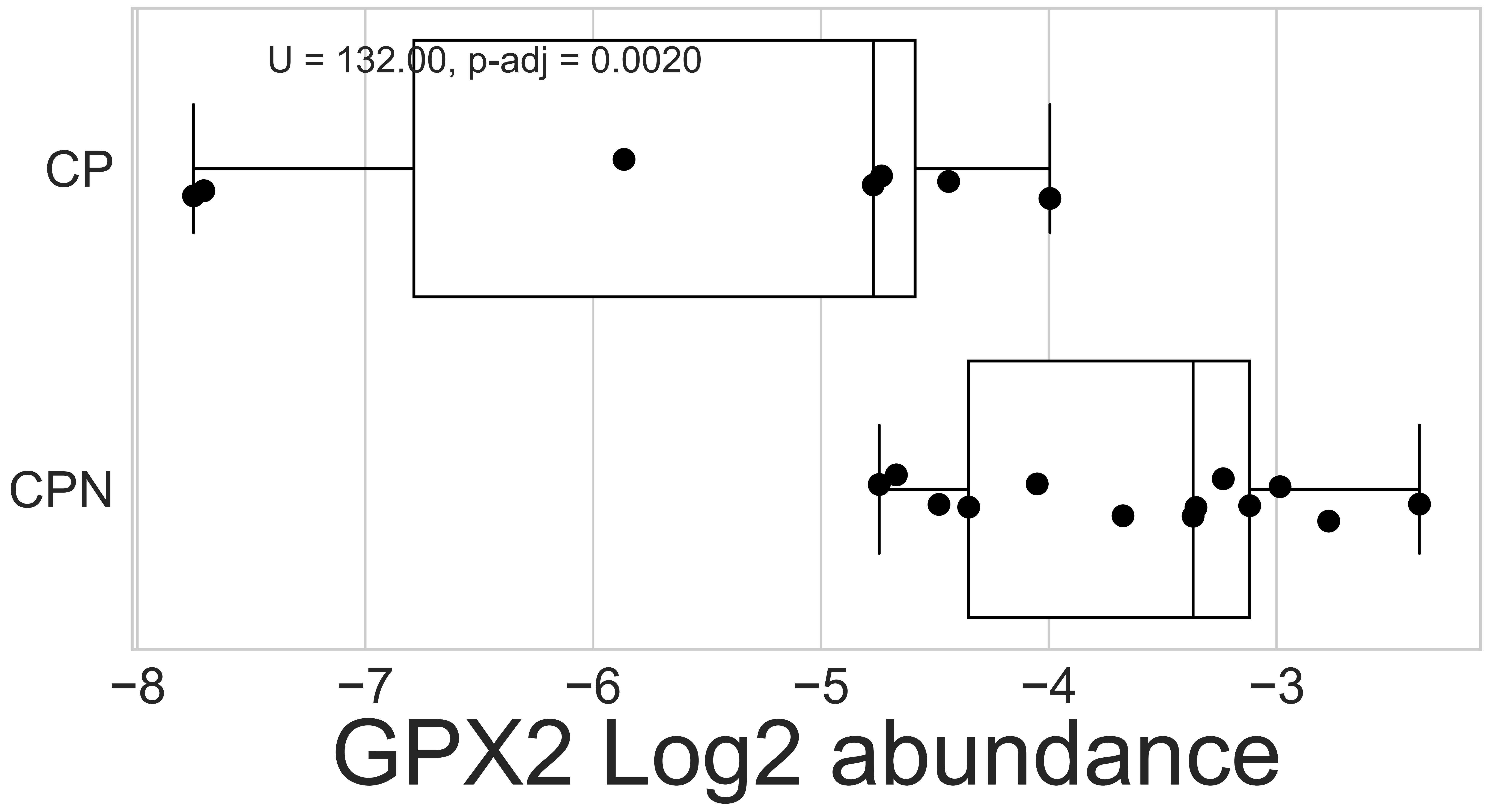
U = 132.00, p-adj = 0.0020
CP
CPN
−8
−7
−6
−5
−4
−3
GPX2 Log2 abundance

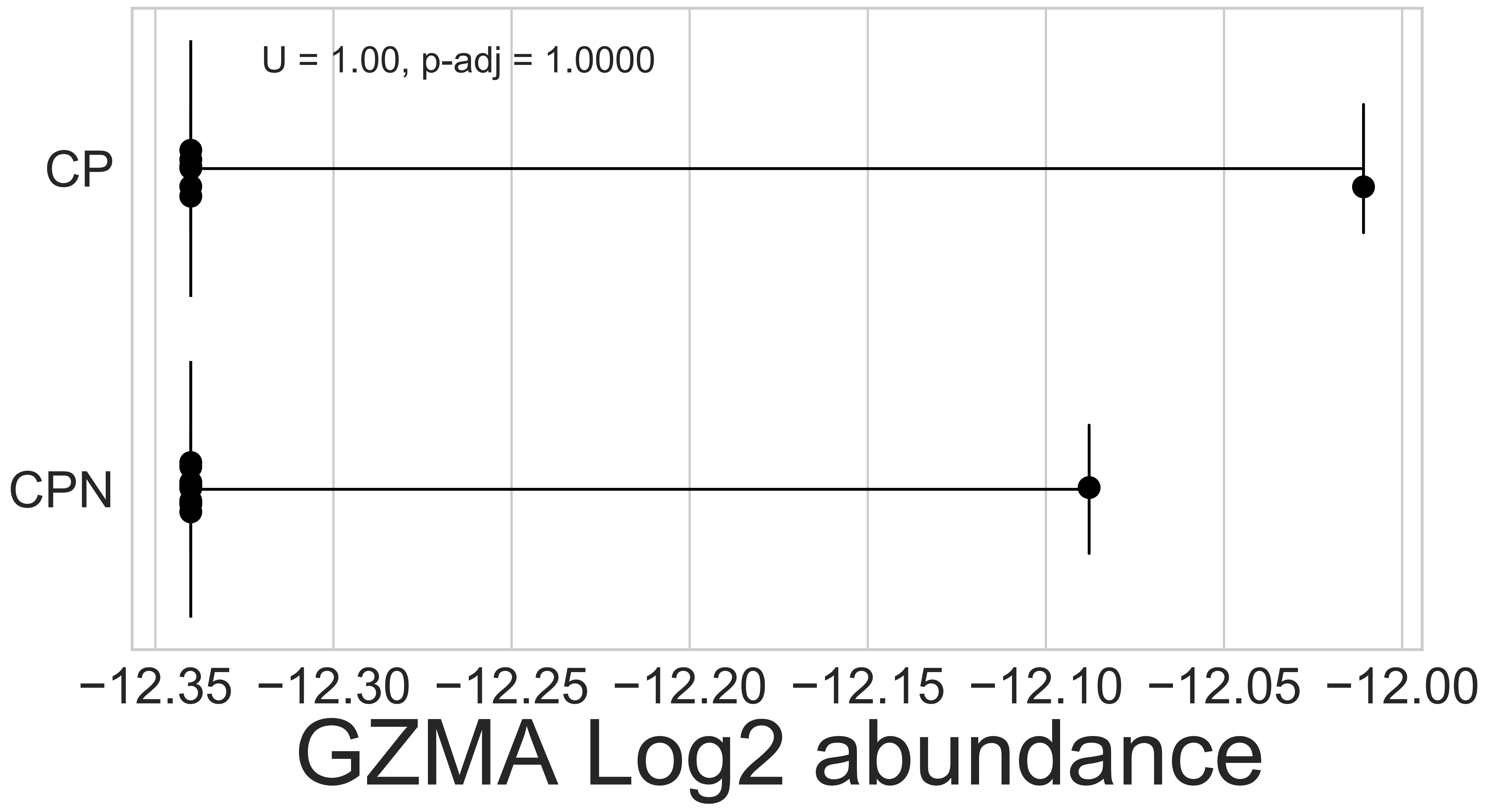
U = 1.00, p-adj = 1.0000
CP
CPN
−12.35
−12.30
−12.25
−12.20
−12.15
−12.10
−12.05
−12.00
GZMA Log2 abundance

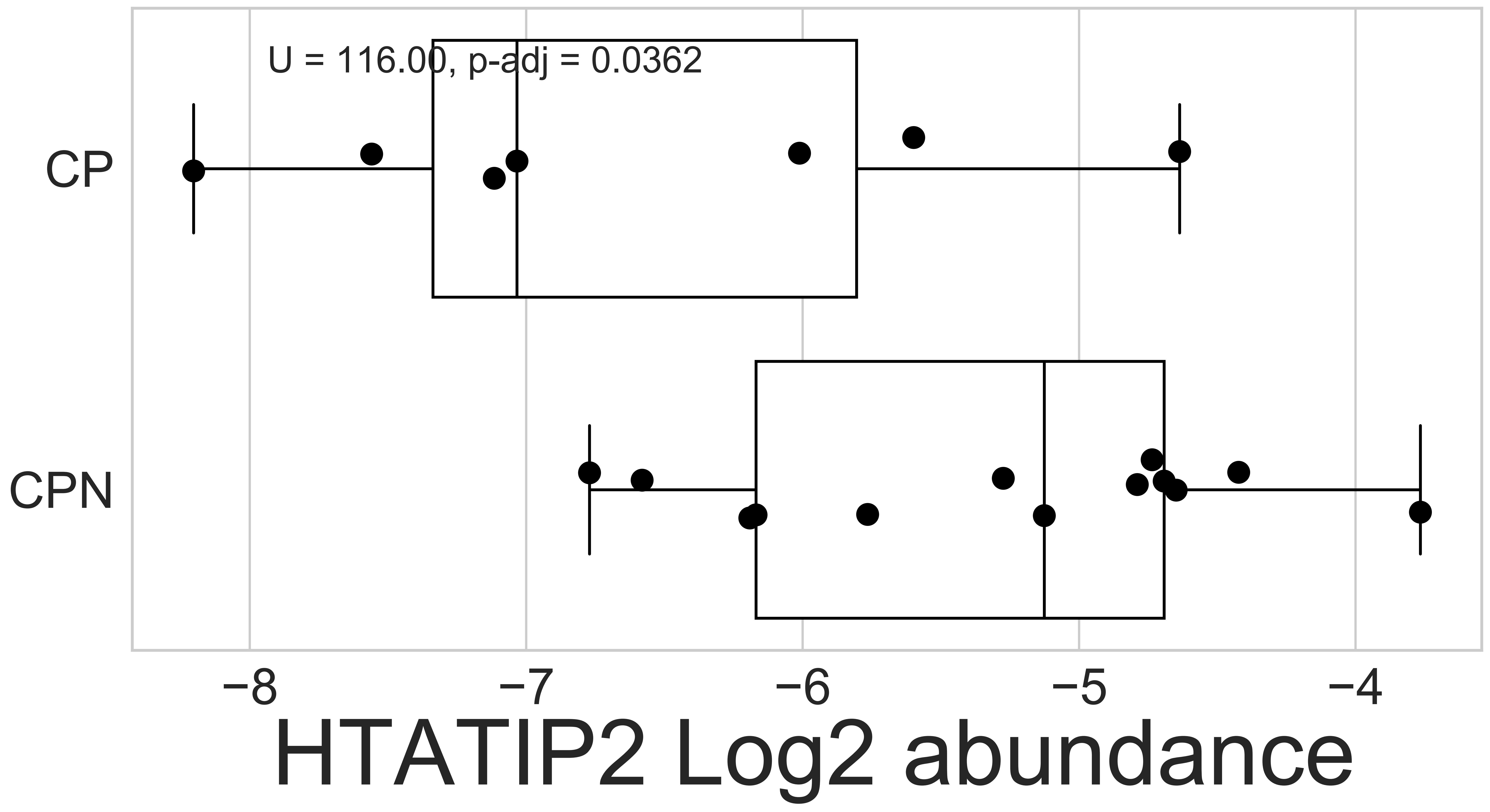
U = 116.00, p-adj = 0.0362
CP
CPN
−8
−7
−6
−5
−4
HTATIP2 Log2 abundance

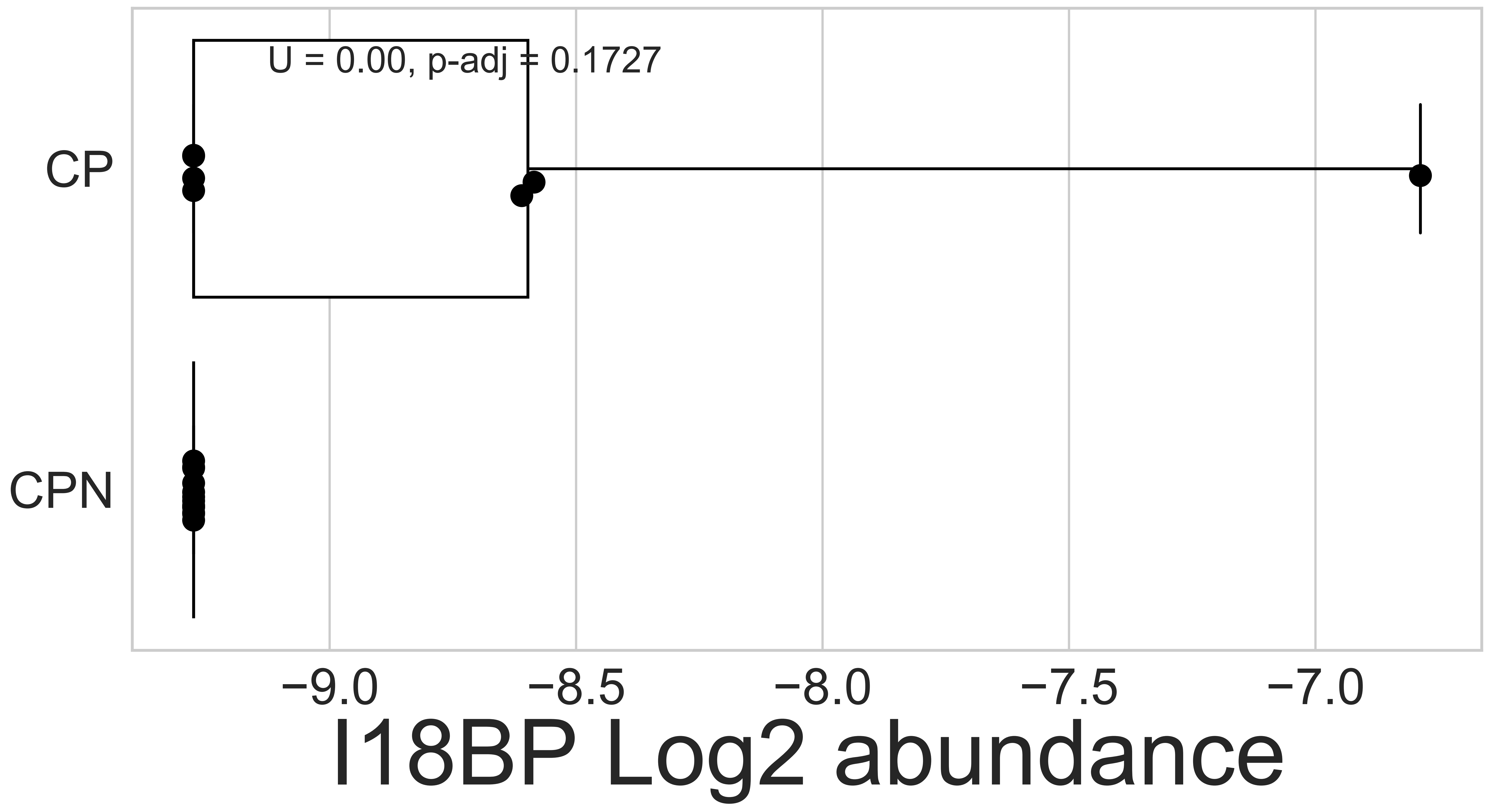

U = 0.00, p-adj = 0.1727
CP
CPN
−9.0
−8.5
−8.0
−7.5
−7.0
I18BP Log2 abundance

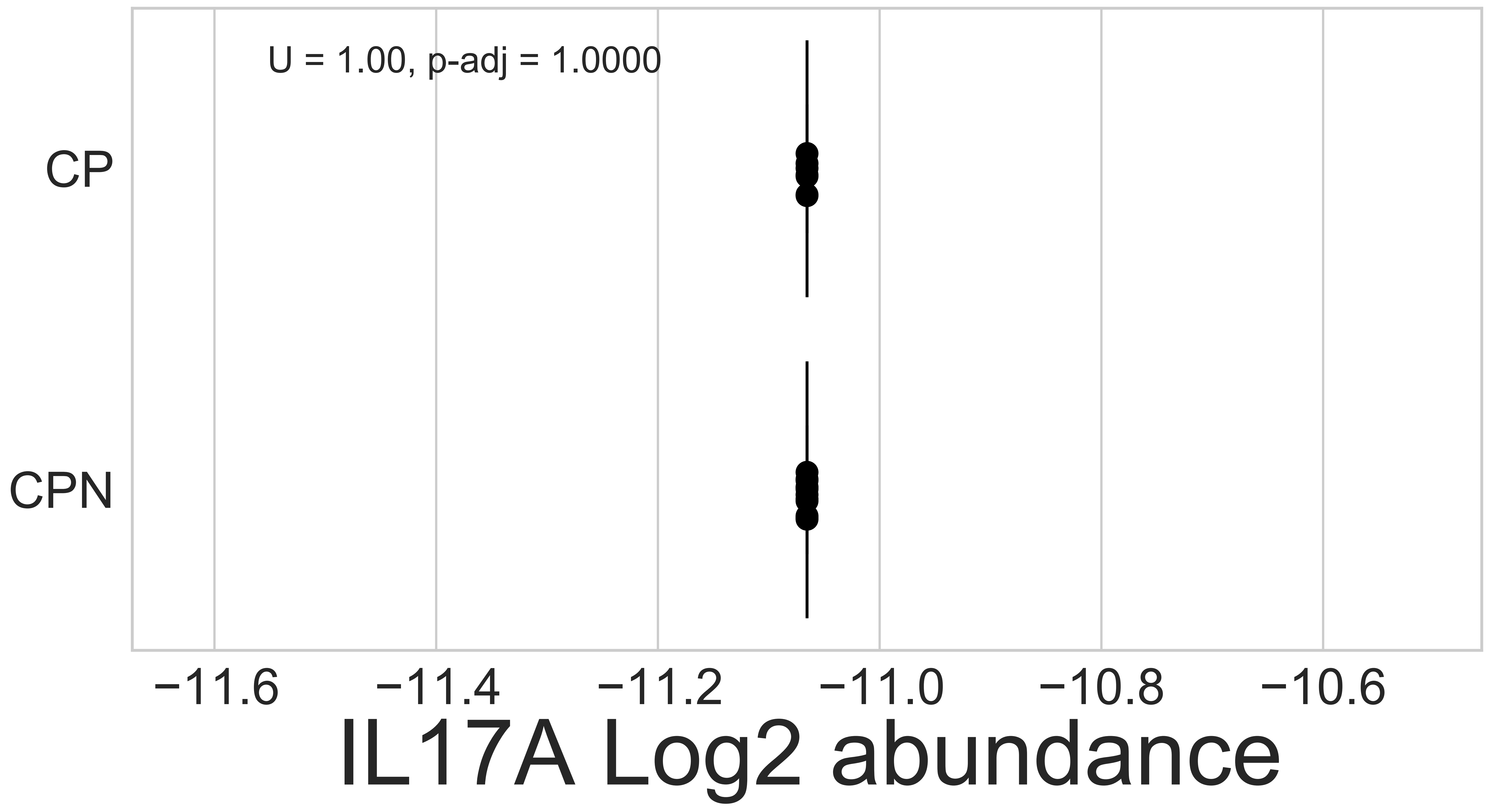

U = 1.00, p-adj = 1.0000
CP
CPN
−11.6
−11.4
−11.2
−11.0
−10.8
−10.6
IL17A Log2 abundance

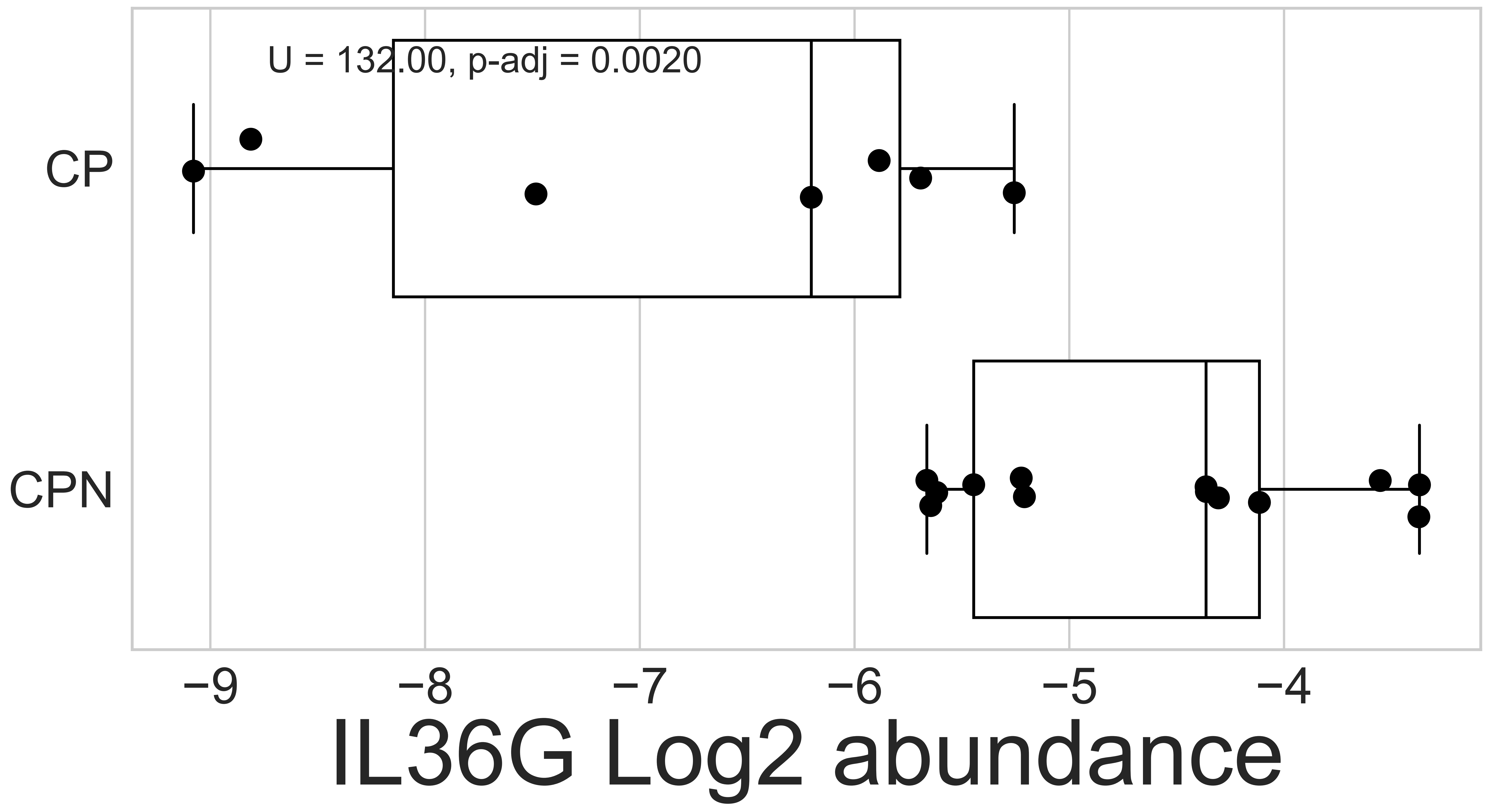
U = 132.00, p-adj = 0.0020
CP
CPN
−9
−8
−7
−6
−5
−4
IL36G Log2 abundance

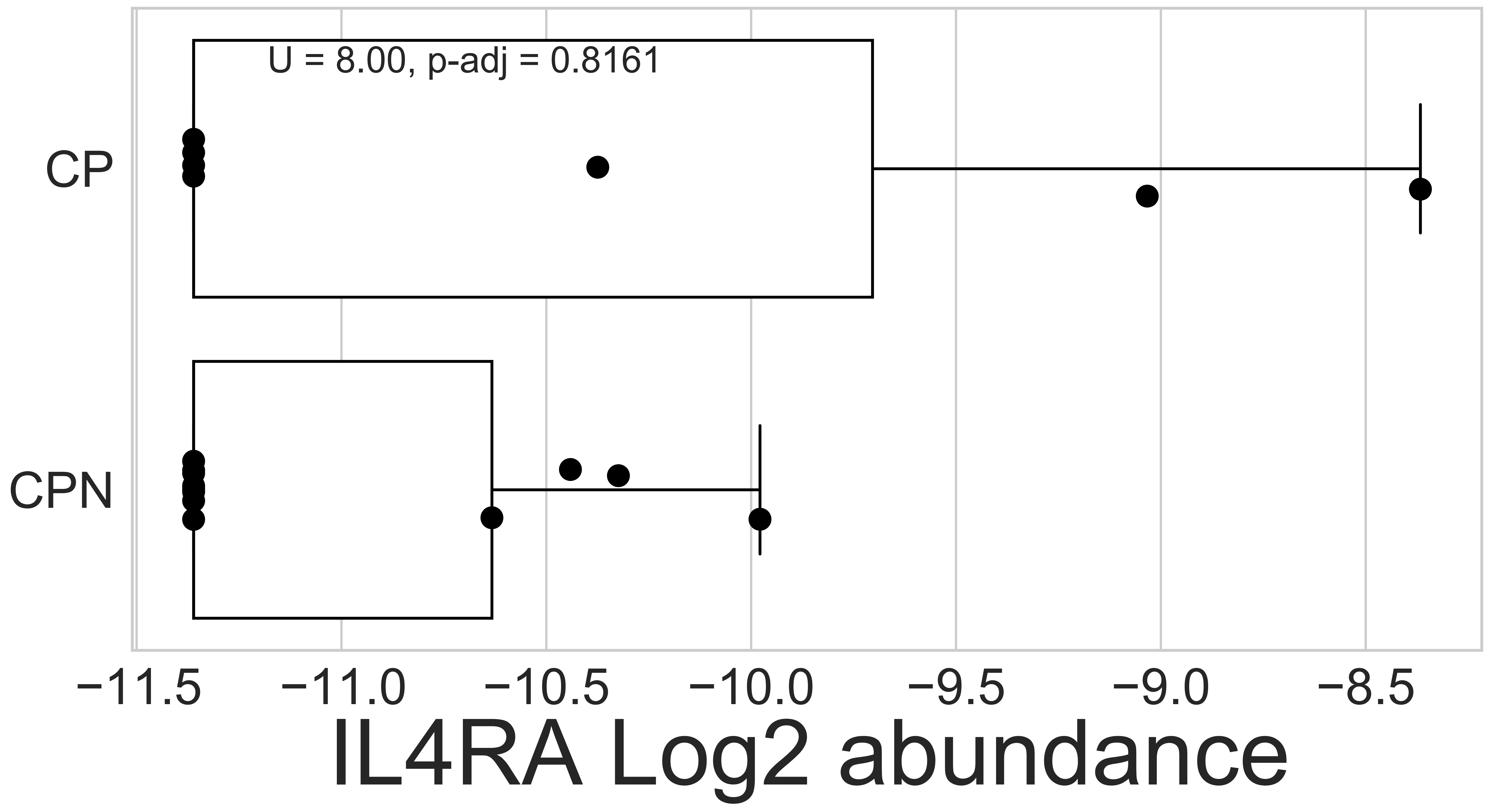
U = 8.00, p-adj = 0.8161
CP
CPN
−11.5
−11.0
−10.5
−10.0
−9.5
−9.0
−8.5
IL4RA Log2 abundance

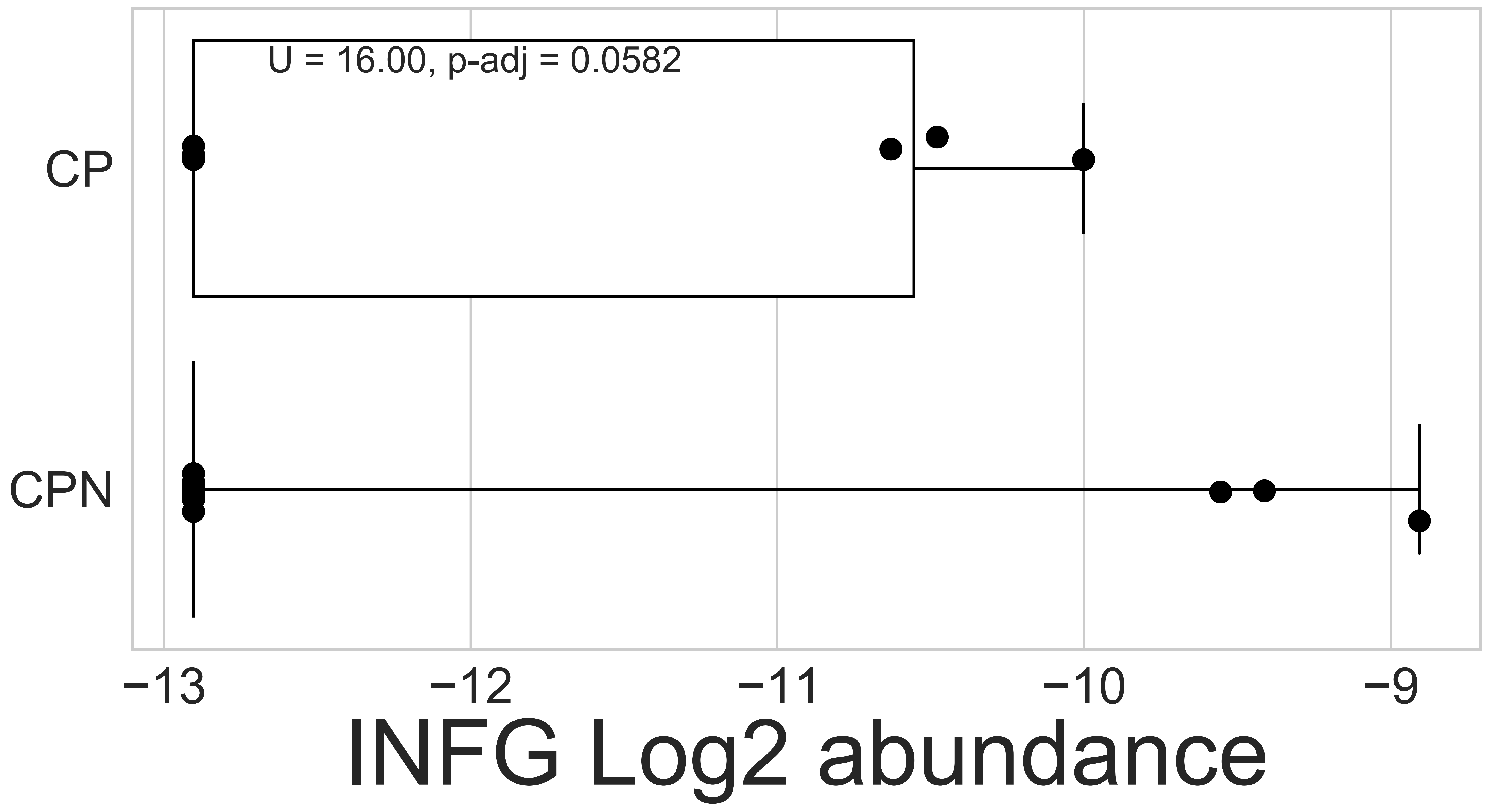

U = 16.00, p-adj = 0.0582
CP
CPN
−13
−12
−11
−10
−9
INFG Log2 abundance

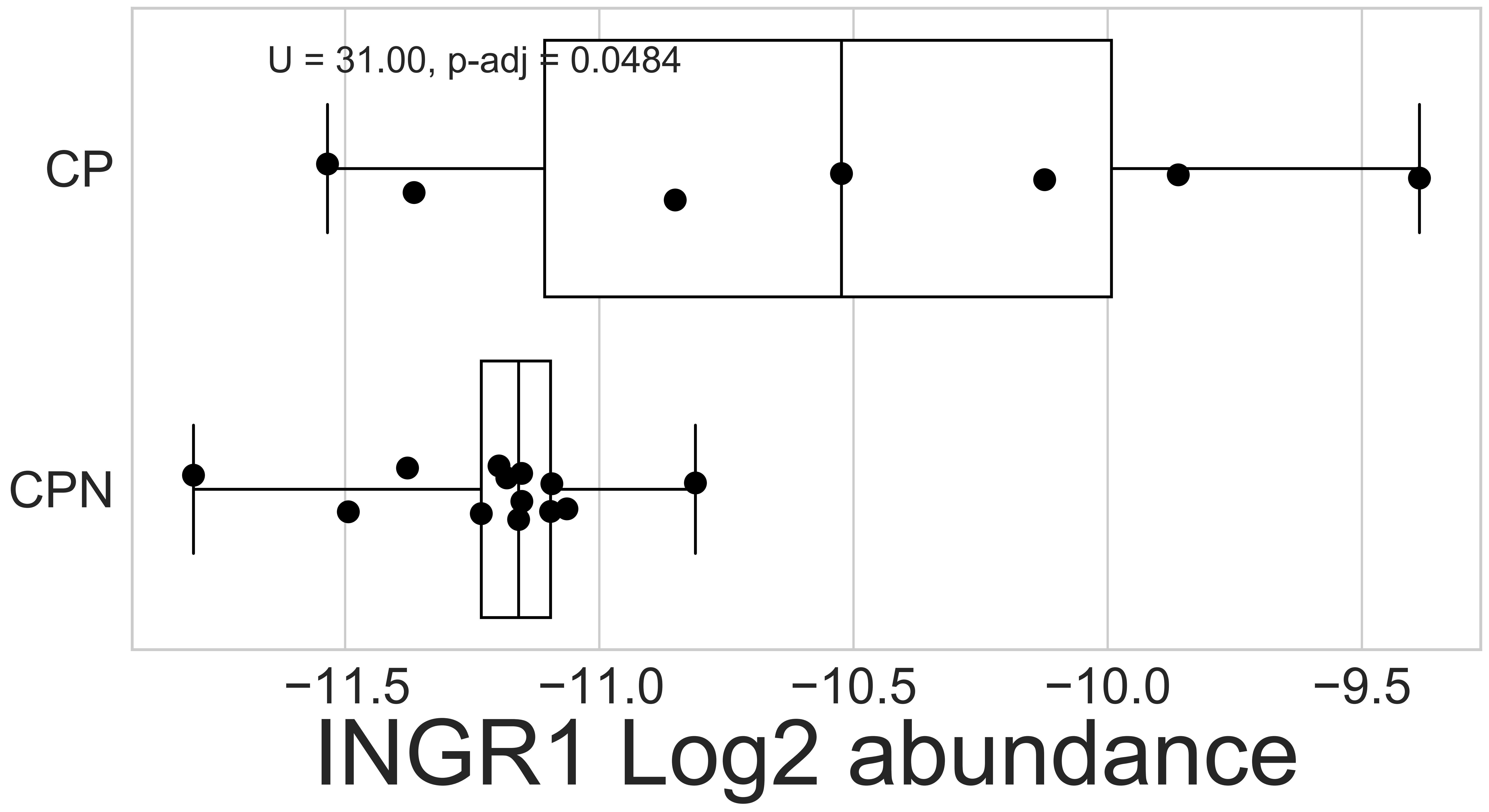
U = 31.00, p-adj = 0.0484
CP
CPN
−11.5
−11.0
−10.5
−10.0
−9.5
INGR1 Log2 abundance

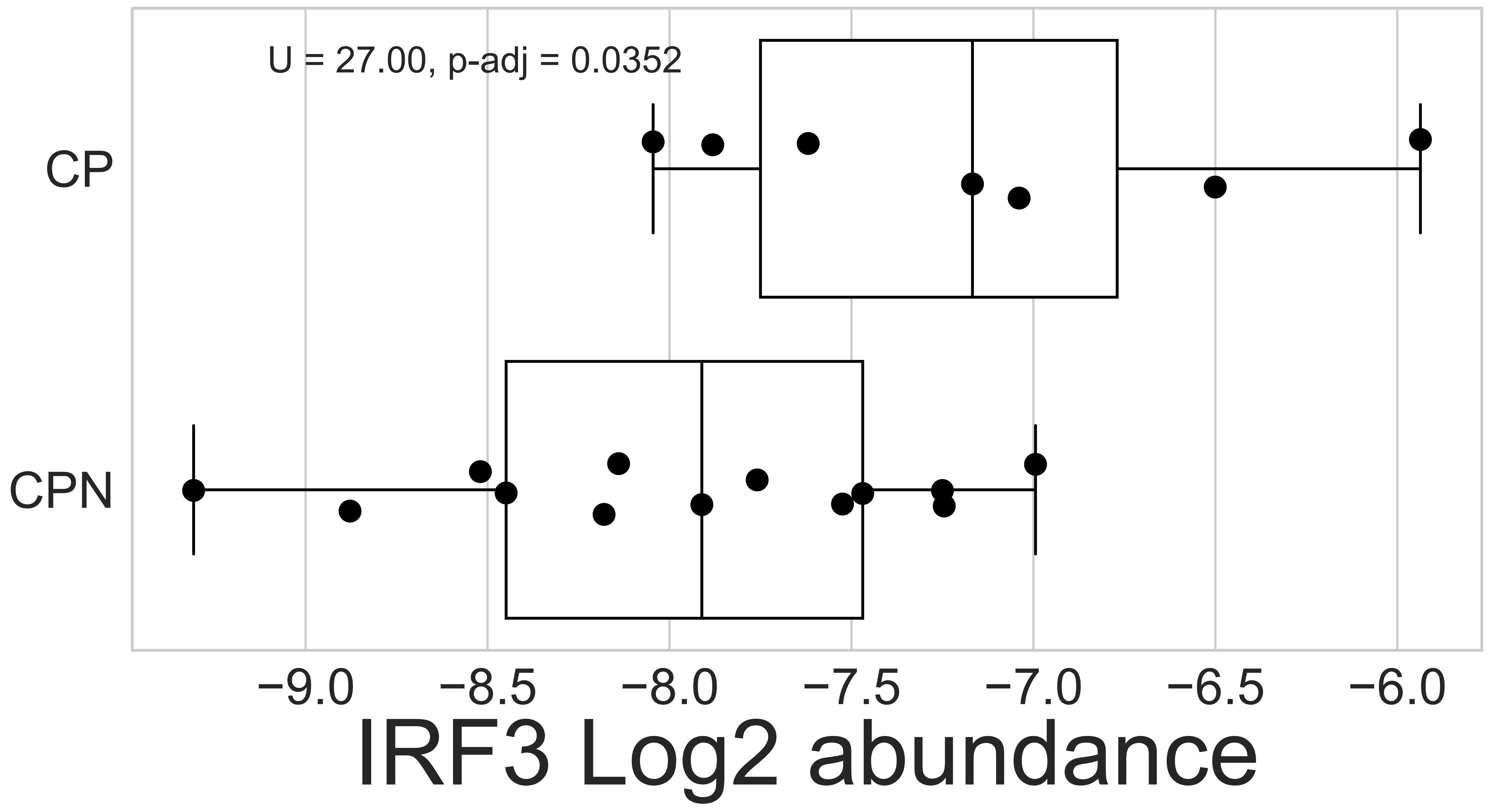
U = 27.00, p-adj = 0.0352
CP
CPN
−9.0
−8.5
−8.0
−7.5
−7.0
−6.5
−6.0
IRF3 Log2 abundance

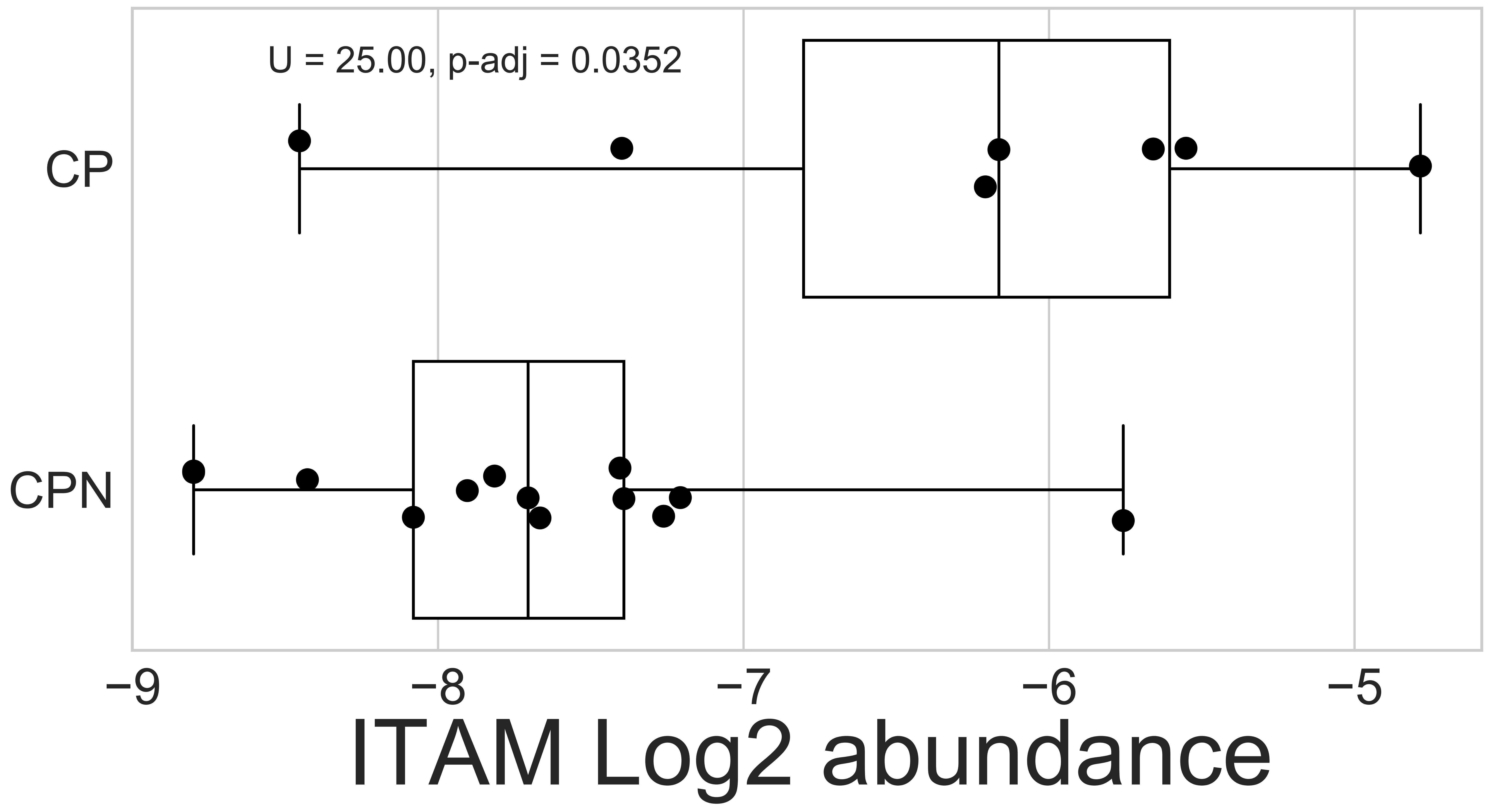

U = 25.00, p-adj = 0.0352
CP
CPN
−9
−8
−7
−6
−5
ITAM Log2 abundance

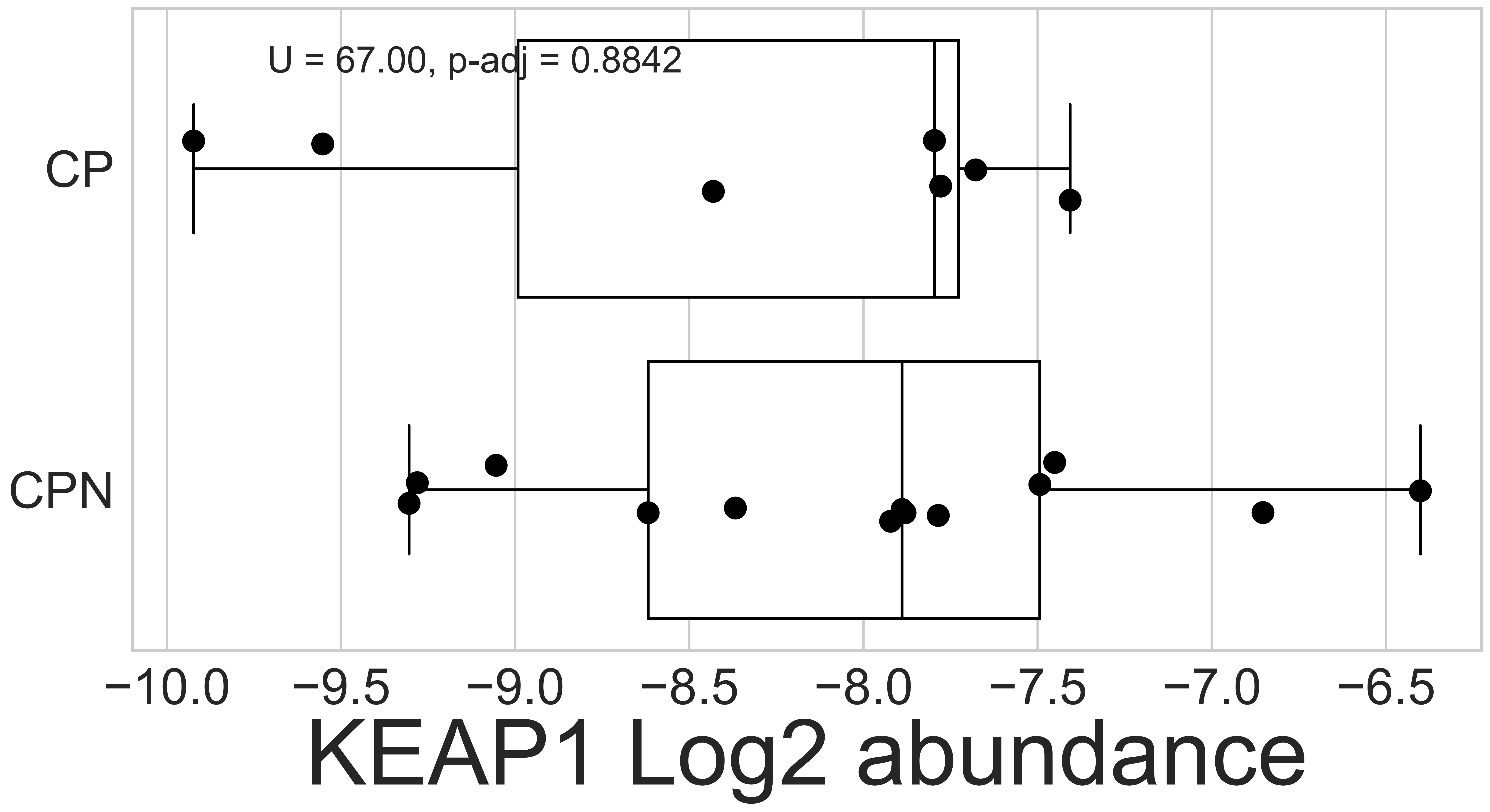
U = 67.00, p-adj = 0.8842
CP
CPN
−10.0
−9.5
−9.0
−8.5
−8.0
−7.5
−7.0
−6.5
KEAP1 Log2 abundance

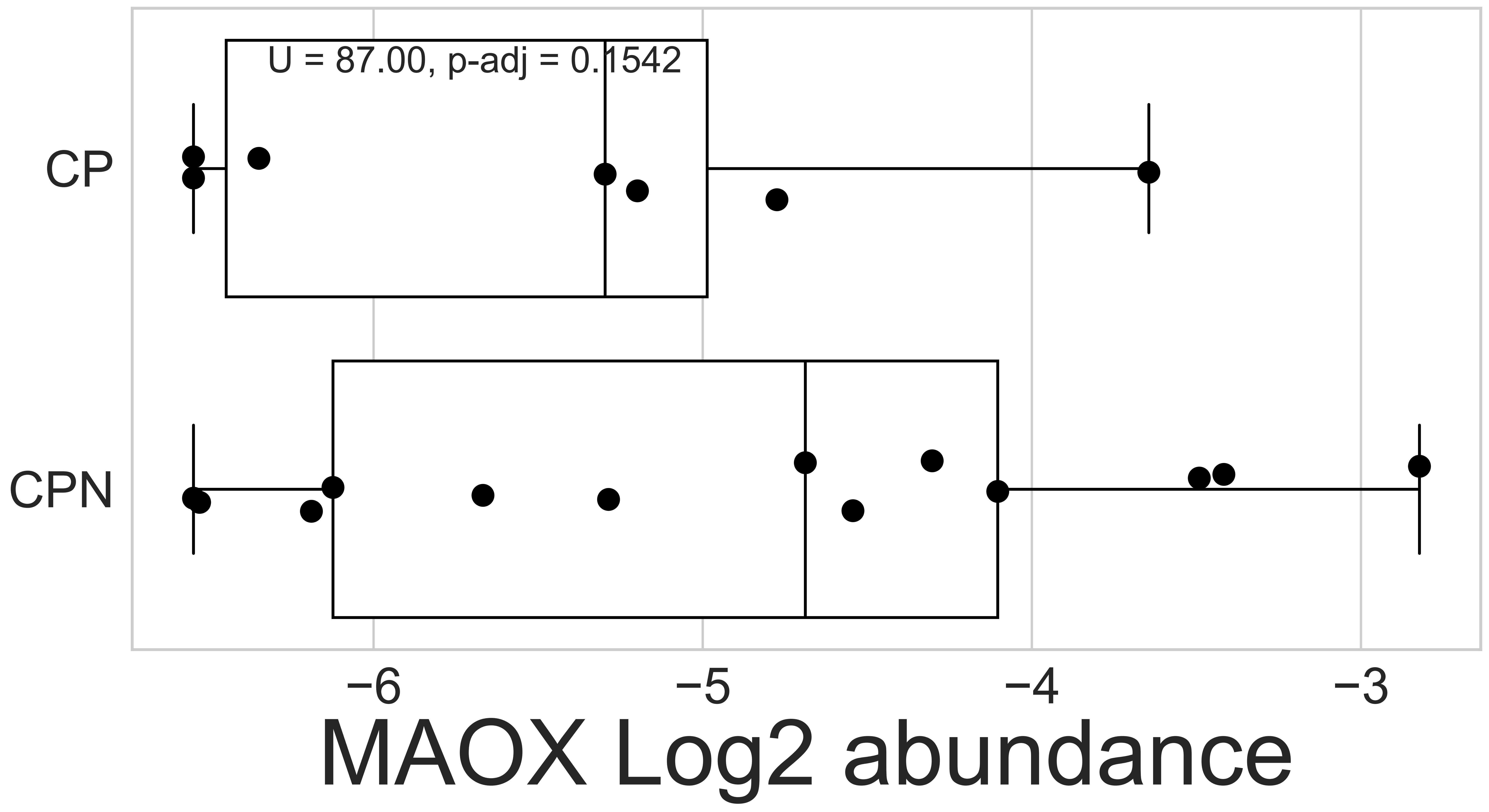
U = 87.00, p-adj = 0.1542
CP
CPN
−6
−5
−4
−3
MAOX Log2 abundance

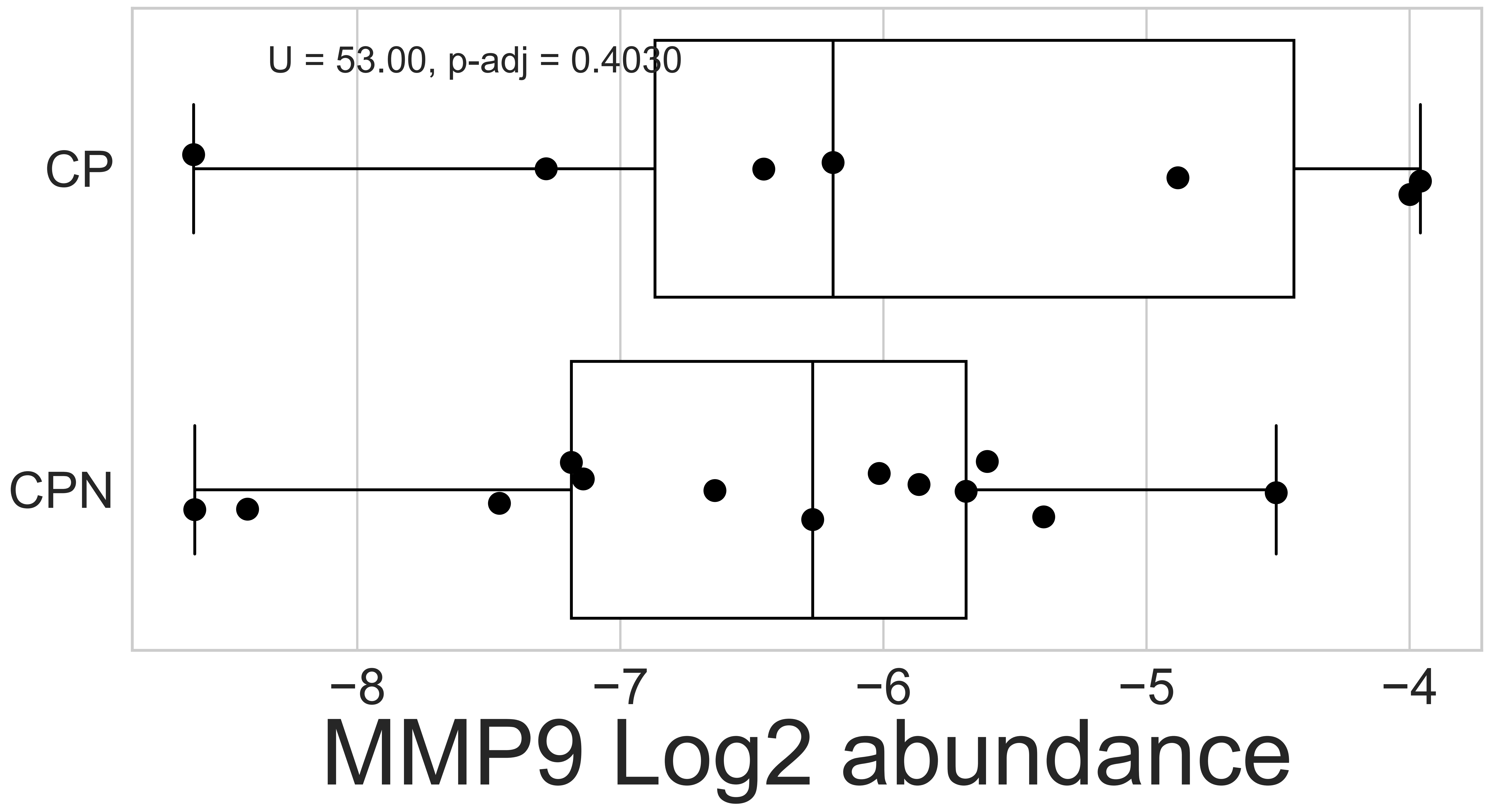
U = 53.00, p-adj = 0.4030
CP
CPN
−8
−7
−6
−5
−4
MMP9 Log2 abundance

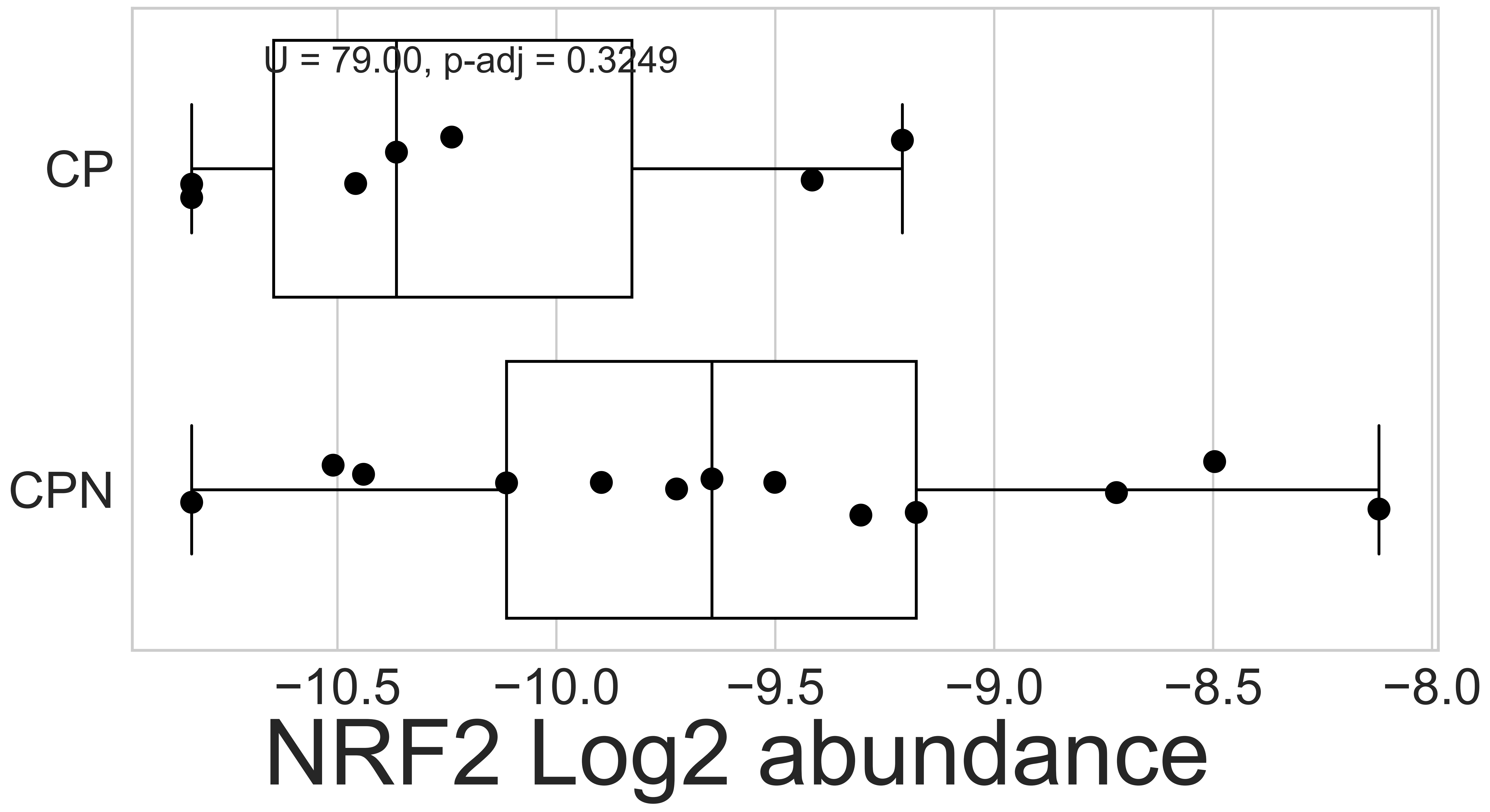
U = 79.00, p-adj = 0.3249
CP
CPN
−10.5
−10.0
−9.5
−9.0
−8.5
−8.0
NRF2 Log2 abundance

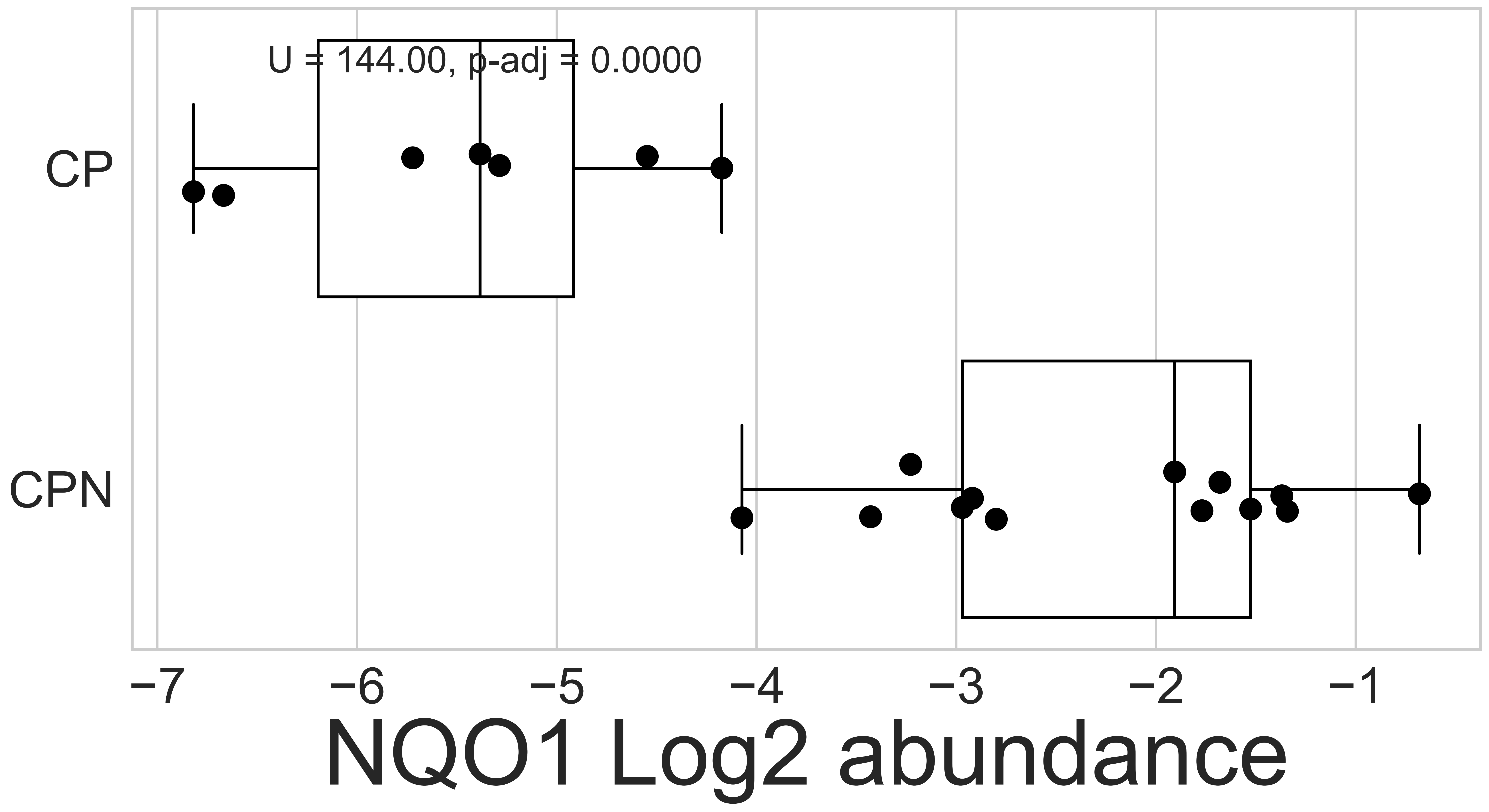
U = 144.00, p-adj = 0.0000
CP
CPN
−7
−6
−5
−4
−3
−2
−1
NQO1 Log2 abundance

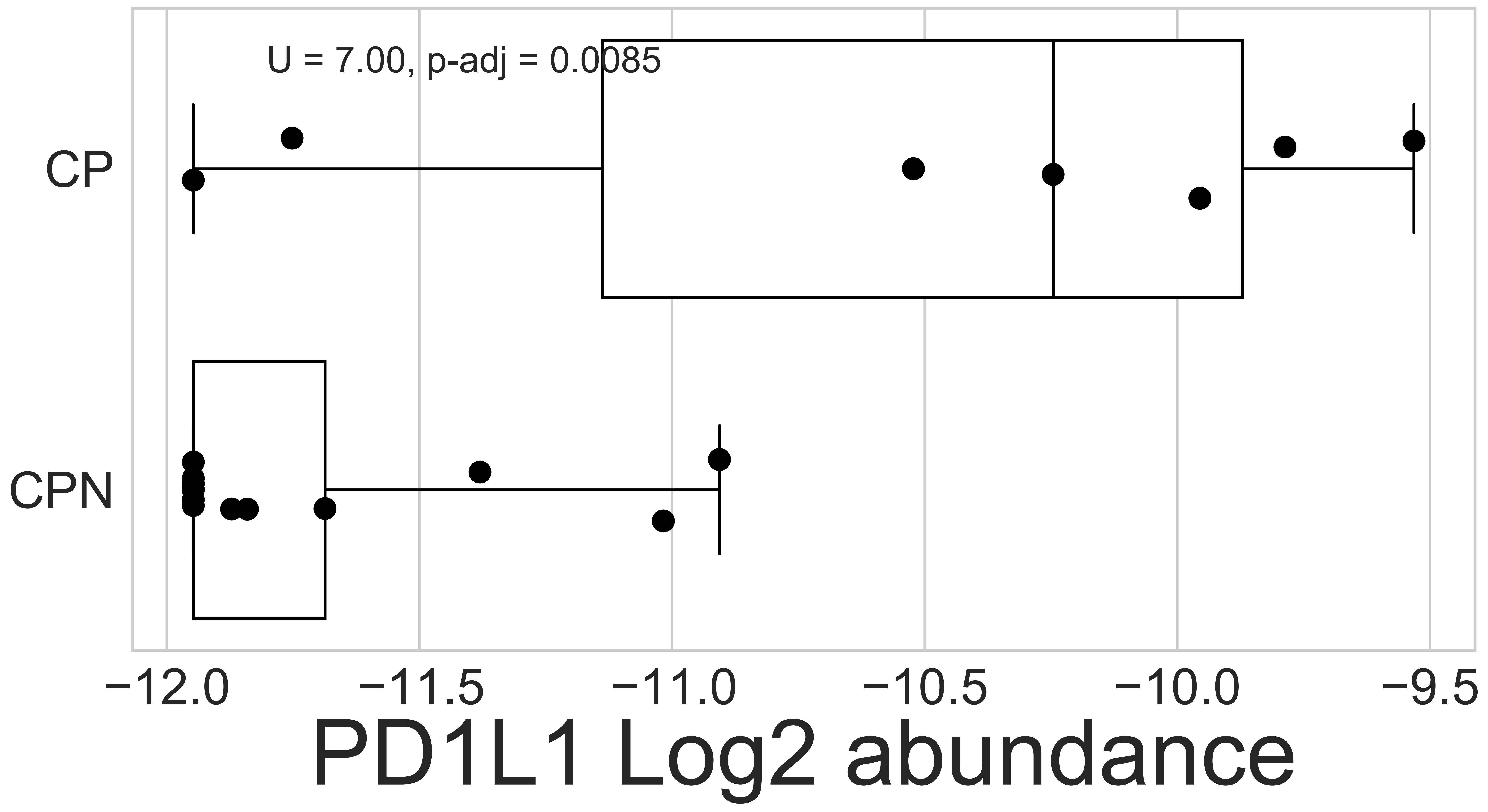
U = 7.00, p-adj = 0.0085
CP
CPN
−12.0
−11.5
−11.0
−10.5
−10.0
−9.5
PD1L1 Log2 abundance

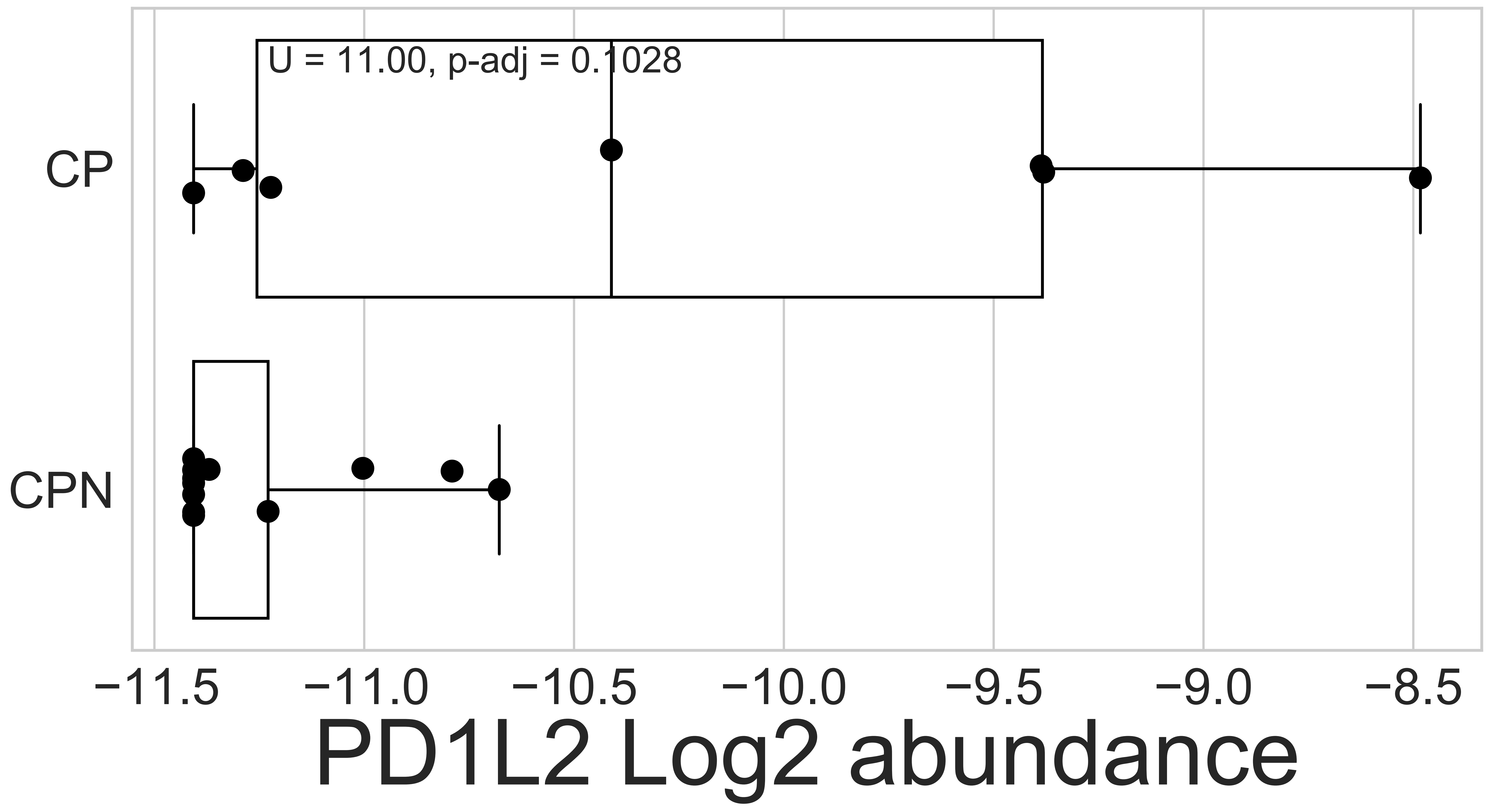
U = 11.00, p-adj = 0.1028
CP
CPN
−11.5
−11.0
−10.5
−10.0
−9.5
−9.0
−8.5
PD1L2 Log2 abundance

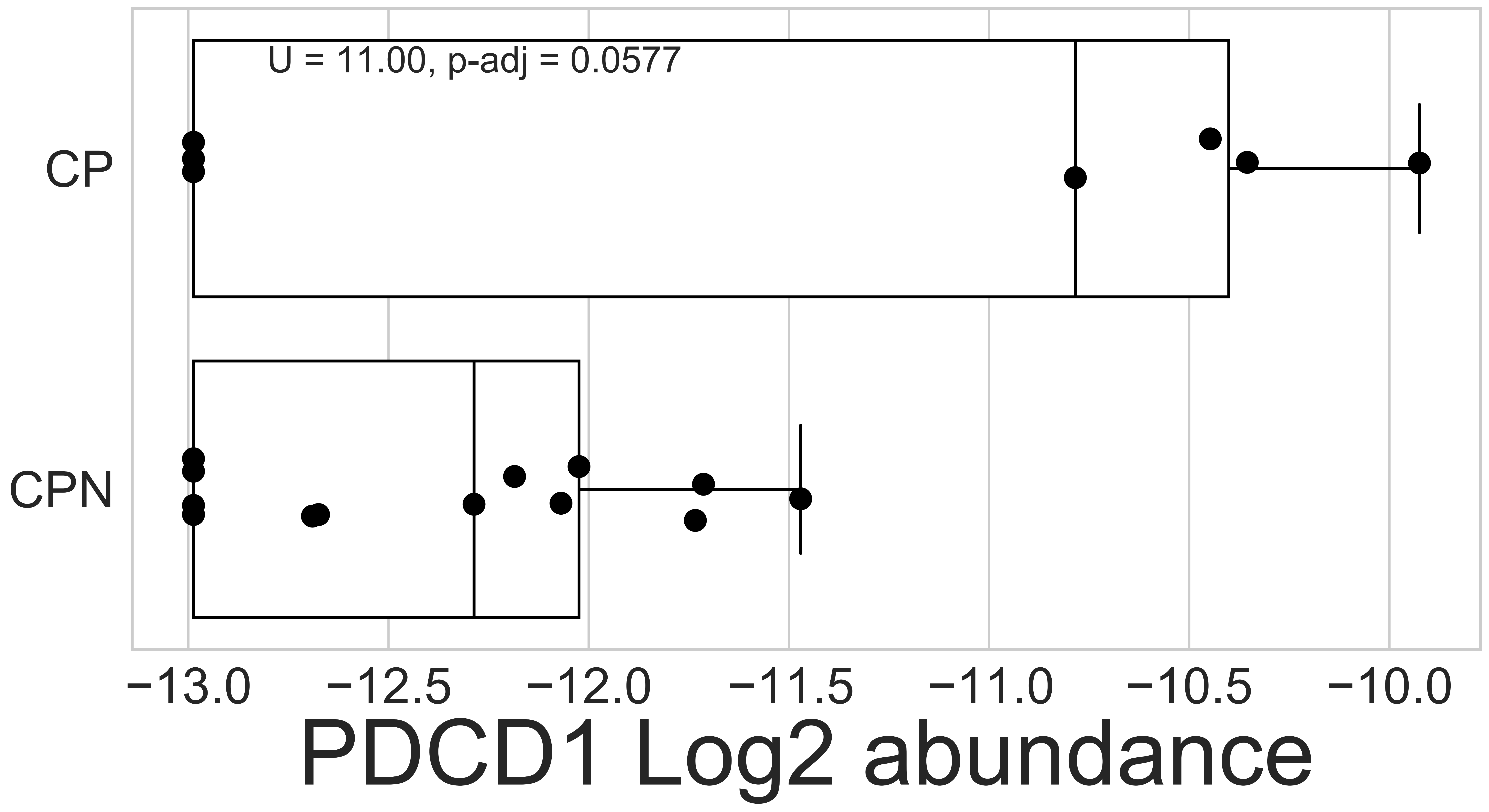
U = 11.00, p-adj = 0.0577
CP
CPN
−13.0
−12.5
−12.0
−11.5
−11.0
−10.5
−10.0
PDCD1 Log2 abundance

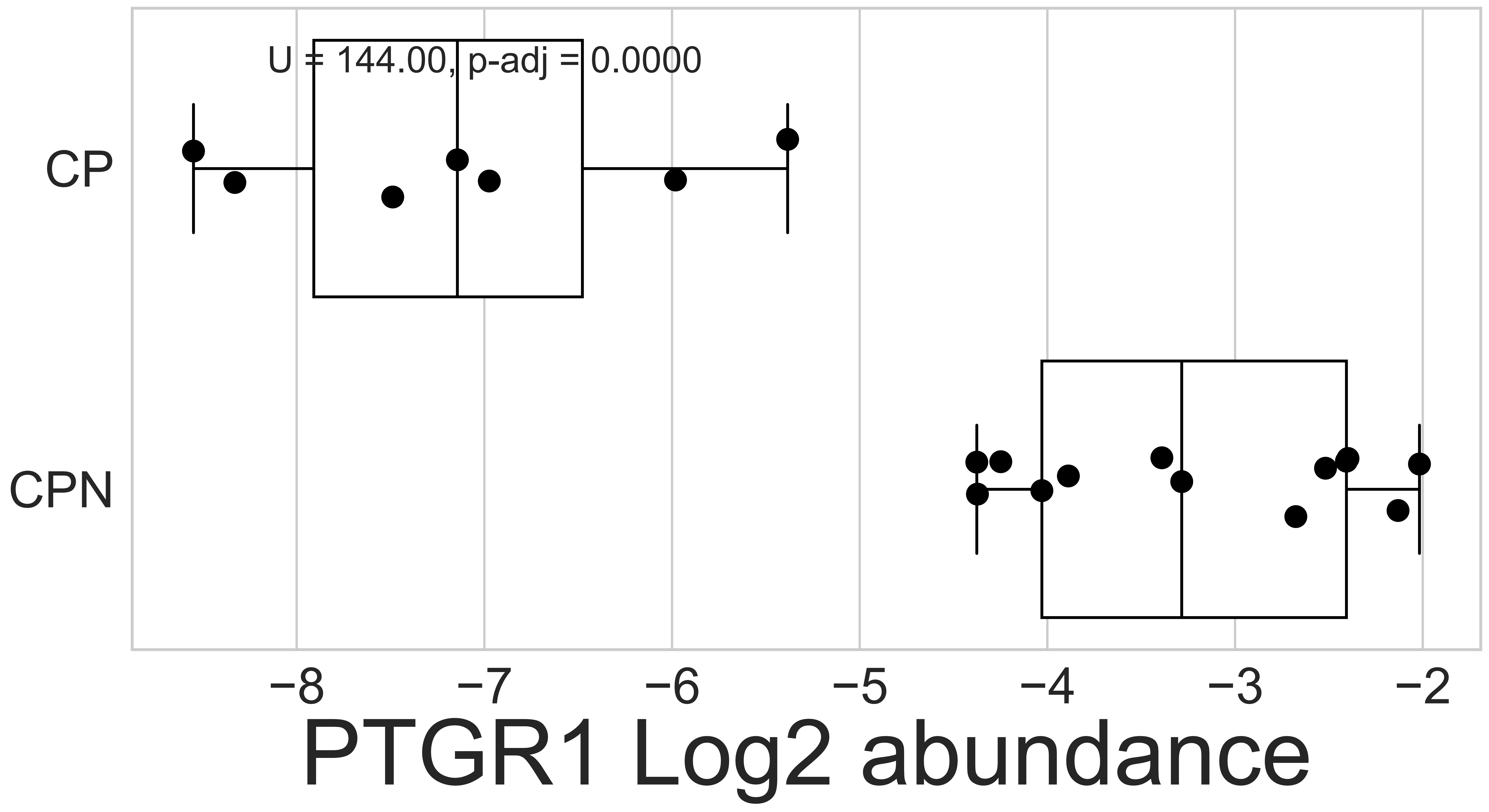
U = 144.00, p-adj = 0.0000
CP
CPN
−8
−7
−6
−5
−4
−3
−2
PTGR1 Log2 abundance

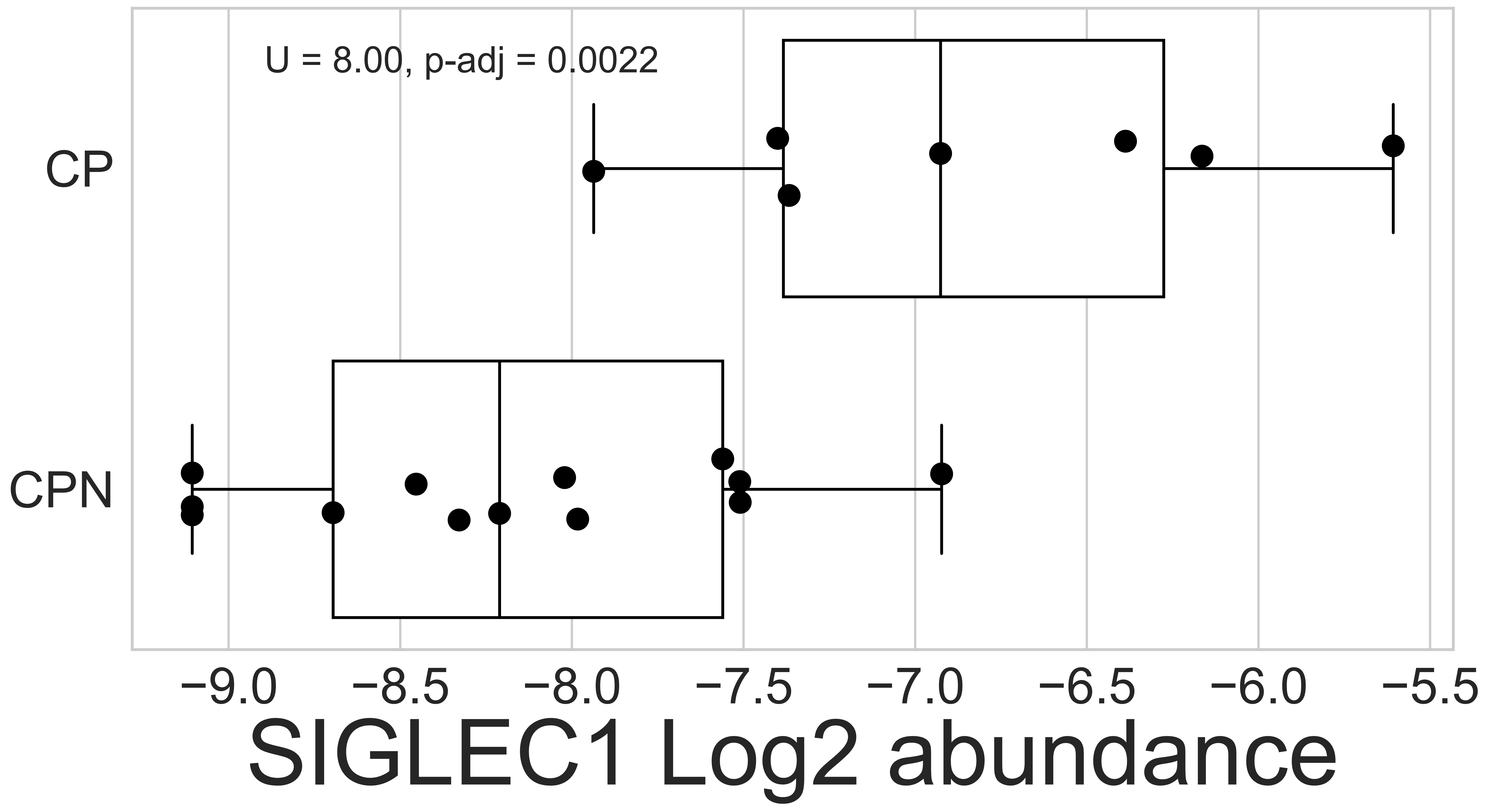
U = 8.00, p-adj = 0.0022
CP
CPN
−9.0
−8.5
−8.0
−7.5
−7.0
−6.5
−6.0
−5.5
SIGLEC1 Log2 abundance

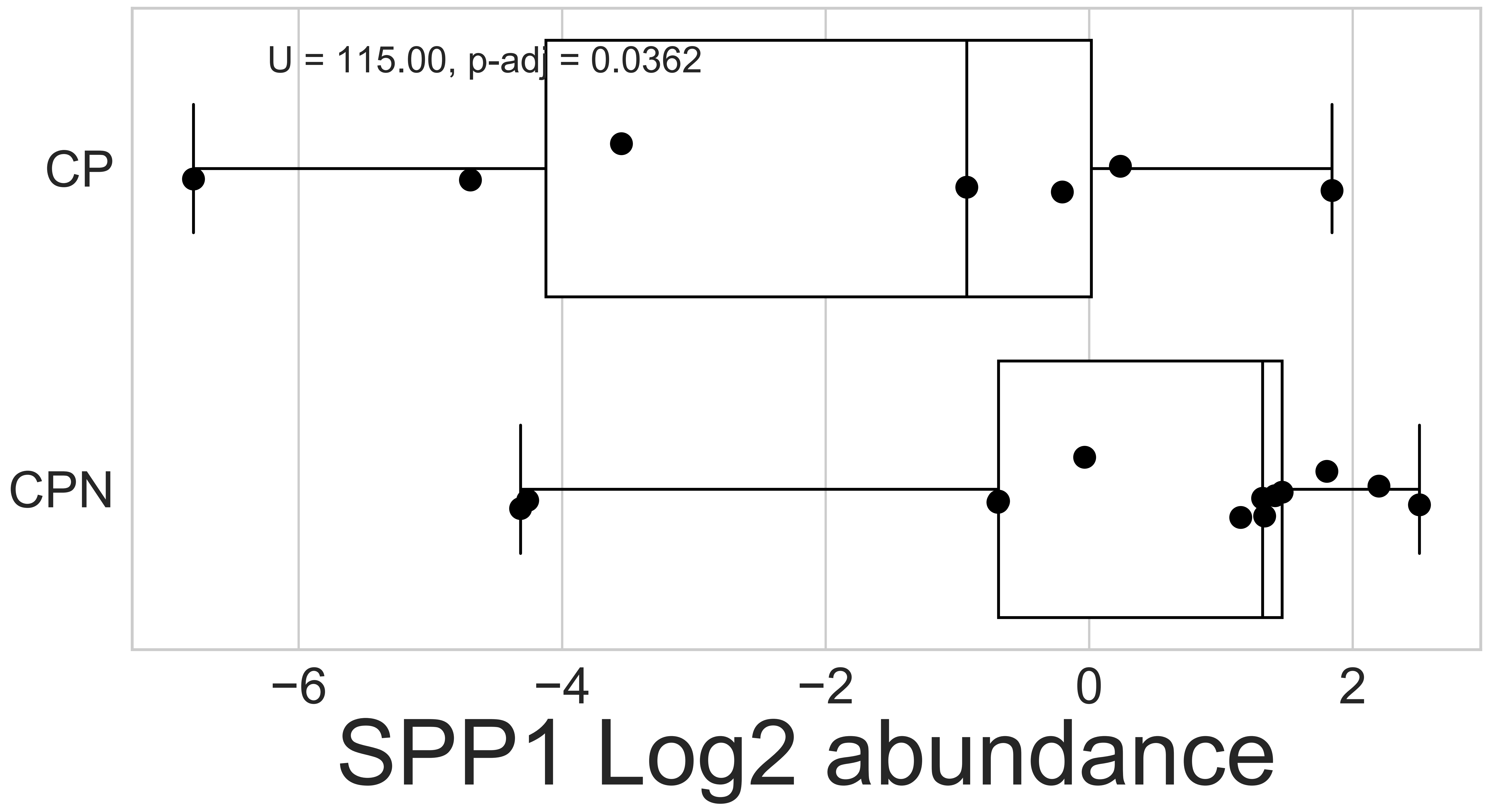

U = 115.00, p-adj = 0.0362
CP
CPN
−6
−4
−2
0
2
SPP1 Log2 abundance

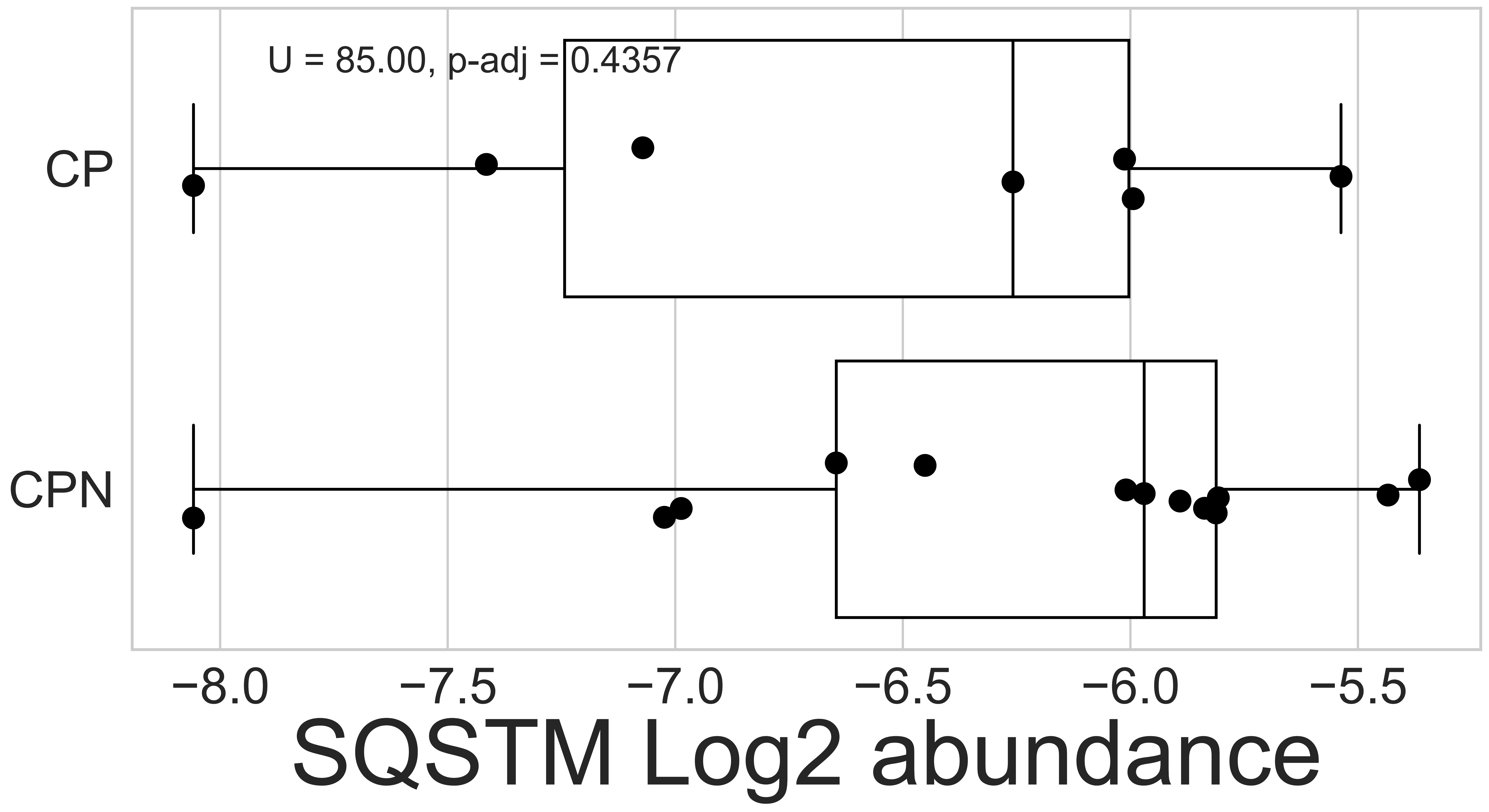
U = 85.00, p-adj = 0.4357
CP
CPN
−8.0
−7.5
−7.0
−6.5
−6.0
−5.5
SQSTM Log2 abundance

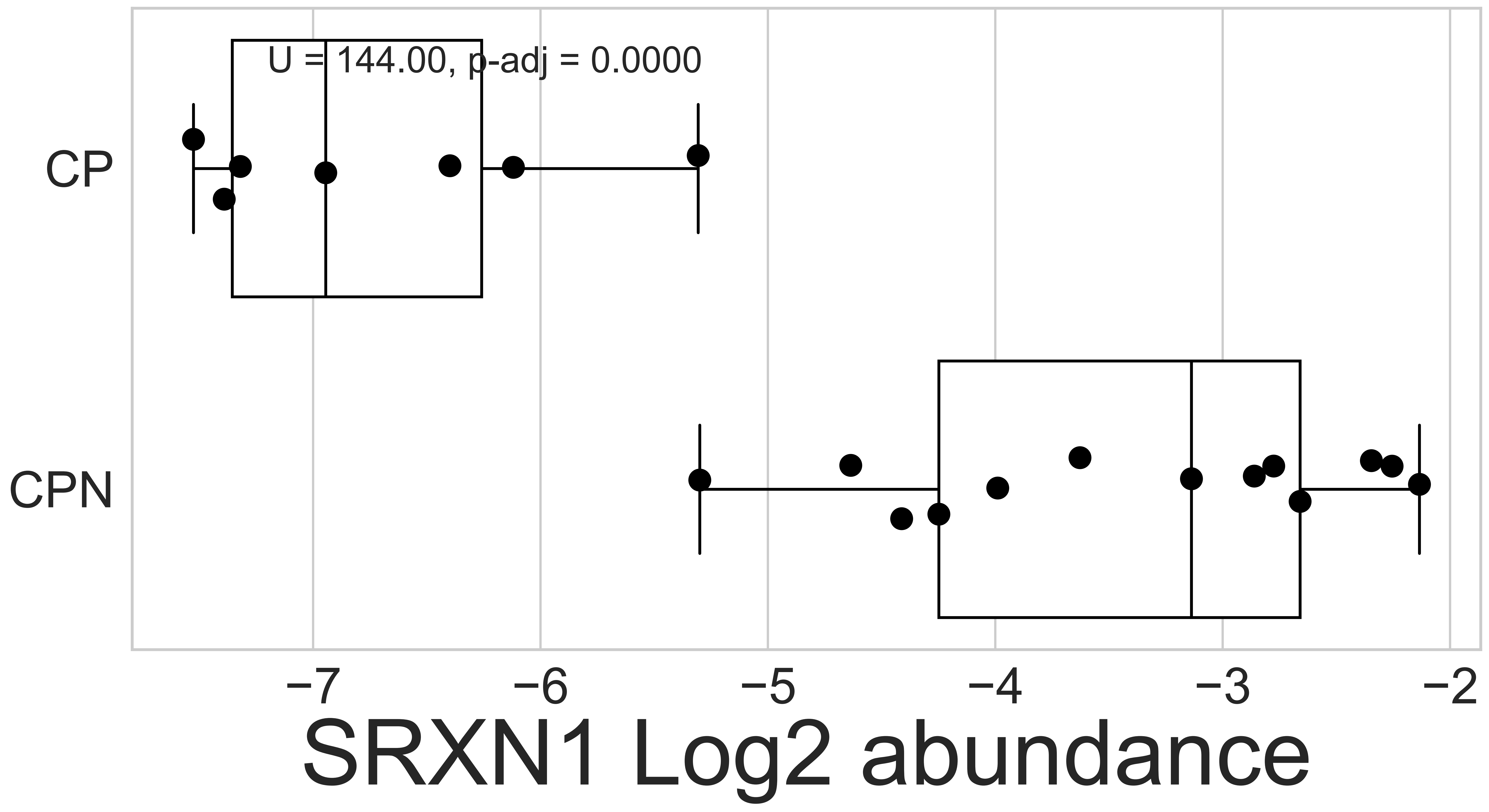
U = 144.00, p-adj = 0.0000
CP
CPN
−7
−6
−5
−4
−3
−2
SRXN1 Log2 abundance

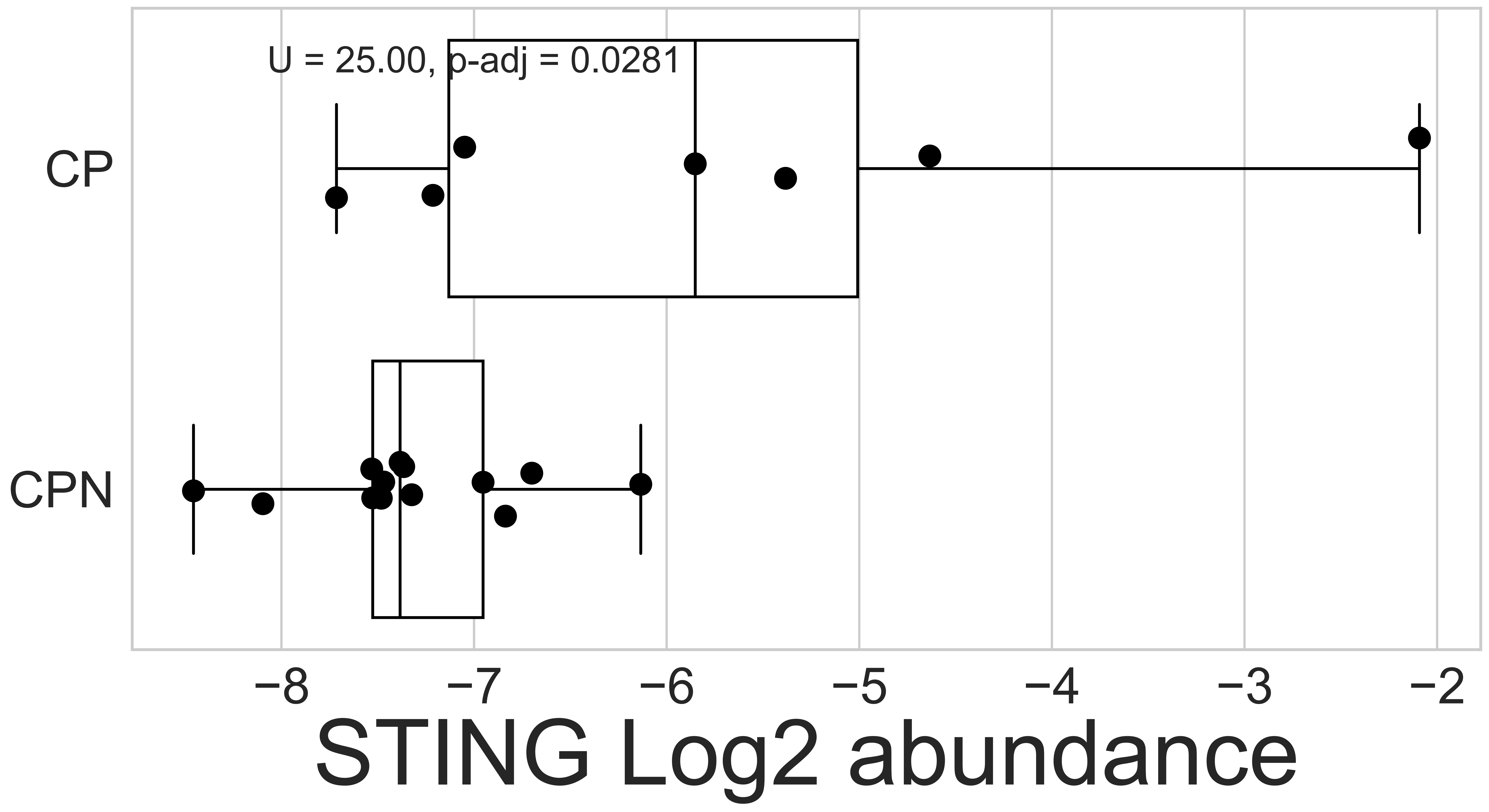

U = 25.00, p-adj = 0.0281
CP
CPN
−8
−7
−6
−5
−4
−3
−2
STING Log2 abundance

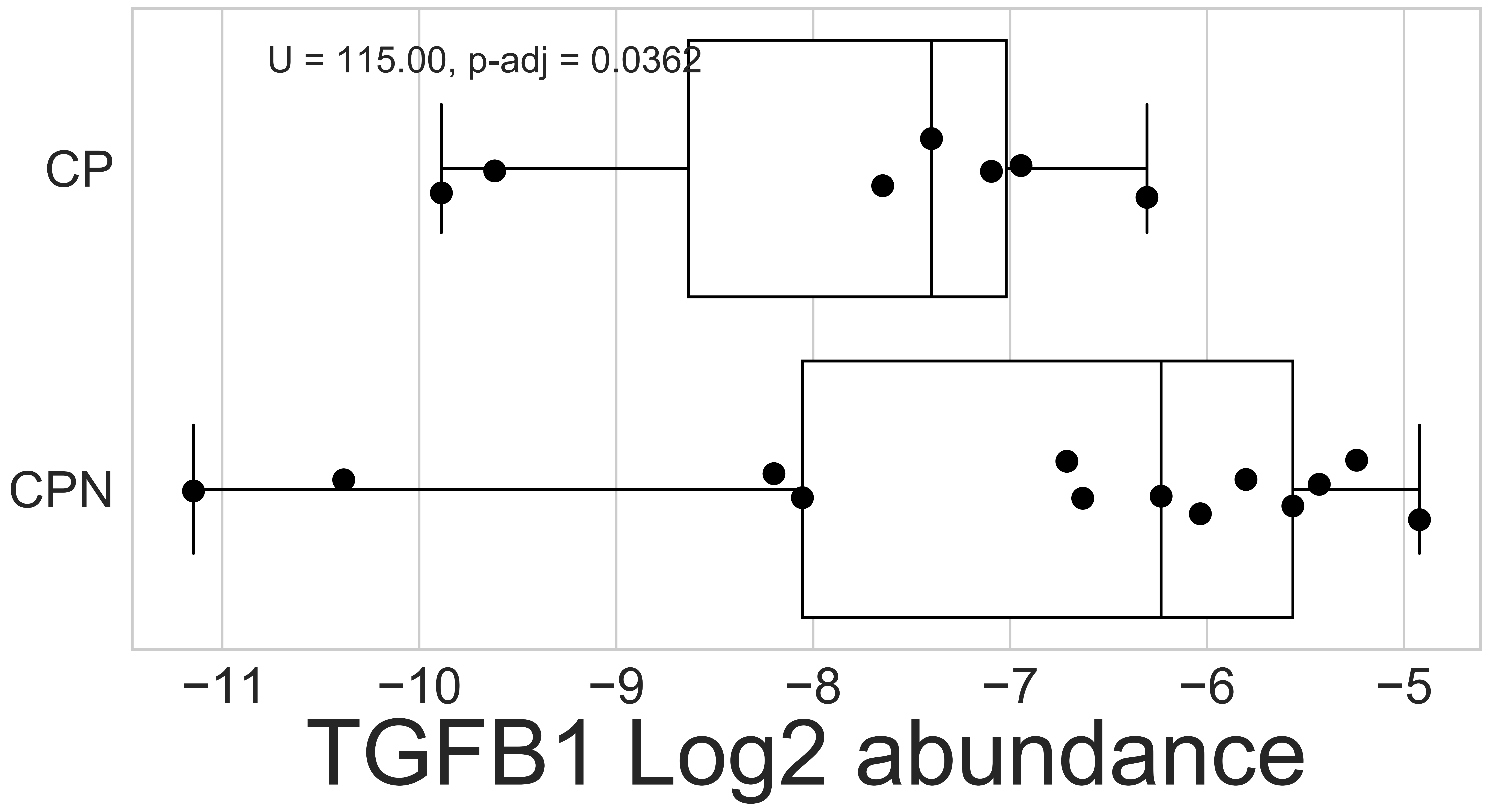

U = 115.00, p-adj = 0.0362
CP
CPN
−11
−10
−9
−8
−7
−6
−5
TGFB1 Log2 abundance

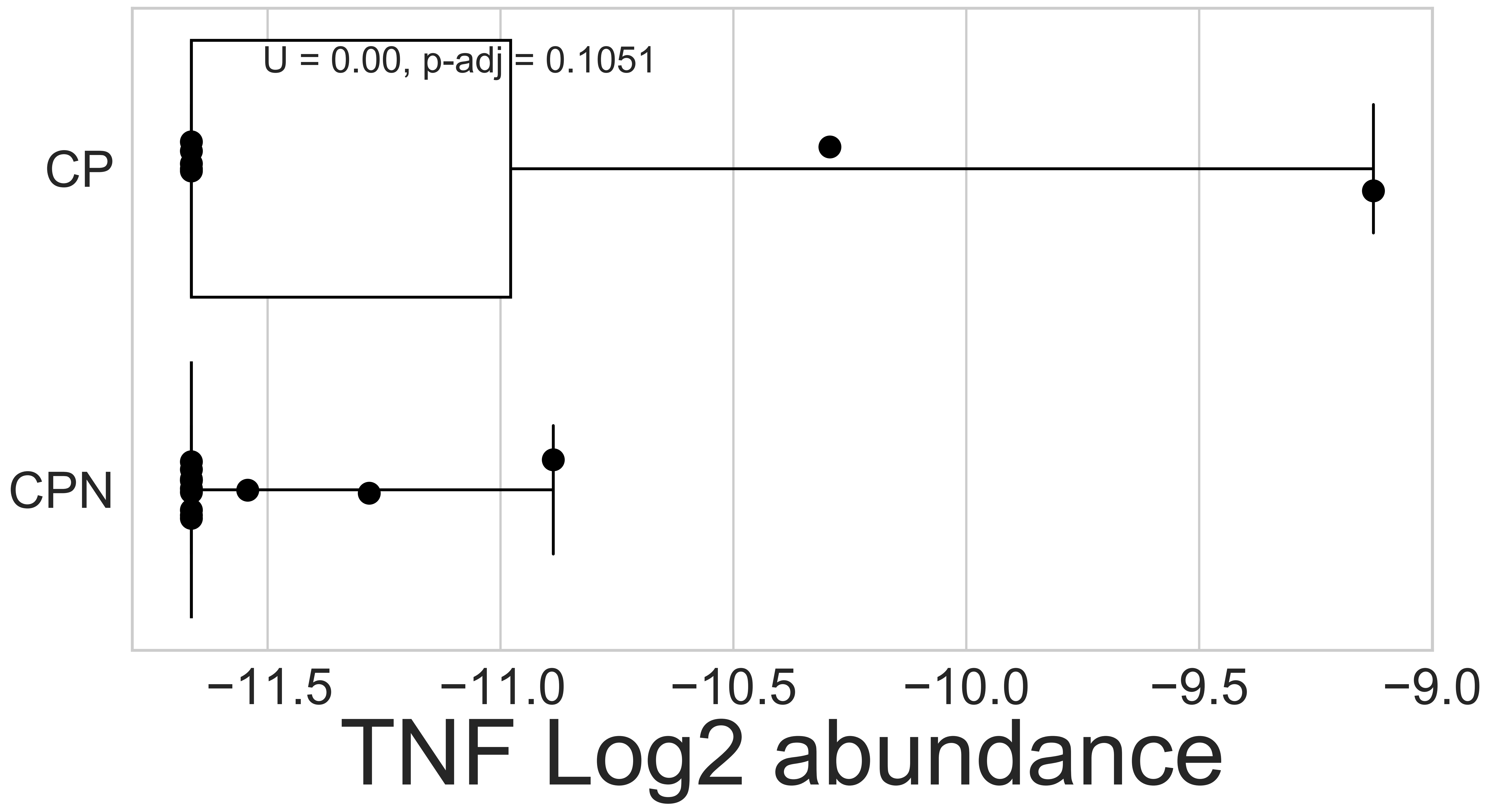
U = 0.00, p-adj = 0.1051
CP
CPN
−11.5
−11.0
−10.5
−10.0
−9.5
−9.0
TNF Log2 abundance

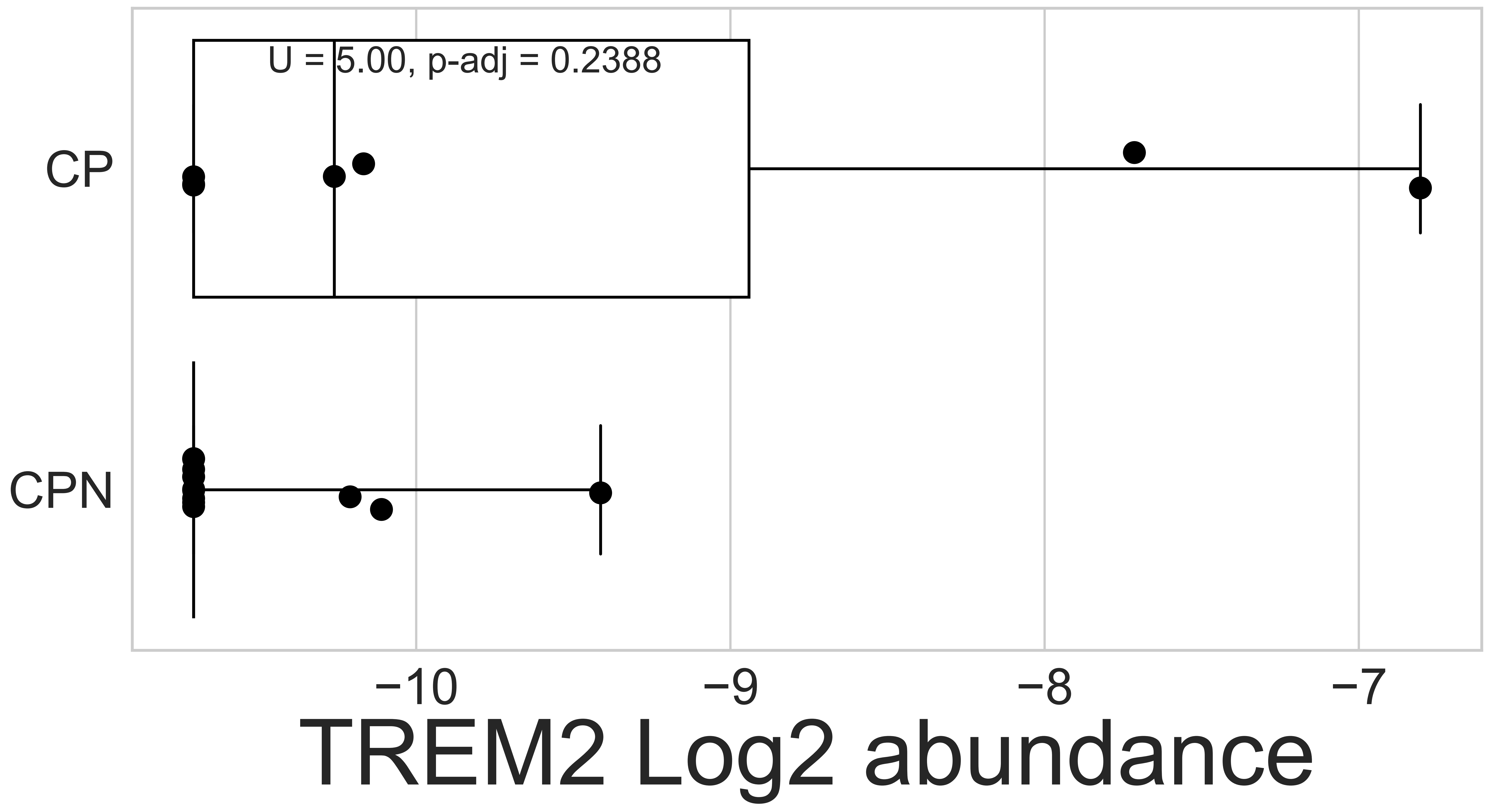
U = 5.00, p-adj = 0.2388
CP
CPN
−10
−9
−8
−7
TREM2 Log2 abundance

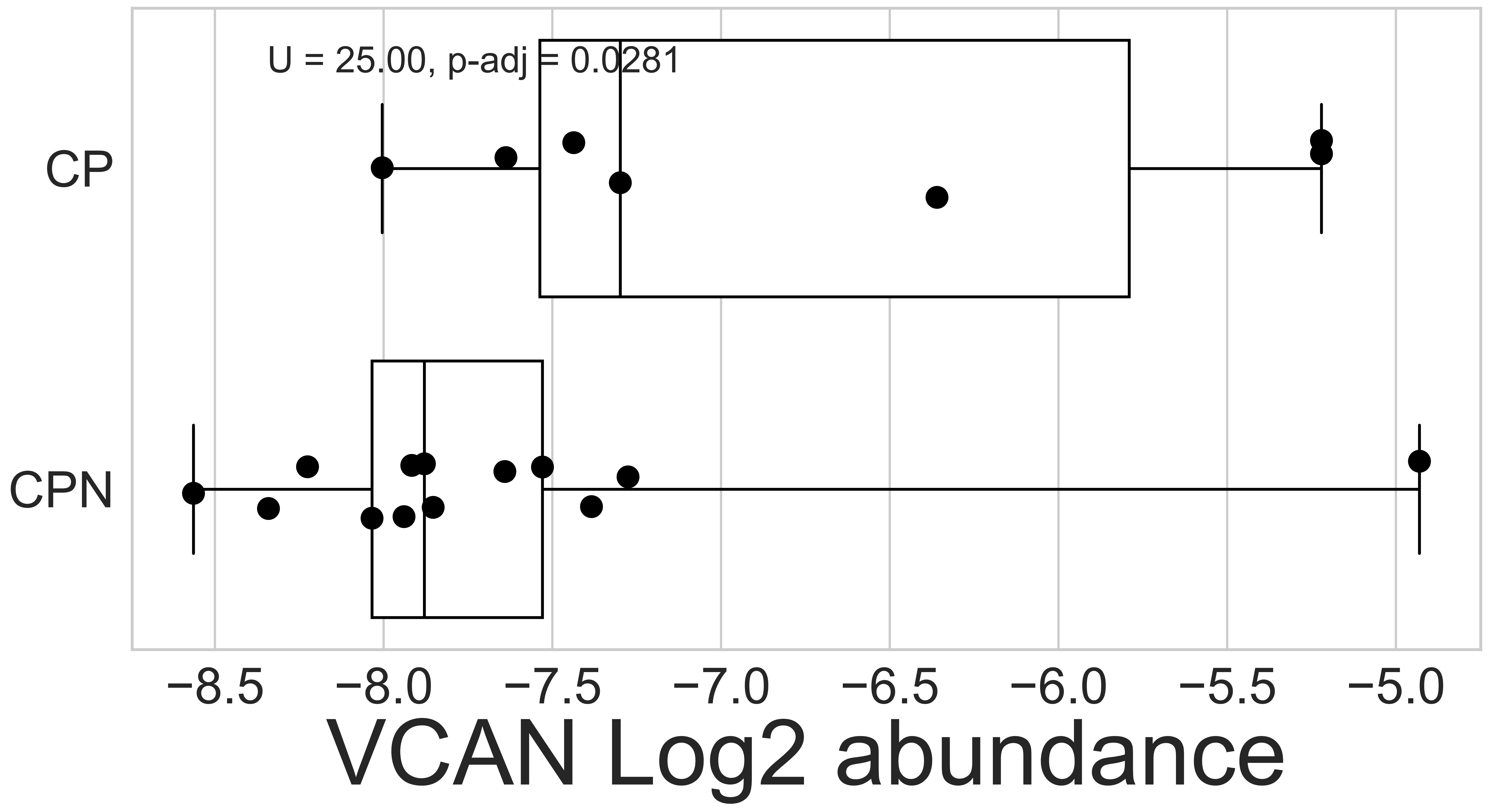
U = 25.00, p-adj = 0.0281
CP
CPN
−8.5
−8.0
−7.5
−7.0
−6.5
−6.0
−5.5
−5.0
VCAN Log2 abundance

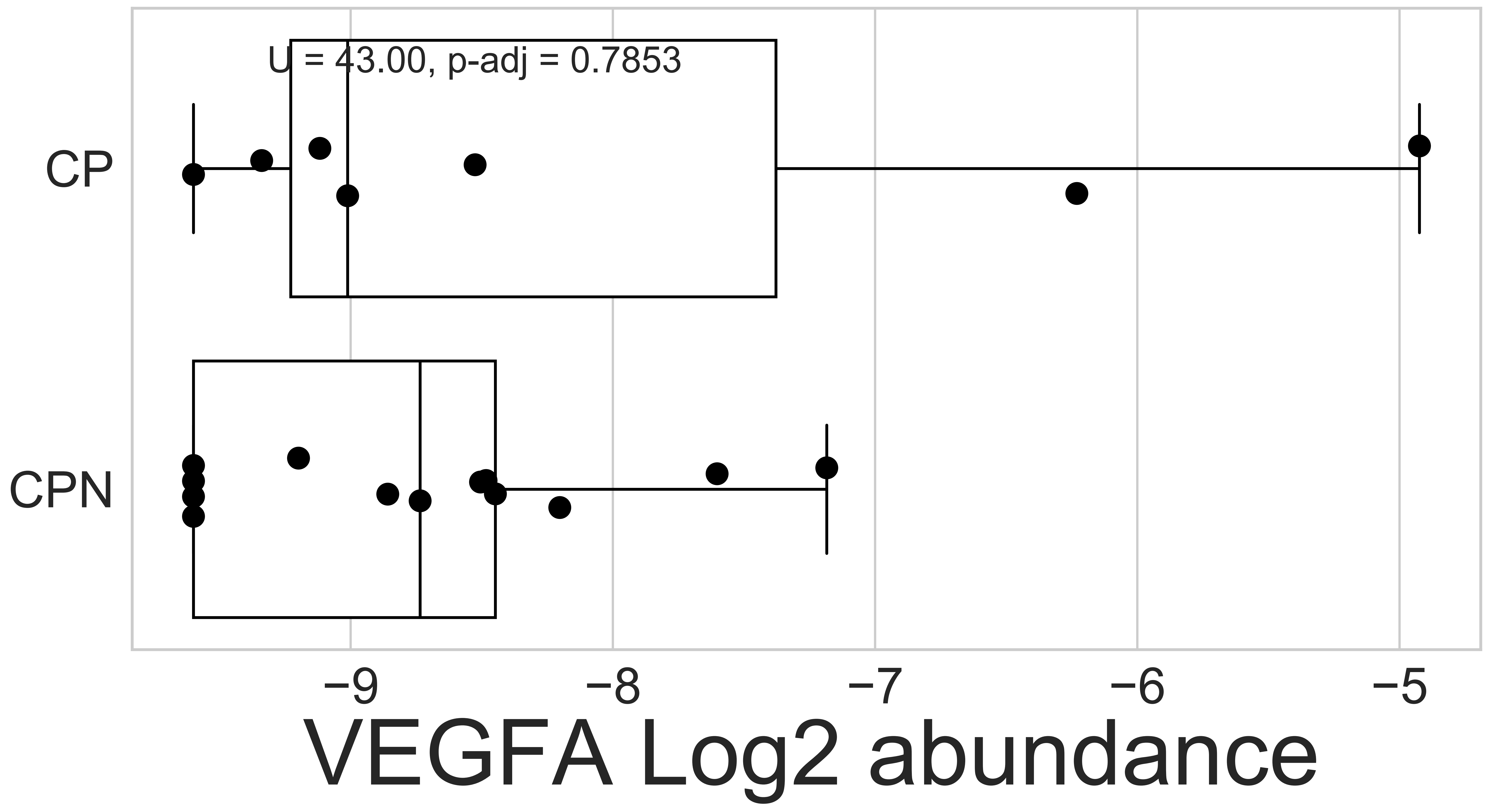
U = 43.00, p-adj = 0.7853
CP
CPN
−9
−8
−7
−6
−5
VEGFA Log2 abundance

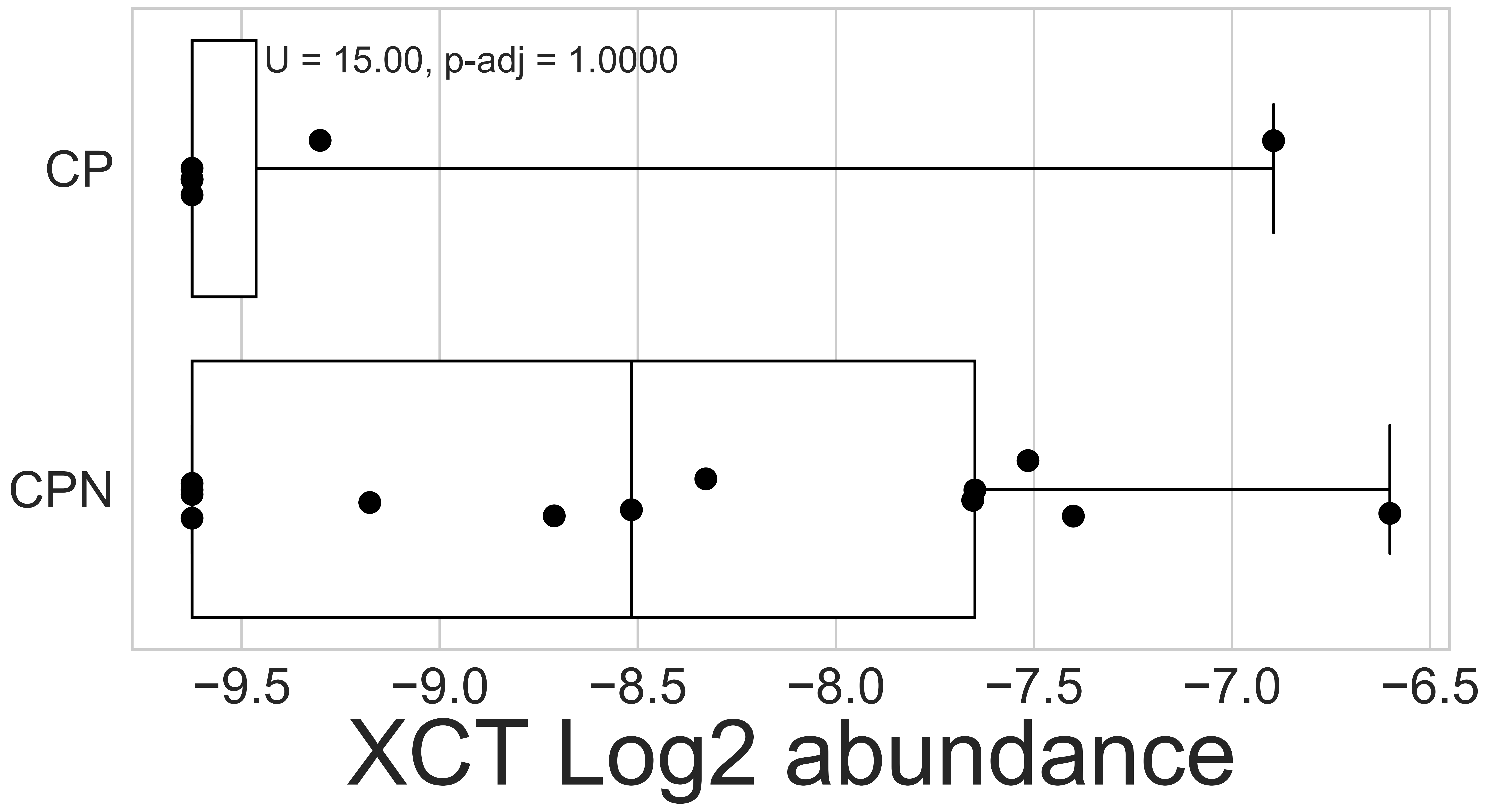
U = 15.00, p-adj = 1.0000
CP
CPN
−9.5
−9.0
−8.5
−8.0
−7.5
−7.0
−6.5
XCT Log2 abundance
